# Multimorbidity in Latin America and the Caribbean: a systematic review and meta-analysis

**DOI:** 10.1136/bmjopen-2021-050409

**Published:** 2021-07-23

**Authors:** Alessandra M Huaquía-Díaz, Tarick S Chalán-Dávila, Rodrigo M Carrillo-Larco, Antonio Bernabe-Ortiz

**Affiliations:** 1 Universidad Científica del Sur, Lima, Peru; 2 Department of Epidemiology and Bisotatistics, School of Public Health, Imperial College London, London, UK; 3 CRONICAS Centre of Excellence in Chronic Diseases, Universidad Peruana Cayetano Heredia, Lima, Peru; 4 School of Public Health and Administration, Universidad Peruana Cayetano Heredia, Lima, Peru

**Keywords:** epidemiology, public health, hypertension, diabetes & endocrinology

## Abstract

**Objective:**

To estimate the pooled prevalence of multimorbidity (≥2 non-communicable diseases in the same individual) among adults of the general population of Latin American and the Caribbean (LAC).

**Design:**

Systematic review and meta-analysis.

**Data sources:**

MEDLINE, Embase, Global Health, Scopus and LILACS up to 1 July 2020.

**Eligibility criteria for selecting studies:**

The outcome was the prevalence of multimorbidity. Reports were selected whether they enrolled adult individuals (age ≥18 years) from the general population.

**Data extraction and synthesis:**

Reviewers extracted relevant data and assessed risk of bias independently. A random-effects meta-analysis was conducted to report pooled prevalence estimates of multimorbidity; pooled estimates by pre-specified subgroups (eg, national studies) were also pursued.

**Results:**

From 5830 results, we selected 28 reports, mostly from Brazil and 16 were based on a nationally representative sample. From the 28 selected reports, 26 were further included in the meta-analysis revealing a pooled multimorbidity prevalence of 43% (95% CI: 35% to 51%; I^2^: 99.9%). When only reports with a nationally representative sample were combined, the pooled prevalence was 37% (95% CI: 27% to 47%; I^2^: 99.9%). When the ascertainment of multimorbidity was based on self-reports alone, the pooled prevalence was 40% (95% CI: 31% to 48%; I^2^: 99.9%); this raised to 52% (95% CI: 33% to 70%; I^2^: 99.9%) for reports including self-reported and objective diagnosis.

**Conclusions:**

Our results complement and advance those from global efforts by incorporating much more reports from LAC. We revealed a larger presence of multimorbidity in LAC than previously reported.

**PROSPERO registration number:**

CRD42020196177.

**Strengths and limitations of this study**

- Most of the analysed data came from Brazil preventing region representativity.
- High heterogeneity was present in the analysis, mainly due to different conditions included to define multimorbidity.
- Participant’s age seems to explain high heterogeneity as age range in studies is very wide.
- A bias due to self-reporting of conditions may underestimate the real burden of multimorbidity in Latin American and the Caribbean region.

## Introduction

The Academy of Medical Sciences defines multimorbidity as ‘the existence of two or more medical chronic conditions in a single individual’.^1^ Subjects with multimorbidity tend to increase healthcare utilisation and costs of primary and secondary care services^2^; also, multimorbidity has a subsequent impact on quality of life.^3^

In high-income countries, multimorbidity rates are heterogeneous but seem depend on individual’s age. Thus, in a cross-sectional study using the data set of medical practices in Scotland, the prevalence of multimorbidity was 23% using a list of 40 conditions, and was present mainly in older people.^4^ Moreover, multimorbidity seems to be increasing in low-income and middle-income countries (LMIC), where data are yet scarce.^5^ The increase of life expectancy in Latin American and the Caribbean (LAC) has been associated with greater incidence of non-communicable conditions,^6^ with the consequent emergence of multimorbidity.

Multimorbidity prevalence has been explored and summarised in some systematic reviews around the world^7 8^ and results ranged from 5% to 98%; nevertheless, their results were mainly informed by data from high-income countries. In a relatively recent systematic review,^9^ 31 LMIC were included with a prevalence of multimorbidity of 30%, compared with an estimate of 38% in high-income countries. However, only nine studies from the LAC region were included. Moreover, pooled estimates by region were not elucidated, preventing to have appropriate indicators of the burden of multimorbidity in this region. A more recent systematic review has reported a pooled prevalence of multimorbidity in LMIC between 3% and 90%, with almost 80% of the studies being from Brazil, China, South Africa, India, Mexico and Iran.^10^

The lack of evidence about multimorbidity may have important consequences for research, public health and clinical management in LAC region. For example, multimorbidity was not appropriately defined up to 2018; in addition, whether estimates depend on sex or setting characteristics (ie, rural vs urban areas) should be also studied. Moreover, the need of surveillance systems to assess multimorbidity may be elucidated as these estimates have not been estimated in LAC region. Thus, from the public health perspective may not be easy to take appropriate decisions or implement adequate strategies to tackle the problem of multimorbidity.

As a result, we aimed at providing robust evidence about multimorbidity prevalence estimates in LAC region through a systematic review and meta-analysis of population-based surveys. These results evidence may help to guide interventions and policies so that they can focus on the most pressing frequent multimorbidity phenotypes in LAC.

## Methods

### Protocol

This systematic review was registered in PROSPERO. We aimed to identify the population-based prevalence of multimorbidity in LAC, and to study whether this prevalence varies by multimorbidity definitions, sex and urban–rural settings.

### Eligibility criteria

Reports were selected whether they enrolled adult individuals (age ≥18 years) from the general population. We focused on LAC populations; therefore, we excluded studies with LAC individuals in countries outside the LAC region, and studies with only foreign subjects in LAC nations. Population-based studies were defined as those following a random sampling approach, and such sample was taken from the general population. On the contrary, studies addressing specific populations (eg, pregnant women), those with individuals with specific conditions (eg, people with hypertension) or subjects with specific risk factors (eg, obese or alcohol disorders) were excluded.

The outcome of interest was the prevalence of multimorbidity, defined as the existence of ≥2 chronic conditions in the same person.^1^ Other different definitions of multimorbidity were considered in this review (eg, ≥3, ≥4 or ≥5 conditions) as the current definition (≥2 conditions) is relatively recent. In addition, the presence of chronic conditions could have been measured, self-reported or a combination of these approaches.

### Information sources

The search was conducted on 10 January 2020 and then updated on 1 July 2020. We used Ovid search engine, comprising MEDLINE, Embase and Global Health databases; and in parallel, we also searched Scopus and LILACS. In all of these, searching was carried out without time or language restriction. The search strategy and terms used is detailed in online supplemental tables 1–3.

10.1136/bmjopen-2021-050409.supp1 Supplementary data

### Study selection

Results from each search engine were downloaded and saved in EndNote where duplicates were removed. After that, information was transferred to Rayyan, an open access online tool for systematic reviews.^11^ Titles and abstracts were reviewed by two researchers in an independent way, and disagreements were solved by a third party. After this screening phase, selected reports were downloaded and independently studied in detail by two researchers, and similarly, discrepancies were solved by a third party. Finally, selected studies were examined again to check for data duplication, that is, different reports that used the same data (eg, multiple reports based on the same underlying data). In this case, the paper with more information or the one with the largest sample size was included in the review and meta-analysis.

### Data collection

An extraction template form was built by the authors and tested with a random sample of selected studies. After starting data collection, the form was not further modified. This form included study characteristics: study design, country, if it was a nationally representative sample, sample size, year of data collection, age range, age mean, proportion of women and if it was urban, rural or both. The extraction form also collated the definition of multimorbidity used, self-reported or a combination of self-reported and measured, the number and a list of chronic conditions studied, and the prevalence of multimorbidity (overall, by sex, and by rural or urban settings).

### Risk of bias of individual studies

Risk of bias of selected studies was evaluated using the Newcastle-Ottawa Quality Assessment Scale adapted for cross-sectional studies as in a previous report.^12^ This tool is focused on selection process (representativeness, sample size and non-respondents), and the assessment of the outcome (independent blind assessment, self-report or not description). The items of this scale were implemented in an Excel spreadsheet and assessed independently by two reviewers; discrepancies were solved by a third party.

### Summary measures

Our systematic review followed the Preferred Reporting Items for Systematic Review and Meta-Analysis toolkit (see checklist in online supplemental table 4). We presented a qualitative and quantitative summary. The qualitative summary described the characteristics of the study (as listed above), whereas the quantitative summary explored pooled prevalence estimates.

Statistical analyses were performed using Stata V.16 for Windows (StataCorp). The ‘metaprop’ command attains a pooled estimate as a weighted average, by fitting a logistic-normal random-effect model without covariates, but random intercepts.^13^ After that, the pooled estimate was calculated using the Freeman-Tukey arcsine transformation as suggested in literature.^14^

Because the selected studies were different in nature, scope (eg, national surveys vs community/subnational studies, or urban vs rural settings) and sample size, we conducted random-effects meta-analysis for comparing estimates in specific subgroups (eg, national studies only). In addition, stratified meta-analysis (eg, by sex, and by urban/rural settings) was pursued. Sensitivity analyses were also conducted that focused on age by including studies with individuals aged 50+ and 60+ years, and also with studies whose data was collected from 2010 and onwards, as well as 2015 and onwards. Besides, as many of the studies were conducted in Brazil, a comparison of pooled estimated between Brazil and other countries together was also carried out.

Finally, meta-regression was also conducted as a high level of heterogeneity was expected. Meta-regression command in Stata investigates if between-study heterogeneity can be explained by one or more of the variables included in the review.

### Patient and public involvement

Patients or the public were not involved in the design, or conduct, or reporting, or dissemination plans of our research.

## Results

### Selection process

**Figure 1:**
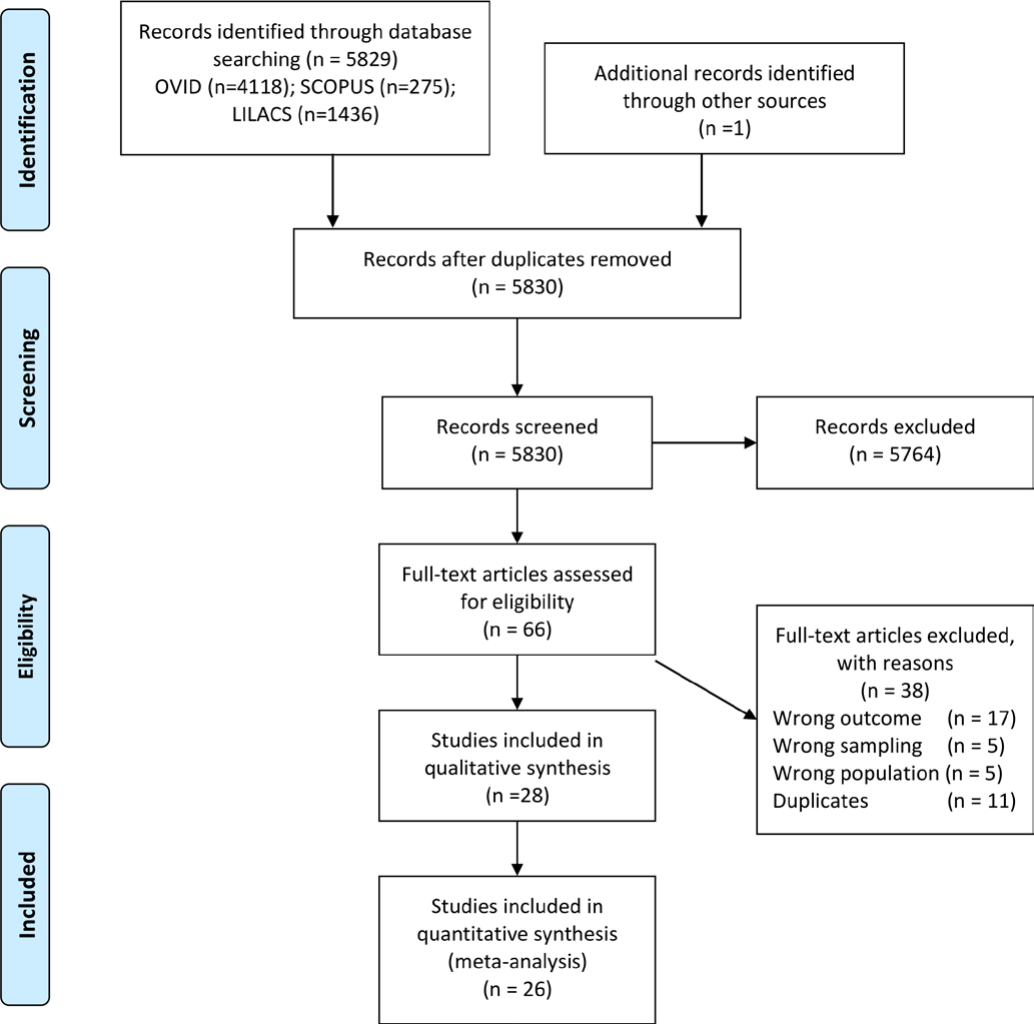
Flowchart of studies included in the systematic review.

The search strategy yielded 5830 titles and abstracts after removing duplicates (see figure 1); of these 66 were studied in detail and 28 reports met the inclusion criteria. For quantitative analyses, 26 reports were included (2 were excluded as the definition of multimorbidity was ≥5 chronic conditions), representing information for 12 LAC countries.

### General characteristics of selected studies

As shown in table 1, Brazil contributes with a total of 20 data points^15–34^; Mexico with 4 data points^23 35–37^; Peru with 3 data points^36 38 39^; Colombia with 2 data points^23 40^; and Argentina,^41^ Cuba,^36^ Dominican Republic,^36^ El Salvador,^23^ Jamaica,^23^ Panama,^23^ Puerto Rico^36^ and Venezuela^36^ with 1 data point each. On the other hand, only 16 studies analysed a nationally representative sample (table 1).

**Table 1:** Characteristics of the studies included in the systematic review

First author	Country	Study design	Data collected in	Sample size	Definition of multimorbidity	Age range (years)	Age mean (years)	% women	% urban
Andrade^17^	Brazil	Cross-sectional	1994–1995	1464	≥2 (self-reported)	18+	–	57.4	100
Boing^19^	Brazil	Cross-sectional	2009–2010	1720	≥2 (self-reported)	20–59	–	55.6	100
De Souza Santos Machado^22^	Brazil	Cross-sectional	2005	377	≥2 (self-reported)	40–65	–	100	100
Aguiar^15^	Brazil	Cross-sectional	2011	622	≥2 (self-reported)	50+	64.1	100	100
Nunes^29^	Brazil	Cross-sectional	2008	1593	≥2 and ≥3 (self-reported)	60+	–	62.8	100
Agrawal^35^	Mexico	Cross-sectional	2007–2010	2725	≥2 (self-reported)	18+	63.1	61.8	73.7
Nunes^26^	Brazil	Cross-sectional	2012	2927	≥2 and ≥3 (self-reported)	20+	45.7	58.9	100
Valadares^33^	Brazil	Cross-sectional	2012–2013	736	≥2 (self-reported)	45–60	52.5	100	100
Bustos-Vazquez^37^	Mexico	Cross-sectional	2012	7967	≥2 (self-reported)	60+	69.3	53.4	–
Cavalcanti^42^	Brazil	Cross-sectional	2010–2011	676	≥2 (self-reported)	60+	70.0	54.6	69.4
Nunes^27^	Brazil	Cross-sectional	2013	60 202	≥2 and ≥3 (self-reported)	18+	43.7	55.1	86.5
Nunes^28^	Brazil	Cross-sectional	2008	1593	≥2 and ≥3 (self-reported)	60+	–	62.8	–
Olivares^41^	Argentina	Cross-sectional	2014–2015	1044	≥2 (self-reported)	18+	43.0	65	–
Taype-Rondan^39^	Peru	Longitudinal	2013–2014	2433	≥2 (self-reported and measured)	35+	57.2	51.3	51.3
Amaral^16^	Brazil	Cross-sectional	2010	264	≥2 (self-reported)	60+	–	61	100
Araujo^18^	Brazil	Cross-sectional	2015	4001	≥2 and ≥3 (self-reported)	18+	–	52.7	86.8
Camargo-Casas^40^	Colombia	Cross-sectional	2012	2000	≥2 (self-reported)	60+	71.1	63.4	–
Costa^20^	Brazil	Cross-sectional	2014	1451	≥2 and ≥3 (self-reported and measured)	60+	–	63	–
Nunes^25^	Brazil	Longitudinal	2015–2016	9412	≥2 and ≥3 (self-reported)	50+	62.9	54	84.7
Bao^36^	Cuba	Longitudinal	2003–2007	2944	≥2 (self-reported and measured)	65+	73.9	64	–
	Dominican Republic			2011			74.5	65	–
	Puerto Rico			2009			75.2	67	–
	Venezuela			1965			71.5	63	–
	Peru			1933			74.2	60	–
	Mexico			2003			73.7	64	–
Macinko^23^	Brazil	Cross-sectional	2013–2014	1486	≥2 (self-reported)	18+	–	51.1	–
	Colombia			1485			–	51.1	–
	El Salvador			1460			–	52.8	–
	Jamaica			1480			–	52.6	–
	Mexico			1492			–	51.4	–
	Panama			1475			–	51.6	–
Miranda^38^	Peru	Longitudinal	2010–2012	2890	≥2 (self-reported and measured)	35+	55.2	51	50.9
Petarli^31^	Brazil	Cross-sectional	2016–2017	790	≥2 and≥3 (self-reported)	18–59	–	47.7	–
Tavares^32^	Brazil	Cross-sectional	2012	1691	≥2 (self-reported)	60+	72.5	63.7	100
Wang^34^	Brazil	Cross-sectional	2005–2007	2713	≥2 (self-reported)	18–64	–	52,4	–
Montes^24^	Brazil	Cross-sectional	2014	1336	≥5 (self-reported)	60+	–	63.1	–
Padilha Pereira^30^	Brazil	Cross-sectional	2014	1426	≥5 (self-reported)	60+	–	63	–
Da Silva Almeida^21^	Brazil	Cross-sectional	2016	850	≥2 (self-reported and measured)	18+	–	61.2	–

Data from 37 country-level points were used to calculate pooled estimates using a definition of multimorbidity of ≥2 chronic conditions. Only one country (Brazil) used an additional definition of multimorbidity (ie, ≥3 chronic conditions) in eight different reports. Fourteen reports had information to calculate estimates for urban settings,^15 17–19 22 25–27 29 33 35 38 39 42^ whereas seven had information from rural areas.^18 25 27 35 38 39 42^

Finally, the number of conditions evaluated to define multimorbidity ranged from 5 to 29, with a mean of 12.3 (SD: 5.7) conditions (online supplemental table 5). Hypertension and type 2 diabetes were the only conditions included in all the definitions of multimorbidity.

### Synthesis of results

**Figure 2:**
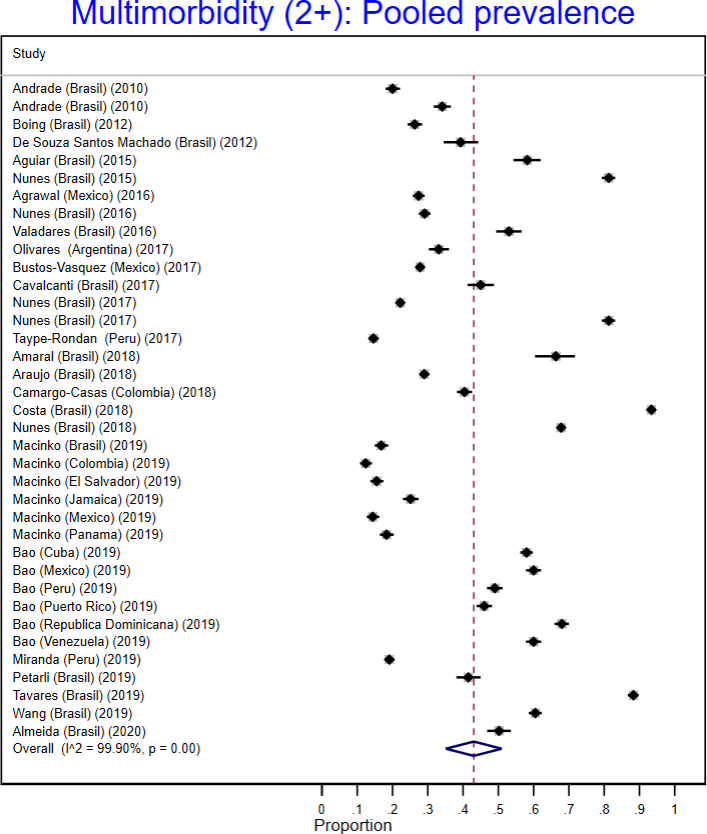
Forest plot of the pooled prevalence of multimorbidity defined as ≥2 chronic conditions.

The meta-analysis included 26 studies with information with a pooled estimate of multimorbidity defined as ≥2 chronic conditions of 43% (95% CI: 35% to 51%; I^2^: 99.9%; see figure 2); whereas the pooled estimate for multimorbidity using ≥3 chronic conditions was 40% (95% CI: 22% to 57%; I^2^: 99.9%).

As many of the studies were from Brazil, the pooled estimate of multimorbidity (≥2 chronic conditions) for this country was 50% (95% CI: 37% to 63%; I^2^: 99.9%), whereas the pooled estimate for other countries together was 35% (95% CI: 26% to 43%; I^2^: 99.7%). Nationally representative samples had a pooled estimate of multimorbidity of 37% (95% CI: 27% to 47%; I^2^: 99.9%), while this estimate was 48% (95% CI: 35% to 61%; I^2^: 99.9%) for non-nationally representative samples. Similarly, when multimorbidity was assessed as self-reported, pooled prevalence was 40% (95% CI: 31% to 48%; I^2^: 99.9%), but the prevalence was 52% (95% CI: 33% to 70%; I^2^: 99.9%) for those which have objectively measured and self-reported chronic conditions.

On the other hand, when analysis was conducted using studies which data was collected from 2010 and onwards, pooled prevalence of multimorbidity was 48% (95% CI: 34% to 61%; I^2^: 99.9%), whereas this estimate was 44% (95% CI: 24% to 65%, I^2^: 99.8%) for studies from 2015 and onwards. This approach was used because time can have an impact on estimations due to the health transition in LMIC. Similarly, when assessing only studies including subjects of 50 years and over, the pooled prevalence of multimorbidity was 62% (95% CI: 51% to 73%; I^2^: 99.9%), and there was no variation when the pooled prevalence was estimated for subject of 60 years and over: 62% (95% CI: 49% to 75%; I^2^: 99.9%).

In stratified analyses, pooled prevalence of multimorbidity was 38.9% (95% CI: 28.6% to 49.1%; I^2^: 99.7%) for men and 50.5% (95% CI: 38.3% to 62.7%; I^2^: 99.8%) for women. These estimates were 38.1% (95% CI: 26.1% to 50.1%; I^2^: 87.3%) and 24.7% (95% CI: 12.5% to 36.8%; I^2^: 50.8%) for urban and rural dwellers, respectively (see online supplemental figures 1–4).

In meta-regression analysis, the number of chronic conditions defining multimorbidity was strongly associated with heterogeneity (β: 0.02 per additional condition, p<0.001). Similarly, mean age was also strongly associated (β: 0.01 per additional year, p<0.001) and the proportion of women involved in the study (β: 0.75, p=0.008). In addition, setting (urban vs rural) was almost associated with heterogeneity (β: 0.59 compared with rural settings, p=0.06).

### Risk of bias

All reviewed studies had low risk of bias (online supplemental table 6). Nevertheless, sample size was not justified in three studies,^23 36 38^ and the outcome was self-reported in most of the studies except in five of them.^21 36 38 39 41^

## Discussion

### Summary of evidence

This systematic review provides a comprehensive analysis of the burden of multimorbidity in the LAC region. Accordingly, the pooled prevalence of multimorbidity was 43%. Nevertheless, there was a high heterogeneity among studies included, and results varied by the characteristics of the study as well as some of population characteristics. Thus, the pooled prevalence was lower in nationally representative compared with subnational samples, whereas estimates were higher when only studies with measured and self-reported chronic conditions were included. In addition, age seems to be an important predictor as the prevalence of multimorbidity among those aged 50 years and over was high compared with the pooled estimate of multimorbidity. Finally, the pooled prevalence of multimorbidity was higher among women compared with men, highlighting the link between sex and multimorbidity and among urban compared with those rural dwellers highlighting perhaps better access to diagnostic care in urban sites.

### Limitations of the review

There are some limitations in this review that should be highlighted. First, high heterogeneity is present in almost all the results. This finding may be attributable to age group inclusion criteria since a great proportion of studies enrolled individuals aged ≥50+ years and the pooled estimate of multimorbidity was high in this group. In addition, the proportion of women enrolled in each individual study may be relevant as pointed out by the meta-regression analyses. Second, the number of chronic conditions as well as the list of them used to define multimorbidity is very dissimilar. Defining specific clusters of multimorbidity is needed to guarantee comparability between studies, but this is not usually reported. Therefore, it is relevant to standardise the definition of multimorbidity and the conditions included in such definition to estimate which clusters of multimorbidity are more frequent and relevant for LAC region. In addition to that, before recent definition of multimorbidity, some reports used other different definitions (≥3 or ≥5 chronic conditions), which could affect pooled results. Fortunately, analysis was possible to include only those with ≥2 chronic conditions. Third, most of the studies included in the review were from Brazil, preventing inferability to the whole region, but also highlighting the need of population-based studies on multimorbidity in other countries of the region. Of note, the pooled prevalence of multimorbidity was higher in Brazil compared with other countries, perhaps because Brazilian researchers have addressed a common definition of multimorbidity, using a list of 12 conditions. Finally, a bias due to self-reporting can affect our results. Therefore, whether multimorbidity is defined by self-report or by more objective measurements may have an impact on prevalence estimates. Awareness of some chronic conditions are usually low and varying; for example, hypertension and diabetes awareness is around 64% and 78%, respectively, in urban areas of Latin America,^43^ but tend to be lower in rural settings.^44^ Thus, our results may be underestimating the real burden of multimorbidity in the LAC region.

### Public health relevance

Global trends suggest that multimorbidity is a public health challenge; thus, understanding the epidemiology of multimorbidity in LAC region may be relevant as this issue has received little attention from researchers, but especially, policymakers. Moreover, much of the response of the health system has been developed based on one specific condition or specific body system, instead of an integral approach whereby multiple conditions are addressed synergistically. Therefore, disperse and heterogeneous information have been available for this systematic review.

Our results highlight the need of implementing a surveillance system focused on multimorbidity. This can be done by including some specific conditions in routine health surveys (Demographic Health Surveys or similar) and other population-based research studies. In addition, these surveys may include the most common clusters of multimorbidity, and those with higher morbidity and mortality,^45^ however, such relevant clusters need to be appropriately defined.

Our results also imply that health systems need to be adapted to face the challenge of multimorbidity which increases healthcare use and costs related to primary and secondary prevention.^2^ This adaptation process includes the appropriate training of human resources as well as improving of health services infrastructure and care delivery. It is also needed to develop guidelines for multimorbidity care as that of National Institute for Health and Care Excellence that includes clinical assessment and adequate management,^46^ but also highlight related issues as polypharmacy and life expectancy. Therefore, a holistic approach may be needed to tackle this global health problem.

## Conclusions

Our systematic review shows that 4 of 10 participants have multimorbidity at the population level in LAC. There is, however, a marked variability, depending on participant’s age and the number of chronic conditions assessed, highlighting the need of better designed and standardised studies to inform the landscape of multimorbidity in LAC.

## Supplementary Material

Reviewer comments

Author's manuscript

## Data Availability

As this is a systematic review and meta-analysis, all data relevant to the study are included in the article or uploaded as supplemental information.

## References

[1] The Academy of Medical Sciences. Multimorbidity: a priority for health research. London, UK: Academy of Medical Sciences, 2018.

[2] Soley-Bori M, Ashworth M, Bisquera A, et al. Impact of multimorbidity on healthcare costs and utilisation: a systematic review of the UK literature. Br J Gen Pract 2021;71:e39–46. 10.3399/bjgp20X713897 33257463 PMC7716874

[3] Makovski TT, Schmitz S, Zeegers MP, et al. Multimorbidity and quality of life: systematic literature review and meta-analysis. Ageing Res Rev 2019;53:100903. 10.1016/j.arr.2019.04.005 31048032

[4] Barnett K, Mercer SW, Norbury M, et al. Epidemiology of multimorbidity and implications for health care, research, and medical education: a cross-sectional study. Lancet 2012;380:37–43. 10.1016/S0140-6736(12)60240-2 22579043

[5] Banerjee A, Hurst J, Fottrell E, et al. Multimorbidity: not just for the West. Glob Heart 2020;15:45. 10.5334/gh.835 32923339 PMC7413145

[6] Bilal U, Hessel P, Perez-Ferrer C, et al. Life expectancy and mortality in 363 cities of Latin America. Nat Med 2021;27:463-470. 10.1038/s41591-020-01214-4 33495602 PMC7960508

[7] Violan C, Foguet-Boreu Q, Flores-Mateo G, et al. Prevalence, determinants and patterns of multimorbidity in primary care: a systematic review of observational studies. PLoS One 2014;9:e102149. 10.1371/journal.pone.0102149 25048354 PMC4105594

[8] Pati S, Swain S, Hussain MA, et al. Prevalence and outcomes of multimorbidity in South Asia: a systematic review. BMJ Open 2015;5:e007235. 10.1136/bmjopen-2014-007235 PMC4606435 26446164

[9] Nguyen H, Manolova G, Daskalopoulou C, et al. Prevalence of multimorbidity in community settings: a systematic review and meta-analysis of observational studies. J Comorb 2019;9:2235042X1987093. 10.1177/2235042X19870934 PMC6710708 31489279

[10] Abebe F, Schneider M, Asrat B, et al. Multimorbidity of chronic non-communicable diseases in low- and middle-income countries: a scoping review. J Comorb 2020;10:2235042X2096191. 10.1177/2235042X20961919 PMC7573723 33117722

[11] Ouzzani M, Hammady H, Fedorowicz Z, et al. Rayyan-a web and mobile APP for systematic reviews. Syst Rev 2016;5:210. 10.1186/s13643-016-0384-4 27919275 PMC5139140

[12] Modesti PA, Reboldi G, Cappuccio FP, et al. Panethnic differences in blood pressure in Europe: a systematic review and meta-analysis. PLoS One 2016;11:e0147601. 10.1371/journal.pone.0147601 26808317 PMC4725677

[13] Nyaga VN, Arbyn M, Aerts M. Metaprop: a Stata command to perform meta-analysis of binomial data. Arch Public Health 2014;72:39. 10.1186/2049-3258-72-39 25810908 PMC4373114

[14] Freeman MF, Tukey JW. Transformations related to the angular and the square root. Ann Math Statist. 1950;21:607–11. 10.1214/aoms/1177729756

[15] Aguiar LB, Baccaro LF, de Souza Santos Machado V, et al. Disability and multimorbidity in women older than 50 years: a population-based household survey. Menopause 2015;22:660–6. 10.1097/GME.0000000000000355 25380276

[16] Amaral TLM, Amaral CdeA, Lima NSde, CdA A, NSd L, et al. Multimorbidity, depression and quality of life among elderly people assisted in the family health strategy in Senador Guiomard, ACRE, Brazil. Cien Saude Colet 2018;23:3077–84. 10.1590/1413-81232018239.22532016 30281744

[17] Andrade LH, Benseñor IM, Viana MC, et al. Clustering of psychiatric and somatic illnesses in the general population: multimorbidity and socioeconomic correlates. Braz J Med Biol Res 2010;43:483–91. 10.1590/s0100-879x2010007500024 20379689

[18] Araujo MEA, Silva MT, Galvao TF, et al. Prevalence and patterns of multimorbidity in Amazon region of Brazil and associated determinants: a cross-sectional study. BMJ Open 2018;8:e023398. 10.1136/bmjopen-2018-023398 PMC6231594 30391918

[19] Boing AF, Melo GR, Boing AC, et al. Associação entre depressão E doenças crônicas: um estudo populacional. Rev Saúde Pública 2012;46:617–23. 10.1590/S0034-89102012005000044 22735271

[20] CdS C, Flores TR, Wendt A. Inequalities in multimorbidity among elderly: a population-based study in a City in southern Brazil. Cad Saúde Pública 2018;34:e00040718. 10.1590/0102-311X00040718 30484558

[21] da Silva Almeida IL, dos Santos SR, Morbeck de Queiroz B. Lifestyle, morbidity and multimorbity in adult Quilombolas. ABCS health sci 2020;45:1325. 10.7322/abcshs.45.2020.1325

[22] de Souza Santos Machado V, Valadares ALR, da Costa-Paiva LS, et al. Multimorbidity and associated factors in Brazilian women aged 40 to 65 years: a population-based study. Menopause 2012;19:569–75. 10.1097/gme.0b013e3182455963 22415564

[23] Macinko J, Andrade FCD, Nunes BP, et al. Primary care and multimorbidity in six Latin American and Caribbean countries. Rev Panam Salud Publica 2019;43:e8–e. 10.26633/RPSP.2019.8 31093232 PMC6393736

[24] Montes MC, Bortolotto CC, Tomasi E, et al. Strength and multimorbidity among community-dwelling elderly from southern Brazil. Nutrition 2020;71:110636. 10.1016/j.nut.2019.110636 31877451

[25] Nunes BP, Batista SRR, FBd A. Multimorbidity: the Brazilian longitudinal study of aging (ELSI-Brazil). Rev Saude Publica 2018;2. 10.11606/S1518-8787.2018052000637 PMC6254906 30379288

[26] Nunes BP, Camargo-Figuera FA, Guttier M, et al. Multimorbidity in adults from a southern Brazilian City: occurrence and patterns. Int J Public Health 2016;61:1013–20. 10.1007/s00038-016-0819-7 27105883

[27] Nunes BP, Chiavegatto Filho ADP, Pati S, et al. Contextual and individual inequalities of multimorbidity in Brazilian adults: a cross-sectional national-based study. BMJ Open 2017;7:e015885. 10.1136/bmjopen-2017-015885 PMC5726142 28601836

[28] Nunes BP, Soares MU, Wachs LS, et al. Hospitalization in older adults: association with multimorbidity, primary health care and private health plan. Rev Saude Publica 2017;51:43. 10.1590/S1518-8787.2017051006646 28492761 PMC5433790

[29] Nunes BP, Thumé E, Facchini LA. Multimorbidity in older adults: magnitude and challenges for the Brazilian health system. BMC Public Health 2015;15:1172. 10.1186/s12889-015-2505-8 26602756 PMC4658761

[30] Pereira BP, Bortolotto CC, Tomasi E, Padilha Pereira B, Cardozo Bortolotto C, et al. Food consumption and multimorbidity among non-institutionalized elderly people in Pelotas, 2014: a cross-sectional study. Epidemiol Serv Saude 2020;29:e2019050. 10.5123/S1679-49742020000300015 32578666

[31] Petarli GB, Cattafesta M, Sant'Anna MM, et al. Multimorbidity and complex multimorbidity in Brazilian rural workers. PLoS One 2019;14:e0225416. 10.1371/journal.pone.0225416 31743369 PMC6863555

[32] Tavares DMDS, Pelizaro PB, Pegorari MS, et al. Prevalence of self-reported morbidities and associated factors among community-dwelling elderly in Uberaba, Minas Gerais, Brazil. Cien Saude Colet 2019;24:3305–13. 10.1590/1413-81232018249.31912017 31508751

[33] Valadares ALR, Lui-Filho JF, Costa-Paiva L, et al. Middle-aged female sexual dysfunction and multimorbidity: a population-based study. Menopause 2016;23:304–10. 10.1097/GME.0000000000000533 26506501

[34] Wang Y-P, Nunes BP, Coêlho BM, et al. Multilevel analysis of the patterns of Physical-Mental multimorbidity in general population of São Paulo metropolitan area, Brazil. Sci Rep 2019;9:2390. 10.1038/s41598-019-39326-8 30787376 PMC6382818

[35] Agrawal S, Agrawal PK. Association between body mass index and prevalence of multimorbidity in Low-and middle-income countries: a cross-sectional study. Int J Med Public Health 2016;6:73–83. 10.5530/ijmedph.2016.2.5 28894693 PMC5591643

[36] Bao J, Chua K-C, Prina M, et al. Multimorbidity and care dependence in older adults: a longitudinal analysis of findings from the 10/66 study. BMC Public Health 2019;19:585. 10.1186/s12889-019-6961-4 31096943 PMC6524243

[37] Bustos-Vázquez E, Fernández-Niño JA, Astudillo-Garcia CI. Self-Rated health, multimorbidity and depression in Mexican older adults: proposal and evaluation of a simple conceptual model. Biomedica 2017;37:92–103. 10.7705/biomedica.v37i3.3070 28527271

[38] Miranda JJ, Bernabe-Ortiz A, Gilman RH, et al. Multimorbidity at sea level and high-altitude urban and rural settings: the CRONICAS cohort study. J Comorb 2019;9:2235042X1987529–10. 10.1177/2235042X19875297 PMC8240099 34249770

[39] Taype-Rondan A, Abbs ES, Lazo-Porras M, et al. Association between chronic conditions and health-related quality of life: differences by level of urbanization in Peru. Qual Life Res 2017;26:3439–47. 10.1007/s11136-017-1649-7 28712003 PMC5681970

[40] Camargo-Casas S, Suarez-Monsalve S, Zepeda MUP, et al. [Multimorbidity, depressive symptoms, and self-reported health in older adults: a secondary analysis of the sabe bogota study]. Rev Invest Clin 2018;70:192–7. 10.24875/RIC.18002478 30067723

[41] Olivares DEV, Chambi FRV, Chañi EMM, et al. Risk factors for chronic diseases and multimorbidity in a primary care context of central Argentina: a web-based interactive and cross-sectional study. Int J Environ Res Public Health 2017;14. 10.3390/ijerph14030251. [Epub ahead of print: 02 03 2017]. PMC5369087 28257087

[42] Cavalcanti G, Doring M, Portella MR, et al. Multimorbidity associated with polypharmacy and negative self-perception of health. Rev bras geriatr gerontol 2017;20:634–42. 10.1590/1981-22562017020.170059

[43] Silva H, Hernandez-Hernandez R, Vinueza R, et al. Cardiovascular risk awareness, treatment, and control in urban Latin America. Am J Ther 2010;17:159–66. 10.1097/MJT.0b013e3181a84ec5 19535966

[44] Lerner AG, Bernabe-Ortiz A, Gilman RH, et al. The "rule of halves" does not apply in Peru: awareness, treatment, and control of hypertension and diabetes in rural, urban, and rural-to-urban migrants. Crit Pathw Cardiol 2013;12:53–8. 10.1097/HPC.0b013e318285ef60 23680809 PMC4656025

[45] Whitty CJM, Watt FM. Map clusters of diseases to tackle multimorbidity. Nature 2020;579:494–6. 10.1038/d41586-020-00837-4 32210388

[46] National Institute for Health and Care Excellence (NICE). Multimorbidity: clinical assessment and management: NICE guideline [NG56]. NICE, 2016. Available: https://www.nice.org.uk/guidance/ng56 [Accessed 30 Jan 2021].

