# Supplementary material for: Multimorbidity in Latin America and the Caribbean: a systematic review and meta-analysis
Source: BMJ Open. 2021 Jul 23;11(7):e050409. doi: 10.1136/bmjopen-2021-050409 (PMC8311299; doi:10.1136/bmjopen-2021-050409)
Supplement: Supplementary data [file bmjopen-2021-050409supp001.pdf]

SUPPLEMENTAL MATERIAL

E-Figure 1: Forest plot with pooled estimates of multimorbidity among males

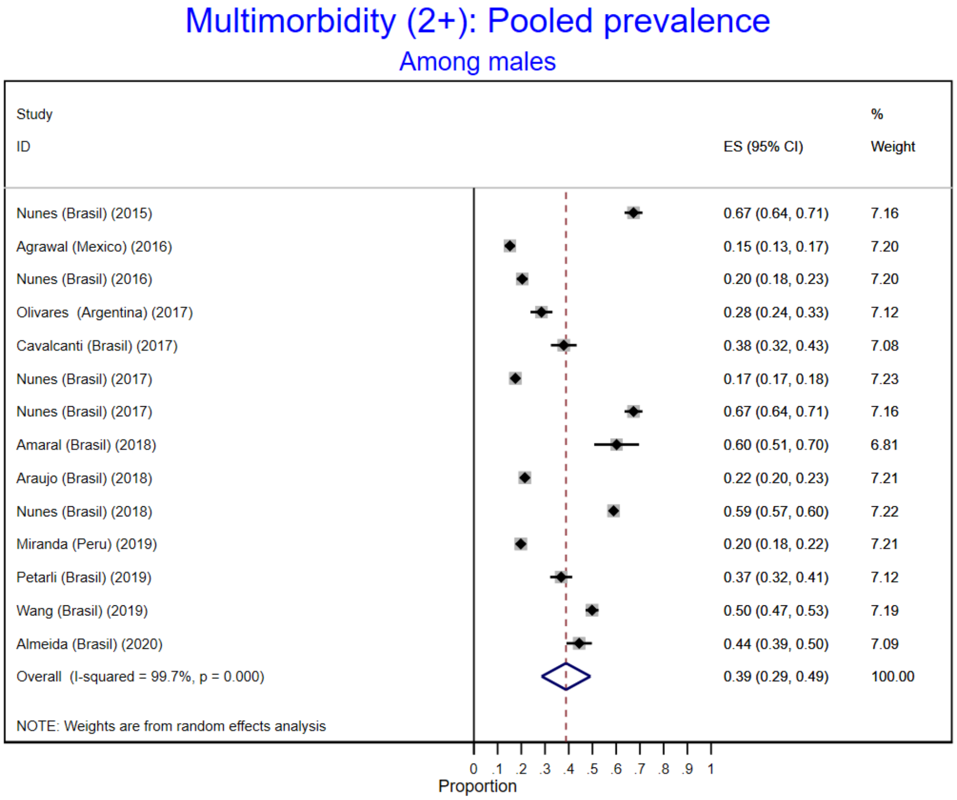

E-Figure 2: Forest plot with pooled estimates of multimorbidity among females

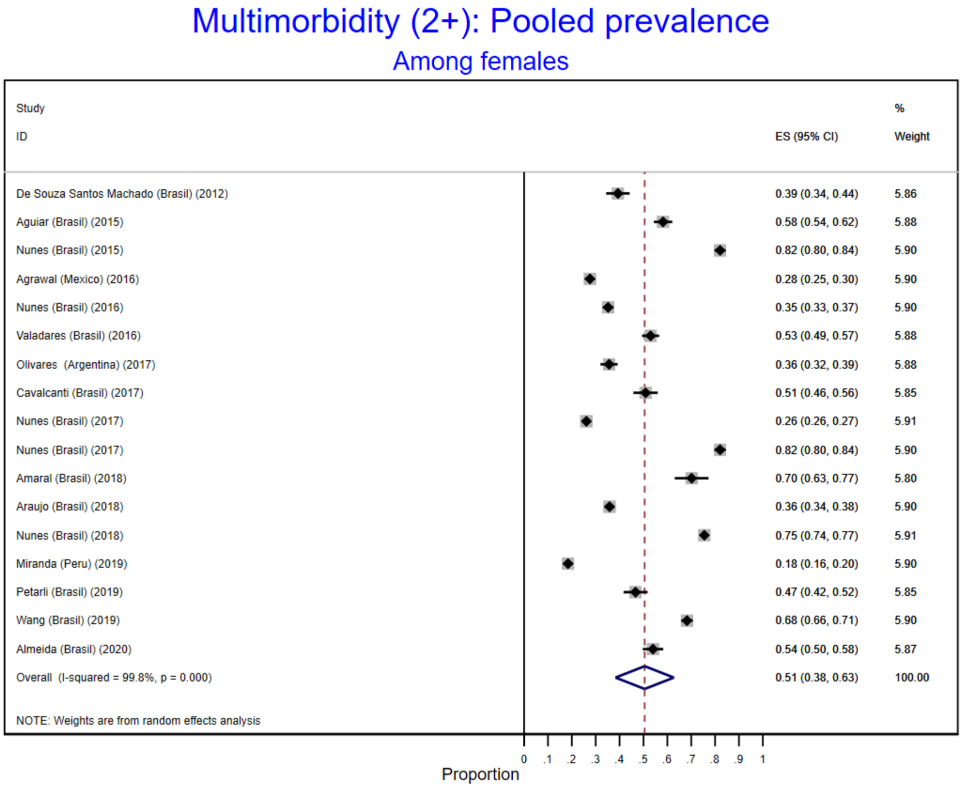

E-Figure 3: Forest plot with pooled estimates of multimorbidity among urban dwellers

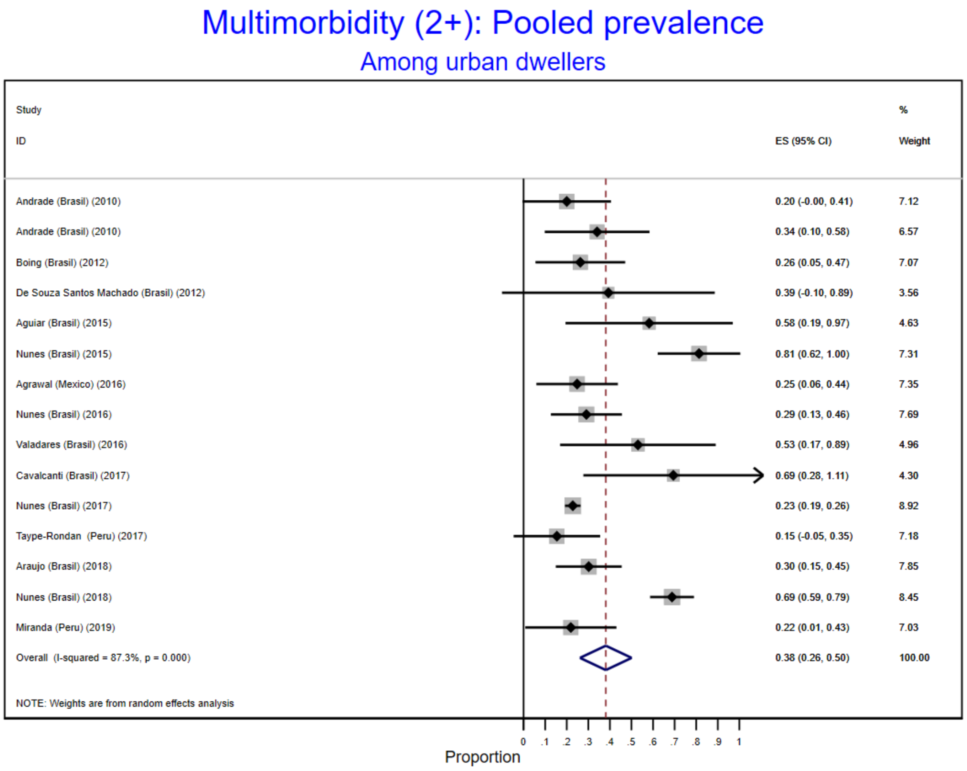

E-Figure 4: Forest plot with pooled estimates of multimorbidity among rural dwellers

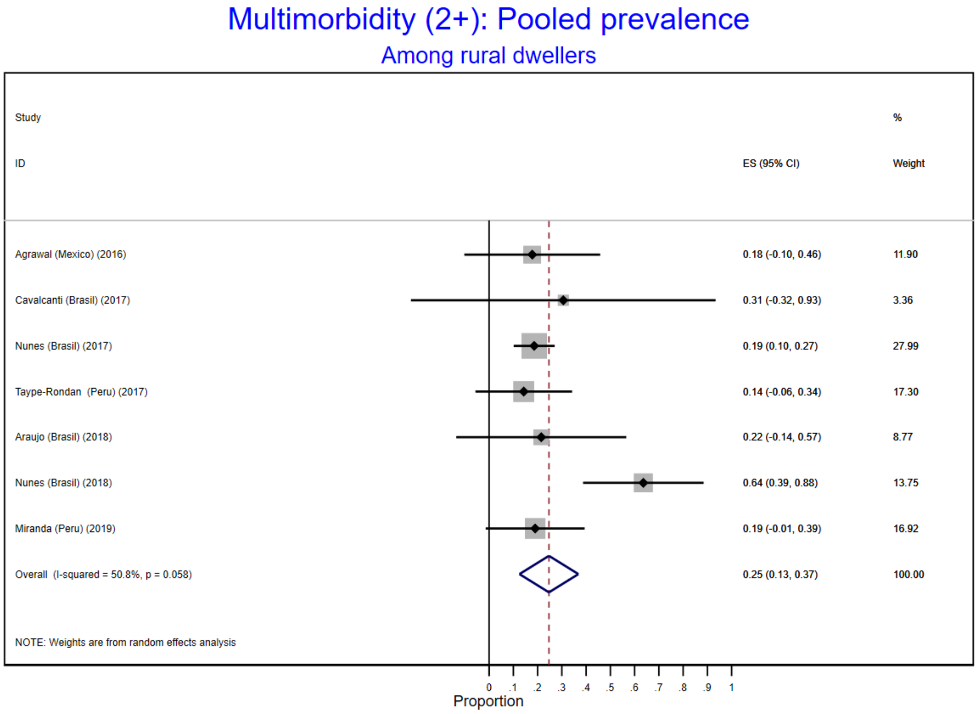

E-Table 1: Search terms and strategy using OVID (Embase, Medline and Global Health)

1	multimorbidit*.mp
2	multi-morbidit*.mp
3	(multi adj morbidit*).mp
4	multiple morbidit*.mp
5	(co adj morbidit*).mp
6	comorbidit*.mp
7	“multiple chronic conditions”.mp
8	“multiple chronic illnesses”.mp
9	“multiple chronic diseases”.mp
10	(poly adj morbidit*).mp
11	polymorbidit*.mp
12	poly-morbidit*.mp
13	1 or 2 or 3 or 4 or 5 or 6 or 7 or 8 or 9 or 10 or 11 or 12
14	(("Antigua and Barbuda") or ("Argentina") or ("Bahamas") or ("Barbados") or ("Belize") or ("Bolivia") or ("Brazil") or ("United States Virgin Islands") or ("British Virgin Islands") or ("Chile") or ("Colombia") or ("Costa Rica") or ("Cuba") or ("Dominica") or ("Dominican Republic") or ("Ecuador") or ("El Salvador") or ("Grenada") or ("Guatemala") or ("Guyana") or ("Haiti") or ("Honduras") or ("Jamaica") or ("Mexico") or ("Nicaragua") or ("Panama") or ("Paraguay") or ("Peru") or ("Puerto Rico") or ("Saint Kitts and Nevis") or ("Saint Lucia") or ("Saint Vincent and the Grenadines") or ("Suriname") or ("Trinidad and Tobago") or ("West Indies") or ("Uruguay") or ("Venezuela") or ("Latin America") or latin amer$ or ("South America") or south amer$ or ("Central America") or central amer$ or ("Caribbean Region"))
15	13 and 14
16	exp animals/ not humans.sh.
17	15 not 16
18	remove duplicates from 17

E-Table 2: Search terms and strategy using Scopus

(TITLE-ABS-KEY(multimorbidity$) OR TITLE-ABS-KEY(multi-morbidit$) OR TITLE-ABS-KEY(multi-morbidit$) OR TITLE-ABS-KEY(multi Pre/1 morbidit$) OR TITLE-ABS-KEY(multiple morbidit$) OR TITLE-ABS-KEY(co Pre/1 morbidit$) OR TITLE-ABS-KEY(comorbidit$) OR TITLE-ABS-KEY(multiple chronic conditions) OR TITLE-ABS-KEY(multiple chronic illnesses) OR TITLE-ABS-KEY(multiple chronic diseases) OR TITLE-ABS-KEY(poly Pre/1 morbidit$) OR TITLE-ABS-KEY(polymorbidit$) OR TITLE-ABS-KEY(poly-morbidit$)) AND (TITLE-ABS-KEY("Antigua and Barbuda") or TITLE-ABS-KEY("Argentina") or TITLE-ABS-KEY("Bahamas") or TITLE-ABS-KEY("Barbados") or TITLE-ABS-KEY("Belize") or TITLE-ABS-KEY("Bolivia") or TITLE-ABS-KEY("Brazil") or TITLE-ABS-KEY("United States Virgin Islands") or TITLE-ABS-KEY("British Virgin Islands") or TITLE-ABS-KEY("Chile") or TITLE-ABS-KEY("Colombia") or TITLE-ABS-KEY("Costa Rica") or TITLE-ABS-KEY("Cuba") or TITLE-ABS-KEY("Dominica") or TITLE-ABS-KEY("Dominican Republic") or TITLE-ABS-KEY("Ecuador") or TITLE-ABS-KEY("El Salvador") or TITLE-ABS-KEY("Grenada") or TITLE-ABS-KEY("Guatemala") or TITLE-ABS-KEY("Guyana") or TITLE-ABS-KEY("Haiti") or TITLE-ABS-KEY("Honduras") or TITLE-ABS-KEY("Jamaica") or TITLE-ABS-KEY("Mexico") or TITLE-ABS-KEY("Nicaragua") or TITLE-ABS-KEY("Panama") or TITLE-ABS-KEY("Paraguay") or TITLE-ABS-KEY("Peru") or TITLE-ABS-KEY("Puerto Rico") or TITLE-ABS-KEY("Saint Kitts and Nevis") or TITLE-ABS-KEY("Saint Lucia") or TITLE-ABS-KEY("Saint Vincent and the Grenadines") or TITLE-ABS-KEY("Suriname") or TITLE-ABS-KEY("Trinidad and Tobago") or TITLE-ABS-KEY("West Indies") or TITLE-ABS-KEY("Uruguay") or TITLE-ABS-KEY("Venezuela") or TITLE-ABS-KEY("Latin America") or TITLE-ABS-KEY(latin amer$) or TITLE-ABS-KEY("South America") or TITLE-ABS-KEY(south amer$) or TITLE-ABS-KEY("Central America") or TITLE-ABS-KEY(central amer$) or TITLE-ABS-KEY("Caribbean Region")) AND NOT DBCOLL(medl)

E-Table 3: Search terms and strategy using LILACS

((multi-morbilidad) OR (multimorbilidad) OR (co-morbilidad) OR (comorbilidad) OR (multiple morbilidad) OR (multiples condiciones cronicas) OR (multiples enfermedades cronicas) OR (multiples patologias cronicas) OR (poli-morbilidad) OR (polimorbilidad)) AND (("Antigua y Barbuda") OR ("Argentina") OR ("Aruba") OR ("Bahamas") OR ("Barbados") OR ("Belice") OR ("Bolivia") OR ("Brasil") OR ("Islas Vírgenes de los Estados Unidos") OR ("Islas Vírgenes Británicas") OR ("Islas Caimán") OR ("Chile") OR ("Colombia") OR ("Costa Rica") OR ("Cuba") OR ("Curazao") OR ("Dominica") OR ("Republica Dominicana") OR ("Ecuador") OR ("El Salvador") OR ("Granada") OR ("Guatemala") OR ("Guyana") OR ("Haití") OR ("Honduras") OR ("Jamaica") OR ("México") OR ("Nicaragua") OR ("Panamá") OR ("Paraguay") OR ("Perú") OR ("Puerto Rico") OR ("San Cristóbal y Nieves ") OR ("Santa Lucía") OR ("San Vicente y las Granadinas ") OR ("Surinam") OR ("Trinidad y Tobago") OR ("Turcas y Caicos ") OR ("Uruguay") OR ("Venezuela") OR ("América Latina") OR ("Latinoamérica") OR ("América del Sur") OR ("Sudamérica") OR ("Suramérica") OR ("América Central") OR ("Centroamérica") OR ("América del Centro") OR ("Caribe"))

E-Table 4: PRISMA Checklist

Section/topic	#	Checklist item	Reported on page #
TITLE			
Title	1	Identify the report as a systematic review, meta-analysis, or both.	01
ABSTRACT			
Structured summary	2	Provide a structured summary including, as applicable: background; objectives; data sources; study eligibility criteria, participants, and interventions; study appraisal and synthesis methods; results; limitations; conclusions and implications of key findings; systematic review registration number.	02
INTRODUCTION			
Rationale	3	Describe the rationale for the review in the context of what is already known.	03
Objectives	4	Provide an explicit statement of questions being addressed with reference to participants, interventions, comparisons, outcomes, and study design (PICOS).	03
METHODS			
Protocol and registration	5	Indicate if a review protocol exists, if and where it can be accessed (e.g., Web address), and, if available, provide registration information including registration number.	03-04
Eligibility criteria	6	Specify study characteristics (e.g., PICOS, length of follow-up) and report characteristics (e.g., years considered, language, publication status) used as criteria for eligibility, giving rationale.	04
Information sources	7	Describe all information sources (e.g., databases with dates of coverage, contact with study authors to identify additional studies) in the search and date last searched.	04
Search	8	Present full electronic search strategy for at least one database, including any limits used, such that it could be repeated.	Suppl material e-Tables 1-3
Study selection	9	State the process for selecting studies (i.e., screening, eligibility, included in systematic review, and, if applicable, included in the meta-analysis).	04
Data collection process	10	Describe method of data extraction from reports (e.g., piloted forms, independently, in duplicate) and any processes for obtaining and confirming data from investigators.	04

Data items	11	List and define all variables for which data were sought (e.g., PICOS, funding sources) and any assumptions and simplifications made.	04
Risk of bias in individual studies	12	Describe methods used for assessing risk of bias of individual studies (including specification of whether this was done at the study or outcome level), and how this information is to be used in any data synthesis.	04-05
Summary measures	13	State the principal summary measures (e.g., risk ratio, difference in means).	05
Synthesis of results	14	Describe the methods of handling data and combining results of studies, if done, including measures of consistency (e.g., I^2) for each meta-analysis.	05
Risk of bias across studies	15	Specify any assessment of risk of bias that may affect the cumulative evidence (e.g., publication bias, selective reporting within studies).	NA
Additional analyses	16	Describe methods of additional analyses (e.g., sensitivity or subgroup analyses, meta-regression), if done, indicating which were pre-specified.	05
RESULTS			
Study selection	17	Give numbers of studies screened, assessed for eligibility, and included in the review, with reasons for exclusions at each stage, ideally with a flow diagram.	05 and Figure 1
Study characteristics	18	For each study, present characteristics for which data were extracted (e.g., study size, PICOS, follow-up period) and provide the citations.	05 and Table 1
Risk of bias within studies	19	Present data on risk of bias of each study and, if available, any outcome level assessment (see item 12).	Suppl Material eTable 5
Results of individual studies	20	For all outcomes considered (benefits or harms), present, for each study: (a) simple summary data for each intervention group (b) effect estimates and confidence intervals, ideally with a forest plot.	06 and Suppl Material e-Table 6
Synthesis of results	21	Present results of each meta-analysis done, including confidence intervals and measures of consistency.	06 and Tables
Risk of bias across studies	22	Present results of any assessment of risk of bias across studies (see Item 15).	NA
Additional analysis	23	Give results of additional analyses, if done (e.g., sensitivity or subgroup analyses, meta-regression [see Item 16]).	06 and Suppl Material e-Figure 1-4

DISCUSSION			
Summary of evidence	24	Summarize the main findings including the strength of evidence for each main outcome; consider their relevance to key groups (e.g., healthcare providers, users, and policy makers).	06
Limitations	25	Discuss limitations at study and outcome level (e.g., risk of bias), and at review-level (e.g., incomplete retrieval of identified research, reporting bias).	07
Conclusions	26	Provide a general interpretation of the results in the context of other evidence, and implications for future research.	07-08
FUNDING			
Funding	27	Describe sources of funding for the systematic review and other support (e.g., supply of data); role of funders for the systematic review.	01

E-Table 5: Coverage and conditions included in the definition of multimorbidity

First author	Study coverage	Number of conditions	Conditions included in the definition of multimorbidity
Andrade [1]	Sub-national	8	Back pain, hypertension, asthma, headache, heart problems, diabetes, stroke, and cancer
Andrade [1]	Sub-national	15	Cognitive impairment, non-affective psychosis, panic attacks, generalized anxiety, any phobias, obsessive-compulsive disorders, depression, dysthymia, bipolar disorder, bulimia, alcohol use disorder, substance use disorder, nicotine dependence, somatoform disorder, and dissociative disorder
Boing [2]	Sub-national	11	Problems in the spine or on the back, arthritis or rheumatism, cancer, diabetes, bronchitis or asthma, hypertension, heart disease, chronic renal failure, tuberculosis, tendinitis or tenosynovitis, and cirrhosis
De Souza Santos Machado [3]	Sub-national	5	Depression, hypertension, diabetes mellitus, urinary incontinence, and insomnia
Aguiar [4]	Sub-national	10	Hypertension, myocardial infarction, stroke, diabetes, pulmonary emphysema, bronchitis/asthma, arthrosis, osteoporosis, cataract, and urinary incontinence
Nunes [5]	Sub-national	17	High blood pressure, diabetes, lung problem, heart problem, stroke, rheumatism, arthritis or arthrosis, disease in spinal column (any problem reported), cancer, kidney problem, cognitive impairment, depression, urinary incontinence, amputation in any part of the body, eyesight problem, hearing problem, problem chewing food, and falls
Agrawal [6]	National	9	Angina pectoris, arthritis, asthma, chronic lung disease, diabetes, hypertension, stroke, depression, and low visual acuity
Nunes [7]	Sub-national	11	High blood pressure, depression, heart disease, hypercholesterolemia, diabetes, bronchitis, asthma/wheezy bronchitis, hypothyroidism, osteoporosis, joint problems, and emphysema
Valadares [8]	Sub-national	11	Hypertension, diabetes mellitus, dyslipidemia, myocardial infarction, stroke, deep venous thrombosis or pulmonary embolus, asthma or bronchitis, chronic rheumatic diseases, depression, anxiety, and cancer
Bustos-Vazquez [9]	National	7	Cancer, systemic arterial hypertension, diabetes, depression, cerebral infarction, hypercholesterolemia, and cardiovascular diseases
Cavalcanti [10]	Sub-national	8	Systemic arterial hypertension, diabetes mellitus, rheumatism, pulmonary problems (asthma, bronchitis and pulmonary emphysema), stroke/cerebral ischemia, arthritis/osteoporosis, heart problems (coronary disease, angina, congestive disease, others), and depression

Nunes [11]	National	22	High blood pressure, spinal column problem, hypercholesterolemia, depression, diabetes, arthritis/rheumatism, asthma/wheezy bronchitis, cancer, other heart disease, work-related muscle-skeletal disorders, stroke, heart attack, kidney problem, heart failure, bronchitis, angina, emphysema, other lung disease, bipolar disorder, other mental disease, schizophrenia, and obsessive-compulsive disorder
Nunes [12]	Sub-national	17	Systemic arterial hypertension, diabetes mellitus, heart disease, pulmonary problems, stroke, rheumatism/arthritis/osteoarthritis, spine problems, cancer, kidney failure, cognitive deficit, depression, visual problems, urinary incontinence, amputation, auditory problems, problem or difficulty chewing food, and falls
Olivares [13]	Sub-national	9	Hypertension, diabetes, high cholesterol, AMI or stroke, asthma, hypothyroidism, celiac disease, cancer and other chronic diseases (arthritis, osteoarthritis, osteoporosis, depression, chronic allergies, COPD; gastritis, migraine, irritable bowel syndrome, epilepsy, psychosis, multiple sclerosis, psoriasis, gout disease, herniated disc, inflammatory vasculitis)
Taype-Rondan [14]	Sub-national	6	Hypertension, chronic bronchitis, depressive mood, type 2 diabetes, heart disease, and stroke
Amaral [15]	Sub-national	8	Hypertension, diabetes, cardiovascular disorder (poor circulation, cerebrovascular accident (CVA or stroke) and heart problem), musculoskeletal disorder (spinal problem, rheumatism, arthritis/ arthrosis and osteoporosis), insomnia, cataracts, anemia and gastrointestinal/urinary disorders
Araujo [16]	Sub-national	12	Hypertension, diabetes, high cholesterol, heart disease (heart attack, angina, heart failure or other), stroke, asthma or asthmatic bronchitis, arthritis or rheumatism, depression, pulmonary disease (pulmonary emphysema, chronic bronchitis or chronic obstructive pulmonary disease), cancer, chronic kidney disease, and chronic spinal problems (chronic back or neck pain, low back pain, sciatic pain, vertebral or disc problems)
Camargo-Casas [17]	Sub-national	12	Hypertension, diabetes, cancer, chronic obstructive pulmonary disease, heart attack, heart failure, stroke, arthritis or osteoarthritis, osteoporosis, gastroesophageal reflux disease, gastritis, and ulcer
Costa [18]	Sub-national	29	High blood pressure, diabetes, heart problem, heart failure, asthma, bronchitis, emphysema, arthritis, Parkinson’s disease, kidney failure, hypercholesterolemia, seizure, stomach ulcer, osteoporosis, urinary incontinence, constipation, fecal incontinence, depression, glaucoma, deafness, difficulty swallowing, insomnia, fainting, rhinitis, difficulty speaking, stroke, obesity, mental disorders, and cancer
Nunes [19]	National	19	Systemic arterial hypertension, back problems, high cholesterol, cataract, arthritis or rheumatism, depression, diabetes, osteoporosis heart problem, glaucoma, emphysema

			chronic bronchitis or chronic obstructive pulmonary disease, cerebrovascular accident, cancer, asthma, chronic renal failure, diabetic retinopathy, macular degeneration, Parkinson's disease, and Alzheimer's disease
Bao [20]	National	15	Vision, hearing, joints, skin, gastrointestinal, stroke, chronic obstructive pulmonary disease, hypertension, diabetes, ischemic heart disease, heart failure, heart valve disease, depression, anxiety, and dementia
Macinko [21]	National	8	Arthritis, asthma, cancer, depression, diabetes, heart disease, high cholesterol, and hypertension
Miranda [22]	Sub-national	6	Alcohol disorder, asthma, chronic obstructive pulmonary disease, depression, diabetes, and hypertension
Petarli [23]	Sub-national	20	Heart arrhythmia, infarction, stroke, diabetes mellitus, herniated disk, arthrosis, repetitive strain injuries/work-related musculoskeletal disorders, renal disease, Parkinson's, Alzheimer's, hepatic cirrhosis, infertility, cancer, asthma, chronic bronchitis, and pulmonary emphysema, arterial hypertension, dyslipidemia, depression and thyroid disorders
Tavares [24]	Sub-national	25	Rheumatism, arthritis/arthrosis, osteoporosis, asthma or bronchitis, tuberculosis, embolism, systemic arterial hypertension, poor circulation (varicose veins), heart problems, diabetes mellitus, obesity, stroke, Parkinson's, urinary incontinence, fecal incontinence, intestinal constipation, sleeping problems, cataract, glaucoma, back problems, kidney problem, accident/trauma sequelae, malignant tumors, benign tumors, and vision problems
Wang [25]	Sub-national	16	Cardiovascular diseases (heart attack, heart disease, and stroke), hypertension, diabetes mellitus, arthritis, chronic musculoskeletal pain, headache or migraine, digestive, respiratory (seasonal allergies, asthma, obstructive pulmonary disease, and emphysema), cancer, neurological diseases (Parkinson disease, epilepsy, and multiple sclerosis), anxiety disorder, mood disorder, heavy drinking, impulse-control disorder, substance use disorder, and premenstrual dysphoria
Montes [26]	Sub-national	28	Systemic arterial hypertension, myocardial infarction, diabetes, heart failure, emphysema, asthma, bronchitis, arthritis, Parkinson's disease, renal failure, seizure, hypercholesterolemia, stomach ulcer, osteoporosis, urinary incontinence, constipation, fecal incontinence, depression, glaucoma, deafness, difficulty swallowing, insomnia, fainting, rhinitis, difficulty speaking, stroke, mental disorders and cancer
Padilha Pereira [27]	Sub-national	28	Arterial hypertension, diabetes, heart problems, heart failure, asthma, bronchitis, emphysema, arthritis, Parkinson's disease, kidney failure, hypercholesterolemia, convulsions, stomach ulcer, osteoporosis, urinary incontinence, constipation, fecal

			incontinence, depression, glaucoma, deafness, difficulty in swallowing, insomnia, fainting, rhinitis, difficulty in speaking, stroke, mental disorders, and cancer
Da Silva Almeida [28]	Sub-national	5	Hypertension, diabetes, dyslipidemias, obesity, and arthritis or rheumatism

E-Table 6: Risk of bias of included studies

First author	Representativeness	Sample size	Non-respondents	Exposure	Outcome	Statistical test
Andrade [1]	Low	Low	High	NA	High	NA
Boing [2]	Low	Low	Low	NA	High	NA
De Souza Santos Machado [3]	Low	Low	Low	NA	High	NA
Aguiar [4]	Low	Low	Low	NA	High	NA
Nunes [5]	Low	Low	Low	NA	High	NA
Agrawal [6]	Low	Low	High	NA	High	NA
Nunes [7]	Low	Low	Low	NA	High	NA
Valadares [8]	Low	Low	Low	NA	High	NA
Bustos-Vazquez [9]	Low	Low	Low	NA	High	NA
Cavalcanti [10]	Low	Low	Low	NA	High	NA
Nunes [11]	Low	Low	High	NA	High	NA
Nunes [12]	Low	Low	Low	NA	High	NA
Olivares [13]	Low	Low	Low	NA	Low	NA
Taype-Rondan [14]	Low	Low	Low	NA	Low	NA
Amaral [15]	Low	Low	High	NA	High	NA
Araujo [16]	Low	Low	Low	NA	High	NA
Camargo-Casas [17]	Low	Low	Low	NA	High	NA
Costa [18]	Low	Low	Low	NA	High	NA
Nunes [19]	Low	Low	Low	NA	High	NA
Bao [20]	Low	High	Low	NA	Low	NA
Macinko [21]	Low	High	High	NA	High	NA
Miranda [22]	Low	High	Low	NA	Low	NA
Petarli [23]	Low	Low	Low	NA	High	NA
Tavares [24]	Low	Low	Low	NA	High	NA
Wang [25]	Low	Low	Low	NA	High	NA
Montes [26]	Low	Low	Low	NA	High	NA
Padilha Pereira [27]	Low	Low	Low	NA	High	NA
Da Silva Almeida [28]	Low	Low	Low	NA	Low	NA

REFERENCES

1. Andrade LH, Benseñor IM, Viana MC, Andreoni S, Wang YP. Clustering of psychiatric and somatic illnesses in the general population: multimorbidity and socioeconomic correlates. *Braz j med biol res* 2010;43(5):483-91.
2. Boing AF, Melo GR, Boing AC, Moretti-Pires RO, Peres KG, Peres MA. Associação entre depressão e doenças crônicas: um estudo populacional. *Rev saúde pública* 2012;46(4):617-23.
3. de Souza Santos Machado V, Valadares ALR, da Costa-Paiva LS, Moraes SS, Pinto-Neto AM. Multimorbidity and associated factors in Brazilian women aged 40 to 65 years: a population-based study. *Menopause (New York, NY)* 2012;19(5):569-75.
4. Aguiar LB, Baccaro LF, de Souza Santos Machado V, Pinto-Neto AM, Costa-Paiva L. Disability and multimorbidity in women older than 50 years: a population-based household survey. *Menopause (New York, NY)* 2015;22(6):660-6.
5. Nunes BP, Thume E, Facchini LA. Multimorbidity in older adults: magnitude and challenges for the Brazilian health system. *BMC public health* 2015;15:1172.
6. Agrawal S, Agrawal PK. Association Between Body Mass index and Prevalence of Multimorbidity in Low-and Middle-income Countries: A Cross-Sectional Study. *International journal of medicine and public health* 2016;6(2):73-83.
7. Nunes BP, Camargo-Figuera FA, Guttier M, de Oliveira PD, Munhoz TN, Matijasevich A, et al. Multimorbidity in adults from a southern Brazilian city: occurrence and patterns. *International journal of public health* 2016;61(9):1013-20.
8. Valadares ALR, Lui-Filho JF, Costa-Paiva L, Pinto-Neto AM. Middle-aged female sexual dysfunction and multimorbidity: a population-based study. *Menopause (New York, NY)* 2016;23(3):304-10.
9. Bustos-Vazquez E, Fernandez-Nino JA, Astudillo-Garcia CI. Self-rated health, multimorbidity and depression in Mexican older adults: Proposal and evaluation of a simple conceptual model. *Biomedica : revista del Instituto Nacional de Salud* 2017;37(0):92-103.
10. Cavalcanti G, Doring M, Portella MR, Bortoluzzi EC, Mascarelo A, Dellani MP. Multimorbidity associated with polypharmacy and negative self-perception of health. *Rev bras geriatr gerontol (Online)* 2017;20(5):634-42.
11. Nunes BP, Chiavegatto Filho ADP, Pati S, Cruz Teixeira DS, Flores TR, Camargo-Figuera FA, et al. Contextual and individual inequalities of multimorbidity in Brazilian adults: a cross-sectional national-based study. *BMJ open* 2017;7(6):e015885.
12. Nunes BP, Soares MU, Wachs LS, Volz PM, Saes MdO, Duro SMS, et al. Hospitalization in older adults: association with multimorbidity, primary health care and private health plan. *Rev saúde pública* 2017;51:43-.
13. Olivares DEV, Chambi FRV, Chani EMM, Craig WJ, Pacheco SOS, Pacheco FJ. Risk Factors for Chronic Diseases and Multimorbidity in a Primary Care Context of Central Argentina: A Web-Based Interactive and Cross-Sectional Study. *International journal of environmental research and public health* 2017;14(3).
14. Taype-Rondan A, Abbs ES, Lazo-Porras M, Checkley W, Gilman RH, Smeeth L, et al. Association between chronic conditions and health-related quality of life: differences by level of urbanization in Peru. *Quality of life research : an international journal of quality of life aspects of treatment, care and rehabilitation* 2017;26(12):3439-47.

15. Amaral TLM, Amaral CdA, Lima NSd, Herculano PV, Prado PRd, Monteiro GTR. Multimorbidity, depression and quality of life among elderly people assisted in the Family Health Strategy in Senador Guiomard, Acre, Brazil. *Multimorbidade, depressao e qualidade de vida em idosos atendidos pela Estrategia de Saude da Familia em Senador Guiomard, Acre, Brasil* 2018;23(9):3077-84.
16. Araujo MEA, Silva MT, Galvao TF, Nunes BP, Pereira MG. Prevalence and patterns of multimorbidity in Amazon Region of Brazil and associated determinants: a cross-sectional study. *BMJ open* 2018;8(11):e023398.
17. Camargo-Casas S, Suarez-Monsalve S, Zepeda MUP, Garcia-Pena C, Cano-Gutierrez CA. [Multimorbidity, Depressive Symptoms, and Self-Reported Health in Older Adults: a Secondary Analysis of the Sabe Bogota Study]. *Revista de investigacion clinica; organo del Hospital de Enfermedades de la Nutricion* 2018;70(4):192-7.
18. Costa CdS, Flores TR, Wendt A, Neves RG, Tomasi E, Cesar JA, et al. Inequalities in multimorbidity among elderly: a population-based study in a city in Southern Brazil. *Cad Saúde Pública (Online)* 2018;34(11):e00040718-e.
19. Nunes BP, Batista SRR, Andrade FBd, Souza Junior PRBd, Lima-Costa MF, Facchini LA. Multimorbidity: The Brazilian Longitudinal Study of Aging (ELSI-Brazil). *Revista de saude publica* 2018;52Suppl 2(Suppl 2):10s.
20. Bao J, Chua K-C, Prina M, Prince M. Multimorbidity and care dependence in older adults: a longitudinal analysis of findings from the 10/66 study. *BMC public health* 2019;19(1):585.
21. Macinko J, Andrade FCD, Nunes BP, Guanais FC. Primary care and multimorbidity in six Latin American and Caribbean countries. *Rev panam salud pública* 2019;43:e8-e.
22. Miranda JJ, Bernabe-Ortiz A, Gilman RH, Smeeth L, Malaga G, Wise RA, et al. Multimorbidity at sea level and high-altitude urban and rural settings: The CRONICAS Cohort Study. *Journal of Comorbiddity* 2019;9:1-10.
23. Petarli GB, Cattafesta M, Sant'Anna MM, Bezerra OMdPA, Zandonade E, Salaroli LB. Multimorbidity and complex multimorbidity in Brazilian rural workers. *PLoS ONE* 2019;14(11):e0225416.
24. Tavares DMDS, Pelizaro PB, Pegorari MS, Paiva MMd, Marchiori GF. Prevalence of self-reported morbidities and associated factors among community-dwelling elderly in Uberaba, Minas Gerais, Brazil. *Prevalencia de morbidades autorreferidas e fatores associados entre idosos comunitarios de Uberaba, Minas Gerais, Brasil* 2019;24(9):3305-13.
25. Wang Y-P, Nunes BP, Coelho BM, Santana GL, do Nascimento CF, Viana MC, et al. Multilevel Analysis of the Patterns of Physical-Mental Multimorbidity in General Population of Sao Paulo Metropolitan Area, Brazil. *Scientific reports* 2019;9(1):2390.
26. Montes MC, Bortolotto CC, Tomasi E, Gonzalez MC, Barbosa-Silva TG, Domingues MR, et al. Strength and multimorbidity among community-dwelling elderly from southern Brazil. *Nutrition* 2020;71.
27. Padilha Pereira B, Cardozo Bortolotto C, Tomasi E, Gonzalez MC, Gomes AP, Goncalves H, et al. Food consumption and multimorbidity among non-institutionalized elderly people in Pelotas, 2014: a cross-sectional study. *Epidemiol serv saúde* 2020;29(3):e2019050.
28. da Silva Almeida IL, dos Santos SR, Morbeck de Queiroz B, de Freitas Mussi RF. Lifestyle, morbidity and multimorbidity in adult Quilombolas. *ABCS health sci* 2020;45:1325.
