# Supplementary material for: Multimorbidity in Latin America and the Caribbean: a systematic review and meta-analysis
Source: BMJ Open. 2021 Jul 23;11(7):e050409. doi: 10.1136/bmjopen-2021-050409 (PMC8311299; doi:10.1136/bmjopen-2021-050409)
Supplement: Author's manuscript [file bmjopen-2021-050409.draft_revisions.pdf]

BMJ Open is committed to open peer review. As part of this commitment we make the peer review history of every article we publish publicly available.

When an article is published we post the peer reviewers' comments and the authors' responses online. We also post the versions of the paper that were used during peer review. These are the versions that the peer review comments apply to.

The versions of the paper that follow are the versions that were submitted during the peer review process. They are not the versions of record or the final published versions. They should not be cited or distributed as the published version of this manuscript.

BMJ Open is an open access journal and the full, final, typeset and author-corrected version of record of the manuscript is available on our site with no access controls, subscription charges or pay-per-view fees (http://bmjopen.bmj.com).

If you have any questions on BMJ Open's open peer review process please email info.bmjopen@bmj.com

Multimorbidity in Latin America and the Caribbean: A systematic review and meta-analysis

Journal:	*BMJ Open*
Manuscript ID	bmjopen-2021-050409
Article Type:	Original research
Date Submitted by the Author:	20-Feb-2021

Complete List of Authors:	Huaquía-Díaz, Alessandra; Universidad Científica del Sur<br>Chalán-Dávila, Tarick; Universidad Científica del Sur<br>Carrillo-Larco, Rodrigo; Imperial College London, ; CRONICAS Center of Excellence in Chronic Diseases<br>Bernabe-Ortiz, Antonio; Universidad Peruana Cayetano Heredia, CRONICAS Centre of Excellence in Chronic Diseases; Universidad Cientifica del Sur

Keywords:	EPIDEMIOLOGY, PUBLIC HEALTH, Hypertension < CARDIOLOGY, DIABETES & ENDOCRINOLOGY

SCHOLARONE™ Manuscripts

I, the Submitting Author has the right to grant and does grant on behalf of all authors of the Work (as defined in the below author licence), an exclusive licence and/or a non-exclusive licence for contributions from authors who are: i) UK Crown employees; ii) where BMJ has agreed a CC-BY licence shall apply, and/or iii) in accordance with the terms applicable for US Federal Government officers or employees acting as part of their official duties; on a worldwide, perpetual, irrevocable, royalty-free basis to BMJ Publishing Group Ltd ("BMJ") its licensees and where the relevant Journal is co-owned by BMJ to the co-owners of the Journal, to publish the Work in this journal and any other BMJ products and to exploit all rights, as set out in our licence.

The Submitting Author accepts and understands that any supply made under these terms is made by BMJ to the Submitting Author unless you are acting as an employee on behalf of your employer or a postgraduate student of an affiliated institution which is paying any applicable article publishing charge ("APC") for Open Access articles. Where the Submitting Author wishes to make the Work available on an Open Access basis (and intends to pay the relevant APC), the terms of reuse of such Open Access shall be governed by a Creative Commons licence – details of these licences and which Creative Commons licence will apply to this Work are set out in our licence referred to above.

Other than as permitted in any relevant BMJ Author's Self Archiving Policies, I confirm this Work has not been accepted for publication elsewhere, is not being considered for publication elsewhere and does not duplicate material already published. I confirm all authors consent to publication of this Work and authorise the granting of this licence.

For peer review only

Multimorbidity in Latin America and the Caribbean: A systematic review and meta-analysis

Alessandra M. Huaquía-Diaz, BS[1]; Tarick S. Chalán-Dávila, BS[1]; Rodrigo M. Carrillo-Larco, MD[2,3,4]; Antonio Bernabé-Ortiz, MD, MPH, PhD[1,2,5]

1. Universidad Científica del Sur, Lima, Peru
2. CRONICAS Center of Excellence in Chronic Diseases, Universidad Peruana Cayetano Heredia, Lima, Peru
3. Department of Epidemiology and Biostatistics, School of Public Health, Imperial College London, London, UK
4. Universidad Católica Los Ángeles de Chimbote, Instituto de Investigación, Chimbote, Peru
5. School of Public Health and Administration, Universidad Peruana Cayetano Heredia, Lima, Peru

Corresponding author:
Antonio Bernabe-Ortiz, MD, MPH, PhD
Universidad Científica del Sur, Lima, Peru
Email: Antonio.Bernabe@upch.pe

Author's contribution

TSC-D, AMH-D and AB-O conceived the idea of the manuscript. TSC-D and AMH-D conducted the review with support of AB-O and RMC-L. RMC-L and AB-O conducted the statistical analysis. TSC-D and AMH-D drafted the first version of the manuscript, with relevant comments and suggestions of RMC-L and AB-O. All the authors approved the version submitted for publication.

Conflict of interest:

None

For peer review only

Funding:

Rodrigo M Carrillo-Larco is supported by a Wellcome Trust International Training Fellowship (214185/Z/18/Z).

For peer review only

ABSTRACT

Objective: To estimate the pooled prevalence of multimorbidity (≥2 non-communicable diseases in the same individual) among adults of the general population of LAC.

Design: Systematic review and meta-analysis

Data sources: Medline, Embase, Global Health, Scopus, and LILACS up to July 1st, 2020

Eligibility criteria for selecting studies: The outcome of interest was the prevalence of multimorbidity. Reports were selected whether they enrolled adult individuals (age ≥18 years) from the general population.

Data extraction and synthesis: Reviewers extracted relevant data and assessed risk of bias independently. A random-effects meta-analysis was conducted to report pooled prevalence estimates of multimorbidity; pooled estimates by pre-specified sub-groups (e.g., national studies) was also pursued.

Results: From 5,830 results, we selected 28 reports, mostly from Brazil and 16 were based on a nationally representative sample. From the 28 selected reports, 26 were further included in the meta-analysis revealing a pooled multimorbidity (≥2 chronic conditions) prevalence of 43% (95% CI: 35% - 51%; I^2: 99.9%). When only reports with a nationally representative sample were combined, the pooled prevalence was 37% (95% CI: 27%-47%; I^2: 99.9%). When the ascertainment of multimorbidity was based on self-reports alone, the pooled prevalence was 40% (95% CI: 31% - 48%; I^2: 99.9%); this raised to 52% (95% CI: 33% - 70%; I^2: 99.9%) for reports including self-reported and objective diagnosis.

Conclusions: Our results complement and advance those from global efforts by incorporating much more reports from LAC. We revealed a larger presence of multimorbidity in LAC than previously reported.

Key words: Multimorbidity, systematic review, prevalence, Latin American and the Caribbean

For peer review only

STRENGHTS AND LIMITATIONS OF THIS STUDY

- This systematic review and meta-analysis provide a comprehensive analysis of the burden of multimorbidity in the LAC region.
- High heterogeneity is present in almost all the studies and results, mainly due to different conditions included to define multimorbidity.
- Our results may underestimate the real burden of multimorbidity in LAC as bias due to self-reporting may be present.

For peer review only

INTRODUCTION

The Academy of Medical Sciences defines multimorbidity as "*the existence of two or more medical chronic conditions in a single individual*" [1]. Subjects with multimorbidity tend to increase healthcare utilization and costs of primary and secondary care services [2]; also, multimorbidity has a subsequent impact on quality of life [3].

In high-income countries, multimorbidity rates is heterogeneous but seems depend on the age. Thus, in a cross-sectional study using the dataset of medical practices in Scotland, the prevalence of multimorbidity was 23% using a list of 40 conditions, and was present mainly in older people [4]. Moreover, multimorbidity seems to be increasing in low- and middle-income countries, where data is yet scarce [5]. The increase of life expectancy in Latin American and the Caribbean (LAC) has been associated with greater incidence of noncommunicable conditions (NDC) [6], with the consequent emergence of multimorbidity.

Multimorbidity prevalence has been explored and summarized in some systematic reviews around the world [7, 8] and results ranged from 5% to 98%; nevertheless, their results were mainly informed by high-income countries. In a relatively recent systematic review [9], 31 low- and middle-income countries (LMIC) were included with a prevalence of multimorbidity of 30%, compared to an estimate of 38% in high-income countries. However, only nine studies from the LAC region were included, but pooled estimates by region were not elucidated, which prevent to better understand the role of multimorbidity in this region. A more recent systematic review has reported a pooled prevalence of multimorbidity in LMIC between 3% and 90%, with almost 80% of the studies being from Brazil, China, South Africa, India, Mexico, and Iran [10].

The existing lack of evidence may have important consequences for research, public health, and clinical management in LAC region. For example, the conditions used to define multimorbidity were not appropriately defined up to 2018; in addition, whether estimates depend upon sex or setting characteristics should be also studied. Moreover, the need of surveillance systems to assess multimorbidity may be elucidated as these estimates have not been estimated in LAC region. Thus, from the public health perspective may not be easy to take appropriate decisions or implement adequate strategies to tackle the problem of multimorbidity.

As a result, we aimed at providing robust evidence about multimorbidity prevalence estimates in LAC region through a systematic review and meta-analysis of population-based surveys. These

For peer review only

results evidence may help to guide interventions and policies so that they can focus on the most pressing frequent multimorbidity phenotypes in LAC.

METHODS

Protocol

This systematic review was registered in PROSPERO under the code CRD42020196177. We aimed to identify the population-based prevalence of multimorbidity in LAC, and to study whether this prevalence varies by multimorbidity definitions, sex, and urban-rural settings.

Eligibility criteria

Reports were selected whether they enrolled adult individuals (age ≥18 years) from the general population. We focused on LAC populations; therefore, we excluded studies with LAC individuals in countries outside the LAC region, and studies with only foreign subjects in LAC nations. Population-based studies were defined as those following a random sampling approach, and such sample was taken from the general population. On the contrary, studies addressing specific populations (e.g., pregnant women), those with individuals with specific conditions (e.g., people with hypertension), or subjects with specific risk factors (e.g., obese or alcohol disorders) were excluded.

The outcome of interest was the prevalence of multimorbidity, defined as the existence of ≥ 2 chronic conditions in the same person [1]. Different definitions of multimorbidity were considered in this review (e.g., ≥2, ≥3, ≥4, or ≥5 conditions) as the current definition (≥2 conditions) is relatively recent. In addition, the presence of chronic conditions could have been measured, self-reported or a combination of these approaches.

Information sources

The search was conducted on January 10, 2020, and then updated on July 1st, 2020. We used Ovid search engine, comprising Medline, Embase and Global Health databases; and in parallel, we also searched Scopus and LILACS. In all of these, searching was carried out without time or language restriction. The search strategy and terms used is detailed in Supplementary Material: e-Table 1-3.

Study selection

Results from each search engine were downloaded and saved in EndNote where duplicates were removed. After that, information was transferred to Rayyan, an open-access online tool for

For peer review only

systematic reviews [11]. Titles and abstracts were reviewed by two researchers in an independent way, and disagreements were solved by a third party. After this screening phase, selected reports were downloaded and independently studied in detail by two researchers, and similarly, discrepancies were solved by a third party. Finally, selected studies were examined again to check for data duplication, i.e., different reports that used the same data (e.g., multiple reports based on the same underlying data). In this case, the paper with more information or the one with the largest sample size was included in the review and meta-analysis.

Data collection

An extraction template form was built by the authors and tested with a random sample of selected studies. After starting data collection, the form was not further modified. This form included study characteristics: study design, country, if it was a nationally representative sample, sample size, year of data collection, age range, proportion of women, and if it was urban, rural or both. The extraction form also collated the definition of multimorbidity used, self-reported or a combination of self-reported and measured, the number and a list of chronic conditions studies, and prevalence of multimorbidity (overall, by sex, and by rural or urban settings).

Risk of bias of individual studies

Risk of bias of selected studies was evaluated using the Newcastle-Ottawa Quality Assessment Scale adapted for cross-sectional studies as in a previous report [12]. This tool is focused on selection process (representativeness, sample size, and non-respondents), and the assessment of the outcome (independent blind assessment, self-report or not description). The items of this scale were implemented in an Excel spreadsheet and assessed independently by two reviewers; discrepancies were solved by a third party.

Summary measures

Our systematic review followed the Preferred Reporting Items for Systematic Review and Meta-Analysis (PRISMA) toolkit (See Checklist in Supplemental Material: e-Table 4). We present a qualitative and quantitative summary. The qualitative summary described the characteristics of the study (as listed above), whereas the quantitative summary explored pooled prevalence estimates.

Statistical analyses were performed using Stata version 16 for Windows (StataCorp, College Station, TX, US). The "*metaprop*" command attains a pooled estimate as a weighted average, by fitting a logistic-normal random-effect model without covariates, but random intercepts [13]. After

For peer review only

that, the pooled estimate was calculated using the Freeman-Tukey Arcsine Transformation as suggested in literature [14].

Because the selected studies were different in nature, scope (e.g., national surveys versus community/subnational studies, or urban vs. rural settings) and sample size, we also conducted random-effects meta-analysis for comparing estimates in specific subgroups (e.g., national studies only): In addition, stratified meta-analysis (e.g., by sex, and by urban/rural settings) was pursued. Finally, meta-regression was also conducted as a high level of heterogeneity was expected. Meta-regression command in STATA investigates if between-study heterogeneity can be explained by one or more of the variables included in the review.

Patient and Public Involvement

Patients or the public were not involved in the design, or conduct, or reporting, or dissemination plans of our research.

Ethical considerations

The systematic review protocol was reviewed and approved by the Ethical Committee in Universidad Científica del Sur, Lima, Peru.

RESULTS

Selection process

The search strategy yielded 5,830 titles and abstracts after removing duplicates (See Figure 1); of these 66 were studied in detail and 28 reports met the inclusion criteria. For quantitative analyses, 26 reports were included (2 were excluded as the definition of multimorbidity was ≥5 chronic conditions), representing information for 12 LAC countries.

General characteristics of selected studies

As shown in Table 1, Brazil contributes with a t total of 20 data points [15-34]; Mexico with 4 data points [23, 35-37]; Peru with 3 data points [36, 38, 39]; Colombia with 2 data points [23, 40]; and Argentina [41], Cuba [36], Dominican Republic [36], El Salvador [23], Jamaica [23], Panama [23], Puerto Rico [36], and Venezuela [36] with 1 data point each. On the other hand, only 16 studies analyzed a nationally representative sample (Table 1).

Data from 37 country-level points were used to calculate pooled estimates using a definition of multimorbidity of ≥2 chronic conditions. Only one country (Brazil) used an additional definition of multimorbidity (i.e., ≥3 chronic conditions) in 8 different reports. Fourteen reports had

For peer review only

information to calculate estimates for urban settings [15, 17-19, 22, 25-27, 29, 33, 35, 38, 39, 42], whereas 7 had information from rural areas [18, 25, 27, 35, 38, 39, 42].

Finally, the number of conditions evaluated to define multimorbidity ranged from 5 to 29, with a mean of 12.3 (SD: 5.7) conditions (Supplemental Material: e-Table 5). Hypertension and type 2 diabetes were the only conditions included in all the definitions of multimorbidity.

Synthesis of results

The meta-analysis included 26 studies with information with a pooled estimate of multimorbidity defined as ≥2 chronic conditions of 43% (95% CI: 35% - 51%; I^2: 99.9%; See Figure 2); whereas the pooled estimate for multimorbidity using ≥3 chronic conditions was 40% (95% CI: 22% - 57%; I^2: 99.9%).

On the other hand, nationally representative samples had a pooled estimate of multimorbidity (≥2 chronic conditions) of 37% (95% CI: 27% - 47%; I^2: 99.9%), whilst this estimate was 48% (95% CI: 35% - 61%; I^2: 99.9%) for non-nationally representative samples. Similarly, when multimorbidity was assessed as self-reported, pooled prevalence was 40% (95% CI: 31% - 48%; I^2: 99.9%), but the prevalence was 52% (95% CI: 33% - 70%; I^2: 99.9%) for those which have objectively measured and self-reported chronic conditions.

In stratified analyses, pooled prevalence of multimorbidity was 38.9% (95% CI: 28.6% - 49.1%; I^2: 99.7%) for males and 50.5% (95% CI: 38.3% - 62.7%; I^2: 99.8%) for females. These estimates were 38.1% (95% CI: 26.1% - 50.1%; I^2: 87.3%) and 24.7% (95% CI: 12.5% - 36.8%; I^2: 50.8%) for urban and rural dwellers, respectively (See Figures in Supplementary Material: e-Figure 1-4).

In meta-regression analysis, predictors positively associated with heterogeneity were age, number of chronic conditions used for defining multimorbidity, and the proportion of women in the study.

Risk of bias

All reviewed studies had low risk of bias (Supplementary Material: e-Table 6). Nevertheless, sample size was not justified in three studies [23, 36, 38], and the outcome was self-reported in most of the studies except in 5 of them [21, 36, 38, 39, 41].

DISCUSSION

Summary of evidence

For peer review only

This systematic review provides a comprehensive analysis of the burden of multimorbidity in the LAC region. According to our results, the pooled prevalence of multimorbidity was 43%. Nevertheless, there is a high heterogeneity among studies included, and results may vary according to the characteristics of the study as well as some of population characteristics. Thus, the pooled prevalence was lower in nationally representative compared to sub-national samples, whereas estimates were higher when only studies with measured and self-reported chronic conditions were included. Finally, the pooled prevalence of multimorbidity was higher among females compared to males, highlighting the link between sex and multimorbidity, and among urban compared to those rural dwellers highlighting perhaps better access to diagnostic care in urban sites.

Limitations of the review

There are some limitations in this review that should be highlighted. First, high heterogeneity is present in almost all the results. This finding may be attributable to age group inclusion criteria since a great proportion of studies enrolled individuals aged ≥50+ years as well as the proportion of women enrolled in each individual study as pointed out by the meta-regression analysis. Second, the number of chronic conditions as well as the list of them to define multimorbidity is very dissimilar. Defining clusters of multimorbidity is needed to guarantee comparability between studies, but this is not usually reported. Thus, standardization of the definition and assessment of multimorbidity is relevant to better understand this phenomenon. In addition to that, before recent definition of multimorbidity, some reports used other different definitions (≥ 3 or ≥ 5 chronic conditions); which could affect pooled results. Fortunately, analysis was possible to include only those with ≥2 chronic conditions. Finally, a bias due to self-reporting can affect our results. Therefore, whether multimorbidity is defined by self-report or by more objective measurements may have an impact on prevalence estimates. Awareness of some chronic conditions are usually low and varying; for example, hypertension and diabetes awareness is around 64% and 78%, respectively in urban areas of Latin America [43], but tend to be lower in rural settings [44]. Thus, our results may be underestimating the real burden of multimorbidity in the LAC region.

Public Health Relevance

Global trends suggest that multimorbidity is a public health challenge; thus, understanding the epidemiology of multimorbidity in LAC region may be relevant as this issue has received little attention from researchers, but especially, policymakers. Moreover, much of the response of the health system has been developed based on one specific condition or specific body system, instead

For peer review only

of an integral approach whereby multiple conditions are addressed synergistically. Therefore, disperse and heterogeneous information have been available for this systematic review.

Our results highlight the need of implementing a surveillance system focused on multimorbidity. This can be done by including some specific conditions in routine health surveys (Demographic Health Surveys or similar) and other population-based research studies. In addition, these surveys may include the most common clusters of multimorbidity, and those with higher morbidity and mortality [45].

Our results also imply that health systems need to be adapted to face the challenge of multimorbidity which increases healthcare use and costs related to primary and secondary prevention [2]. This adaptation process includes the appropriate training of human resources as well as improving of health services infrastructure and care delivery. It is also needed to develop guidelines for multimorbidity care as that of NICE that includes clinical assessment and adequate management [46], but also highlight related issues as polypharmacy and life expectancy. Therefore, a holistic approach may be needed to tackle this global health problem.

Conclusions

Our systematic review shows that four of ten participants have multimorbidity at the population level in LAC. There is, however, a marked variability, depending on participant's age and the number of chronic conditions assessed, highlighting the need of better designed and standardized studies to inform the landscape of multimorbidity in LAC.

REFERENCES

1. The Academy of Medical Sciences. *Multimorbidity: a priority for health research*. London, UK: Academy of Medical Sciences; 2018.
2. Soley-Bori M, Ashworth M, Bisquera A, Dodhia H, Lynch R, Wang Y, et al. Impact of multimorbidity on healthcare costs and utilisation: a systematic review of the UK literature. *Br J Gen Pract* 2021;71(702):e39-e46.
3. Makovski TT, Schmitz S, Zeegers MP, Stranges S, van den Akker M. Multimorbidity and quality of life: Systematic literature review and meta-analysis. *Ageing Res Rev* 2019;53:100903.

For peer review only

4. Barnett K, Mercer SW, Norbury M, Watt G, Wyke S, Guthrie B. Epidemiology of multimorbidity and implications for health care, research, and medical education: a cross-sectional study. *Lancet* 2012;380(9836):37-43.
5. Banerjee A, Hurst J, Fottrell E, Miranda JJ. Multimorbidity: Not Just for the West. *Glob Heart* 2020;15(1):45.
6. Bilal U, Hessel P, Perez-Ferrer C, Michael YL, Alfaro T, Tenorio-Mucha J, et al. Life expectancy and mortality in 363 cities of Latin America. *Nat Med* 2021.
7. Violan C, Foguet-Boreu Q, Flores-Mateo G, Salisbury C, Blom J, Freitag M, et al. Prevalence, determinants and patterns of multimorbidity in primary care: a systematic review of observational studies. *PLoS ONE* 2014;9(7):e102149.
8. Pati S, Swain S, Hussain MA, van den Akker M, Metsemakers J, Knottnerus JA, et al. Prevalence and outcomes of multimorbidity in South Asia: a systematic review. *BMJ open* 2015;5(10):e007235.
9. Nguyen H, Manolova G, Daskalopoulou C, Vitoratou S, Prince M, Prina AM. Prevalence of multimorbidity in community settings: A systematic review and meta-analysis of observational studies. *J Comorb* 2019;9:2235042x19870934.
10. Abebe F, Schneider M, Asrat B, Ambaw F. Multimorbidity of chronic non-communicable diseases in low- and middle-income countries: A scoping review. *J Comorb* 2020;10:2235042x20961919.
11. Ouzzani M, Hammady H, Fedorowicz Z, Elmagarmid A. Rayyan-a web and mobile app for systematic reviews. *Syst Rev* 2016;5(1):210.
12. Modesti PA, Reboldi G, Cappuccio FP, Agyemang C, Remuzzi G, Rapi S, et al. Panethnic Differences in Blood Pressure in Europe: A Systematic Review and Meta-Analysis. *PLoS ONE* 2016;11(1):e0147601.
13. Nyaga VN, Arbyn M, Aerts M. Metaprop: a Stata command to perform meta-analysis of binomial data. *Arch Public Health* 2014;72(1):39.
14. Freeman MF, Tukey JW. Transformations related to the angular and the square root. *Ann Math Stat* 1950;21:607-11.
15. Aguiar LB, Baccaro LF, de Souza Santos Machado V, Pinto-Neto AM, Costa-Paiva L. Disability and multimorbidity in women older than 50 years: a population-based household survey. *Menopause (New York, NY)* 2015;22(6):660-6.

For peer review only

16. Amaral TLM, Amaral CdA, Lima NSd, Herculano PV, Prado PRd, Monteiro GTR. Multimorbidity, depression and quality of life among elderly people assisted in the Family Health Strategy in Senador Guiomard, Acre, Brazil. *Multimorbidade, depressao e qualidade de vida em idosos atendidos pela Estrategia de Saude da Familia em Senador Guiomard, Acre, Brasil* 2018;23(9):3077-84.
17. Andrade LH, Benseñor IM, Viana MC, Andreoni S, Wang YP. Clustering of psychiatric and somatic illnesses in the general population: multimorbidity and socioeconomic correlates. *Braz j med biol res* 2010;43(5):483-91.
18. Araujo MEA, Silva MT, Galvao TF, Nunes BP, Pereira MG. Prevalence and patterns of multimorbidity in Amazon Region of Brazil and associated determinants: a cross-sectional study. *BMJ open* 2018;8(11):e023398.
19. Boing AF, Melo GR, Boing AC, Moretti-Pires RO, Peres KG, Peres MA. Associação entre depressão e doenças crônicas: um estudo populacional. *Rev saúde pública* 2012;46(4):617-23.
20. Costa CdS, Flores TR, Wendt A, Neves RG, Tomasi E, Cesar JA, et al. Inequalities in multimorbidity among elderly: a population-based study in a city in Southern Brazil. *Cad Saúde Pública (Online)* 2018;34(11):e00040718-e.
21. da Silva Almeida IL, dos Santos SR, Morbeck de Queiroz B, de Freitas Mussi RF. Lifestyle, morbidity and multimorbidity in adult Quilombolas. *ABCS health sci* 2020;45:1325.
22. de Souza Santos Machado V, Valadares ALR, da Costa-Paiva LS, Moraes SS, Pinto-Neto AM. Multimorbidity and associated factors in Brazilian women aged 40 to 65 years: a population-based study. *Menopause (New York, NY)* 2012;19(5):569-75.
23. Macinko J, Andrade FCD, Nunes BP, Guanais FC. Primary care and multimorbidity in six Latin American and Caribbean countries. *Rev panam salud pública* 2019;43:e8-e.
24. Montes MC, Bortolotto CC, Tomasi E, Gonzalez MC, Barbosa-Silva TG, Domingues MR, et al. Strength and multimorbidity among community-dwelling elderly from southern Brazil. *Nutrition* 2020;71.
25. Nunes BP, Batista SRR, Andrade FBd, Souza Junior PRBd, Lima-Costa MF, Facchini LA. Multimorbidity: The Brazilian Longitudinal Study of Aging (ELSI-Brazil). *Revista de saude publica* 2018;52Suppl 2(Suppl 2):10s.

For peer review only

26. Nunes BP, Camargo-Figuera FA, Guttier M, de Oliveira PD, Munhoz TN, Matijasevich A, et al. Multimorbidity in adults from a southern Brazilian city: occurrence and patterns. *International journal of public health* 2016;61(9):1013-20.

27. Nunes BP, Chiavegatto Filho ADP, Pati S, Cruz Teixeira DS, Flores TR, Camargo-Figuera FA, et al. Contextual and individual inequalities of multimorbidity in Brazilian adults: a cross-sectional national-based study. *BMJ open* 2017;7(6):e015885.

28. Nunes BP, Soares MU, Wachs LS, Volz PM, Saes MdO, Duro SMS, et al. Hospitalization in older adults: association with multimorbidity, primary health care and private health plan. *Rev saúde pública* 2017;51:43-.

29. Nunes BP, Thume E, Facchini LA. Multimorbidity in older adults: magnitude and challenges for the Brazilian health system. *BMC public health* 2015;15:1172.

30. Padilha Pereira B, Cardozo Bortolotto C, Tomasi E, Gonzalez MC, Gomes AP, Goncalves H, et al. Food consumption and multimorbidity among non-institutionalized elderly people in Pelotas, 2014: a cross-sectional study. *Epidemiol serv saúde* 2020;29(3):e2019050.

31. Petarli GB, Cattafesta M, Sant'Anna MM, Bezerra OMdPA, Zandonade E, Salaroli LB. Multimorbidity and complex multimorbidity in Brazilian rural workers. *PLoS ONE* 2019;14(11):e0225416.

32. Tavares DMDS, Pelizaro PB, Pegorari MS, Paiva MMd, Marchiori GF. Prevalence of self-reported morbidities and associated factors among community-dwelling elderly in Uberaba, Minas Gerais, Brazil. *Prevalencia de morbidades autorreferidas e fatores associados entre idosos comunitarios de Uberaba, Minas Gerais, Brasil* 2019;24(9):3305-13.

33. Valadares ALR, Lui-Filho JF, Costa-Paiva L, Pinto-Neto AM. Middle-aged female sexual dysfunction and multimorbidity: a population-based study. *Menopause (New York, NY)* 2016;23(3):304-10.

34. Wang Y-P, Nunes BP, Coelho BM, Santana GL, do Nascimento CF, Viana MC, et al. Multilevel Analysis of the Patterns of Physical-Mental Multimorbidity in General Population of Sao Paulo Metropolitan Area, Brazil. *Scientific reports* 2019;9(1):2390.

35. Agrawal S, Agrawal PK. Association Between Body Mass index and Prevalence of Multimorbidity in Low-and Middle-income Countries: A Cross-Sectional Study. *International journal of medicine and public health* 2016;6(2):73-83.

For peer review only

36. Bao J, Chua K-C, Prina M, Prince M. Multimorbidity and care dependence in older adults: a longitudinal analysis of findings from the 10/66 study. *BMC public health* 2019;19(1):585.

37. Bustos-Vazquez E, Fernandez-Nino JA, Astudillo-Garcia CI. Self-rated health, multimorbidity and depression in Mexican older adults: Proposal and evaluation of a simple conceptual model. *Biomedica : revista del Instituto Nacional de Salud* 2017;37(0):92-103.

38. Miranda JJ, Bernabe-Ortiz A, Gilman RH, Smeeth L, Malaga G, Wise RA, et al. Multimorbidity at sea level and high-altitude urban and rural settings: The CRONICAS Cohort Study. *Journal of Comorbiddity* 2019;9:1-10.

39. Taype-Rondan A, Abbs ES, Lazo-Porras M, Checkley W, Gilman RH, Smeeth L, et al. Association between chronic conditions and health-related quality of life: differences by level of urbanization in Peru. *Quality of life research : an international journal of quality of life aspects of treatment, care and rehabilitation* 2017;26(12):3439-47.

40. Camargo-Casas S, Suarez-Monsalve S, Zepeda MUP, Garcia-Pena C, Cano-Gutierrez CA. [Multimorbidity, Depressive Symptoms, and Self-Reported Health in Older Adults: a Secondary Analysis of the Sabe Bogota Study]. *Revista de investigacion clinica; organo del Hospital de Enfermedades de la Nutricion* 2018;70(4):192-7.

41. Olivares DEV, Chambi FRV, Chani EMM, Craig WJ, Pacheco SOS, Pacheco FJ. Risk Factors for Chronic Diseases and Multimorbidity in a Primary Care Context of Central Argentina: A Web-Based Interactive and Cross-Sectional Study. *International journal of environmental research and public health* 2017;14(3).

42. Cavalcanti G, Doring M, Portella MR, Bortoluzzi EC, Mascarelo A, Dellani MP. Multimorbidity associated with polypharmacy and negative self-perception of health. *Rev bras geriatr gerontol (Online)* 2017;20(5):634-42.

43. Silva H, Hernandez-Hernandez R, Vinueza R, Velasco M, Boissonnet CP, Escobedo J, et al. Cardiovascular risk awareness, treatment, and control in urban Latin America. *Am J Ther* 2010;17(2):159-66.

44. Lerner AG, Bernabe-Ortiz A, Gilman RH, Smeeth L, Miranda JJ. The "rule of halves" does not apply in Peru: awareness, treatment, and control of hypertension and diabetes in rural, urban, and rural-to-urban migrants. *Crit Pathw Cardiol* 2013;12(2):53-8.

45. Whitty CJM, Watt FM. Map clusters of diseases to tackle multimorbidity. *Nature* 2020;579(7800):494-6.

For peer review only

46. National Institute for Health and Care Excellence (NICE). Multimorbidity: clinical assessment and management: NICE guideline [NG56]. NICE; 2016 [updated 2016; cited 2021 January 30]; Available from: https://www.nice.org.uk/guidance/ng56.

For peer review only

TABLES AND FIGURES

Figure 1: Flowchart of studies included in the systematic review

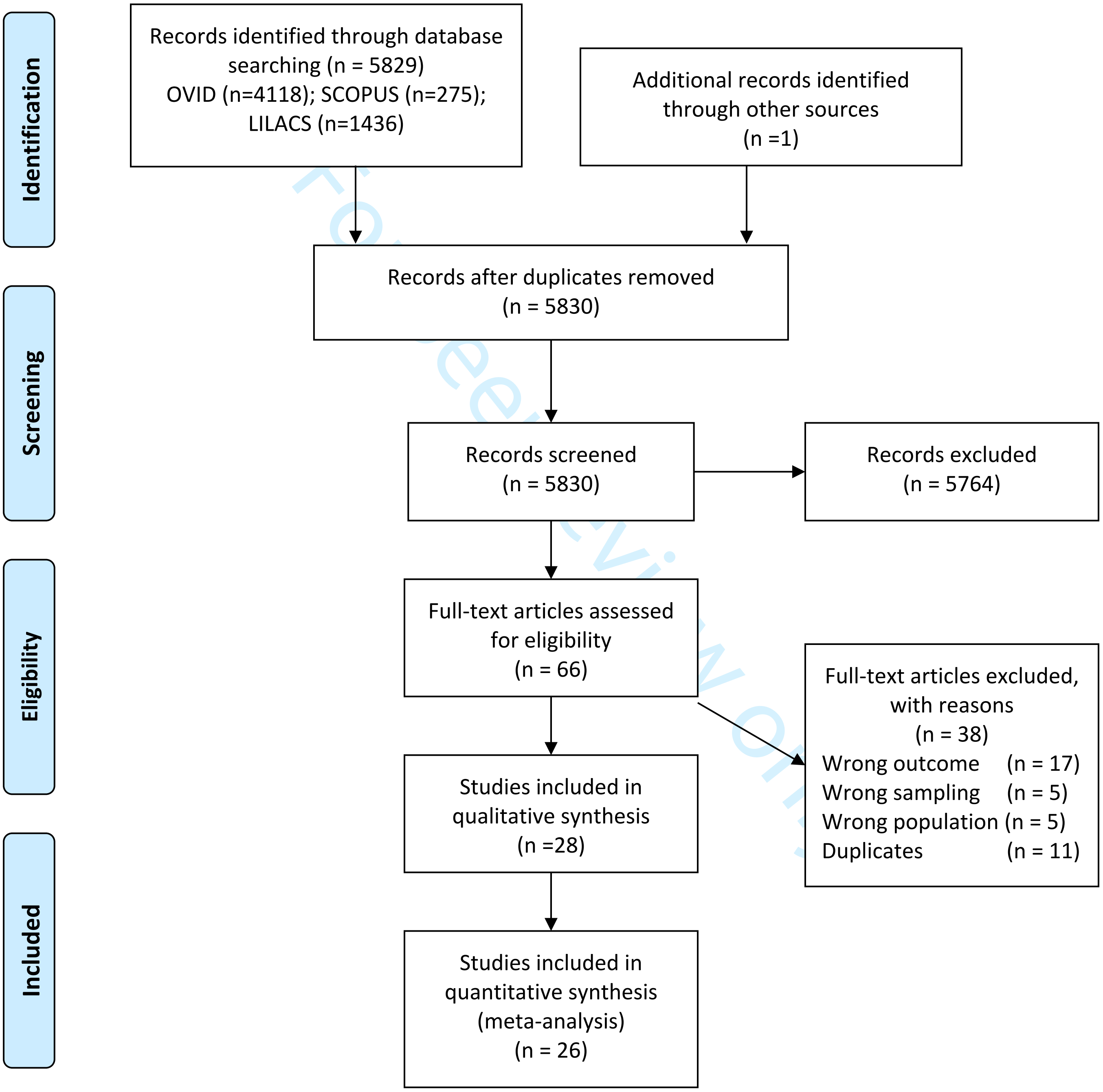

Figure 2: Forest plot of the pooled prevalence of multimorbidity defined as ≥2 chronic conditions

For peer review only

Table 1: Characteristics of the studies included in the systematic review

First author	Country	Study design	Data collected in	Sample size	Definition of multimorbidity	Age range	% women	% urban
Andrade [17]	Brazil	Cross-sectional	1994-1995	1,464	≥2 (self-reported)	18+	57.4%	100%
Boing [19]	Brazil	Cross-sectional	2009-2010	1,720	≥2 (self-reported)	20 - 59	55.6%	100%
De Souza Santos Machado [22]	Brazil	Cross-sectional	2005	377	≥2 (self-reported)	40 - 65	100%	100%
Aguiar [15]	Brazil	Cross-sectional	2011	622	≥2 (self-reported)	50+	100%	100%
Nunes [29]	Brazil	Cross-sectional	2008	1,593	≥2 and ≥3 (self-reported)	60+	62.8%	100%
Agrawal [35]	Mexico	Cross-sectional	2007-2010	2,725	≥2 (self-reported)	18+	61.8%	73.7%
Nunes [26]	Brazil	Cross-sectional	2012	2,927	≥2 and ≥3 (self-reported)	20+	58.9%	100%
Valadares [33]	Brazil	Cross-sectional	2012-2013	736	≥2 (self-reported)	45 - 60	100%	100%
Bustos-Vazquez [37]	Mexico	Cross-sectional	2012	7,967	≥2 (self-reported)	60+	53.4%	--
Cavalcanti [42]	Brazil	Cross-sectional	2010-2011	676	≥2 (self-reported)	60+	54.6%	69.4%
Nunes [27]	Brazil	Cross-sectional	2013	60,202	≥2 and ≥3 (self-reported)	18+	55.1%	86.5%
Nunes [28]	Brazil	Cross-sectional	2008	1,593	≥2 and ≥3 (self-reported)	60+	62.8%	--
Olivares [41]	Argentina	Cross-sectional	2014-2015	1,044	≥2 (self-reported)	18+	65%	--
Taype-Rondan [39]	Peru	Longitudinal	2013-2014	2,433	≥2	35+	51.3%	51.3%

For peer review only

					(self-reported and measured)			
Amaral [16]	Brazil	Cross-sectional	2010	264	≥2 (self-reported)	60+	61%	100%
Araujo [18]	Brazil	Cross-sectional	2015	4,001	≥2 and ≥3 (self-reported)	18+	52.7%	86.8%
Camargo-Casas [40]	Colombia	Cross-sectional	2012	2,000	≥2 (self-reported)	60+	63.4%	--
Costa [20]	Brazil	Cross-sectional	2014	1,451	≥2 and ≥3 (self-reported and measured)	60+	63%	--
Nunes [25]	Brazil	Longitudinal	2015-2016	9,412	≥2 and ≥3 (self-reported)	50+	54%	84.7%
Bao [36]	Cuba,<br>Dominican Republic,<br>Puerto Rico,<br>Venezuela,<br>Peru,<br>Mexico	Longitudinal	2003-2007	2,944<br>2,011<br>2,009<br>1,965<br>1,933<br>2,003	≥2 (self-reported and measured)	65+	64%<br>65%<br>67%<br>63%<br>60%<br>64%	--
Macinko [23]	Brazil,<br>Colombia,<br>El Salvador,<br>Jamaica,<br>Mexico,<br>Panama	Cross-sectional	2013-2014	1,486<br>1,485<br>1,460<br>1,480<br>1,492<br>1,475	≥2 (self-reported)	18+	51.1%<br>51.1%<br>52.8%<br>52.6%<br>51.4%<br>51.6%	--
Miranda [38]	Peru	Longitudinal	2010-2012	2,890	≥2 (self-reported and measured)	35+	51%	50.9%
Petarli [31]	Brazil	Cross-sectional	2016-2017	790	≥2 and ≥3 (self-reported)	18 - 59	47.7%	--
Tavares [32]	Brazil	Cross-sectional	2012	1,691	≥2 (self-reported)	60+	63.7%	100%
Wang [34]	Brazil	Cross-sectional	2005-2007	2,713	≥2 (self-reported)	18 - 64	52,4%	--
Montes [24]	Brazil	Cross-sectional	2014	1,336	≥5 (self-reported)	60+	63.1%	--

Padilha Pereira [30]	Brazil	Cross-sectional	2014	1,426	≥5 (self-reported)	60+	63%	--
Da Silva Almeida [21]	Brazil	Cross-sectional	2016	850	≥2 (self-reported and measured)	18+	61.2%	--

For peer review only

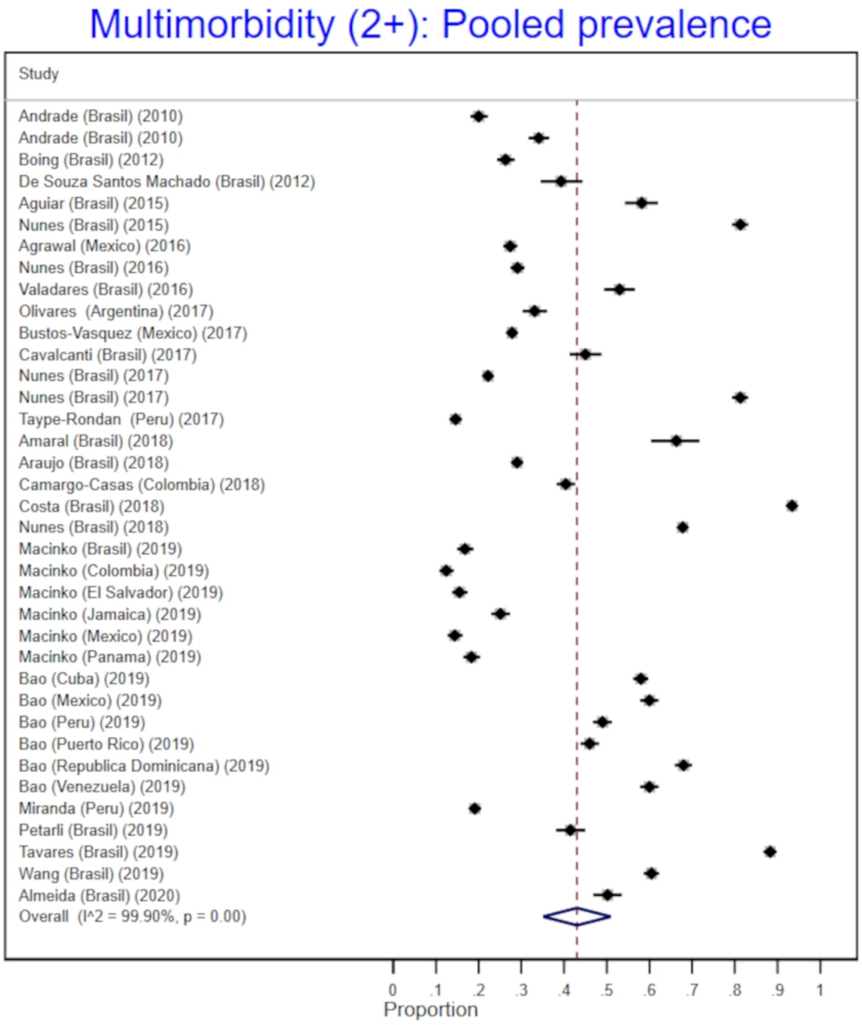

Figure 2: Forest plot of the pooled prevalence of multimorbidity defined as ≥2 chronic conditions

124x137mm (300 x 300 DPI)

SUPPLEMENTAL MATERIAL

E-Figure 1: Forest plot with pooled estimates of multimorbidity among males

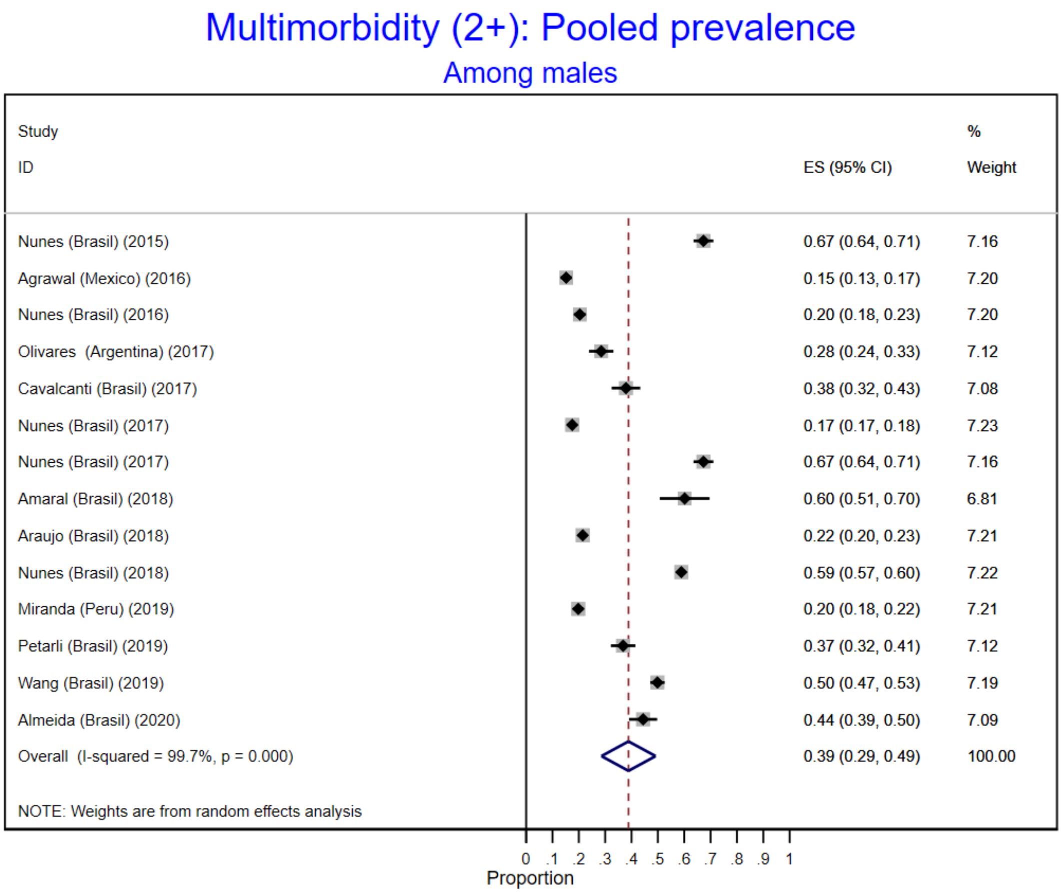

E-Figure 2: Forest plot with pooled estimates of multimorbidity among females

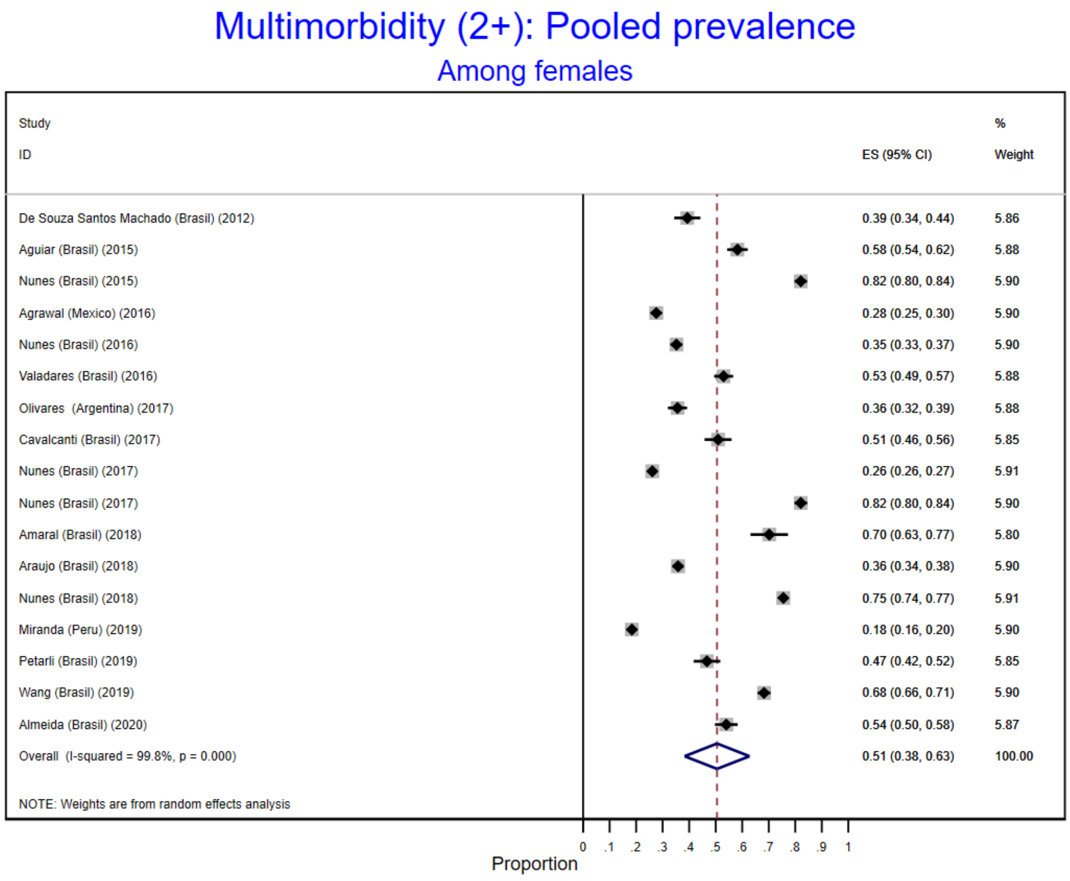

E-Figure 3: Forest plot with pooled estimates of multimorbidity among urban dwellers

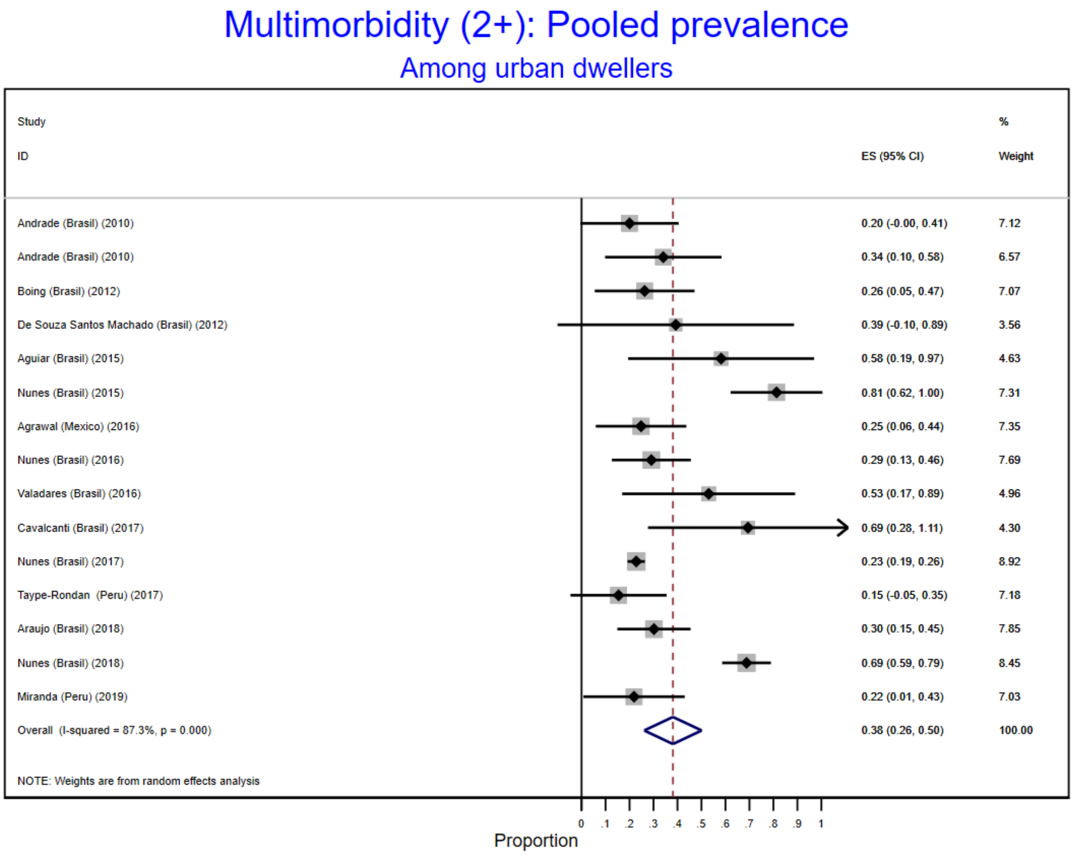

E-Figure 4: Forest plot with pooled estimates of multimorbidity among rural dwellers

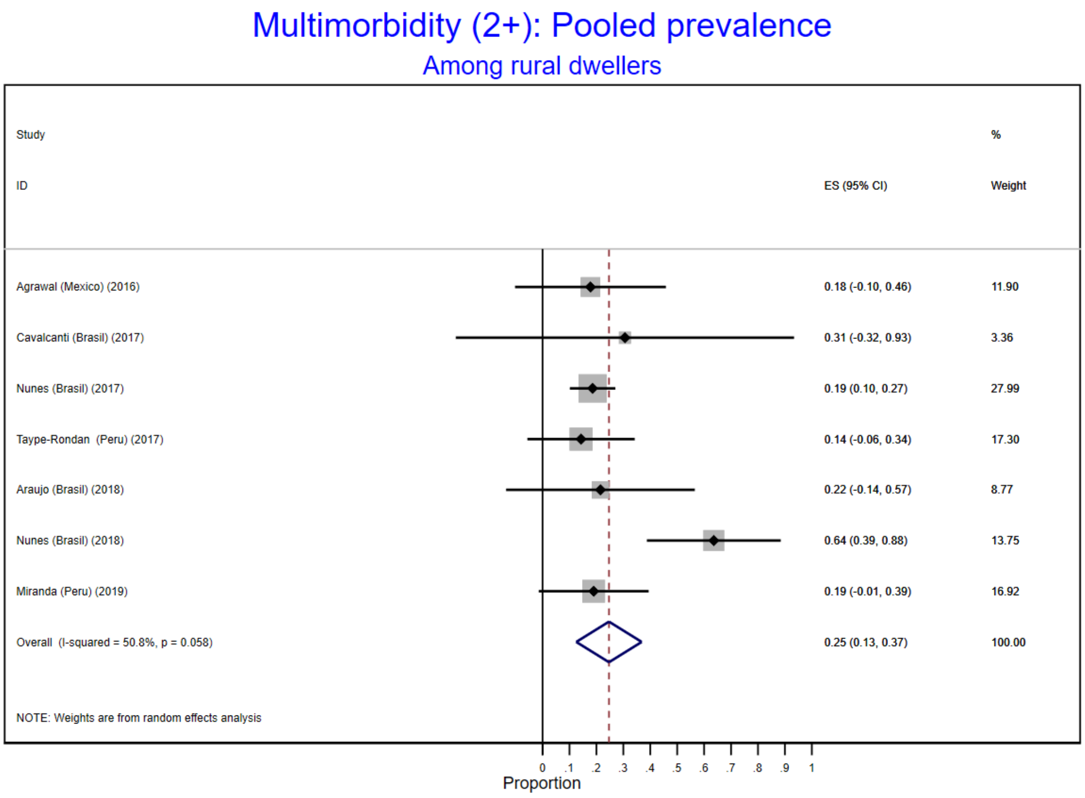

E-Table 1: Search terms and strategy using OVID (Embase, Medline and Global Health)

1	multimorbidit*.mp
2	multi-morbidit*.mp
3	(multi adj morbidit*).mp
4	multiple morbidit*.mp
5	(co adj morbidit*).mp
6	comorbidit*.mp
7	“multiple chronic conditions”.mp
8	“multiple chronic illnesses”.mp
9	“multiple chronic diseases”.mp
10	(poly adj morbidit*).mp
11	polymorbidit*.mp
12	poly-morbidit*.mp
13	1 or 2 or 3 or 4 or 5 or 6 or 7 or 8 or 9 or 10 or 11 or 12
14	(("Antigua and Barbuda") or ("Argentina") or ("Bahamas") or ("Barbados") or ("Belize") or ("Bolivia") or ("Brazil") or ("United States Virgin Islands") or ("British Virgin Islands") or ("Chile") or ("Colombia") or ("Costa Rica") or ("Cuba") or ("Dominica") or ("Dominican Republic") or ("Ecuador") or ("El Salvador") or ("Grenada") or ("Guatemala") or ("Guyana") or ("Haiti") or ("Honduras") or ("Jamaica") or ("Mexico") or ("Nicaragua") or ("Panama") or ("Paraguay") or ("Peru") or ("Puerto Rico") or ("Saint Kitts and Nevis") or ("Saint Lucia") or ("Saint Vincent and the Grenadines") or ("Suriname") or ("Trinidad and Tobago") or ("West Indies") or ("Uruguay") or ("Venezuela") or ("Latin America") or latin amer$ or ("South America") or south amer$ or ("Central America") or central amer$ or ("Caribbean Region"))
15	13 and 14
16	exp animals/ not humans.sh.
17	15 not 16
18	remove duplicates from 17

For peer review only

E-Table 2: Search terms and strategy using Scopus

(TITLE-ABS-KEY(multimorbidity$) OR TITLE-ABS-KEY(multi-morbidit$) OR TITLE-ABS-KEY(multi-morbidit$) OR TITLE-ABS-KEY(multi Pre/1 morbidit$) OR TITLE-ABS-KEY(multiple morbidit$) OR TITLE-ABS-KEY(co Pre/1 morbidit$) OR TITLE-ABS-KEY(comorbidit$) OR TITLE-ABS-KEY(multiple chronic conditions) OR TITLE-ABS-KEY(multiple chronic illnesses) OR TITLE-ABS-KEY(multiple chronic diseases) OR TITLE-ABS-KEY(poly Pre/1 morbidit$) OR TITLE-ABS-KEY(polymorbidit$) OR TITLE-ABS-KEY(poly-morbidit$)) AND (TITLE-ABS-KEY("Antigua and Barbuda") or TITLE-ABS-KEY("Argentina") or TITLE-ABS-KEY("Bahamas") or TITLE-ABS-KEY("Barbados") or TITLE-ABS-KEY("Belize") or TITLE-ABS-KEY("Bolivia") or TITLE-ABS-KEY("Brazil") or TITLE-ABS-KEY("United States Virgin Islands") or TITLE-ABS-KEY("British Virgin Islands") or TITLE-ABS-KEY("Chile") or TITLE-ABS-KEY("Colombia") or TITLE-ABS-KEY("Costa Rica") or TITLE-ABS-KEY("Cuba") or TITLE-ABS-KEY("Dominica") or TITLE-ABS-KEY("Dominican Republic") or TITLE-ABS-KEY("Ecuador") or TITLE-ABS-KEY("El Salvador") or TITLE-ABS-KEY("Grenada") or TITLE-ABS-KEY("Guatemala") or TITLE-ABS-KEY("Guyana") or TITLE-ABS-KEY("Haiti") or TITLE-ABS-KEY("Honduras") or TITLE-ABS-KEY("Jamaica") or TITLE-ABS-KEY("Mexico") or TITLE-ABS-KEY("Nicaragua") or TITLE-ABS-KEY("Panama") or TITLE-ABS-KEY("Paraguay") or TITLE-ABS-KEY("Peru") or TITLE-ABS-KEY("Puerto Rico") or TITLE-ABS-KEY("Saint Kitts and Nevis") or TITLE-ABS-KEY("Saint Lucia") or TITLE-ABS-KEY("Saint Vincent and the Grenadines") or TITLE-ABS-KEY("Suriname") or TITLE-ABS-KEY("Trinidad and Tobago") or TITLE-ABS-KEY("West Indies") or TITLE-ABS-KEY("Uruguay") or TITLE-ABS-KEY("Venezuela") or TITLE-ABS-KEY("Latin America") or TITLE-ABS-KEY(latin amer$) or TITLE-ABS-KEY("South America") or TITLE-ABS-KEY(south amer$) or TITLE-ABS-KEY("Central America") or TITLE-ABS-KEY(central amer$) or TITLE-ABS-KEY("Caribbean Region")) AND NOT DBCOLL(medl)

E-Table 3: Search terms and strategy using LILACS

((multi-morbilidad) OR (multimorbilidad) OR (co-morbilidad) OR (comorbilidad) OR (multiple morbilidad) OR (multiples condiciones cronicas) OR (multiples enfermedades cronicas) OR (multiples patologias cronicas) OR (poli-morbilidad) OR (polimorbilidad)) AND (("Antigua y Barbuda") OR ("Argentina") OR ("Aruba") OR ("Bahamas") OR ("Barbados") OR ("Belice") OR ("Bolivia") OR ("Brasil") OR ("Islas Vírgenes de los Estados Unidos") OR ("Islas Vírgenes Británicas") OR ("Islas Caimán") OR ("Chile") OR ("Colombia") OR ("Costa Rica") OR ("Cuba") OR ("Curazao") OR ("Dominica") OR ("Republica Dominicana") OR ("Ecuador") OR ("El Salvador") OR ("Granada") OR ("Guatemala") OR ("Guyana") OR ("Haití") OR ("Honduras") OR ("Jamaica") OR ("México") OR ("Nicaragua") OR ("Panamá") OR ("Paraguay") OR ("Perú") OR ("Puerto Rico") OR ("San Cristóbal y Nieves ") OR ("Santa Lucía") OR ("San Vicente y las Granadinas ") OR ("Surinam") OR ("Trinidad y Tobago") OR ("Turcas y Caicos ") OR ("Uruguay") OR ("Venezuela") OR ("América Latina") OR ("Latinoamérica") OR ("América del Sur") OR ("Sudamérica") OR ("Suramérica") OR ("América Central") OR ("Centroamérica") OR ("América del Centro") OR ("Caribe"))

E-Table 4: PRISMA Checklist

Section/topic	#	Checklist item	Reported on page #
TITLE			
Title	1	Identify the report as a systematic review, meta-analysis, or both.	01
ABSTRACT			
Structured summary	2	Provide a structured summary including, as applicable: background; objectives; data sources; study eligibility criteria, participants, and interventions; study appraisal and synthesis methods; results; limitations; conclusions and implications of key findings; systematic review registration number.	02
INTRODUCTION			
Rationale	3	Describe the rationale for the review in the context of what is already known.	03
Objectives	4	Provide an explicit statement of questions being addressed with reference to participants, interventions, comparisons, outcomes, and study design (PICOS).	03
METHODS			
Protocol and registration	5	Indicate if a review protocol exists, if and where it can be accessed (e.g., Web address), and, if available, provide registration information including registration number.	03-04
Eligibility criteria	6	Specify study characteristics (e.g., PICOS, length of follow-up) and report characteristics (e.g., years considered, language, publication status) used as criteria for eligibility, giving rationale.	04
Information sources	7	Describe all information sources (e.g., databases with dates of coverage, contact with study authors to identify additional studies) in the search and date last searched.	04
Search	8	Present full electronic search strategy for at least one database, including any limits used, such that it could be repeated.	Suppl material e-Tables 1-3
Study selection	9	State the process for selecting studies (i.e., screening, eligibility, included in systematic review, and, if applicable, included in the meta-analysis).	04
Data collection process	10	Describe method of data extraction from reports (e.g., piloted forms, independently, in duplicate) and any processes for obtaining and confirming data from investigators.	04

For peer review only

Data items	11	List and define all variables for which data were sought (e.g., PICOS, funding sources) and any assumptions and simplifications made.	04
Risk of bias in individual studies	12	Describe methods used for assessing risk of bias of individual studies (including specification of whether this was done at the study or outcome level), and how this information is to be used in any data synthesis.	04-05
Summary measures	13	State the principal summary measures (e.g., risk ratio, difference in means).	05
Synthesis of results	14	Describe the methods of handling data and combining results of studies, if done, including measures of consistency (e.g., I^2) for each meta-analysis.	05
Risk of bias across studies	15	Specify any assessment of risk of bias that may affect the cumulative evidence (e.g., publication bias, selective reporting within studies).	NA
Additional analyses	16	Describe methods of additional analyses (e.g., sensitivity or subgroup analyses, meta-regression), if done, indicating which were pre-specified.	05
RESULTS			
Study selection	17	Give numbers of studies screened, assessed for eligibility, and included in the review, with reasons for exclusions at each stage, ideally with a flow diagram.	05 and Figure 1
Study characteristics	18	For each study, present characteristics for which data were extracted (e.g., study size, PICOS, follow-up period) and provide the citations.	05 and Table 1
Risk of bias within studies	19	Present data on risk of bias of each study and, if available, any outcome level assessment (see item 12).	Suppl Material eTable 5
Results of individual studies	20	For all outcomes considered (benefits or harms), present, for each study: (a) simple summary data for each intervention group (b) effect estimates and confidence intervals, ideally with a forest plot.	06 and Suppl Material e-Table 6
Synthesis of results	21	Present results of each meta-analysis done, including confidence intervals and measures of consistency.	06 and Tables
Risk of bias across studies	22	Present results of any assessment of risk of bias across studies (see Item 15).	NA
Additional analysis	23	Give results of additional analyses, if done (e.g., sensitivity or subgroup analyses, meta-regression [see Item 16]).	06 and Suppl Material e-Figure 1-4

DISCUSSION			
Summary of evidence	24	Summarize the main findings including the strength of evidence for each main outcome; consider their relevance to key groups (e.g., healthcare providers, users, and policy makers).	06
Limitations	25	Discuss limitations at study and outcome level (e.g., risk of bias), and at review-level (e.g., incomplete retrieval of identified research, reporting bias).	07
Conclusions	26	Provide a general interpretation of the results in the context of other evidence, and implications for future research.	07-08
FUNDING			
Funding	27	Describe sources of funding for the systematic review and other support (e.g., supply of data); role of funders for the systematic review.	01

For peer review only

E-Table 5: Coverage and conditions included in the definition of multimorbidity

First author	Study coverage	Number of conditions	Conditions included in the definition of multimorbidity
Andrade [1]	Sub-national	8	Back pain, hypertension, asthma, headache, heart problems, diabetes, stroke, and cancer
Andrade [1]	Sub-national	15	Cognitive impairment, non-affective psychosis, panic attacks, generalized anxiety, any phobias, obsessive-compulsive disorders, depression, dysthymia, bipolar disorder, bulimia, alcohol use disorder, substance use disorder, nicotine dependence, somatoform disorder, and dissociative disorder
Boing [2]	Sub-national	11	Problems in the spine or on the back, arthritis or rheumatism, cancer, diabetes, bronchitis or asthma, hypertension, heart disease, chronic renal failure, tuberculosis, tendinitis or tenosynovitis, and cirrhosis
De Souza Santos Machado [3]	Sub-national	5	Depression, hypertension, diabetes mellitus, urinary incontinence, and insomnia
Aguiar [4]	Sub-national	10	Hypertension, myocardial infarction, stroke, diabetes, pulmonary emphysema, bronchitis/asthma, arthrosis, osteoporosis, cataract, and urinary incontinence
Nunes [5]	Sub-national	17	High blood pressure, diabetes, lung problem, heart problem, stroke, rheumatism, arthritis or arthrosis, disease in spinal column (any problem reported), cancer, kidney problem, cognitive impairment, depression, urinary incontinence, amputation in any part of the body, eyesight problem, hearing problem, problem chewing food, and falls
Agrawal [6]	National	9	Angina pectoris, arthritis, asthma, chronic lung disease, diabetes, hypertension, stroke, depression, and low visual acuity
Nunes [7]	Sub-national	11	High blood pressure, depression, heart disease, hypercholesterolemia, diabetes, bronchitis, asthma/wheezy bronchitis, hypothyroidism, osteoporosis, joint problems, and emphysema
Valadares [8]	Sub-national	11	Hypertension, diabetes mellitus, dyslipidemia, myocardial infarction, stroke, deep venous thrombosis or pulmonary embolus, asthma or bronchitis, chronic rheumatic diseases, depression, anxiety, and cancer
Bustos-Vazquez [9]	National	7	Cancer, systemic arterial hypertension, diabetes, depression, cerebral infarction, hypercholesterolemia, and cardiovascular diseases
Cavalcanti [10]	Sub-national	8	Systemic arterial hypertension, diabetes mellitus, rheumatism, pulmonary problems (asthma, bronchitis and pulmonary emphysema), stroke/cerebral ischemia, arthritis/osteoporosis, heart problems (coronary disease, angina, congestive disease, others), and depression

For peer review only

Nunes [11]	National	22	High blood pressure, spinal column problem, hypercholesterolemia, depression, diabetes, arthritis/rheumatism, asthma/wheezy bronchitis, cancer, other heart disease, work-related muscle-skeletal disorders, stroke, heart attack, kidney problem, heart failure, bronchitis, angina, emphysema, other lung disease, bipolar disorder, other mental disease, schizophrenia, and obsessive-compulsive disorder
Nunes [12]	Sub-national	17	Systemic arterial hypertension, diabetes mellitus, heart disease, pulmonary problems, stroke, rheumatism/arthritis/osteoarthritis, spine problems, cancer, kidney failure, cognitive deficit, depression, visual problems, urinary incontinence, amputation, auditory problems, problem or difficulty chewing food, and falls
Olivares [13]	Sub-national	9	Hypertension, diabetes, high cholesterol, AMI or stroke, asthma, hypothyroidism, celiac disease, cancer and other chronic diseases (arthritis, osteoarthritis, osteoporosis, depression, chronic allergies, COPD; gastritis, migraine, irritable bowel syndrome, epilepsy, psychosis, multiple sclerosis, psoriasis, gout disease, herniated disc, inflammatory vasculitis)
Taype-Rondan [14]	Sub-national	6	Hypertension, chronic bronchitis, depressive mood, type 2 diabetes, heart disease, and stroke
Amaral [15]	Sub-national	8	Hypertension, diabetes, cardiovascular disorder (poor circulation, cerebrovascular accident (CVA or stroke) and heart problem), musculoskeletal disorder (spinal problem, rheumatism, arthritis/ arthrosis and osteoporosis), insomnia, cataracts, anemia and gastrointestinal/urinary disorders
Araujo [16]	Sub-national	12	Hypertension, diabetes, high cholesterol, heart disease (heart attack, angina, heart failure or other), stroke, asthma or asthmatic bronchitis, arthritis or rheumatism, depression, pulmonary disease (pulmonary emphysema, chronic bronchitis or chronic obstructive pulmonary disease), cancer, chronic kidney disease, and chronic spinal problems (chronic back or neck pain, low back pain, sciatic pain, vertebral or disc problems)
Camargo-Casas [17]	Sub-national	12	Hypertension, diabetes, cancer, chronic obstructive pulmonary disease, heart attack, heart failure, stroke, arthritis or osteoarthritis, osteoporosis, gastroesophageal reflux disease, gastritis, and ulcer
Costa [18]	Sub-national	29	High blood pressure, diabetes, heart problem, heart failure, asthma, bronchitis, emphysema, arthritis, Parkinson's disease, kidney failure, hypercholesterolemia, seizure, stomach ulcer, osteoporosis, urinary incontinence, constipation, fecal incontinence, depression, glaucoma, deafness, difficulty swallowing, insomnia, fainting, rhinitis, difficulty speaking, stroke, obesity, mental disorders, and cancer
Nunes [19]	National	19	Systemic arterial hypertension, back problems, high cholesterol, cataract, arthritis or rheumatism, depression, diabetes, osteoporosis heart problem, glaucoma, emphysema

For peer review only

			chronic bronchitis or chronic obstructive pulmonary disease, cerebrovascular accident, cancer, asthma, chronic renal failure, diabetic retinopathy, macular degeneration, Parkinson's disease, and Alzheimer's disease
Bao [20]	National	15	Vision, hearing, joints, skin, gastrointestinal, stroke, chronic obstructive pulmonary disease, hypertension, diabetes, ischemic heart disease, heart failure, heart valve disease, depression, anxiety, and dementia
Macinko [21]	National	8	Arthritis, asthma, cancer, depression, diabetes, heart disease, high cholesterol, and hypertension
Miranda [22]	Sub-national	6	Alcohol disorder, asthma, chronic obstructive pulmonary disease, depression, diabetes, and hypertension
Petarli [23]	Sub-national	20	Heart arrhythmia, infarction, stroke, diabetes mellitus, herniated disk, arthrosis, repetitive strain injuries/work-related musculoskeletal disorders, renal disease, Parkinson's, Alzheimer's, hepatic cirrhosis, infertility, cancer, asthma, chronic bronchitis, and pulmonary emphysema, arterial hypertension, dyslipidemia, depression and thyroid disorders
Tavares [24]	Sub-national	25	Rheumatism, arthritis/arthrosis, osteoporosis, asthma or bronchitis, tuberculosis, embolism, systemic arterial hypertension, poor circulation (varicose veins), heart problems, diabetes mellitus, obesity, stroke, Parkinson's, urinary incontinence, fecal incontinence, intestinal constipation, sleeping problems, cataract, glaucoma, back problems, kidney problem, accident/trauma sequelae, malignant tumors, benign tumors, and vision problems
Wang [25]	Sub-national	16	Cardiovascular diseases (heart attack, heart disease, and stroke), hypertension, diabetes mellitus, arthritis, chronic musculoskeletal pain, headache or migraine, digestive, respiratory (seasonal allergies, asthma, obstructive pulmonary disease, and emphysema), cancer, neurological diseases (Parkinson disease, epilepsy, and multiple sclerosis), anxiety disorder, mood disorder, heavy drinking, impulse-control disorder, substance use disorder, and premenstrual dysphoria
Montes [26]	Sub-national	28	Systemic arterial hypertension, myocardial infarction, diabetes, heart failure, emphysema, asthma, bronchitis, arthritis, Parkinson's disease, renal failure, seizure, hypercholesterolemia, stomach ulcer, osteoporosis, urinary incontinence, constipation, fecal incontinence, depression, glaucoma, deafness, difficulty swallowing, insomnia, fainting, rhinitis, difficulty speaking, stroke, mental disorders and cancer
Padilha Pereira [27]	Sub-national	28	Arterial hypertension, diabetes, heart problems, heart failure, asthma, bronchitis, emphysema, arthritis, Parkinson's disease, kidney failure, hypercholesterolemia, convulsions, stomach ulcer, osteoporosis, urinary incontinence, constipation, fecal

For peer review only

			incontinence, depression, glaucoma, deafness, difficulty in swallowing, insomnia, fainting, rhinitis, difficulty in speaking, stroke, mental disorders, and cancer
Da Silva Almeida [28]	Sub-national	5	Hypertension, diabetes, dyslipidemias, obesity, and arthritis or rheumatism

For peer review only

E-Table 6: Risk of bias of included studies

First author	Representativeness	Sample size	Non-respondents	Exposure	Outcome	Statistical test
Andrade [1]	Low	Low	High	NA	High	NA
Boing [2]	Low	Low	Low	NA	High	NA
De Souza Santos Machado [3]	Low	Low	Low	NA	High	NA
Aguiar [4]	Low	Low	Low	NA	High	NA
Nunes [5]	Low	Low	Low	NA	High	NA
Agrawal [6]	Low	Low	High	NA	High	NA
Nunes [7]	Low	Low	Low	NA	High	NA
Valadares [8]	Low	Low	Low	NA	High	NA
Bustos-Vazquez [9]	Low	Low	Low	NA	High	NA
Cavalcanti [10]	Low	Low	Low	NA	High	NA
Nunes [11]	Low	Low	High	NA	High	NA
Nunes [12]	Low	Low	Low	NA	High	NA
Olivares [13]	Low	Low	Low	NA	Low	NA
Taype-Rondan [14]	Low	Low	Low	NA	Low	NA
Amaral [15]	Low	Low	High	NA	High	NA
Araujo [16]	Low	Low	Low	NA	High	NA
Camargo-Casas [17]	Low	Low	Low	NA	High	NA
Costa [18]	Low	Low	Low	NA	High	NA
Nunes [19]	Low	Low	Low	NA	High	NA
Bao [20]	Low	High	Low	NA	Low	NA
Macinko [21]	Low	High	High	NA	High	NA
Miranda [22]	Low	High	Low	NA	Low	NA
Petarli [23]	Low	Low	Low	NA	High	NA
Tavares [24]	Low	Low	Low	NA	High	NA
Wang [25]	Low	Low	Low	NA	High	NA
Montes [26]	Low	Low	Low	NA	High	NA
Padilha Pereira [27]	Low	Low	Low	NA	High	NA
Da Silva Almeida [28]	Low	Low	Low	NA	Low	NA

For peer review only

REFERENCES

1. Andrade LH, Benseñor IM, Viana MC, Andreoni S, Wang YP. Clustering of psychiatric and somatic illnesses in the general population: multimorbidity and socioeconomic correlates. *Braz j med biol res* 2010;43(5):483-91.
2. Boing AF, Melo GR, Boing AC, Moretti-Pires RO, Peres KG, Peres MA. Associação entre depressão e doenças crônicas: um estudo populacional. *Rev saúde pública* 2012;46(4):617-23.
3. de Souza Santos Machado V, Valadares ALR, da Costa-Paiva LS, Moraes SS, Pinto-Neto AM. Multimorbidity and associated factors in Brazilian women aged 40 to 65 years: a population-based study. *Menopause (New York, NY)* 2012;19(5):569-75.
4. Aguiar LB, Baccaro LF, de Souza Santos Machado V, Pinto-Neto AM, Costa-Paiva L. Disability and multimorbidity in women older than 50 years: a population-based household survey. *Menopause (New York, NY)* 2015;22(6):660-6.
5. Nunes BP, Thume E, Facchini LA. Multimorbidity in older adults: magnitude and challenges for the Brazilian health system. *BMC public health* 2015;15:1172.
6. Agrawal S, Agrawal PK. Association Between Body Mass index and Prevalence of Multimorbidity in Low-and Middle-income Countries: A Cross-Sectional Study. *International journal of medicine and public health* 2016;6(2):73-83.
7. Nunes BP, Camargo-Figuera FA, Guttier M, de Oliveira PD, Munhoz TN, Matijasevich A, et al. Multimorbidity in adults from a southern Brazilian city: occurrence and patterns. *International journal of public health* 2016;61(9):1013-20.
8. Valadares ALR, Lui-Filho JF, Costa-Paiva L, Pinto-Neto AM. Middle-aged female sexual dysfunction and multimorbidity: a population-based study. *Menopause (New York, NY)* 2016;23(3):304-10.
9. Bustos-Vazquez E, Fernandez-Nino JA, Astudillo-Garcia CI. Self-rated health, multimorbidity and depression in Mexican older adults: Proposal and evaluation of a simple conceptual model. *Biomedica : revista del Instituto Nacional de Salud* 2017;37(0):92-103.
10. Cavalcanti G, Doring M, Portella MR, Bortoluzzi EC, Mascarelo A, Dellani MP. Multimorbidity associated with polypharmacy and negative self-perception of health. *Rev bras geriatr gerontol (Online)* 2017;20(5):634-42.
11. Nunes BP, Chiavegatto Filho ADP, Pati S, Cruz Teixeira DS, Flores TR, Camargo-Figuera FA, et al. Contextual and individual inequalities of multimorbidity in Brazilian adults: a cross-sectional national-based study. *BMJ open* 2017;7(6):e015885.
12. Nunes BP, Soares MU, Wachs LS, Volz PM, Saes MdO, Duro SMS, et al. Hospitalization in older adults: association with multimorbidity, primary health care and private health plan. *Rev saúde pública* 2017;51:43-.
13. Olivares DEV, Chambi FRV, Chani EMM, Craig WJ, Pacheco SOS, Pacheco FJ. Risk Factors for Chronic Diseases and Multimorbidity in a Primary Care Context of Central Argentina: A Web-Based Interactive and Cross-Sectional Study. *International journal of environmental research and public health* 2017;14(3).
14. Taype-Rondan A, Abbs ES, Lazo-Porras M, Checkley W, Gilman RH, Smeeth L, et al. Association between chronic conditions and health-related quality of life: differences by level of urbanization in Peru. *Quality of life research : an international journal of quality of life aspects of treatment, care and rehabilitation* 2017;26(12):3439-47.

For peer review only

15. Amaral TLM, Amaral CdA, Lima NSd, Herculano PV, Prado PRd, Monteiro GTR. Multimorbidity, depression and quality of life among elderly people assisted in the Family Health Strategy in Senador Guiomard, Acre, Brazil. *Multimorbidade, depressao e qualidade de vida em idosos atendidos pela Estrategia de Saude da Familia em Senador Guiomard, Acre, Brasil* 2018;23(9):3077-84.
16. Araujo MEA, Silva MT, Galvao TF, Nunes BP, Pereira MG. Prevalence and patterns of multimorbidity in Amazon Region of Brazil and associated determinants: a cross-sectional study. *BMJ open* 2018;8(11):e023398.
17. Camargo-Casas S, Suarez-Monsalve S, Zepeda MUP, Garcia-Pena C, Cano-Gutierrez CA. [Multimorbidity, Depressive Symptoms, and Self-Reported Health in Older Adults: a Secondary Analysis of the Sabe Bogota Study]. *Revista de investigacion clinica; organo del Hospital de Enfermedades de la Nutricion* 2018;70(4):192-7.
18. Costa CdS, Flores TR, Wendt A, Neves RG, Tomasi E, Cesar JA, et al. Inequalities in multimorbidity among elderly: a population-based study in a city in Southern Brazil. *Cad Saúde Pública (Online)* 2018;34(11):e00040718-e.
19. Nunes BP, Batista SRR, Andrade FBd, Souza Junior PRBd, Lima-Costa MF, Facchini LA. Multimorbidity: The Brazilian Longitudinal Study of Aging (ELSI-Brazil). *Revista de saude publica* 2018;52Suppl 2(Suppl 2):10s.
20. Bao J, Chua K-C, Prina M, Prince M. Multimorbidity and care dependence in older adults: a longitudinal analysis of findings from the 10/66 study. *BMC public health* 2019;19(1):585.
21. Macinko J, Andrade FCD, Nunes BP, Guanais FC. Primary care and multimorbidity in six Latin American and Caribbean countries. *Rev panam salud pública* 2019;43:e8-e.
22. Miranda JJ, Bernabe-Ortiz A, Gilman RH, Smeeth L, Malaga G, Wise RA, et al. Multimorbidity at sea level and high-altitude urban and rural settings: The CRONICAS Cohort Study. *Journal of Comorbiddity* 2019;9:1-10.
23. Petarli GB, Cattafesta M, Sant'Anna MM, Bezerra OMdPA, Zandonade E, Salaroli LB. Multimorbidity and complex multimorbidity in Brazilian rural workers. *PLoS ONE* 2019;14(11):e0225416.
24. Tavares DMDS, Pelizaro PB, Pegorari MS, Paiva MMd, Marchiori GF. Prevalence of self-reported morbidities and associated factors among community-dwelling elderly in Uberaba, Minas Gerais, Brazil. *Prevalencia de morbidades autorreferidas e fatores associados entre idosos comunitarios de Uberaba, Minas Gerais, Brasil* 2019;24(9):3305-13.
25. Wang Y-P, Nunes BP, Coelho BM, Santana GL, do Nascimento CF, Viana MC, et al. Multilevel Analysis of the Patterns of Physical-Mental Multimorbidity in General Population of Sao Paulo Metropolitan Area, Brazil. *Scientific reports* 2019;9(1):2390.
26. Montes MC, Bortolotto CC, Tomasi E, Gonzalez MC, Barbosa-Silva TG, Domingues MR, et al. Strength and multimorbidity among community-dwelling elderly from southern Brazil. *Nutrition* 2020;71.
27. Padilha Pereira B, Cardozo Bortolotto C, Tomasi E, Gonzalez MC, Gomes AP, Goncalves H, et al. Food consumption and multimorbidity among non-institutionalized elderly people in Pelotas, 2014: a cross-sectional study. *Epidemiol serv saúde* 2020;29(3):e2019050.
28. da Silva Almeida IL, dos Santos SR, Morbeck de Queiroz B, de Freitas Mussi RF. Lifestyle, morbidity and multimorbidity in adult Quilombolas. *ABCS health sci* 2020;45:1325.

For peer review only

Multimorbidity in Latin America and the Caribbean: A systematic review and meta-analysis

Journal:	*BMJ Open*
Manuscript ID	bmjopen-2021-050409.R1
Article Type:	Original research
Date Submitted by the Author:	23-Jun-2021
Complete List of Authors:	Huaquía-Díaz, Alessandra; Universidad Científica del Sur<br>Chalán-Dávila, Tarick; Universidad Científica del Sur<br>Carrillo-Larco, Rodrigo; Imperial College London, ; CRONICAS Center of Excellence in Chronic Diseases<br>Bernabe-Ortiz, Antonio; Universidad Peruana Cayetano Heredia, CRONICAS Centre of Excellence in Chronic Diseases; Universidad Cientifica del Sur
**Primary Subject Heading**:	Epidemiology
Secondary Subject Heading:	Global health, Public health
Keywords:	EPIDEMIOLOGY, PUBLIC HEALTH, Hypertension < CARDIOLOGY, DIABETES & ENDOCRINOLOGY

SCHOLARONE™
Manuscripts

I, the Submitting Author has the right to grant and does grant on behalf of all authors of the Work (as defined in the below author licence), an exclusive licence and/or a non-exclusive licence for contributions from authors who are: i) UK Crown employees; ii) where BMJ has agreed a CC-BY licence shall apply, and/or iii) in accordance with the terms applicable for US Federal Government officers or employees acting as part of their official duties; on a worldwide, perpetual, irrevocable, royalty-free basis to BMJ Publishing Group Ltd ("BMJ") its licensees and where the relevant Journal is co-owned by BMJ to the co-owners of the Journal, to publish the Work in this journal and any other BMJ products and to exploit all rights, as set out in our licence.

The Submitting Author accepts and understands that any supply made under these terms is made by BMJ to the Submitting Author unless you are acting as an employee on behalf of your employer or a postgraduate student of an affiliated institution which is paying any applicable article publishing charge ("APC") for Open Access articles. Where the Submitting Author wishes to make the Work available on an Open Access basis (and intends to pay the relevant APC), the terms of reuse of such Open Access shall be governed by a Creative Commons licence – details of these licences and which Creative Commons licence will apply to this Work are set out in our licence referred to above.

Other than as permitted in any relevant BMJ Author's Self Archiving Policies, I confirm this Work has not been accepted for publication elsewhere, is not being considered for publication elsewhere and does not duplicate material already published. I confirm all authors consent to publication of this Work and authorise the granting of this licence.

For peer review only

Multimorbidity in Latin America and the Caribbean: A systematic review and meta-analysis

Alessandra M. Huaquía-Diaz, BS[1]; Tarick S. Chalán-Dávila, BS[1]; Rodrigo M. Carrillo-Larco, MD[2,3,4]; Antonio Bernabé-Ortiz, MD, MPH, PhD[1,2,5]

1. Universidad Científica del Sur, Lima, Peru
2. CRONICAS Center of Excellence in Chronic Diseases, Universidad Peruana Cayetano Heredia, Lima, Peru
3. Department of Epidemiology and Biostatistics, School of Public Health, Imperial College London, London, UK
4. Universidad Católica Los Ángeles de Chimbote, Instituto de Investigación, Chimbote, Peru
5. School of Public Health and Administration, Universidad Peruana Cayetano Heredia, Lima, Peru

Corresponding author:

Antonio Bernabe-Ortiz, MD, MPH, PhD

Universidad Científica del Sur, Lima, Peru

Email: Antonio.Bernabe@upch.pe

For peer review only

ABSTRACT

Objective: To estimate the pooled prevalence of multimorbidity (≥2 non-communicable diseases in the same individual) among adults of the general population of LAC.

Design: Systematic review and meta-analysis.

Data sources: Medline, Embase, Global Health, Scopus, and LILACS up to July 1st, 2020.

Eligibility criteria for selecting studies: The outcome was the prevalence of multimorbidity. Reports were selected whether they enrolled adult individuals (age ≥18 years) from the general population.

Data extraction and synthesis: Reviewers extracted relevant data and assessed risk of bias independently. A random-effects meta-analysis was conducted to report pooled prevalence estimates of multimorbidity; pooled estimates by pre-specified sub-groups (e.g., national studies) was also pursued.

Results: From 5,830 results, we selected 28 reports, mostly from Brazil and 16 were based on a nationally representative sample. From the 28 selected reports, 26 were further included in the meta-analysis revealing a pooled multimorbidity prevalence of 43% (95% CI: 35% - 51%; I^2: 99.9%). When only reports with a nationally representative sample were combined, the pooled prevalence was 37% (95% CI: 27%-47%; I^2: 99.9%). When the ascertainment of multimorbidity was based on self-reports alone, the pooled prevalence was 40% (95% CI: 31% - 48%; I^2: 99.9%); this raised to 52% (95% CI: 33% - 70%; I^2: 99.9%) for reports including self-reported and objective diagnosis.

Conclusions: Our results complement and advance those from global efforts by incorporating much more reports from LAC. We revealed a larger presence of multimorbidity in LAC than previously reported.

Key words: Multimorbidity, systematic review, prevalence, Latin American and the Caribbean

For peer review only

STRENGHTS AND LIMITATIONS OF THIS STUDY

- Most of the analyzed data came from Brazin preventing region representativity.
- High heterogeneity was present in the analysis, mainly due to different conditions included to define multimorbidity.
- Participant’s age seems to explain high heterogeneity as age range in studies is very wide.
- A bias due to self-reporting of conditions may underestimate the real burden of multimorbidity in LAC region.

For peer review only

INTRODUCTION

The Academy of Medical Sciences defines multimorbidity as "*the existence of two or more medical chronic conditions in a single individual*" [1]. Subjects with multimorbidity tend to increase healthcare utilization and costs of primary and secondary care services [2]; also, multimorbidity has a subsequent impact on quality of life [3].

In high-income countries, multimorbidity rates are heterogeneous but seem depend on individual's age. Thus, in a cross-sectional study using the dataset of medical practices in Scotland, the prevalence of multimorbidity was 23% using a list of 40 conditions, and was present mainly in older people [4]. Moreover, multimorbidity seems to be increasing in low- and middle-income countries, where data is yet scarce [5]. The increase of life expectancy in Latin American and the Caribbean (LAC) has been associated with greater incidence of noncommunicable conditions (NDC) [6], with the consequent emergence of multimorbidity.

Multimorbidity prevalence has been explored and summarized in some systematic reviews around the world [7, 8] and results ranged from 5% to 98%; nevertheless, their results were mainly informed by data from high-income countries. In a relatively recent systematic review [9], 31 low- and middle-income countries (LMIC) were included with a prevalence of multimorbidity of 30%, compared to an estimate of 38% in high-income countries. However, only nine studies from the LAC region were included. Moreover, pooled estimates by region were not elucidated, preventing to have appropriate indicators of the burden of multimorbidity in this region. A more recent systematic review has reported a pooled prevalence of multimorbidity in LMIC between 3% and 90%, with almost 80% of the studies being from Brazil, China, South Africa, India, Mexico, and Iran [10].

The lack of evidence about multimorbidity may have important consequences for research, public health, and clinical management in LAC region. For example, multimorbidity was not appropriately defined up to 2018; in addition, whether estimates depend upon sex or setting characteristics (i.e., rural vs. urban areas) should be also studied. Moreover, the need of surveillance systems to assess multimorbidity may be elucidated as these estimates have not been estimated in LAC region. Thus, from the public health perspective may not be easy to take appropriate decisions or implement adequate strategies to tackle the problem of multimorbidity.

As a result, we aimed at providing robust evidence about multimorbidity prevalence estimates in LAC region through a systematic review and meta-analysis of population-based surveys. These

For peer review only

results evidence may help to guide interventions and policies so that they can focus on the most pressing frequent multimorbidity phenotypes in LAC.

METHODS

Protocol

This systematic review was registered in PROSPERO under the code CRD42020196177. We aimed to identify the population-based prevalence of multimorbidity in LAC, and to study whether this prevalence varies by multimorbidity definitions, sex, and urban-rural settings.

Eligibility criteria

Reports were selected whether they enrolled adult individuals (age ≥18 years) from the general population. We focused on LAC populations; therefore, we excluded studies with LAC individuals in countries outside the LAC region, and studies with only foreign subjects in LAC nations. Population-based studies were defined as those following a random sampling approach, and such sample was taken from the general population. On the contrary, studies addressing specific populations (e.g., pregnant women), those with individuals with specific conditions (e.g., people with hypertension), or subjects with specific risk factors (e.g., obese or alcohol disorders) were excluded.

The outcome of interest was the prevalence of multimorbidity, defined as the existence of ≥ 2 chronic conditions in the same person [1]. Different definitions of multimorbidity were considered in this review (e.g., ≥2, ≥3, ≥4, or ≥5 conditions) as the current definition (≥2 conditions) is relatively recent. In addition, the presence of chronic conditions could have been measured, self-reported or a combination of these approaches.

Information sources

The search was conducted on January 10, 2020, and then updated on July 1st, 2020. We used Ovid search engine, comprising Medline, Embase and Global Health databases; and in parallel, we also searched Scopus and LILACS. In all of these, searching was carried out without time or language restriction. The search strategy and terms used is detailed in Supplementary Material: e-Table 1-3.

Study selection

Results from each search engine were downloaded and saved in EndNote where duplicates were removed. After that, information was transferred to Rayyan, an open-access online tool for

For peer review only - http://bmjopen.bmj.com/site/about/guidelines.xhtml

systematic reviews [11]. Titles and abstracts were reviewed by two researchers in an independent way, and disagreements were solved by a third party. After this screening phase, selected reports were downloaded and independently studied in detail by two researchers, and similarly, discrepancies were solved by a third party. Finally, selected studies were examined again to check for data duplication, i.e., different reports that used the same data (e.g., multiple reports based on the same underlying data). In this case, the paper with more information or the one with the largest sample size was included in the review and meta-analysis.

Data collection

An extraction template form was built by the authors and tested with a random sample of selected studies. After starting data collection, the form was not further modified. This form included study characteristics: study design, country, if it was a nationally representative sample, sample size, year of data collection, age range, age mean, proportion of women, and if it was urban, rural or both. The extraction form also collated the definition of multimorbidity used, self-reported or a combination of self-reported and measured, the number and a list of chronic conditions studies, and prevalence of multimorbidity (overall, by sex, and by rural or urban settings).

Risk of bias of individual studies

Risk of bias of selected studies was evaluated using the Newcastle-Ottawa Quality Assessment Scale adapted for cross-sectional studies as in a previous report [12]. This tool is focused on selection process (representativeness, sample size, and non-respondents), and the assessment of the outcome (independent blind assessment, self-report or not description). The items of this scale were implemented in an Excel spreadsheet and assessed independently by two reviewers; discrepancies were solved by a third party.

For peer review only

Summary measures

Our systematic review followed the Preferred Reporting Items for Systematic Review and Meta-Analysis (PRISMA) toolkit (See Checklist in Supplemental Material: e-Table 4). We presented a qualitative and quantitative summary. The qualitative summary described the characteristics of the study (as listed above), whereas the quantitative summary explored pooled prevalence estimates.

Statistical analyses were performed using Stata version 16 for Windows (StataCorp, College Station, TX, US). The "*metaprop*" command attains a pooled estimate as a weighted average, by fitting a logistic-normal random-effect model without covariates, but random intercepts [13]. After

that, the pooled estimate was calculated using the Freeman-Tukey Arcsine Transformation as suggested in literature [14].

Because the selected studies were different in nature, scope (e.g., national surveys versus community/subnational studies, or urban vs. rural settings) and sample size, we also conducted random-effects meta-analysis for comparing estimates in specific subgroups (e.g., national studies only). In addition, stratified meta-analysis (e.g., by sex, and by urban/rural settings) was pursued. Sensitivity analyses were also conducted focused on age by including studies with individuals aged 50 years and over, and also with studies whose data was collected from 2010 and onwards. Besides, as many of the studies were conducted in Brazil, a comparison of pooled estimated between Brazil and other countries together was also pursued.

Finally, meta-regression was also conducted as a high level of heterogeneity was expected. Meta-regression command in STATA investigates if between-study heterogeneity can be explained by one or more of the variables included in the review.

Patient and Public Involvement

Patients or the public were not involved in the design, or conduct, or reporting, or dissemination plans of our research.

Ethical considerations

The systematic review protocol was reviewed and approved by the Ethical Committee in Universidad Científica del Sur, Lima, Peru.

RESULTS

Selection process

The search strategy yielded 5,830 titles and abstracts after removing duplicates (See Figure 1); of these 66 were studied in detail and 28 reports met the inclusion criteria. For quantitative analyses, 26 reports were included (2 were excluded as the definition of multimorbidity was ≥5 chronic conditions), representing information for 12 LAC countries.

General characteristics of selected studies

As shown in Table 1, Brazil contributes with a total of 20 data points [15-34]; Mexico with 4 data points [23, 35-37]; Peru with 3 data points [36, 38, 39]; Colombia with 2 data points [23, 40]; and Argentina [41], Cuba [36], Dominican Republic [36], El Salvador [23], Jamaica [23], Panama [23],

For peer review only

Puerto Rico [36], and Venezuela [36] with 1 data point each. On the other hand, only 16 studies analyzed a nationally representative sample (Table 1).

Data from 37 country-level points were used to calculate pooled estimates using a definition of multimorbidity of ≥2 chronic conditions. Only one country (Brazil) used an additional definition of multimorbidity (i.e., ≥3 chronic conditions) in 8 different reports. Fourteen reports had information to calculate estimates for urban settings [15, 17-19, 22, 25-27, 29, 33, 35, 38, 39, 42], whereas 7 had information from rural areas [18, 25, 27, 35, 38, 39, 42].

Finally, the number of conditions evaluated to define multimorbidity ranged from 5 to 29, with a mean of 12.3 (SD: 5.7) conditions (Supplemental Material: e-Table 5). Hypertension and type 2 diabetes were the only conditions included in all the definitions of multimorbidity.

Synthesis of results

The meta-analysis included 26 studies with information with a pooled estimate of multimorbidity defined as ≥2 chronic conditions of 43% (95% CI: 35% - 51%; I^2: 99.9%; See Figure 2); whereas the pooled estimate for multimorbidity using ≥3 chronic conditions was 40% (95% CI: 22% - 57%; I^2: 99.9%).

As many of the studies were from Brazil, the pooled estimate of multimorbidity (≥2 chronic conditions) for this country was 50% (95% CI: 37% - 63%; I^2: 99.9%), whereas the pooled estimate for other countries together was 35% (95% CI: 26% - 43%; I^2: 99.7%). Nationally representative samples had a pooled estimate of multimorbidity of 37% (95% CI: 27% - 47%; I^2: 99.9%), whilst this estimate was 48% (95% CI: 35% - 61%; I^2: 99.9%) for non-nationally representative samples. Similarly, when multimorbidity was assessed as self-reported, pooled prevalence was 40% (95% CI: 31% - 48%; I^2: 99.9%), but the prevalence was 52% (95% CI: 33% - 70%; I^2: 99.9%) for those which have objectively measured and self-reported chronic conditions.

On the other hand, when analysis was conducted using studies whose data was collected from 2010 and onwards, pooled prevalence of multimorbidity was 48% (95% CI: 34% - 61%; I^2: 99.9%). This approach was used because time can have an impact on estimations due to the health transition in LMIC. Similarly, when assessing only studies including subjects of 50 years and over, the pooled prevalence of multimorbidity was 62% (95% CI: 51% - 73%; I^2: 99.9%).

In stratified analyses, pooled prevalence of multimorbidity was 38.9% (95% CI: 28.6% - 49.1%; I^2: 99.7%) for males and 50.5% (95% CI: 38.3% - 62.7%; I^2: 99.8%) for females. These estimates

For peer review only

were 38.1% (95% CI: 26.1% - 50.1%; I^2: 87.3%) and 24.7% (95% CI: 12.5% - 36.8%; I^2: 50.8%) for urban and rural dwellers, respectively (See Figures in Supplementary Material: e-Figure 1-4). In meta-regression analysis, predictors positively associated with heterogeneity were age, number of chronic conditions used for defining multimorbidity, and the proportion of women in the study.

Risk of bias

All reviewed studies had low risk of bias (Supplementary Material: e-Table 6). Nevertheless, sample size was not justified in three studies [23, 36, 38], and the outcome was self-reported in most of the studies except in 5 of them [21, 36, 38, 39, 41].

For peer review only

DISCUSSION

Summary of evidence

This systematic review provides a comprehensive analysis of the burden of multimorbidity in the LAC region. Accordingly, the pooled prevalence of multimorbidity was 43%. Nevertheless, there was a high heterogeneity among studies included, and results varied by the characteristics of the study as well as some of population characteristics. Thus, the pooled prevalence was lower in nationally representative compared to sub-national samples, whereas estimates were higher when only studies with measured and self-reported chronic conditions were included. In addition, age seems to be an important predictor as the prevalence of multimorbidity among those aged 50 years and over was high compared to the pooled estimate of multimorbidity. Finally, the pooled prevalence of multimorbidity was higher among females compared to males, highlighting the link between sex and multimorbidity, and among urban compared to those rural dwellers highlighting perhaps better access to diagnostic care in urban sites.

Limitations of the review

There are some limitations in this review that should be highlighted. First, high heterogeneity is present in almost all the results. This finding may be attributable to age group inclusion criteria since a great proportion of studies enrolled individuals aged ≥50+ years and the pooled estimate of multimorbidity was high in this group. In addition, the proportion of women enrolled in each individual study may be relevant as pointed out by the meta-regression analysis. Second, the number of chronic conditions as well as the list of them used to define multimorbidity is very dissimilar. Defining specific clusters of multimorbidity is needed to guarantee comparability between studies, but this is not usually reported. Therefore, it is relevant to standardize the

definition of multimorbidity and the conditions included in such definition to estimate which clusters of multimorbidity are more frequent and relevant for LAC region. In addition to that, before recent definition of multimorbidity, some reports used other different definitions (≥ 3 or ≥ 5 chronic conditions); which could affect pooled results. Fortunately, analysis was possible to include only those with ≥2 chronic conditions. Third, most of the studies included in the review were from Brazil, preventing inferability to the whole region, but also highlighting the need of population-based studies on multimorbidity in other countries of the region. Of note, the pooled prevalence of multimorbidity was higher in Brazil compared to other countries, perhaps because Brazilian researchers have addressed a common definition of multimorbidity, using a list of 12 conditions. Finally, a bias due to self-reporting can affect our results. Therefore, whether multimorbidity is defined by self-report or by more objective measurements may have an impact on prevalence estimates. Awareness of some chronic conditions are usually low and varying; for example, hypertension and diabetes awareness is around 64% and 78%, respectively in urban areas of Latin America [43], but tend to be lower in rural settings [44]. Thus, our results may be underestimating the real burden of multimorbidity in the LAC region.

Public Health Relevance

Global trends suggest that multimorbidity is a public health challenge; thus, understanding the epidemiology of multimorbidity in LAC region may be relevant as this issue has received little attention from researchers, but especially, policymakers. Moreover, much of the response of the health system has been developed based on one specific condition or specific body system, instead of an integral approach whereby multiple conditions are addressed synergistically. Therefore, disperse and heterogeneous information have been available for this systematic review.

Our results highlight the need of implementing a surveillance system focused on multimorbidity. This can be done by including some specific conditions in routine health surveys (Demographic Health Surveys or similar) and other population-based research studies. In addition, these surveys may include the most common clusters of multimorbidity, and those with higher morbidity and mortality [45] , however, such relevant clusters need to be appropriately defined.

Our results also imply that health systems need to be adapted to face the challenge of multimorbidity which increases healthcare use and costs related to primary and secondary prevention [2]. This adaptation process includes the appropriate training of human resources as well as improving of health services infrastructure and care delivery. It is also needed to develop

For peer review only

guidelines for multimorbidity care as that of NICE that includes clinical assessment and adequate management [46], but also highlight related issues as polypharmacy and life expectancy. Therefore, a holistic approach may be needed to tackle this global health problem.

Conclusions

Our systematic review shows that four of ten participants have multimorbidity at the population level in LAC. There is, however, a marked variability, depending on participant's age and the number of chronic conditions assessed, highlighting the need of better designed and standardized studies to inform the landscape of multimorbidity in LAC.

Statements

Contributorship statement: TSC-D, AMH-D and AB-O conceived the idea of the manuscript. TSC-D and AMH-D conducted the review with support of AB-O and RMC-L. RMC-L and AB-O conducted the statistical analysis. TSC-D and AMH-D drafted the first version of the manuscript, with relevant comments and suggestions of RMC-L and AB-O. All the authors approved the version submitted for publication.

Competing interests: None declared.

Funding: Rodrigo M Carrillo-Larco is supported by a Wellcome Trust International Training Fellowship (214185/Z/18/Z).

Data sharing statement: All the information of the present systematic review is included in the manuscript. There is no additional data available.

REFERENCES

1. The Academy of Medical Sciences. *Multimorbidity: a priority for health research*. London, UK: Academy of Medical Sciences; 2018.
2. Soley-Bori M, Ashworth M, Bisquera A, Dodhia H, Lynch R, Wang Y, et al. Impact of multimorbidity on healthcare costs and utilisation: a systematic review of the UK literature. *Br J Gen Pract* 2021;71(702):e39-e46.
3. Makovski TT, Schmitz S, Zeegers MP, Stranges S, van den Akker M. Multimorbidity and quality of life: Systematic literature review and meta-analysis. *Ageing Res Rev* 2019;53:100903.

For peer review only

4. Barnett K, Mercer SW, Norbury M, Watt G, Wyke S, Guthrie B. Epidemiology of multimorbidity and implications for health care, research, and medical education: a cross-sectional study. *Lancet* 2012;380(9836):37-43.
5. Banerjee A, Hurst J, Fottrell E, Miranda JJ. Multimorbidity: Not Just for the West. *Glob Heart* 2020;15(1):45.
6. Bilal U, Hessel P, Perez-Ferrer C, Michael YL, Alfaro T, Tenorio-Mucha J, et al. Life expectancy and mortality in 363 cities of Latin America. *Nat Med* 2021.
7. Violan C, Foguet-Boreu Q, Flores-Mateo G, Salisbury C, Blom J, Freitag M, et al. Prevalence, determinants and patterns of multimorbidity in primary care: a systematic review of observational studies. *PLoS ONE* 2014;9(7):e102149.
8. Pati S, Swain S, Hussain MA, van den Akker M, Metsemakers J, Knottnerus JA, et al. Prevalence and outcomes of multimorbidity in South Asia: a systematic review. *BMJ open* 2015;5(10):e007235.
9. Nguyen H, Manolova G, Daskalopoulou C, Vitoratou S, Prince M, Prina AM. Prevalence of multimorbidity in community settings: A systematic review and meta-analysis of observational studies. *J Comorb* 2019;9:2235042x19870934.
10. Abebe F, Schneider M, Asrat B, Ambaw F. Multimorbidity of chronic non-communicable diseases in low- and middle-income countries: A scoping review. *J Comorb* 2020;10:2235042x20961919.
11. Ouzzani M, Hammady H, Fedorowicz Z, Elmagarmid A. Rayyan-a web and mobile app for systematic reviews. *Syst Rev* 2016;5(1):210.
12. Modesti PA, Reboldi G, Cappuccio FP, Agyemang C, Remuzzi G, Rapi S, et al. Panethnic Differences in Blood Pressure in Europe: A Systematic Review and Meta-Analysis. *PLoS ONE* 2016;11(1):e0147601.
13. Nyaga VN, Arbyn M, Aerts M. Metaprop: a Stata command to perform meta-analysis of binomial data. *Arch Public Health* 2014;72(1):39.
14. Freeman MF, Tukey JW. Transformations related to the angular and the square root. *Ann Math Stat* 1950;21:607-11.
15. Aguiar LB, Baccaro LF, de Souza Santos Machado V, Pinto-Neto AM, Costa-Paiva L. Disability and multimorbidity in women older than 50 years: a population-based household survey. *Menopause (New York, NY)* 2015;22(6):660-6.

For peer review only

16. Amaral TLM, Amaral CdA, Lima NSd, Herculano PV, Prado PRd, Monteiro GTR. Multimorbidity, depression and quality of life among elderly people assisted in the Family Health Strategy in Senador Guiomard, Acre, Brazil. *Multimorbidade, depressao e qualidade de vida em idosos atendidos pela Estrategia de Saude da Familia em Senador Guiomard, Acre, Brasil* 2018;23(9):3077-84.
17. Andrade LH, Benseñor IM, Viana MC, Andreoni S, Wang YP. Clustering of psychiatric and somatic illnesses in the general population: multimorbidity and socioeconomic correlates. *Braz j med biol res* 2010;43(5):483-91.
18. Araujo MEA, Silva MT, Galvao TF, Nunes BP, Pereira MG. Prevalence and patterns of multimorbidity in Amazon Region of Brazil and associated determinants: a cross-sectional study. *BMJ open* 2018;8(11):e023398.
19. Boing AF, Melo GR, Boing AC, Moretti-Pires RO, Peres KG, Peres MA. Associação entre depressão e doenças crônicas: um estudo populacional. *Rev saúde pública* 2012;46(4):617-23.
20. Costa CdS, Flores TR, Wendt A, Neves RG, Tomasi E, Cesar JA, et al. Inequalities in multimorbidity among elderly: a population-based study in a city in Southern Brazil. *Cad Saúde Pública (Online)* 2018;34(11):e00040718-e.
21. da Silva Almeida IL, dos Santos SR, Morbeck de Queiroz B, de Freitas Mussi RF. Lifestyle, morbidity and multimorbidity in adult Quilombolas. *ABCS health sci* 2020;45:1325.
22. de Souza Santos Machado V, Valadares ALR, da Costa-Paiva LS, Moraes SS, Pinto-Neto AM. Multimorbidity and associated factors in Brazilian women aged 40 to 65 years: a population-based study. *Menopause (New York, NY)* 2012;19(5):569-75.
23. Macinko J, Andrade FCD, Nunes BP, Guanais FC. Primary care and multimorbidity in six Latin American and Caribbean countries. *Rev panam salud pública* 2019;43:e8-e.
24. Montes MC, Bortolotto CC, Tomasi E, Gonzalez MC, Barbosa-Silva TG, Domingues MR, et al. Strength and multimorbidity among community-dwelling elderly from southern Brazil. *Nutrition* 2020;71.
25. Nunes BP, Batista SRR, Andrade FBd, Souza Junior PRBd, Lima-Costa MF, Facchini LA. Multimorbidity: The Brazilian Longitudinal Study of Aging (ELSI-Brazil). *Revista de saude publica* 2018;52Suppl 2(Suppl 2):10s.

For peer review only

26. Nunes BP, Camargo-Figuera FA, Guttier M, de Oliveira PD, Munhoz TN, Matijasevich A, et al. Multimorbidity in adults from a southern Brazilian city: occurrence and patterns. *International journal of public health* 2016;61(9):1013-20.
27. Nunes BP, Chiavegatto Filho ADP, Pati S, Cruz Teixeira DS, Flores TR, Camargo-Figuera FA, et al. Contextual and individual inequalities of multimorbidity in Brazilian adults: a cross-sectional national-based study. *BMJ open* 2017;7(6):e015885.
28. Nunes BP, Soares MU, Wachs LS, Volz PM, Saes MdO, Duro SMS, et al. Hospitalization in older adults: association with multimorbidity, primary health care and private health plan. *Rev saúde pública* 2017;51:43-.
29. Nunes BP, Thume E, Facchini LA. Multimorbidity in older adults: magnitude and challenges for the Brazilian health system. *BMC public health* 2015;15:1172.
30. Padilha Pereira B, Cardozo Bortolotto C, Tomasi E, Gonzalez MC, Gomes AP, Goncalves H, et al. Food consumption and multimorbidity among non-institutionalized elderly people in Pelotas, 2014: a cross-sectional study. *Epidemiol serv saúde* 2020;29(3):e2019050.
31. Petarli GB, Cattafesta M, Sant'Anna MM, Bezerra OMdPA, Zandonade E, Salaroli LB. Multimorbidity and complex multimorbidity in Brazilian rural workers. *PLoS ONE* 2019;14(11):e0225416.
32. Tavares DMDS, Pelizaro PB, Pegorari MS, Paiva MMd, Marchiori GF. Prevalence of self-reported morbidities and associated factors among community-dwelling elderly in Uberaba, Minas Gerais, Brazil. *Prevalencia de morbidades autorreferidas e fatores associados entre idosos comunitarios de Uberaba, Minas Gerais, Brasil* 2019;24(9):3305-13.
33. Valadares ALR, Lui-Filho JF, Costa-Paiva L, Pinto-Neto AM. Middle-aged female sexual dysfunction and multimorbidity: a population-based study. *Menopause (New York, NY)* 2016;23(3):304-10.
34. Wang Y-P, Nunes BP, Coelho BM, Santana GL, do Nascimento CF, Viana MC, et al. Multilevel Analysis of the Patterns of Physical-Mental Multimorbidity in General Population of Sao Paulo Metropolitan Area, Brazil. *Scientific reports* 2019;9(1):2390.
35. Agrawal S, Agrawal PK. Association Between Body Mass index and Prevalence of Multimorbidity in Low-and Middle-income Countries: A Cross-Sectional Study. *International journal of medicine and public health* 2016;6(2):73-83.

For peer review only

36. Bao J, Chua K-C, Prina M, Prince M. Multimorbidity and care dependence in older adults: a longitudinal analysis of findings from the 10/66 study. *BMC public health* 2019;19(1):585.
37. Bustos-Vazquez E, Fernandez-Nino JA, Astudillo-Garcia CI. Self-rated health, multimorbidity and depression in Mexican older adults: Proposal and evaluation of a simple conceptual model. *Biomedica : revista del Instituto Nacional de Salud* 2017;37(0):92-103.
38. Miranda JJ, Bernabe-Ortiz A, Gilman RH, Smeeth L, Malaga G, Wise RA, et al. Multimorbidity at sea level and high-altitude urban and rural settings: The CRONICAS Cohort Study. *Journal of Comorbiddity* 2019;9:1-10.
39. Taype-Rondan A, Abbs ES, Lazo-Porras M, Checkley W, Gilman RH, Smeeth L, et al. Association between chronic conditions and health-related quality of life: differences by level of urbanization in Peru. *Quality of life research : an international journal of quality of life aspects of treatment, care and rehabilitation* 2017;26(12):3439-47.
40. Camargo-Casas S, Suarez-Monsalve S, Zepeda MUP, Garcia-Pena C, Cano-Gutierrez CA. [Multimorbidity, Depressive Symptoms, and Self-Reported Health in Older Adults: a Secondary Analysis of the Sabe Bogota Study]. *Revista de investigacion clinica; organo del Hospital de Enfermedades de la Nutricion* 2018;70(4):192-7.
41. Olivares DEV, Chambi FRV, Chani EMM, Craig WJ, Pacheco SOS, Pacheco FJ. Risk Factors for Chronic Diseases and Multimorbidity in a Primary Care Context of Central Argentina: A Web-Based Interactive and Cross-Sectional Study. *International journal of environmental research and public health* 2017;14(3).
42. Cavalcanti G, Doring M, Portella MR, Bortoluzzi EC, Mascarelo A, Dellani MP. Multimorbidity associated with polypharmacy and negative self-perception of health. *Rev bras geriatr gerontol (Online)* 2017;20(5):634-42.
43. Silva H, Hernandez-Hernandez R, Vinueza R, Velasco M, Boissonnet CP, Escobedo J, et al. Cardiovascular risk awareness, treatment, and control in urban Latin America. *Am J Ther* 2010;17(2):159-66.
44. Lerner AG, Bernabe-Ortiz A, Gilman RH, Smeeth L, Miranda JJ. The "rule of halves" does not apply in Peru: awareness, treatment, and control of hypertension and diabetes in rural, urban, and rural-to-urban migrants. *Crit Pathw Cardiol* 2013;12(2):53-8.
45. Whitty CJM, Watt FM. Map clusters of diseases to tackle multimorbidity. *Nature* 2020;579(7800):494-6.

For peer review only

46. National Institute for Health and Care Excellence (NICE). Multimorbidity: clinical assessment and management: NICE guideline [NG56]. NICE; 2016 [updated 2016; cited 2021 January 30]; Available from: https://www.nice.org.uk/guidance/ng56.

For peer review only

TABLES AND FIGURES

Figure 1: Flowchart of studies included in the systematic review

For peer review only

Figure 2: Forest plot of the pooled prevalence of multimorbidity defined as ≥2 chronic conditions

For peer review only

Table 1: Characteristics of the studies included in the systematic review

First author	Country	Study design	Data collected in	Sample size	Definition of multimorbidity	Age range	Age mean	% women	% urban
Andrade [17]	Brazil	Cross-sectional	1994-1995	1,464	≥2 (self-reported)	18+	--	57.4%	100%
Boing [19]	Brazil	Cross-sectional	2009-2010	1,720	≥2 (self-reported)	20 - 59	--	55.6%	100%
De Souza Santos Machado [22]	Brazil	Cross-sectional	2005	377	≥2 (self-reported)	40 - 65	--	100%	100%
Aguiar [15]	Brazil	Cross-sectional	2011	622	≥2 (self-reported)	50+	64.1	100%	100%
Nunes [29]	Brazil	Cross-sectional	2008	1,593	≥2 and ≥3 (self-reported)	60+	--	62.8%	100%
Agrawal [35]	Mexico	Cross-sectional	2007-2010	2,725	≥2 (self-reported)	18+	63.1	61.8%	73.7%
Nunes [26]	Brazil	Cross-sectional	2012	2,927	≥2 and ≥3 (self-reported)	20+	45.7	58.9%	100%
Valadares [33]	Brazil	Cross-sectional	2012-2013	736	≥2 (self-reported)	45 - 60	52.5	100%	100%
Bustos-Vazquez [37]	Mexico	Cross-sectional	2012	7,967	≥2 (self-reported)	60+	69.3	53.4%	--
Cavalcanti [42]	Brazil	Cross-sectional	2010-2011	676	≥2 (self-reported)	60+	70.0	54.6%	69.4%
Nunes [27]	Brazil	Cross-sectional	2013	60,202	≥2 and ≥3 (self-reported)	18+	43.7	55.1%	86.5%
Nunes [28]	Brazil	Cross-sectional	2008	1,593	≥2 and ≥3 (self-reported)	60+	--	62.8%	--
Olivares [41]	Argentina	Cross-sectional	2014-2015	1,044	≥2 (self-reported)	18+	43.0	65%	--

For peer review only

Taype-Rondan [39]	Peru	Longitudinal	2013-2014	2,433	≥2 (self-reported and measured)	35+	57.2	51.3%	51.3%
Amaral [16]	Brazil	Cross-sectional	2010	264	≥2 (self-reported)	60+	--	61%	100%
Araujo [18]	Brazil	Cross-sectional	2015	4,001	≥2 and ≥3 (self-reported)	18+	--	52.7%	86.8%
Camargo-Casas [40]	Colombia	Cross-sectional	2012	2,000	≥2 (self-reported)	60+	71.1	63.4%	--
Costa [20]	Brazil	Cross-sectional	2014	1,451	≥2 and ≥3 (self-reported and measured)	60+	--	63%	--
Nunes [25]	Brazil	Longitudinal	2015-2016	9,412	≥2 and ≥3 (self-reported)	50+	62.9	54%	84.7%
Bao [36]	Cuba	Longitudinal	2003-2007	2,944	≥2 (self-reported and measured)	65+	73.9	64%	--
	Dominican Republic			2,011			74.5	65%	--
	Puerto Rico			2,009			75.2	67%	--
	Venezuela			1,965			71.5	63%	--
	Peru			1,933			74.2	60%	--
	Mexico			2,003			73.7	64%	--
Macinko [23]	Brazil	Cross-sectional	2013-2014	1,486	≥2 (self-reported)	18+	--	51.1%	--
	Colombia			1,485			--	51.1%	--
	El Salvador			1,460			--	52.8%	--
	Jamaica			1,480			--	52.6%	--
	Mexico			1,492			--	51.4%	--
	Panama			1,475			--	51.6%	--
Miranda [38]	Peru	Longitudinal	2010-2012	2,890	≥2 (self-reported and measured)	35+	55.2	51%	50.9%
Petarli [31]	Brazil	Cross-sectional	2016-2017	790	≥2 and ≥3 (self-reported)	18 - 59	--	47.7%	--
Tavares [32]	Brazil	Cross-sectional	2012	1,691	≥2 (self-reported)	60+	72.5	63.7%	100%

For peer review only

Wang [34]	Brazil	Cross-sectional	2005-2007	2,713	≥2 (self-reported)	18 - 64	--	52,4%	--
Montes [24]	Brazil	Cross-sectional	2014	1,336	≥5 (self-reported)	60+	--	63.1%	--
Padilha Pereira [30]	Brazil	Cross-sectional	2014	1,426	≥5 (self-reported)	60+	--	63%	--
Da Silva Almeida [21]	Brazil	Cross-sectional	2016	850	≥2 (self-reported and measured)	18+	--	61.2%	--

For peer review only

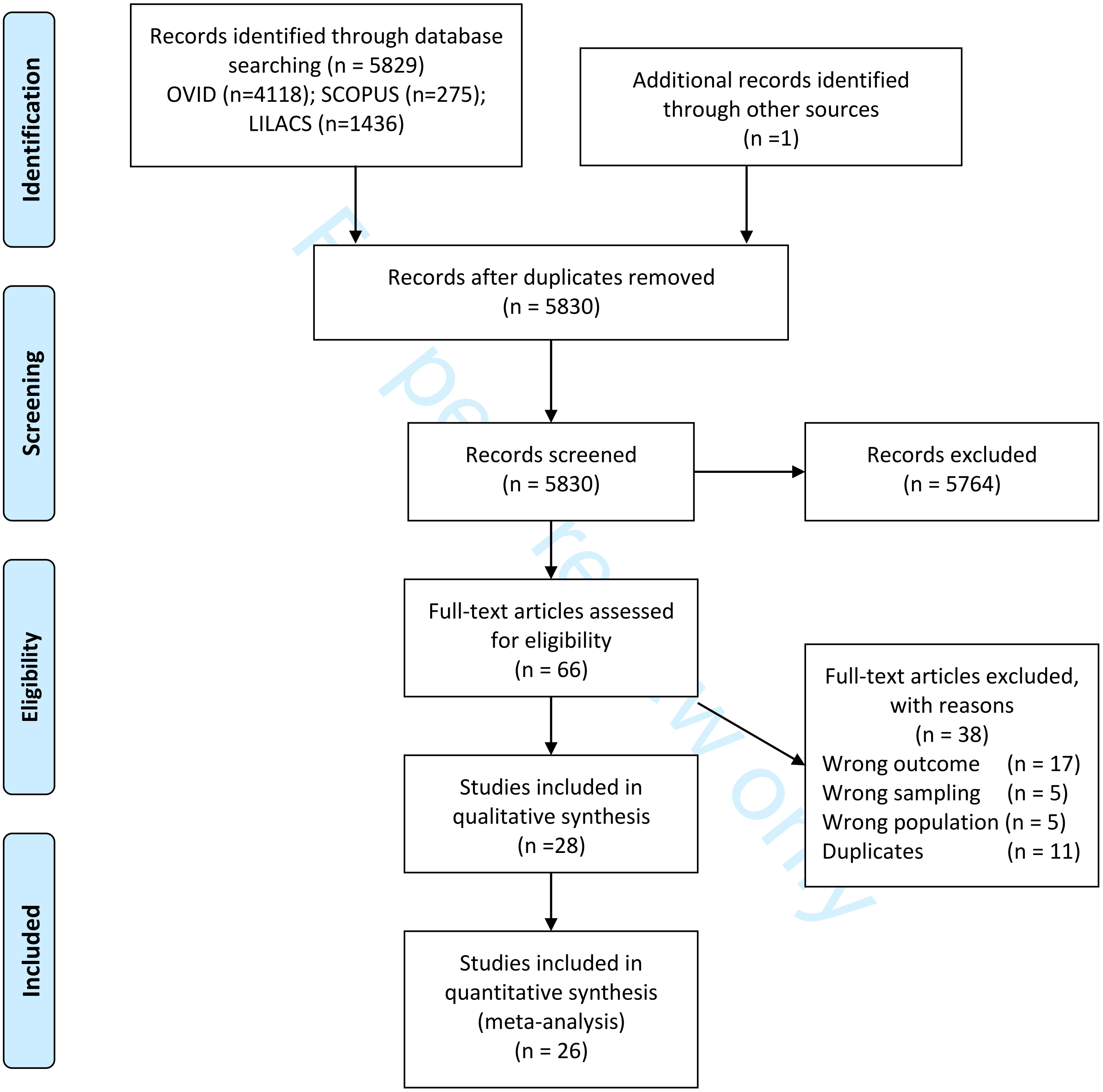
Identification
Screening
Eligibility
Included
Records identified through database searching (n = 5829)
OVID (n=4118); SCOPUS (n=275);
LILACS (n=1436)
Additional records identified through other sources
(n =1)
Records after duplicates removed
(n = 5830)
Records screened
(n = 5830)
Records excluded
(n = 5764)
Full-text articles assessed for eligibility
(n = 66)
Full-text articles excluded, with reasons
(n = 38)
Wrong outcome (n = 17)
Wrong sampling (n = 5)
Wrong population (n = 5)
Duplicates (n = 11)
Studies included in qualitative synthesis
(n =28)
Studies included in quantitative synthesis (meta-analysis)
(n = 26)

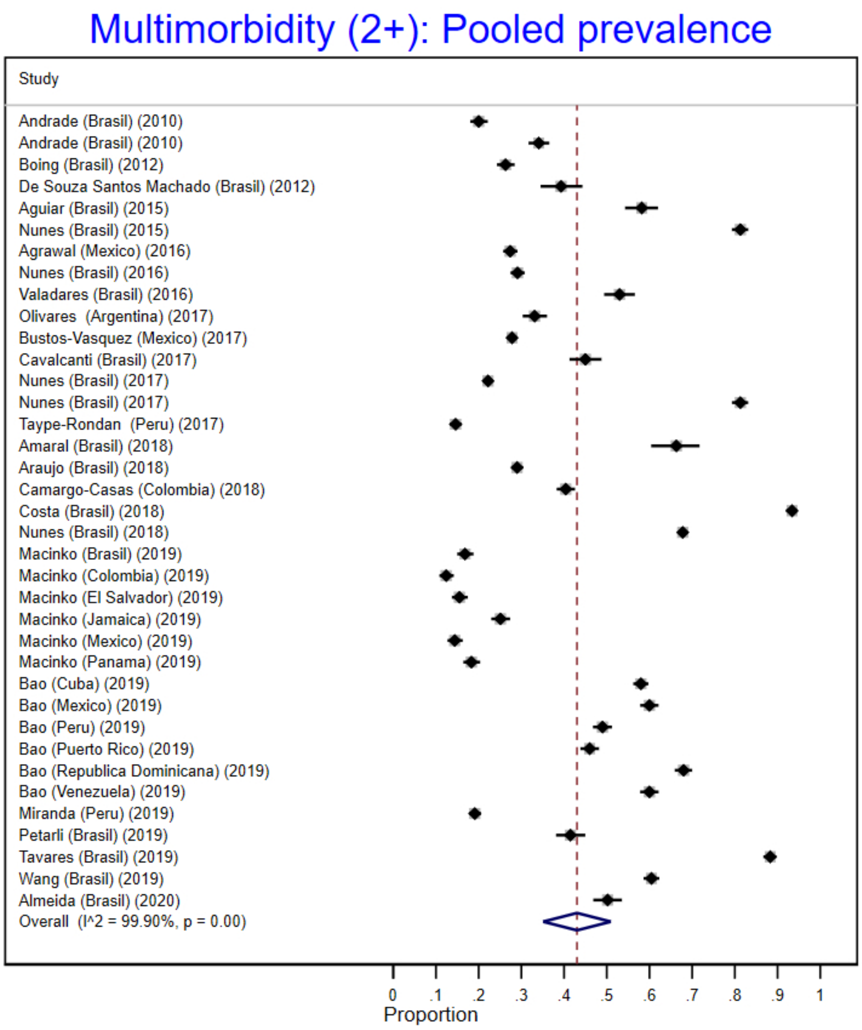

Figure 2: Forest plot of the pooled prevalence of multimorbidity defined as ≥2 chronic conditions

291x319mm (72 x 72 DPI)

SUPPLEMENTAL MATERIAL

E-Figure 1: Forest plot with pooled estimates of multimorbidity among males

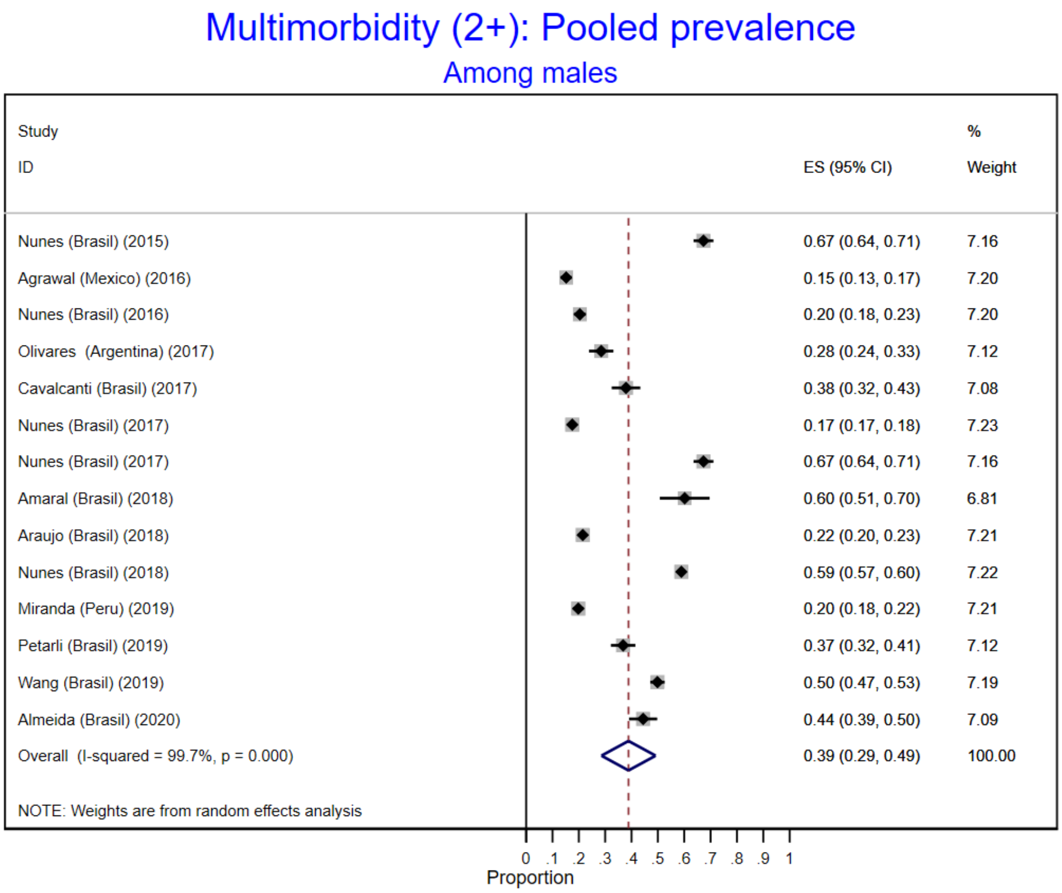

E-Figure 2: Forest plot with pooled estimates of multimorbidity among females

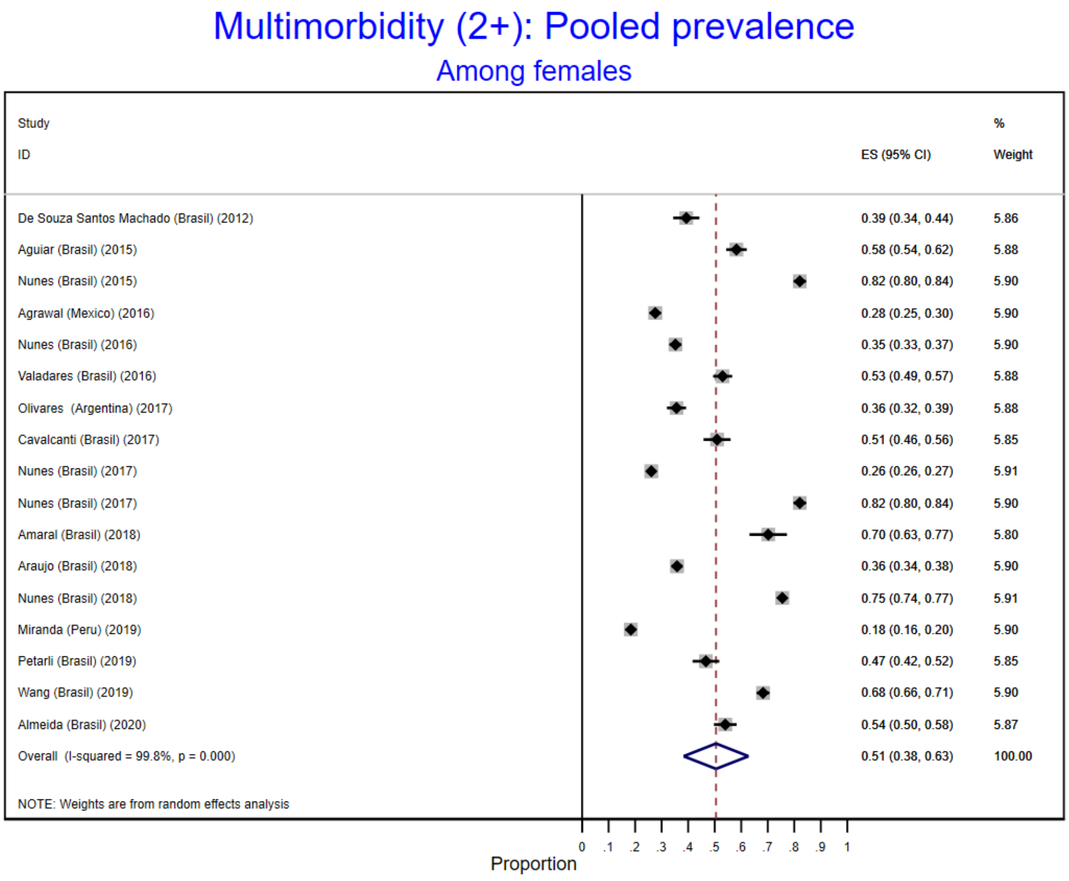

E-Figure 3: Forest plot with pooled estimates of multimorbidity among urban dwellers

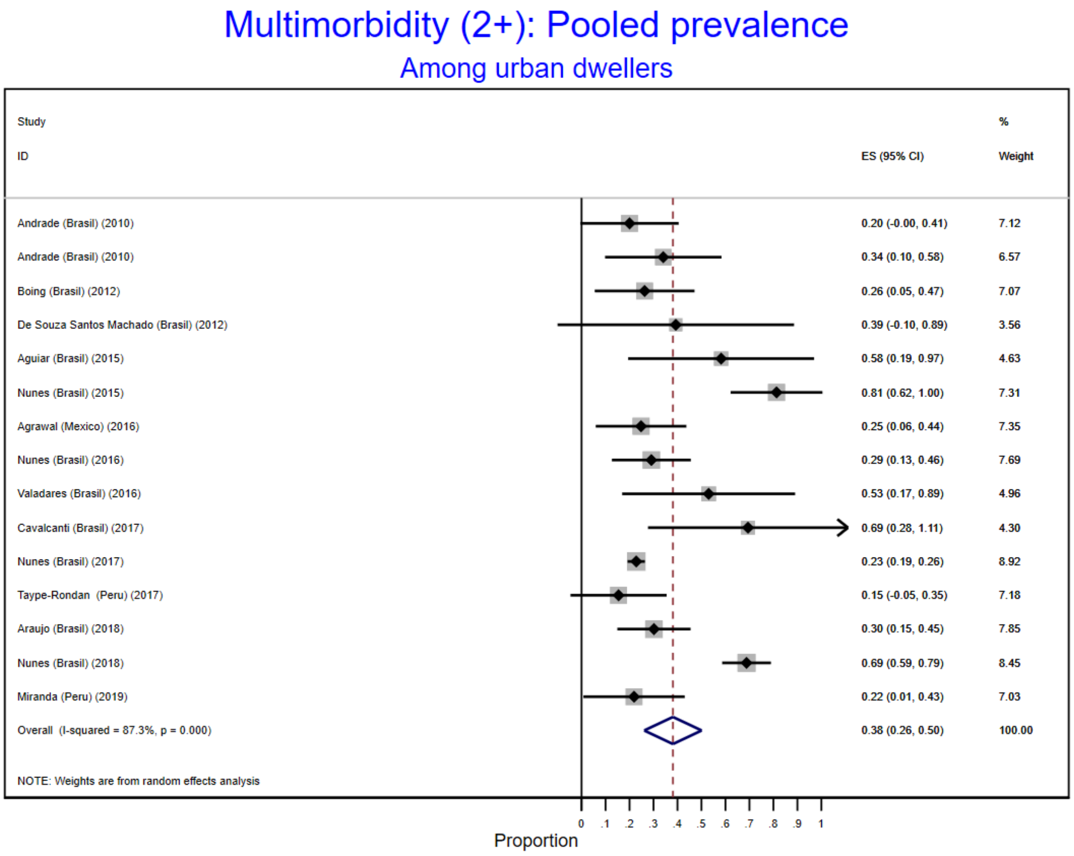

E-Figure 4: Forest plot with pooled estimates of multimorbidity among rural dwellers

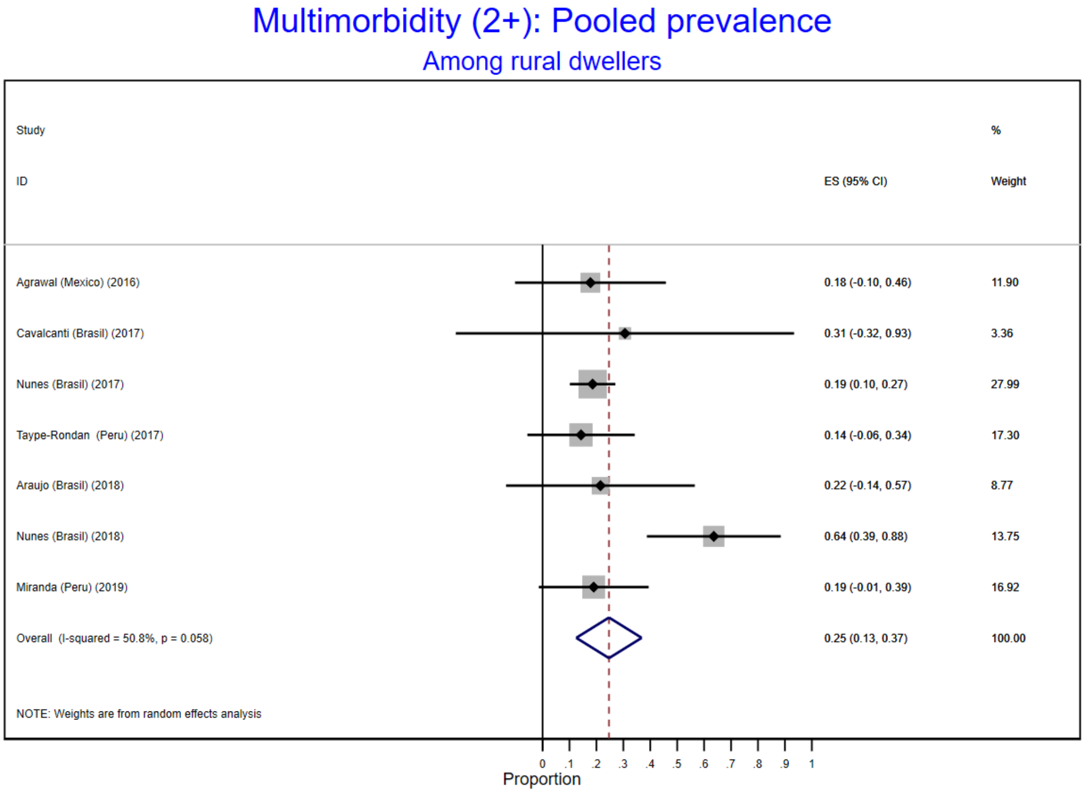

E-Table 1: Search terms and strategy using OVID (Embase, Medline and Global Health)

1	multimorbidit*.mp
2	multi-morbidit*.mp
3	(multi adj morbidit*).mp
4	multiple morbidit*.mp
5	(co adj morbidit*).mp
6	comorbidit*.mp
7	“multiple chronic conditions”.mp
8	“multiple chronic illnesses”.mp
9	“multiple chronic diseases”.mp
10	(poly adj morbidit*).mp
11	polymorbidit*.mp
12	poly-morbidit*.mp
13	1 or 2 or 3 or 4 or 5 or 6 or 7 or 8 or 9 or 10 or 11 or 12
14	(("Antigua and Barbuda") or ("Argentina") or ("Bahamas") or ("Barbados") or ("Belize") or ("Bolivia") or ("Brazil") or ("United States Virgin Islands") or ("British Virgin Islands") or ("Chile") or ("Colombia") or ("Costa Rica") or ("Cuba") or ("Dominica") or ("Dominican Republic") or ("Ecuador") or ("El Salvador") or ("Grenada") or ("Guatemala") or ("Guyana") or ("Haiti") or ("Honduras") or ("Jamaica") or ("Mexico") or ("Nicaragua") or ("Panama") or ("Paraguay") or ("Peru") or ("Puerto Rico") or ("Saint Kitts and Nevis") or ("Saint Lucia") or ("Saint Vincent and the Grenadines") or ("Suriname") or ("Trinidad and Tobago") or ("West Indies") or ("Uruguay") or ("Venezuela") or ("Latin America") or latin amer$ or ("South America") or south amer$ or ("Central America") or central amer$ or ("Caribbean Region"))
15	13 and 14
16	exp animals/ not humans.sh.
17	15 not 16
18	remove duplicates from 17

For peer review only

E-Table 2: Search terms and strategy using Scopus

(TITLE-ABS-KEY(multimorbidity$) OR TITLE-ABS-KEY(multi-morbidit$) OR TITLE-ABS-KEY(multi-morbidit$) OR TITLE-ABS-KEY(multi Pre/1 morbidit$) OR TITLE-ABS-KEY(multiple morbidit$) OR TITLE-ABS-KEY(co Pre/1 morbidit$) OR TITLE-ABS-KEY(comorbidit$) OR TITLE-ABS-KEY(multiple chronic conditions) OR TITLE-ABS-KEY(multiple chronic illnesses) OR TITLE-ABS-KEY(multiple chronic diseases) OR TITLE-ABS-KEY(poly Pre/1 morbidit$) OR TITLE-ABS-KEY(polymorbidit$) OR TITLE-ABS-KEY(poly-morbidit$)) AND (TITLE-ABS-KEY("Antigua and Barbuda") or TITLE-ABS-KEY("Argentina") or TITLE-ABS-KEY("Bahamas") or TITLE-ABS-KEY("Barbados") or TITLE-ABS-KEY("Belize") or TITLE-ABS-KEY("Bolivia") or TITLE-ABS-KEY("Brazil") or TITLE-ABS-KEY("United States Virgin Islands") or TITLE-ABS-KEY("British Virgin Islands") or TITLE-ABS-KEY("Chile") or TITLE-ABS-KEY("Colombia") or TITLE-ABS-KEY("Costa Rica") or TITLE-ABS-KEY("Cuba") or TITLE-ABS-KEY("Dominica") or TITLE-ABS-KEY("Dominican Republic") or TITLE-ABS-KEY("Ecuador") or TITLE-ABS-KEY("El Salvador") or TITLE-ABS-KEY("Grenada") or TITLE-ABS-KEY("Guatemala") or TITLE-ABS-KEY("Guyana") or TITLE-ABS-KEY("Haiti") or TITLE-ABS-KEY("Honduras") or TITLE-ABS-KEY("Jamaica") or TITLE-ABS-KEY("Mexico") or TITLE-ABS-KEY("Nicaragua") or TITLE-ABS-KEY("Panama") or TITLE-ABS-KEY("Paraguay") or TITLE-ABS-KEY("Peru") or TITLE-ABS-KEY("Puerto Rico") or TITLE-ABS-KEY("Saint Kitts and Nevis") or TITLE-ABS-KEY("Saint Lucia") or TITLE-ABS-KEY("Saint Vincent and the Grenadines") or TITLE-ABS-KEY("Suriname") or TITLE-ABS-KEY("Trinidad and Tobago") or TITLE-ABS-KEY("West Indies") or TITLE-ABS-KEY("Uruguay") or TITLE-ABS-KEY("Venezuela") or TITLE-ABS-KEY("Latin America") or TITLE-ABS-KEY(latin amer$) or TITLE-ABS-KEY("South America") or TITLE-ABS-KEY(south amer$) or TITLE-ABS-KEY("Central America") or TITLE-ABS-KEY(central amer$) or TITLE-ABS-KEY("Caribbean Region")) AND NOT DBCOLL(medl)

E-Table 3: Search terms and strategy using LILACS

((multi-morbilidad) OR (multimorbilidad) OR (co-morbilidad) OR (comorbilidad) OR (multiple morbilidad) OR (multiples condiciones cronicas) OR (multiples enfermedades cronicas) OR (multiples patologias cronicas) OR (poli-morbilidad) OR (polimorbilidad)) AND (("Antigua y Barbuda") OR ("Argentina") OR ("Aruba") OR ("Bahamas") OR ("Barbados") OR ("Belice") OR ("Bolivia") OR ("Brasil") OR ("Islas Vírgenes de los Estados Unidos") OR ("Islas Vírgenes Británicas") OR ("Islas Caimán") OR ("Chile") OR ("Colombia") OR ("Costa Rica") OR ("Cuba") OR ("Curazao") OR ("Dominica") OR ("Republica Dominicana") OR ("Ecuador") OR ("El Salvador") OR ("Granada") OR ("Guatemala") OR ("Guyana") OR ("Haití") OR ("Honduras") OR ("Jamaica") OR ("México") OR ("Nicaragua") OR ("Panamá") OR ("Paraguay") OR ("Perú") OR ("Puerto Rico") OR ("San Cristóbal y Nieves ") OR ("Santa Lucía") OR ("San Vicente y las Granadinas ") OR ("Surinam") OR ("Trinidad y Tobago") OR ("Turcas y Caicos ") OR ("Uruguay") OR ("Venezuela") OR ("América Latina") OR ("Latinoamérica") OR ("América del Sur") OR ("Sudamérica") OR ("Suramérica") OR ("América Central") OR ("Centroamérica") OR ("América del Centro") OR ("Caribe"))

For peer review only - http://bmjopen.bmj.com/site/about/guidelines.xhtml

E-Table 4: PRISMA Checklist

Section/topic	#	Checklist item	Reported on page #
TITLE			
Title	1	Identify the report as a systematic review, meta-analysis, or both.	01
ABSTRACT			
Structured summary	2	Provide a structured summary including, as applicable: background; objectives; data sources; study eligibility criteria, participants, and interventions; study appraisal and synthesis methods; results; limitations; conclusions and implications of key findings; systematic review registration number.	02
INTRODUCTION			
Rationale	3	Describe the rationale for the review in the context of what is already known.	03
Objectives	4	Provide an explicit statement of questions being addressed with reference to participants, interventions, comparisons, outcomes, and study design (PICOS).	03
METHODS			
Protocol and registration	5	Indicate if a review protocol exists, if and where it can be accessed (e.g., Web address), and, if available, provide registration information including registration number.	03-04
Eligibility criteria	6	Specify study characteristics (e.g., PICOS, length of follow-up) and report characteristics (e.g., years considered, language, publication status) used as criteria for eligibility, giving rationale.	04
Information sources	7	Describe all information sources (e.g., databases with dates of coverage, contact with study authors to identify additional studies) in the search and date last searched.	04
Search	8	Present full electronic search strategy for at least one database, including any limits used, such that it could be repeated.	Suppl material e-Tables 1-3
Study selection	9	State the process for selecting studies (i.e., screening, eligibility, included in systematic review, and, if applicable, included in the meta-analysis).	04
Data collection process	10	Describe method of data extraction from reports (e.g., piloted forms, independently, in duplicate) and any processes for obtaining and confirming data from investigators.	04

For peer review only

Data items	11	List and define all variables for which data were sought (e.g., PICOS, funding sources) and any assumptions and simplifications made.	04
Risk of bias in individual studies	12	Describe methods used for assessing risk of bias of individual studies (including specification of whether this was done at the study or outcome level), and how this information is to be used in any data synthesis.	04-05
Summary measures	13	State the principal summary measures (e.g., risk ratio, difference in means).	05
Synthesis of results	14	Describe the methods of handling data and combining results of studies, if done, including measures of consistency (e.g., I^2) for each meta-analysis.	05
Risk of bias across studies	15	Specify any assessment of risk of bias that may affect the cumulative evidence (e.g., publication bias, selective reporting within studies).	NA
Additional analyses	16	Describe methods of additional analyses (e.g., sensitivity or subgroup analyses, meta-regression), if done, indicating which were pre-specified.	05
RESULTS			
Study selection	17	Give numbers of studies screened, assessed for eligibility, and included in the review, with reasons for exclusions at each stage, ideally with a flow diagram.	05 and Figure 1
Study characteristics	18	For each study, present characteristics for which data were extracted (e.g., study size, PICOS, follow-up period) and provide the citations.	05 and Table 1
Risk of bias within studies	19	Present data on risk of bias of each study and, if available, any outcome level assessment (see item 12).	Suppl Material eTable 5
Results of individual studies	20	For all outcomes considered (benefits or harms), present, for each study: (a) simple summary data for each intervention group (b) effect estimates and confidence intervals, ideally with a forest plot.	06 and Suppl Material e-Table 6
Synthesis of results	21	Present results of each meta-analysis done, including confidence intervals and measures of consistency.	06 and Tables
Risk of bias across studies	22	Present results of any assessment of risk of bias across studies (see Item 15).	NA
Additional analysis	23	Give results of additional analyses, if done (e.g., sensitivity or subgroup analyses, meta-regression [see Item 16]).	06 and Suppl Material e-Figure 1-4

For peer review only

DISCUSSION			
Summary of evidence	24	Summarize the main findings including the strength of evidence for each main outcome; consider their relevance to key groups (e.g., healthcare providers, users, and policy makers).	06
Limitations	25	Discuss limitations at study and outcome level (e.g., risk of bias), and at review-level (e.g., incomplete retrieval of identified research, reporting bias).	07
Conclusions	26	Provide a general interpretation of the results in the context of other evidence, and implications for future research.	07-08
FUNDING			
Funding	27	Describe sources of funding for the systematic review and other support (e.g., supply of data); role of funders for the systematic review.	01

For peer review only

E-Table 5: Coverage and conditions included in the definition of multimorbidity

First author	Study coverage	Number of conditions	Conditions included in the definition of multimorbidity
Andrade [1]	Sub-national	8	Back pain, hypertension, asthma, headache, heart problems, diabetes, stroke, and cancer
Andrade [1]	Sub-national	15	Cognitive impairment, non-affective psychosis, panic attacks, generalized anxiety, any phobias, obsessive-compulsive disorders, depression, dysthymia, bipolar disorder, bulimia, alcohol use disorder, substance use disorder, nicotine dependence, somatoform disorder, and dissociative disorder
Boing [2]	Sub-national	11	Problems in the spine or on the back, arthritis or rheumatism, cancer, diabetes, bronchitis or asthma, hypertension, heart disease, chronic renal failure, tuberculosis, tendinitis or tenosynovitis, and cirrhosis
De Souza Santos Machado [3]	Sub-national	5	Depression, hypertension, diabetes mellitus, urinary incontinence, and insomnia
Aguiar [4]	Sub-national	10	Hypertension, myocardial infarction, stroke, diabetes, pulmonary emphysema, bronchitis/asthma, arthrosis, osteoporosis, cataract, and urinary incontinence
Nunes [5]	Sub-national	17	High blood pressure, diabetes, lung problem, heart problem, stroke, rheumatism, arthritis or arthrosis, disease in spinal column (any problem reported), cancer, kidney problem, cognitive impairment, depression, urinary incontinence, amputation in any part of the body, eyesight problem, hearing problem, problem chewing food, and falls
Agrawal [6]	National	9	Angina pectoris, arthritis, asthma, chronic lung disease, diabetes, hypertension, stroke, depression, and low visual acuity
Nunes [7]	Sub-national	11	High blood pressure, depression, heart disease, hypercholesterolemia, diabetes, bronchitis, asthma/wheezy bronchitis, hypothyroidism, osteoporosis, joint problems, and emphysema
Valadares [8]	Sub-national	11	Hypertension, diabetes mellitus, dyslipidemia, myocardial infarction, stroke, deep venous thrombosis or pulmonary embolus, asthma or bronchitis, chronic rheumatic diseases, depression, anxiety, and cancer
Bustos-Vazquez [9]	National	7	Cancer, systemic arterial hypertension, diabetes, depression, cerebral infarction, hypercholesterolemia, and cardiovascular diseases
Cavalcanti [10]	Sub-national	8	Systemic arterial hypertension, diabetes mellitus, rheumatism, pulmonary problems (asthma, bronchitis and pulmonary emphysema), stroke/cerebral ischemia, arthritis/osteoporosis, heart problems (coronary disease, angina, congestive disease, others), and depression

For peer review only

Nunes [11]	National	22	High blood pressure, spinal column problem, hypercholesterolemia, depression, diabetes, arthritis/rheumatism, asthma/wheezy bronchitis, cancer, other heart disease, work-related muscle-skeletal disorders, stroke, heart attack, kidney problem, heart failure, bronchitis, angina, emphysema, other lung disease, bipolar disorder, other mental disease, schizophrenia, and obsessive-compulsive disorder
Nunes [12]	Sub-national	17	Systemic arterial hypertension, diabetes mellitus, heart disease, pulmonary problems, stroke, rheumatism/arthritis/osteoarthritis, spine problems, cancer, kidney failure, cognitive deficit, depression, visual problems, urinary incontinence, amputation, auditory problems, problem or difficulty chewing food, and falls
Olivares [13]	Sub-national	9	Hypertension, diabetes, high cholesterol, AMI or stroke, asthma, hypothyroidism, celiac disease, cancer and other chronic diseases (arthritis, osteoarthritis, osteoporosis, depression, chronic allergies, COPD; gastritis, migraine, irritable bowel syndrome, epilepsy, psychosis, multiple sclerosis, psoriasis, gout disease, herniated disc, inflammatory vasculitis)
Taype-Rondan [14]	Sub-national	6	Hypertension, chronic bronchitis, depressive mood, type 2 diabetes, heart disease, and stroke
Amaral [15]	Sub-national	8	Hypertension, diabetes, cardiovascular disorder (poor circulation, cerebrovascular accident (CVA or stroke) and heart problem), musculoskeletal disorder (spinal problem, rheumatism, arthritis/ arthrosis and osteoporosis), insomnia, cataracts, anemia and gastrointestinal/urinary disorders
Araujo [16]	Sub-national	12	Hypertension, diabetes, high cholesterol, heart disease (heart attack, angina, heart failure or other), stroke, asthma or asthmatic bronchitis, arthritis or rheumatism, depression, pulmonary disease (pulmonary emphysema, chronic bronchitis or chronic obstructive pulmonary disease), cancer, chronic kidney disease, and chronic spinal problems (chronic back or neck pain, low back pain, sciatic pain, vertebral or disc problems)
Camargo-Casas [17]	Sub-national	12	Hypertension, diabetes, cancer, chronic obstructive pulmonary disease, heart attack, heart failure, stroke, arthritis or osteoarthritis, osteoporosis, gastroesophageal reflux disease, gastritis, and ulcer
Costa [18]	Sub-national	29	High blood pressure, diabetes, heart problem, heart failure, asthma, bronchitis, emphysema, arthritis, Parkinson's disease, kidney failure, hypercholesterolemia, seizure, stomach ulcer, osteoporosis, urinary incontinence, constipation, fecal incontinence, depression, glaucoma, deafness, difficulty swallowing, insomnia, fainting, rhinitis, difficulty speaking, stroke, obesity, mental disorders, and cancer
Nunes [19]	National	19	Systemic arterial hypertension, back problems, high cholesterol, cataract, arthritis or rheumatism, depression, diabetes, osteoporosis heart problem, glaucoma, emphysema

For peer review only

			chronic bronchitis or chronic obstructive pulmonary disease, cerebrovascular accident, cancer, asthma, chronic renal failure, diabetic retinopathy, macular degeneration, Parkinson’s disease, and Alzheimer’s disease
Bao [20]	National	15	Vision, hearing, joints, skin, gastrointestinal, stroke, chronic obstructive pulmonary disease, hypertension, diabetes, ischemic heart disease, heart failure, heart valve disease, depression, anxiety, and dementia
Macinko [21]	National	8	Arthritis, asthma, cancer, depression, diabetes, heart disease, high cholesterol, and hypertension
Miranda [22]	Sub-national	6	Alcohol disorder, asthma, chronic obstructive pulmonary disease, depression, diabetes, and hypertension
Petarli [23]	Sub-national	20	Heart arrhythmia, infarction, stroke, diabetes mellitus, herniated disk, arthrosis, repetitive strain injuries/work-related musculoskeletal disorders, renal disease, Parkinson’s, Alzheimer’s, hepatic cirrhosis, infertility, cancer, asthma, chronic bronchitis, and pulmonary emphysema, arterial hypertension, dyslipidemia, depression and thyroid disorders
Tavares [24]	Sub-national	25	Rheumatism, arthritis/arthrosis, osteoporosis, asthma or bronchitis, tuberculosis, embolism, systemic arterial hypertension, poor circulation (varicose veins), heart problems, diabetes mellitus, obesity, stroke, Parkinson's, urinary incontinence, fecal incontinence, intestinal constipation, sleeping problems, cataract, glaucoma, back problems, kidney problem, accident/trauma sequelae, malignant tumors, benign tumors, and vision problems
Wang [25]	Sub-national	16	Cardiovascular diseases (heart attack, heart disease, and stroke), hypertension, diabetes mellitus, arthritis, chronic musculoskeletal pain, headache or migraine, digestive, respiratory (seasonal allergies, asthma, obstructive pulmonary disease, and emphysema), cancer, neurological diseases (Parkinson disease, epilepsy, and multiple sclerosis), anxiety disorder, mood disorder, heavy drinking, impulse-control disorder, substance use disorder, and premenstrual dysphoria
Montes [26]	Sub-national	28	Systemic arterial hypertension, myocardial infarction, diabetes, heart failure, emphysema, asthma, bronchitis, arthritis, Parkinson's disease, renal failure, seizure, hypercholesterolemia, stomach ulcer, osteoporosis, urinary incontinence, constipation, fecal incontinence, depression, glaucoma, deafness, difficulty swallowing, insomnia, fainting, rhinitis, difficulty speaking, stroke, mental disorders and cancer
Padilha Pereira [27]	Sub-national	28	Arterial hypertension, diabetes, heart problems, heart failure, asthma, bronchitis, emphysema, arthritis, Parkinson’s disease, kidney failure, hypercholesterolemia, convulsions, stomach ulcer, osteoporosis, urinary incontinence, constipation, fecal

For peer review only

			incontinence, depression, glaucoma, deafness, difficulty in swallowing, insomnia, fainting, rhinitis, difficulty in speaking, stroke, mental disorders, and cancer
Da Silva Almeida [28]	Sub-national	5	Hypertension, diabetes, dyslipidemias, obesity, and arthritis or rheumatism

For peer review only

E-Table 6: Risk of bias of included studies

First author	Representativeness	Sample size	Non-respondents	Exposure	Outcome	Statistical test
Andrade [1]	Low	Low	High	NA	High	NA
Boing [2]	Low	Low	Low	NA	High	NA
De Souza Santos Machado [3]	Low	Low	Low	NA	High	NA
Aguiar [4]	Low	Low	Low	NA	High	NA
Nunes [5]	Low	Low	Low	NA	High	NA
Agrawal [6]	Low	Low	High	NA	High	NA
Nunes [7]	Low	Low	Low	NA	High	NA
Valadares [8]	Low	Low	Low	NA	High	NA
Bustos-Vazquez [9]	Low	Low	Low	NA	High	NA
Cavalcanti [10]	Low	Low	Low	NA	High	NA
Nunes [11]	Low	Low	High	NA	High	NA
Nunes [12]	Low	Low	Low	NA	High	NA
Olivares [13]	Low	Low	Low	NA	Low	NA
Taype-Rondan [14]	Low	Low	Low	NA	Low	NA
Amaral [15]	Low	Low	High	NA	High	NA
Araujo [16]	Low	Low	Low	NA	High	NA
Camargo-Casas [17]	Low	Low	Low	NA	High	NA
Costa [18]	Low	Low	Low	NA	High	NA
Nunes [19]	Low	Low	Low	NA	High	NA
Bao [20]	Low	High	Low	NA	Low	NA
Macinko [21]	Low	High	High	NA	High	NA
Miranda [22]	Low	High	Low	NA	Low	NA
Petarli [23]	Low	Low	Low	NA	High	NA
Tavares [24]	Low	Low	Low	NA	High	NA
Wang [25]	Low	Low	Low	NA	High	NA
Montes [26]	Low	Low	Low	NA	High	NA
Padilha Pereira [27]	Low	Low	Low	NA	High	NA
Da Silva Almeida [28]	Low	Low	Low	NA	Low	NA

For peer review only

REFERENCES

1. Andrade LH, Benseñor IM, Viana MC, Andreoni S, Wang YP. Clustering of psychiatric and somatic illnesses in the general population: multimorbidity and socioeconomic correlates. *Braz j med biol res* 2010;43(5):483-91.
2. Boing AF, Melo GR, Boing AC, Moretti-Pires RO, Peres KG, Peres MA. Associação entre depressão e doenças crônicas: um estudo populacional. *Rev saúde pública* 2012;46(4):617-23.
3. de Souza Santos Machado V, Valadares ALR, da Costa-Paiva LS, Moraes SS, Pinto-Neto AM. Multimorbidity and associated factors in Brazilian women aged 40 to 65 years: a population-based study. *Menopause (New York, NY)* 2012;19(5):569-75.
4. Aguiar LB, Baccaro LF, de Souza Santos Machado V, Pinto-Neto AM, Costa-Paiva L. Disability and multimorbidity in women older than 50 years: a population-based household survey. *Menopause (New York, NY)* 2015;22(6):660-6.
5. Nunes BP, Thume E, Facchini LA. Multimorbidity in older adults: magnitude and challenges for the Brazilian health system. *BMC public health* 2015;15:1172.
6. Agrawal S, Agrawal PK. Association Between Body Mass index and Prevalence of Multimorbidity in Low-and Middle-income Countries: A Cross-Sectional Study. *International journal of medicine and public health* 2016;6(2):73-83.
7. Nunes BP, Camargo-Figuera FA, Guttier M, de Oliveira PD, Munhoz TN, Matijasevich A, et al. Multimorbidity in adults from a southern Brazilian city: occurrence and patterns. *International journal of public health* 2016;61(9):1013-20.
8. Valadares ALR, Lui-Filho JF, Costa-Paiva L, Pinto-Neto AM. Middle-aged female sexual dysfunction and multimorbidity: a population-based study. *Menopause (New York, NY)* 2016;23(3):304-10.
9. Bustos-Vazquez E, Fernandez-Nino JA, Astudillo-Garcia CI. Self-rated health, multimorbidity and depression in Mexican older adults: Proposal and evaluation of a simple conceptual model. *Biomedica : revista del Instituto Nacional de Salud* 2017;37(0):92-103.
10. Cavalcanti G, Doring M, Portella MR, Bortoluzzi EC, Mascarelo A, Dellani MP. Multimorbidity associated with polypharmacy and negative self-perception of health. *Rev bras geriatr gerontol (Online)* 2017;20(5):634-42.
11. Nunes BP, Chiavegatto Filho ADP, Pati S, Cruz Teixeira DS, Flores TR, Camargo-Figuera FA, et al. Contextual and individual inequalities of multimorbidity in Brazilian adults: a cross-sectional national-based study. *BMJ open* 2017;7(6):e015885.
12. Nunes BP, Soares MU, Wachs LS, Volz PM, Saes MdO, Duro SMS, et al. Hospitalization in older adults: association with multimorbidity, primary health care and private health plan. *Rev saúde pública* 2017;51:43-.
13. Olivares DEV, Chambi FRV, Chani EMM, Craig WJ, Pacheco SOS, Pacheco FJ. Risk Factors for Chronic Diseases and Multimorbidity in a Primary Care Context of Central Argentina: A Web-Based Interactive and Cross-Sectional Study. *International journal of environmental research and public health* 2017;14(3).
14. Taype-Rondan A, Abbs ES, Lazo-Porras M, Checkley W, Gilman RH, Smeeth L, et al. Association between chronic conditions and health-related quality of life: differences by level of urbanization in Peru. *Quality of life research : an international journal of quality of life aspects of treatment, care and rehabilitation* 2017;26(12):3439-47.

For peer review only

15. Amaral TLM, Amaral CdA, Lima NSd, Herculano PV, Prado PRd, Monteiro GTR. Multimorbidity, depression and quality of life among elderly people assisted in the Family Health Strategy in Senador Guiomard, Acre, Brazil. *Multimorbidade, depressao e qualidade de vida em idosos atendidos pela Estrategia de Saude da Familia em Senador Guiomard, Acre, Brasil* 2018;23(9):3077-84.
16. Araujo MEA, Silva MT, Galvao TF, Nunes BP, Pereira MG. Prevalence and patterns of multimorbidity in Amazon Region of Brazil and associated determinants: a cross-sectional study. *BMJ open* 2018;8(11):e023398.
17. Camargo-Casas S, Suarez-Monsalve S, Zepeda MUP, Garcia-Pena C, Cano-Gutierrez CA. [Multimorbidity, Depressive Symptoms, and Self-Reported Health in Older Adults: a Secondary Analysis of the Sabe Bogota Study]. *Revista de investigacion clinica; organo del Hospital de Enfermedades de la Nutricion* 2018;70(4):192-7.
18. Costa CdS, Flores TR, Wendt A, Neves RG, Tomasi E, Cesar JA, et al. Inequalities in multimorbidity among elderly: a population-based study in a city in Southern Brazil. *Cad Saúde Pública (Online)* 2018;34(11):e00040718-e.
19. Nunes BP, Batista SRR, Andrade FBd, Souza Junior PRBd, Lima-Costa MF, Facchini LA. Multimorbidity: The Brazilian Longitudinal Study of Aging (ELSI-Brazil). *Revista de saude publica* 2018;52Suppl 2(Suppl 2):10s.
20. Bao J, Chua K-C, Prina M, Prince M. Multimorbidity and care dependence in older adults: a longitudinal analysis of findings from the 10/66 study. *BMC public health* 2019;19(1):585.
21. Macinko J, Andrade FCD, Nunes BP, Guanais FC. Primary care and multimorbidity in six Latin American and Caribbean countries. *Rev panam salud pública* 2019;43:e8-e.
22. Miranda JJ, Bernabe-Ortiz A, Gilman RH, Smeeth L, Malaga G, Wise RA, et al. Multimorbidity at sea level and high-altitude urban and rural settings: The CRONICAS Cohort Study. *Journal of Comorbiddity* 2019;9:1-10.
23. Petarli GB, Cattafesta M, Sant'Anna MM, Bezerra OMdPA, Zandonade E, Salaroli LB. Multimorbidity and complex multimorbidity in Brazilian rural workers. *PLoS ONE* 2019;14(11):e0225416.
24. Tavares DMDS, Pelizaro PB, Pegorari MS, Paiva MMd, Marchiori GF. Prevalence of self-reported morbidities and associated factors among community-dwelling elderly in Uberaba, Minas Gerais, Brazil. *Prevalencia de morbidades autorreferidas e fatores associados entre idosos comunitarios de Uberaba, Minas Gerais, Brasil* 2019;24(9):3305-13.
25. Wang Y-P, Nunes BP, Coelho BM, Santana GL, do Nascimento CF, Viana MC, et al. Multilevel Analysis of the Patterns of Physical-Mental Multimorbidity in General Population of Sao Paulo Metropolitan Area, Brazil. *Scientific reports* 2019;9(1):2390.
26. Montes MC, Bortolotto CC, Tomasi E, Gonzalez MC, Barbosa-Silva TG, Domingues MR, et al. Strength and multimorbidity among community-dwelling elderly from southern Brazil. *Nutrition* 2020;71.
27. Padilha Pereira B, Cardozo Bortolotto C, Tomasi E, Gonzalez MC, Gomes AP, Goncalves H, et al. Food consumption and multimorbidity among non-institutionalized elderly people in Pelotas, 2014: a cross-sectional study. *Epidemiol serv saúde* 2020;29(3):e2019050.
28. da Silva Almeida IL, dos Santos SR, Morbeck de Queiroz B, de Freitas Mussi RF. Lifestyle, morbidity and multimorbidity in adult Quilombolas. *ABCS health sci* 2020;45:1325.

For peer review only

BMJ Open

Multimorbidity in Latin America and the Caribbean: A systematic review and meta-analysis

Journal:	*BMJ Open*
Manuscript ID	bmjopen-2021-050409.R2
Article Type:	Original research
Date Submitted by the Author:	07-Jul-2021
Complete List of Authors:	Huaquía-Díaz, Alessandra; Universidad Científica del Sur<br>Chalán-Dávila, Tarick; Universidad Científica del Sur<br>Carrillo-Larco, Rodrigo; Imperial College London, ; CRONICAS Center of Excellence in Chronic Diseases<br>Bernabe-Ortiz, Antonio; Universidad Peruana Cayetano Heredia, CRONICAS Centre of Excellence in Chronic Diseases; Universidad Cientifica del Sur
**Primary Subject Heading**:	Epidemiology
Secondary Subject Heading:	Global health, Public health
Keywords:	EPIDEMIOLOGY, PUBLIC HEALTH, Hypertension < CARDIOLOGY, DIABETES & ENDOCRINOLOGY

SCHOLARONE™
Manuscripts

I, the Submitting Author has the right to grant and does grant on behalf of all authors of the Work (as defined in the below author licence), an exclusive licence and/or a non-exclusive licence for contributions from authors who are: i) UK Crown employees; ii) where BMJ has agreed a CC-BY licence shall apply, and/or iii) in accordance with the terms applicable for US Federal Government officers or employees acting as part of their official duties; on a worldwide, perpetual, irrevocable, royalty-free basis to BMJ Publishing Group Ltd ("BMJ") its licensees and where the relevant Journal is co-owned by BMJ to the co-owners of the Journal, to publish the Work in this journal and any other BMJ products and to exploit all rights, as set out in our licence.

The Submitting Author accepts and understands that any supply made under these terms is made by BMJ to the Submitting Author unless you are acting as an employee on behalf of your employer or a postgraduate student of an affiliated institution which is paying any applicable article publishing charge ("APC") for Open Access articles. Where the Submitting Author wishes to make the Work available on an Open Access basis (and intends to pay the relevant APC), the terms of reuse of such Open Access shall be governed by a Creative Commons licence – details of these licences and which Creative Commons licence will apply to this Work are set out in our licence referred to above.

Other than as permitted in any relevant BMJ Author's Self Archiving Policies, I confirm this Work has not been accepted for publication elsewhere, is not being considered for publication elsewhere and does not duplicate material already published. I confirm all authors consent to publication of this Work and authorise the granting of this licence.

For peer review only

Multimorbidity in Latin America and the Caribbean: A systematic review and meta-analysis

Alessandra M. Huaquía-Diaz, BS[1]; Tarick S. Chalán-Dávila, BS[1]; Rodrigo M. Carrillo-Larco, MD[2,3,4]; Antonio Bernabé-Ortiz, MD, MPH, PhD[1,2,5]

1. Universidad Científica del Sur, Lima, Peru
2. CRONICAS Center of Excellence in Chronic Diseases, Universidad Peruana Cayetano Heredia, Lima, Peru
3. Department of Epidemiology and Biostatistics, School of Public Health, Imperial College London, London, UK
4. Universidad Católica Los Ángeles de Chimbote, Instituto de Investigación, Chimbote, Peru
5. School of Public Health and Administration, Universidad Peruana Cayetano Heredia, Lima, Peru

Corresponding author:

Antonio Bernabe-Ortiz, MD, MPH, PhD

Universidad Científica del Sur, Lima, Peru

Email: Antonio.Bernabe@upch.pe

For peer review only

ABSTRACT

Objective: To estimate the pooled prevalence of multimorbidity (≥2 non-communicable diseases in the same individual) among adults of the general population of LAC.

Design: Systematic review and meta-analysis.

Data sources: Medline, Embase, Global Health, Scopus, and LILACS up to July 1st, 2020.

Eligibility criteria for selecting studies: The outcome was the prevalence of multimorbidity. Reports were selected whether they enrolled adult individuals (age ≥18 years) from the general population.

Data extraction and synthesis: Reviewers extracted relevant data and assessed risk of bias independently. A random-effects meta-analysis was conducted to report pooled prevalence estimates of multimorbidity; pooled estimates by pre-specified sub-groups (e.g., national studies) was also pursued.

Results: From 5,830 results, we selected 28 reports, mostly from Brazil and 16 were based on a nationally representative sample. From the 28 selected reports, 26 were further included in the meta-analysis revealing a pooled multimorbidity prevalence of 43% (95% CI: 35% - 51%; I^2: 99.9%). When only reports with a nationally representative sample were combined, the pooled prevalence was 37% (95% CI: 27%-47%; I^2: 99.9%). When the ascertainment of multimorbidity was based on self-reports alone, the pooled prevalence was 40% (95% CI: 31% - 48%; I^2: 99.9%); this raised to 52% (95% CI: 33% - 70%; I^2: 99.9%) for reports including self-reported and objective diagnosis.

Conclusions: Our results complement and advance those from global efforts by incorporating much more reports from LAC. We revealed a larger presence of multimorbidity in LAC than previously reported.

Key words: Multimorbidity, systematic review, prevalence, Latin American and the Caribbean

For peer review only

STRENGHTS AND LIMITATIONS OF THIS STUDY

- Most of the analyzed data came from Brazin preventing region representativity.
- High heterogeneity was present in the analysis, mainly due to different conditions included to define multimorbidity.
- Participant’s age seems to explain high heterogeneity as age range in studies is very wide.
- A bias due to self-reporting of conditions may underestimate the real burden of multimorbidity in LAC region.

For peer review only

INTRODUCTION

The Academy of Medical Sciences defines multimorbidity as "*the existence of two or more medical chronic conditions in a single individual*" [1]. Subjects with multimorbidity tend to increase healthcare utilization and costs of primary and secondary care services [2]; also, multimorbidity has a subsequent impact on quality of life [3].

In high-income countries, multimorbidity rates are heterogeneous but seem depend on individual's age. Thus, in a cross-sectional study using the dataset of medical practices in Scotland, the prevalence of multimorbidity was 23% using a list of 40 conditions, and was present mainly in older people [4]. Moreover, multimorbidity seems to be increasing in low- and middle-income countries, where data is yet scarce [5]. The increase of life expectancy in Latin American and the Caribbean (LAC) has been associated with greater incidence of noncommunicable conditions (NDC) [6], with the consequent emergence of multimorbidity.

Multimorbidity prevalence has been explored and summarized in some systematic reviews around the world [7, 8] and results ranged from 5% to 98%; nevertheless, their results were mainly informed by data from high-income countries. In a relatively recent systematic review [9], 31 low- and middle-income countries (LMIC) were included with a prevalence of multimorbidity of 30%, compared to an estimate of 38% in high-income countries. However, only nine studies from the LAC region were included. Moreover, pooled estimates by region were not elucidated, preventing to have appropriate indicators of the burden of multimorbidity in this region. A more recent systematic review has reported a pooled prevalence of multimorbidity in LMIC between 3% and 90%, with almost 80% of the studies being from Brazil, China, South Africa, India, Mexico, and Iran [10].

The lack of evidence about multimorbidity may have important consequences for research, public health, and clinical management in LAC region. For example, multimorbidity was not appropriately defined up to 2018; in addition, whether estimates depend upon sex or setting characteristics (i.e., rural vs. urban areas) should be also studied. Moreover, the need of surveillance systems to assess multimorbidity may be elucidated as these estimates have not been estimated in LAC region. Thus, from the public health perspective may not be easy to take appropriate decisions or implement adequate strategies to tackle the problem of multimorbidity.

As a result, we aimed at providing robust evidence about multimorbidity prevalence estimates in LAC region through a systematic review and meta-analysis of population-based surveys. These

For peer review only

results evidence may help to guide interventions and policies so that they can focus on the most pressing frequent multimorbidity phenotypes in LAC.

METHODS

Protocol

This systematic review was registered in PROSPERO under the code CRD42020196177. We aimed to identify the population-based prevalence of multimorbidity in LAC, and to study whether this prevalence varies by multimorbidity definitions, sex, and urban-rural settings.

Eligibility criteria

Reports were selected whether they enrolled adult individuals (age ≥18 years) from the general population. We focused on LAC populations; therefore, we excluded studies with LAC individuals in countries outside the LAC region, and studies with only foreign subjects in LAC nations. Population-based studies were defined as those following a random sampling approach, and such sample was taken from the general population. On the contrary, studies addressing specific populations (e.g., pregnant women), those with individuals with specific conditions (e.g., people with hypertension), or subjects with specific risk factors (e.g., obese or alcohol disorders) were excluded.

The outcome of interest was the prevalence of multimorbidity, defined as the existence of ≥ 2 chronic conditions in the same person [1]. Different definitions of multimorbidity were considered in this review (e.g., ≥2, ≥3, ≥4, or ≥5 conditions) as the current definition (≥2 conditions) is relatively recent. In addition, the presence of chronic conditions could have been measured, self-reported or a combination of these approaches.

Information sources

The search was conducted on January 10, 2020, and then updated on July 1st, 2020. We used Ovid search engine, comprising Medline, Embase and Global Health databases; and in parallel, we also searched Scopus and LILACS. In all of these, searching was carried out without time or language restriction. The search strategy and terms used is detailed in Supplementary Material: e-Table 1-3.

Study selection

Results from each search engine were downloaded and saved in EndNote where duplicates were removed. After that, information was transferred to Rayyan, an open-access online tool for

For peer review only

systematic reviews [11]. Titles and abstracts were reviewed by two researchers in an independent way, and disagreements were solved by a third party. After this screening phase, selected reports were downloaded and independently studied in detail by two researchers, and similarly, discrepancies were solved by a third party. Finally, selected studies were examined again to check for data duplication, i.e., different reports that used the same data (e.g., multiple reports based on the same underlying data). In this case, the paper with more information or the one with the largest sample size was included in the review and meta-analysis.

Data collection

An extraction template form was built by the authors and tested with a random sample of selected studies. After starting data collection, the form was not further modified. This form included study characteristics: study design, country, if it was a nationally representative sample, sample size, year of data collection, age range, age mean, proportion of women, and if it was urban, rural or both. The extraction form also collated the definition of multimorbidity used, self-reported or a combination of self-reported and measured, the number and a list of chronic conditions studies, and prevalence of multimorbidity (overall, by sex, and by rural or urban settings).

Risk of bias of individual studies

Risk of bias of selected studies was evaluated using the Newcastle-Ottawa Quality Assessment Scale adapted for cross-sectional studies as in a previous report [12]. This tool is focused on selection process (representativeness, sample size, and non-respondents), and the assessment of the outcome (independent blind assessment, self-report or not description). The items of this scale were implemented in an Excel spreadsheet and assessed independently by two reviewers; discrepancies were solved by a third party.

Summary measures

Our systematic review followed the Preferred Reporting Items for Systematic Review and Meta-Analysis (PRISMA) toolkit (See Checklist in Supplemental Material: e-Table 4). We presented a qualitative and quantitative summary. The qualitative summary described the characteristics of the study (as listed above), whereas the quantitative summary explored pooled prevalence estimates.

Statistical analyses were performed using Stata version 16 for Windows (StataCorp, College Station, TX, US). The "*metaprop*" command attains a pooled estimate as a weighted average, by fitting a logistic-normal random-effect model without covariates, but random intercepts [13]. After

For peer review only

that, the pooled estimate was calculated using the Freeman-Tukey Arcsine Transformation as suggested in literature [14].

Because the selected studies were different in nature, scope (e.g., national surveys versus community/subnational studies, or urban vs. rural settings) and sample size, we conducted random-effects meta-analysis for comparing estimates in specific subgroups (e.g., national studies only). In addition, stratified meta-analysis (e.g., by sex, and by urban/rural settings) was pursued. Sensitivity analyses were also conducted focused on age by including studies with individuals aged 50 years and over, and also with studies whose data was collected from 2010 and onwards. Besides, as many of the studies were conducted in Brazil, a comparison of pooled estimated between Brazil and other countries together was also pursued.

Finally, meta-regression was also conducted as a high level of heterogeneity was expected. Meta-regression command in STATA investigates if between-study heterogeneity can be explained by one or more of the variables included in the review.

Patient and Public Involvement

Patients or the public were not involved in the design, or conduct, or reporting, or dissemination plans of our research.

Ethical considerations

Not applicable

RESULTS

Selection process

The search strategy yielded 5,830 titles and abstracts after removing duplicates (See Figure 1); of these 66 were studied in detail and 28 reports met the inclusion criteria. For quantitative analyses, 26 reports were included (2 were excluded as the definition of multimorbidity was ≥5 chronic conditions), representing information for 12 LAC countries.

General characteristics of selected studies

As shown in Table 1, Brazil contributes with a total of 20 data points [15-34]; Mexico with 4 data points [23, 35-37]; Peru with 3 data points [36, 38, 39]; Colombia with 2 data points [23, 40]; and Argentina [41], Cuba [36], Dominican Republic [36], El Salvador [23], Jamaica [23], Panama [23], Puerto Rico [36], and Venezuela [36] with 1 data point each. On the other hand, only 16 studies analyzed a nationally representative sample (Table 1).

For peer review only

Data from 37 country-level points were used to calculate pooled estimates using a definition of multimorbidity of ≥2 chronic conditions. Only one country (Brazil) used an additional definition of multimorbidity (i.e., ≥3 chronic conditions) in 8 different reports. Fourteen reports had information to calculate estimates for urban settings [15, 17-19, 22, 25-27, 29, 33, 35, 38, 39, 42], whereas 7 had information from rural areas [18, 25, 27, 35, 38, 39, 42].

Finally, the number of conditions evaluated to define multimorbidity ranged from 5 to 29, with a mean of 12.3 (SD: 5.7) conditions (Supplemental Material: e-Table 5). Hypertension and type 2 diabetes were the only conditions included in all the definitions of multimorbidity.

Synthesis of results

The meta-analysis included 26 studies with information with a pooled estimate of multimorbidity defined as ≥2 chronic conditions of 43% (95% CI: 35% - 51%; I^2: 99.9%; See Figure 2); whereas the pooled estimate for multimorbidity using ≥3 chronic conditions was 40% (95% CI: 22% - 57%; I^2: 99.9%).

As many of the studies were from Brazil, the pooled estimate of multimorbidity (≥2 chronic conditions) for this country was 50% (95% CI: 37% - 63%; I^2: 99.9%), whereas the pooled estimate for other countries together was 35% (95% CI: 26% - 43%; I^2: 99.7%). Nationally representative samples had a pooled estimate of multimorbidity of 37% (95% CI: 27% - 47%; I^2: 99.9%), whilst this estimate was 48% (95% CI: 35% - 61%; I^2: 99.9%) for non-nationally representative samples. Similarly, when multimorbidity was assessed as self-reported, pooled prevalence was 40% (95% CI: 31% - 48%; I^2: 99.9%), but the prevalence was 52% (95% CI: 33% - 70%; I^2: 99.9%) for those which have objectively measured and self-reported chronic conditions.

On the other hand, when analysis was conducted using studies whose data was collected from 2010 and onwards, pooled prevalence of multimorbidity was 48% (95% CI: 34% - 61%; I^2: 99.9%). This approach was used because time can have an impact on estimations due to the health transition in LMIC. Similarly, when assessing only studies including subjects of 50 years and over, the pooled prevalence of multimorbidity was 62% (95% CI: 51% - 73%; I^2: 99.9%).

In stratified analyses, pooled prevalence of multimorbidity was 38.9% (95% CI: 28.6% - 49.1%; I^2: 99.7%) for males and 50.5% (95% CI: 38.3% - 62.7%; I^2: 99.8%) for females. These estimates were 38.1% (95% CI: 26.1% - 50.1%; I^2: 87.3%) and 24.7% (95% CI: 12.5% - 36.8%; I^2: 50.8%) for urban and rural dwellers, respectively (See Figures in Supplementary Material: e-Figure 1-4).

For peer review only

In meta-regression analysis, the number of chronic conditions defining multimorbidity was strongly associated with heterogeneity (β: 0.02 per additional condition, p <0.001). Similarly, mean age was also strongly associated (β: 0.01 per additional year, p <0.001) and the proportion of women involved in the study (β: 0.75, p = 0.008). In addition, setting (urban vs. rural) was almost associated with heterogeneity (β: 0.59 compared to rural settings, p = 0.06).

Risk of bias

All reviewed studies had low risk of bias (Supplementary Material: e-Table 6). Nevertheless, sample size was not justified in three studies [23, 36, 38], and the outcome was self-reported in most of the studies except in 5 of them [21, 36, 38, 39, 41].

DISCUSSION

Summary of evidence

This systematic review provides a comprehensive analysis of the burden of multimorbidity in the LAC region. Accordingly, the pooled prevalence of multimorbidity was 43%. Nevertheless, there was a high heterogeneity among studies included, and results varied by the characteristics of the study as well as some of population characteristics. Thus, the pooled prevalence was lower in nationally representative compared to sub-national samples, whereas estimates were higher when only studies with measured and self-reported chronic conditions were included. In addition, age seems to be an important predictor as the prevalence of multimorbidity among those aged 50 years and over was high compared to the pooled estimate of multimorbidity. Finally, the pooled prevalence of multimorbidity was higher among females compared to males, highlighting the link between sex and multimorbidity, and among urban compared to those rural dwellers highlighting perhaps better access to diagnostic care in urban sites.

Limitations of the review

There are some limitations in this review that should be highlighted. First, high heterogeneity is present in almost all the results. This finding may be attributable to age group inclusion criteria since a great proportion of studies enrolled individuals aged ≥50+ years and the pooled estimate of multimorbidity was high in this group. In addition, the proportion of women enrolled in each individual study may be relevant as pointed out by the meta-regression analyses. Second, the number of chronic conditions as well as the list of them used to define multimorbidity is very dissimilar. Defining specific clusters of multimorbidity is needed to guarantee comparability

For peer review only

between studies, but this is not usually reported. Therefore, it is relevant to standardize the definition of multimorbidity and the conditions included in such definition to estimate which clusters of multimorbidity are more frequent and relevant for LAC region. In addition to that, before recent definition of multimorbidity, some reports used other different definitions (≥ 3 or ≥ 5 chronic conditions); which could affect pooled results. Fortunately, analysis was possible to include only those with ≥2 chronic conditions. Third, most of the studies included in the review were from Brazil, preventing inferability to the whole region, but also highlighting the need of population-based studies on multimorbidity in other countries of the region. Of note, the pooled prevalence of multimorbidity was higher in Brazil compared to other countries, perhaps because Brazilian researchers have addressed a common definition of multimorbidity, using a list of 12 conditions. Finally, a bias due to self-reporting can affect our results. Therefore, whether multimorbidity is defined by self-report or by more objective measurements may have an impact on prevalence estimates. Awareness of some chronic conditions are usually low and varying; for example, hypertension and diabetes awareness is around 64% and 78%, respectively in urban areas of Latin America [43], but tend to be lower in rural settings [44]. Thus, our results may be underestimating the real burden of multimorbidity in the LAC region.

Public Health Relevance

Global trends suggest that multimorbidity is a public health challenge; thus, understanding the epidemiology of multimorbidity in LAC region may be relevant as this issue has received little attention from researchers, but especially, policymakers. Moreover, much of the response of the health system has been developed based on one specific condition or specific body system, instead of an integral approach whereby multiple conditions are addressed synergistically. Therefore, disperse and heterogeneous information have been available for this systematic review.

Our results highlight the need of implementing a surveillance system focused on multimorbidity. This can be done by including some specific conditions in routine health surveys (Demographic Health Surveys or similar) and other population-based research studies. In addition, these surveys may include the most common clusters of multimorbidity, and those with higher morbidity and mortality [45] , however, such relevant clusters need to be appropriately defined.

Our results also imply that health systems need to be adapted to face the challenge of multimorbidity which increases healthcare use and costs related to primary and secondary prevention [2]. This adaptation process includes the appropriate training of human resources as

For peer review only

well as improving of health services infrastructure and care delivery. It is also needed to develop guidelines for multimorbidity care as that of NICE that includes clinical assessment and adequate management [46], but also highlight related issues as polypharmacy and life expectancy. Therefore, a holistic approach may be needed to tackle this global health problem.

Conclusions

Our systematic review shows that four of ten participants have multimorbidity at the population level in LAC. There is, however, a marked variability, depending on participant's age and the number of chronic conditions assessed, highlighting the need of better designed and standardized studies to inform the landscape of multimorbidity in LAC.

Statements

Contributorship statement: TSC-D, AMH-D and AB-O conceived the idea of the manuscript. TSC-D and AMH-D conducted the review with support of AB-O and RMC-L. RMC-L and AB-O conducted the statistical analysis. TSC-D and AMH-D drafted the first version of the manuscript, with relevant comments and suggestions of RMC-L and AB-O. All the authors approved the version submitted for publication.

Competing interests: None declared.

Funding: Rodrigo M Carrillo-Larco is supported by a Wellcome Trust International Training Fellowship (214185/Z/18/Z).

Data sharing statement: All the information of the present systematic review is included in the manuscript. There is no additional data available.

REFERENCES

1. The Academy of Medical Sciences. *Multimorbidity: a priority for health research*. London, UK: Academy of Medical Sciences; 2018.
2. Soley-Bori M, Ashworth M, Bisquera A, Dodhia H, Lynch R, Wang Y, et al. Impact of multimorbidity on healthcare costs and utilisation: a systematic review of the UK literature. *Br J Gen Pract* 2021;71(702):e39-e46.

For peer review only

3. Makovski TT, Schmitz S, Zeegers MP, Stranges S, van den Akker M. Multimorbidity and quality of life: Systematic literature review and meta-analysis. *Ageing Res Rev* 2019;53:100903.
4. Barnett K, Mercer SW, Norbury M, Watt G, Wyke S, Guthrie B. Epidemiology of multimorbidity and implications for health care, research, and medical education: a cross-sectional study. *Lancet* 2012;380(9836):37-43.
5. Banerjee A, Hurst J, Fottrell E, Miranda JJ. Multimorbidity: Not Just for the West. *Glob Heart* 2020;15(1):45.
6. Bilal U, Hessel P, Perez-Ferrer C, Michael YL, Alfaro T, Tenorio-Mucha J, et al. Life expectancy and mortality in 363 cities of Latin America. *Nat Med* 2021.
7. Violan C, Foguet-Boreu Q, Flores-Mateo G, Salisbury C, Blom J, Freitag M, et al. Prevalence, determinants and patterns of multimorbidity in primary care: a systematic review of observational studies. *PLoS ONE* 2014;9(7):e102149.
8. Pati S, Swain S, Hussain MA, van den Akker M, Metsemakers J, Knottnerus JA, et al. Prevalence and outcomes of multimorbidity in South Asia: a systematic review. *BMJ open* 2015;5(10):e007235.
9. Nguyen H, Manolova G, Daskalopoulou C, Vitoratou S, Prince M, Prina AM. Prevalence of multimorbidity in community settings: A systematic review and meta-analysis of observational studies. *J Comorb* 2019;9:2235042x19870934.
10. Abebe F, Schneider M, Asrat B, Ambaw F. Multimorbidity of chronic non-communicable diseases in low- and middle-income countries: A scoping review. *J Comorb* 2020;10:2235042x20961919.
11. Ouzzani M, Hammady H, Fedorowicz Z, Elmagarmid A. Rayyan-a web and mobile app for systematic reviews. *Syst Rev* 2016;5(1):210.
12. Modesti PA, Reboldi G, Cappuccio FP, Agyemang C, Remuzzi G, Rapi S, et al. Panethnic Differences in Blood Pressure in Europe: A Systematic Review and Meta-Analysis. *PLoS ONE* 2016;11(1):e0147601.
13. Nyaga VN, Arbyn M, Aerts M. Metaprop: a Stata command to perform meta-analysis of binomial data. *Arch Public Health* 2014;72(1):39.
14. Freeman MF, Tukey JW. Transformations related to the angular and the square root. *Ann Math Stat* 1950;21:607-11.

For peer review only

15. Aguiar LB, Baccaro LF, de Souza Santos Machado V, Pinto-Neto AM, Costa-Paiva L. Disability and multimorbidity in women older than 50 years: a population-based household survey. *Menopause (New York, NY)* 2015;22(6):660-6.
16. Amaral TLM, Amaral CdA, Lima NSd, Herculano PV, Prado PRd, Monteiro GTR. Multimorbidity, depression and quality of life among elderly people assisted in the Family Health Strategy in Senador Guiomard, Acre, Brazil. *Multimorbidade, depressao e qualidade de vida em idosos atendidos pela Estrategia de Saude da Familia em Senador Guiomard, Acre, Brasil* 2018;23(9):3077-84.
17. Andrade LH, Benseñor IM, Viana MC, Andreoni S, Wang YP. Clustering of psychiatric and somatic illnesses in the general population: multimorbidity and socioeconomic correlates. *Braz j med biol res* 2010;43(5):483-91.
18. Araujo MEA, Silva MT, Galvao TF, Nunes BP, Pereira MG. Prevalence and patterns of multimorbidity in Amazon Region of Brazil and associated determinants: a cross-sectional study. *BMJ open* 2018;8(11):e023398.
19. Boing AF, Melo GR, Boing AC, Moretti-Pires RO, Peres KG, Peres MA. Associação entre depressão e doenças crônicas: um estudo populacional. *Rev saúde pública* 2012;46(4):617-23.
20. Costa CdS, Flores TR, Wendt A, Neves RG, Tomasi E, Cesar JA, et al. Inequalities in multimorbidity among elderly: a population-based study in a city in Southern Brazil. *Cad Saúde Pública (Online)* 2018;34(11):e00040718-e.
21. da Silva Almeida IL, dos Santos SR, Morbeck de Queiroz B, de Freitas Mussi RF. Lifestyle, morbidity and multimorbidity in adult Quilombolas. *ABCS health sci* 2020;45:1325.
22. de Souza Santos Machado V, Valadares ALR, da Costa-Paiva LS, Moraes SS, Pinto-Neto AM. Multimorbidity and associated factors in Brazilian women aged 40 to 65 years: a population-based study. *Menopause (New York, NY)* 2012;19(5):569-75.
23. Macinko J, Andrade FCD, Nunes BP, Guanais FC. Primary care and multimorbidity in six Latin American and Caribbean countries. *Rev panam salud pública* 2019;43:e8-e.
24. Montes MC, Bortolotto CC, Tomasi E, Gonzalez MC, Barbosa-Silva TG, Domingues MR, et al. Strength and multimorbidity among community-dwelling elderly from southern Brazil. *Nutrition* 2020;71.

For peer review only

25. Nunes BP, Batista SRR, Andrade FBd, Souza Junior PRBd, Lima-Costa MF, Facchini LA. Multimorbidity: The Brazilian Longitudinal Study of Aging (ELSI-Brazil). *Revista de saude publica* 2018;52Suppl 2(Suppl 2):10s.

26. Nunes BP, Camargo-Figuera FA, Guttier M, de Oliveira PD, Munhoz TN, Matijasevich A, et al. Multimorbidity in adults from a southern Brazilian city: occurrence and patterns. *International journal of public health* 2016;61(9):1013-20.

27. Nunes BP, Chiavegatto Filho ADP, Pati S, Cruz Teixeira DS, Flores TR, Camargo-Figuera FA, et al. Contextual and individual inequalities of multimorbidity in Brazilian adults: a cross-sectional national-based study. *BMJ open* 2017;7(6):e015885.

28. Nunes BP, Soares MU, Wachs LS, Volz PM, Saes MdO, Duro SMS, et al. Hospitalization in older adults: association with multimorbidity, primary health care and private health plan. *Rev saúde pública* 2017;51:43-.

29. Nunes BP, Thume E, Facchini LA. Multimorbidity in older adults: magnitude and challenges for the Brazilian health system. *BMC public health* 2015;15:1172.

30. Padilha Pereira B, Cardozo Bortolotto C, Tomasi E, Gonzalez MC, Gomes AP, Goncalves H, et al. Food consumption and multimorbidity among non-institutionalized elderly people in Pelotas, 2014: a cross-sectional study. *Epidemiol serv saúde* 2020;29(3):e2019050.

31. Petarli GB, Cattafesta M, Sant'Anna MM, Bezerra OMdPA, Zandonade E, Salaroli LB. Multimorbidity and complex multimorbidity in Brazilian rural workers. *PLoS ONE* 2019;14(11):e0225416.

32. Tavares DMDS, Pelizaro PB, Pegorari MS, Paiva MMd, Marchiori GF. Prevalence of self-reported morbidities and associated factors among community-dwelling elderly in Uberaba, Minas Gerais, Brazil. *Prevalencia de morbidades autorreferidas e fatores associados entre idosos comunitarios de Uberaba, Minas Gerais, Brasil* 2019;24(9):3305-13.

33. Valadares ALR, Lui-Filho JF, Costa-Paiva L, Pinto-Neto AM. Middle-aged female sexual dysfunction and multimorbidity: a population-based study. *Menopause (New York, NY)* 2016;23(3):304-10.

34. Wang Y-P, Nunes BP, Coelho BM, Santana GL, do Nascimento CF, Viana MC, et al. Multilevel Analysis of the Patterns of Physical-Mental Multimorbidity in General Population of Sao Paulo Metropolitan Area, Brazil. *Scientific reports* 2019;9(1):2390.

For peer review only

35. Agrawal S, Agrawal PK. Association Between Body Mass index and Prevalence of Multimorbidity in Low-and Middle-income Countries: A Cross-Sectional Study. *International journal of medicine and public health* 2016;6(2):73-83.

36. Bao J, Chua K-C, Prina M, Prince M. Multimorbidity and care dependence in older adults: a longitudinal analysis of findings from the 10/66 study. *BMC public health* 2019;19(1):585.

37. Bustos-Vazquez E, Fernandez-Nino JA, Astudillo-Garcia CI. Self-rated health, multimorbidity and depression in Mexican older adults: Proposal and evaluation of a simple conceptual model. *Biomedica : revista del Instituto Nacional de Salud* 2017;37(0):92-103.

38. Miranda JJ, Bernabe-Ortiz A, Gilman RH, Smeeth L, Malaga G, Wise RA, et al. Multimorbidity at sea level and high-altitude urban and rural settings: The CRONICAS Cohort Study. *Journal of Comorbiddity* 2019;9:1-10.

39. Taype-Rondan A, Abbs ES, Lazo-Porras M, Checkley W, Gilman RH, Smeeth L, et al. Association between chronic conditions and health-related quality of life: differences by level of urbanization in Peru. *Quality of life research : an international journal of quality of life aspects of treatment, care and rehabilitation* 2017;26(12):3439-47.

40. Camargo-Casas S, Suarez-Monsalve S, Zepeda MUP, Garcia-Pena C, Cano-Gutierrez CA. [Multimorbidity, Depressive Symptoms, and Self-Reported Health in Older Adults: a Secondary Analysis of the Sabe Bogota Study]. *Revista de investigacion clinica; organo del Hospital de Enfermedades de la Nutricion* 2018;70(4):192-7.

41. Olivares DEV, Chambi FRV, Chani EMM, Craig WJ, Pacheco SOS, Pacheco FJ. Risk Factors for Chronic Diseases and Multimorbidity in a Primary Care Context of Central Argentina: A Web-Based Interactive and Cross-Sectional Study. *International journal of environmental research and public health* 2017;14(3).

42. Cavalcanti G, Doring M, Portella MR, Bortoluzzi EC, Mascarelo A, Dellani MP. Multimorbidity associated with polypharmacy and negative self-perception of health. *Rev bras geriatr gerontol (Online)* 2017;20(5):634-42.

43. Silva H, Hernandez-Hernandez R, Vinueza R, Velasco M, Boissonnet CP, Escobedo J, et al. Cardiovascular risk awareness, treatment, and control in urban Latin America. *Am J Ther* 2010;17(2):159-66.

For peer review only

44. Lerner AG, Bernabe-Ortiz A, Gilman RH, Smeeth L, Miranda JJ. The "rule of halves" does not apply in Peru: awareness, treatment, and control of hypertension and diabetes in rural, urban, and rural-to-urban migrants. *Crit Pathw Cardiol* 2013;12(2):53-8.
45. Whitty CJM, Watt FM. Map clusters of diseases to tackle multimorbidity. *Nature* 2020;579(7800):494-6.
46. National Institute for Health and Care Excellence (NICE). Multimorbidity: clinical assessment and management: NICE guideline [NG56]. NICE; 2016 [updated 2016; cited 2021 January 30]; Available from: https://www.nice.org.uk/guidance/ng56.

For peer review only

TABLES AND FIGURES

Figure 1: Flowchart of studies included in the systematic review

For peer review only

Figure 2: Forest plot of the pooled prevalence of multimorbidity defined as ≥2 chronic conditions

For peer review only

Table 1: Characteristics of the studies included in the systematic review

First author	Country	Study design	Data collected in	Sample size	Definition of multimorbidity	Age range	Age mean	% women	% urban
Andrade [17]	Brazil	Cross-sectional	1994-1995	1,464	≥2 (self-reported)	18+	--	57.4%	100%
Boing [19]	Brazil	Cross-sectional	2009-2010	1,720	≥2 (self-reported)	20 - 59	--	55.6%	100%
De Souza Santos Machado [22]	Brazil	Cross-sectional	2005	377	≥2 (self-reported)	40 - 65	--	100%	100%
Aguiar [15]	Brazil	Cross-sectional	2011	622	≥2 (self-reported)	50+	64.1	100%	100%
Nunes [29]	Brazil	Cross-sectional	2008	1,593	≥2 and ≥3 (self-reported)	60+	--	62.8%	100%
Agrawal [35]	Mexico	Cross-sectional	2007-2010	2,725	≥2 (self-reported)	18+	63.1	61.8%	73.7%
Nunes [26]	Brazil	Cross-sectional	2012	2,927	≥2 and ≥3 (self-reported)	20+	45.7	58.9%	100%
Valadares [33]	Brazil	Cross-sectional	2012-2013	736	≥2 (self-reported)	45 - 60	52.5	100%	100%
Bustos-Vazquez [37]	Mexico	Cross-sectional	2012	7,967	≥2 (self-reported)	60+	69.3	53.4%	--
Cavalcanti [42]	Brazil	Cross-sectional	2010-2011	676	≥2 (self-reported)	60+	70.0	54.6%	69.4%
Nunes [27]	Brazil	Cross-sectional	2013	60,202	≥2 and ≥3 (self-reported)	18+	43.7	55.1%	86.5%
Nunes [28]	Brazil	Cross-sectional	2008	1,593	≥2 and ≥3 (self-reported)	60+	--	62.8%	--
Olivares [41]	Argentina	Cross-sectional	2014-2015	1,044	≥2 (self-reported)	18+	43.0	65%	--

For peer review only

Taype-Rondan [39]	Peru	Longitudinal	2013-2014	2,433	≥2 (self-reported and measured)	35+	57.2	51.3%	51.3%
Amaral [16]	Brazil	Cross-sectional	2010	264	≥2 (self-reported)	60+	--	61%	100%
Araujo [18]	Brazil	Cross-sectional	2015	4,001	≥2 and ≥3 (self-reported)	18+	--	52.7%	86.8%
Camargo-Casas [40]	Colombia	Cross-sectional	2012	2,000	≥2 (self-reported)	60+	71.1	63.4%	--
Costa [20]	Brazil	Cross-sectional	2014	1,451	≥2 and ≥3 (self-reported and measured)	60+	--	63%	--
Nunes [25]	Brazil	Longitudinal	2015-2016	9,412	≥2 and ≥3 (self-reported)	50+	62.9	54%	84.7%
Bao [36]	Cuba	Longitudinal	2003-2007	2,944	≥2 (self-reported and measured)	65+	73.9	64%	--
	Dominican Republic			2,011			74.5	65%	--
	Puerto Rico			2,009			75.2	67%	--
	Venezuela			1,965			71.5	63%	--
	Peru			1,933			74.2	60%	--
	Mexico			2,003			73.7	64%	--
Macinko [23]	Brazil	Cross-sectional	2013-2014	1,486	≥2 (self-reported)	18+	--	51.1%	--
	Colombia			1,485			--	51.1%	--
	El Salvador			1,460			--	52.8%	--
	Jamaica			1,480			--	52.6%	--
	Mexico			1,492			--	51.4%	--
	Panama			1,475			--	51.6%	--
Miranda [38]	Peru	Longitudinal	2010-2012	2,890	≥2 (self-reported and measured)	35+	55.2	51%	50.9%
Petarli [31]	Brazil	Cross-sectional	2016-2017	790	≥2 and ≥3 (self-reported)	18 - 59	--	47.7%	--
Tavares [32]	Brazil	Cross-sectional	2012	1,691	≥2 (self-reported)	60+	72.5	63.7%	100%

For peer review only

Wang [34]	Brazil	Cross-sectional	2005-2007	2,713	≥2 (self-reported)	18 - 64	--	52,4%	--
Montes [24]	Brazil	Cross-sectional	2014	1,336	≥5 (self-reported)	60+	--	63.1%	--
Padilha Pereira [30]	Brazil	Cross-sectional	2014	1,426	≥5 (self-reported)	60+	--	63%	--
Da Silva Almeida [21]	Brazil	Cross-sectional	2016	850	≥2 (self-reported and measured)	18+	--	61.2%	--

For peer review only

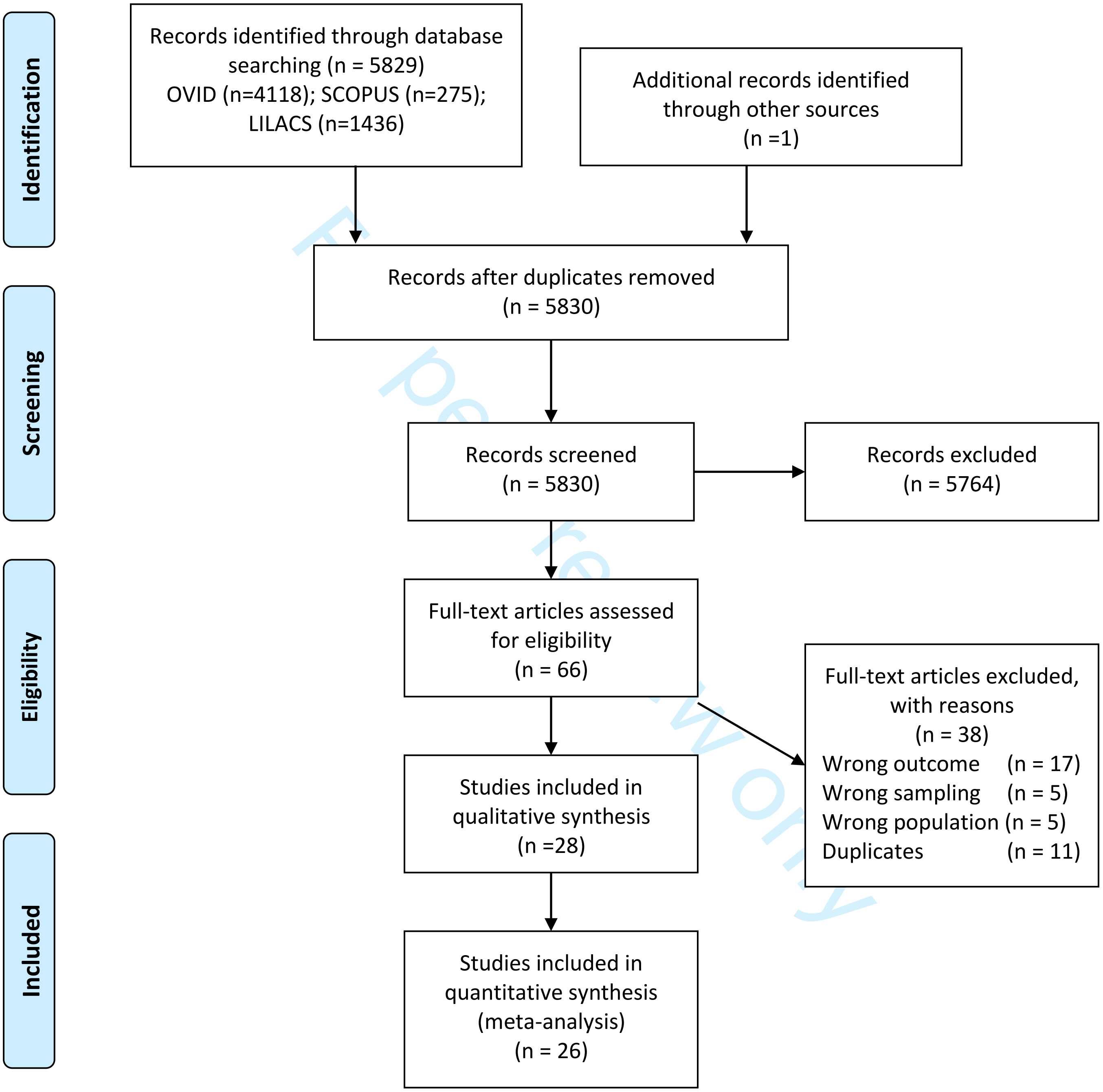

For peer review only - http://bmjopen.bmj.com/site/about/guidelines.xhtml

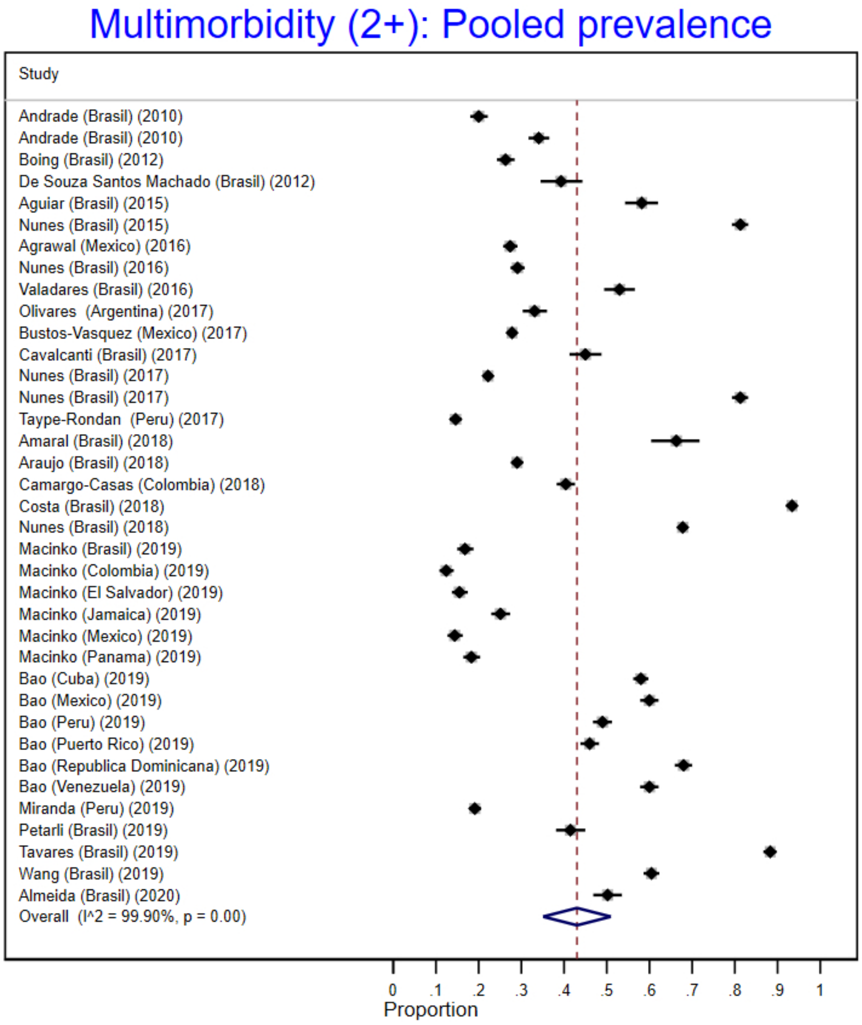

Figure 2: Forest plot of the pooled prevalence of multimorbidity defined as ≥2 chronic conditions

291x319mm (72 x 72 DPI)

SUPPLEMENTAL MATERIAL

E-Figure 1: Forest plot with pooled estimates of multimorbidity among males

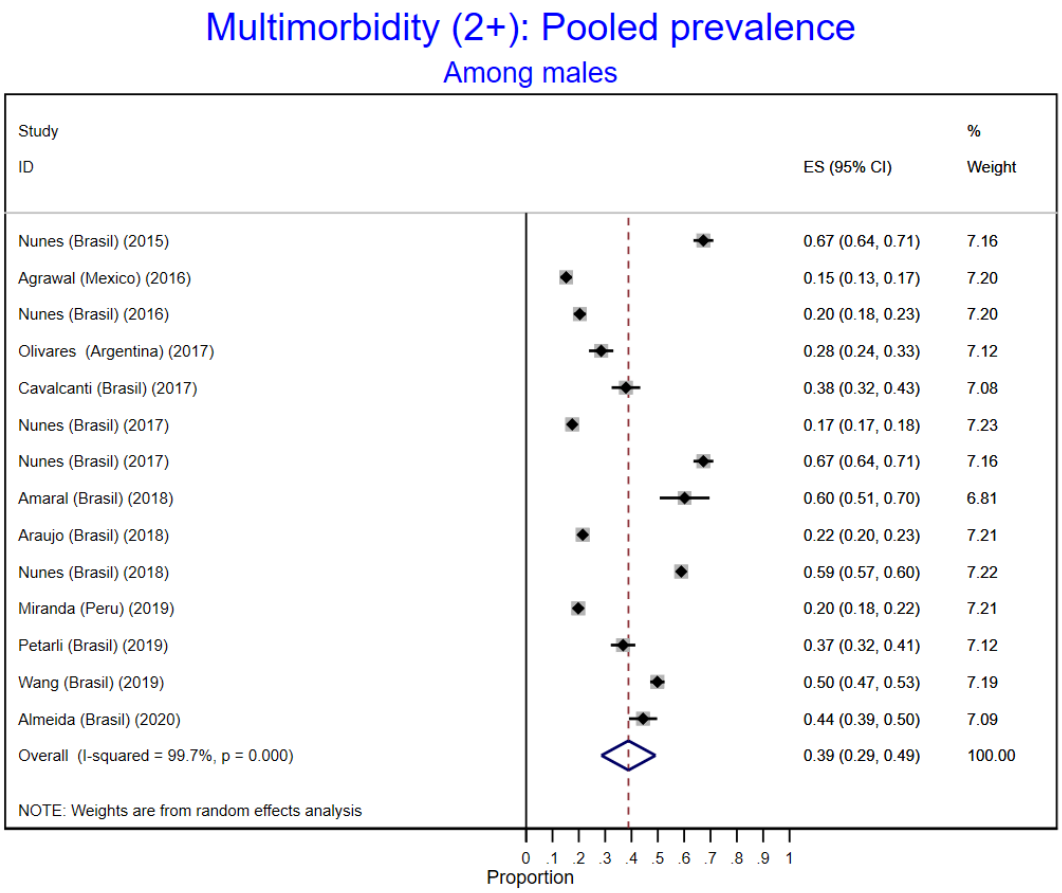

E-Figure 2: Forest plot with pooled estimates of multimorbidity among females

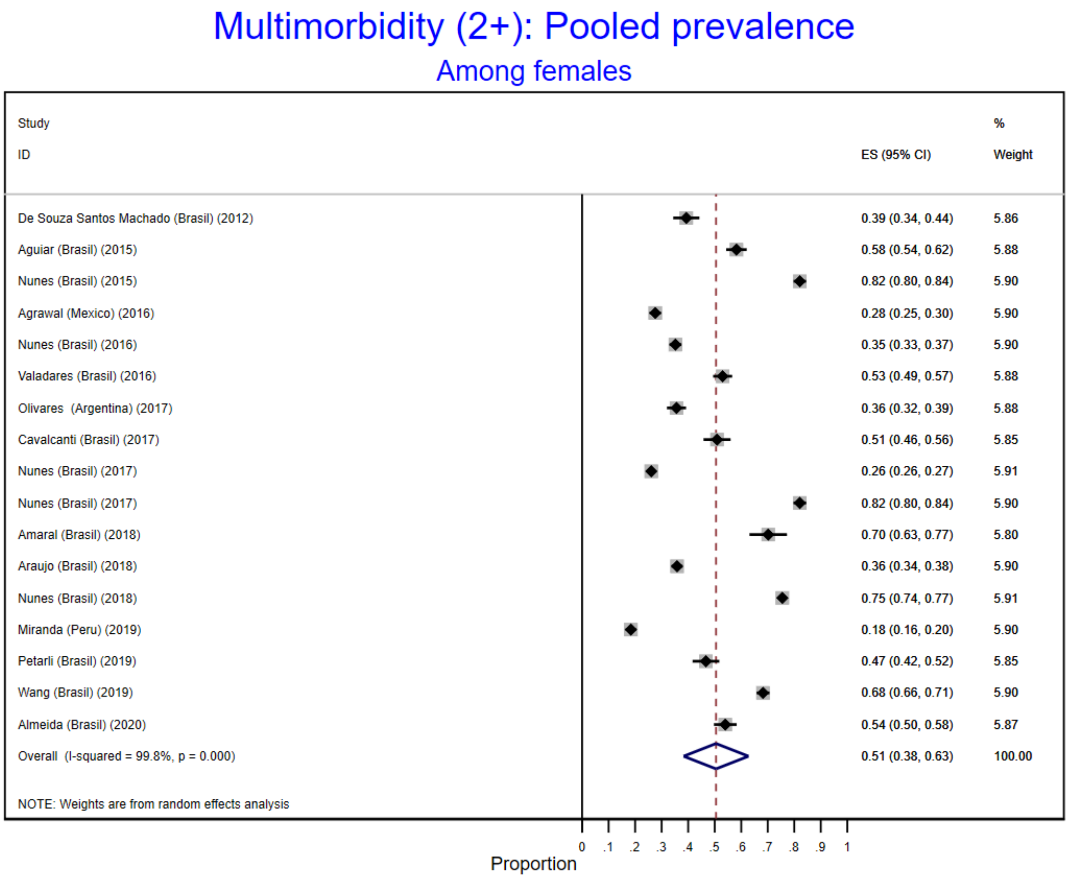

E-Figure 3: Forest plot with pooled estimates of multimorbidity among urban dwellers

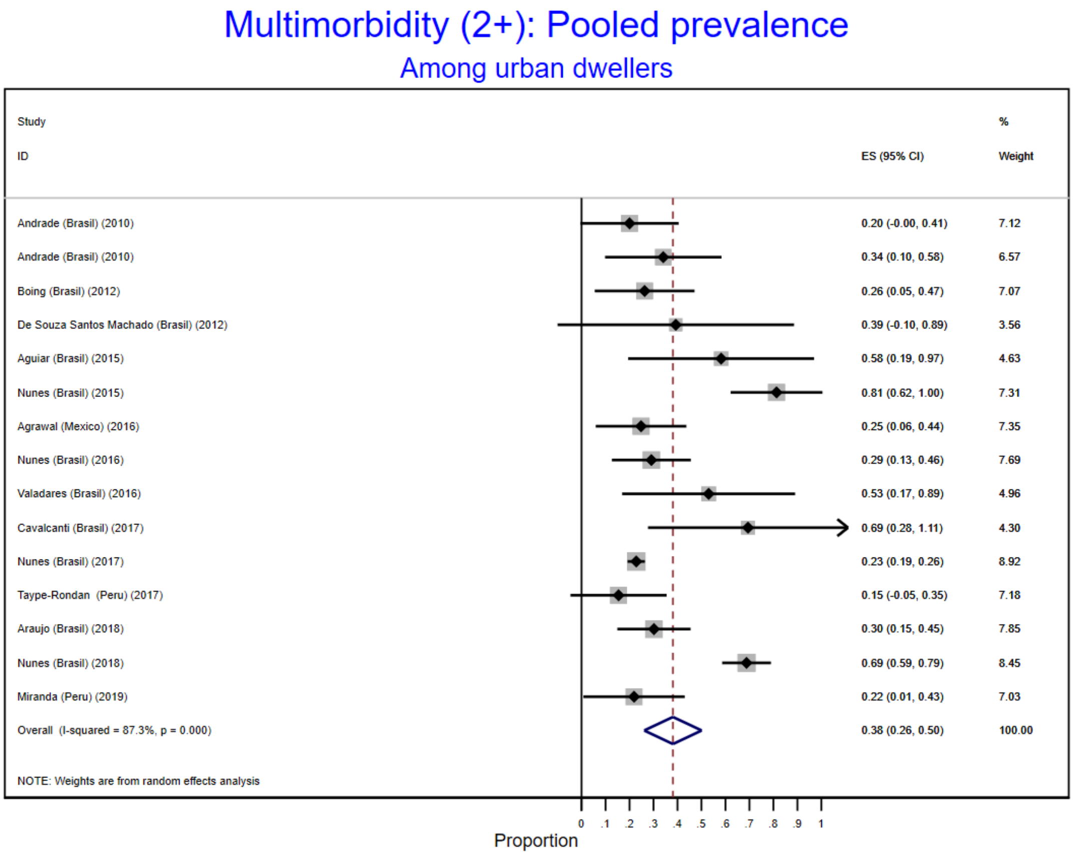

E-Figure 4: Forest plot with pooled estimates of multimorbidity among rural dwellers

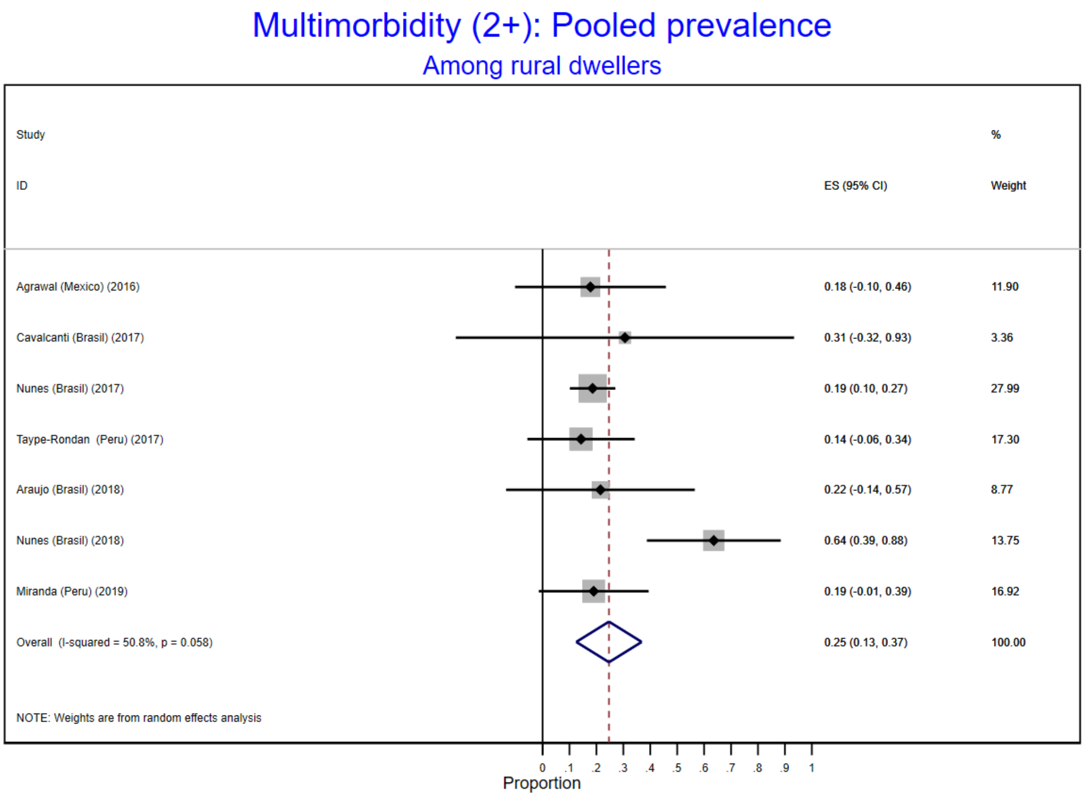

nly

E-Table 1: Search terms and strategy using OVID (Embase, Medline and Global Health)

1	multimorbidit*.mp
2	multi-morbidit*.mp
3	(multi adj morbidit*).mp
4	multiple morbidit*.mp
5	(co adj morbidit*).mp
6	comorbidit*.mp
7	“multiple chronic conditions”.mp
8	“multiple chronic illnesses”.mp
9	“multiple chronic diseases”.mp
10	(poly adj morbidit*).mp
11	polymorbidit*.mp
12	poly-morbidit*.mp
13	1 or 2 or 3 or 4 or 5 or 6 or 7 or 8 or 9 or 10 or 11 or 12
14	(("Antigua and Barbuda") or ("Argentina") or ("Bahamas") or ("Barbados") or ("Belize") or ("Bolivia") or ("Brazil") or ("United States Virgin Islands") or ("British Virgin Islands") or ("Chile") or ("Colombia") or ("Costa Rica") or ("Cuba") or ("Dominica") or ("Dominican Republic") or ("Ecuador") or ("El Salvador") or ("Grenada") or ("Guatemala") or ("Guyana") or ("Haiti") or ("Honduras") or ("Jamaica") or ("Mexico") or ("Nicaragua") or ("Panama") or ("Paraguay") or ("Peru") or ("Puerto Rico") or ("Saint Kitts and Nevis") or ("Saint Lucia") or ("Saint Vincent and the Grenadines") or ("Suriname") or ("Trinidad and Tobago") or ("West Indies") or ("Uruguay") or ("Venezuela") or ("Latin America") or latin amer$ or ("South America") or south amer$ or ("Central America") or central amer$ or ("Caribbean Region"))
15	13 and 14
16	exp animals/ not humans.sh.
17	15 not 16
18	remove duplicates from 17

For peer review only

E-Table 2: Search terms and strategy using Scopus

(TITLE-ABS-KEY(multimorbidity$) OR TITLE-ABS-KEY(multi-morbidit$) OR TITLE-ABS-KEY(multi-morbidit$) OR TITLE-ABS-KEY(multi Pre/1 morbidit$) OR TITLE-ABS-KEY(multiple morbidit$) OR TITLE-ABS-KEY(co Pre/1 morbidit$) OR TITLE-ABS-KEY(comorbidit$) OR TITLE-ABS-KEY(multiple chronic conditions) OR TITLE-ABS-KEY(multiple chronic illnesses) OR TITLE-ABS-KEY(multiple chronic diseases) OR TITLE-ABS-KEY(poly Pre/1 morbidit$) OR TITLE-ABS-KEY(polymorbidit$) OR TITLE-ABS-KEY(poly-morbidit$)) AND (TITLE-ABS-KEY("Antigua and Barbuda") or TITLE-ABS-KEY("Argentina") or TITLE-ABS-KEY("Bahamas") or TITLE-ABS-KEY("Barbados") or TITLE-ABS-KEY("Belize") or TITLE-ABS-KEY("Bolivia") or TITLE-ABS-KEY("Brazil") or TITLE-ABS-KEY("United States Virgin Islands") or TITLE-ABS-KEY("British Virgin Islands") or TITLE-ABS-KEY("Chile") or TITLE-ABS-KEY("Colombia") or TITLE-ABS-KEY("Costa Rica") or TITLE-ABS-KEY("Cuba") or TITLE-ABS-KEY("Dominica") or TITLE-ABS-KEY("Dominican Republic") or TITLE-ABS-KEY("Ecuador") or TITLE-ABS-KEY("El Salvador") or TITLE-ABS-KEY("Grenada") or TITLE-ABS-KEY("Guatemala") or TITLE-ABS-KEY("Guyana") or TITLE-ABS-KEY("Haiti") or TITLE-ABS-KEY("Honduras") or TITLE-ABS-KEY("Jamaica") or TITLE-ABS-KEY("Mexico") or TITLE-ABS-KEY("Nicaragua") or TITLE-ABS-KEY("Panama") or TITLE-ABS-KEY("Paraguay") or TITLE-ABS-KEY("Peru") or TITLE-ABS-KEY("Puerto Rico") or TITLE-ABS-KEY("Saint Kitts and Nevis") or TITLE-ABS-KEY("Saint Lucia") or TITLE-ABS-KEY("Saint Vincent and the Grenadines") or TITLE-ABS-KEY("Suriname") or TITLE-ABS-KEY("Trinidad and Tobago") or TITLE-ABS-KEY("West Indies") or TITLE-ABS-KEY("Uruguay") or TITLE-ABS-KEY("Venezuela") or TITLE-ABS-KEY("Latin America") or TITLE-ABS-KEY(latin amer$) or TITLE-ABS-KEY("South America") or TITLE-ABS-KEY(south amer$) or TITLE-ABS-KEY("Central America") or TITLE-ABS-KEY(central amer$) or TITLE-ABS-KEY("Caribbean Region")) AND NOT DBCOLL(medl)

E-Table 3: Search terms and strategy using LILACS

((multi-morbilidad) OR (multimorbilidad) OR (co-morbilidad) OR (comorbilidad) OR (multiple morbilidad) OR (multiples condiciones cronicas) OR (multiples enfermedades cronicas) OR (multiples patologias cronicas) OR (poli-morbilidad) OR (polimorbilidad)) AND (("Antigua y Barbuda") OR ("Argentina") OR ("Aruba") OR ("Bahamas") OR ("Barbados") OR ("Belice") OR ("Bolivia") OR ("Brasil") OR ("Islas Vírgenes de los Estados Unidos") OR ("Islas Vírgenes Británicas") OR ("Islas Caimán") OR ("Chile") OR ("Colombia") OR ("Costa Rica") OR ("Cuba") OR ("Curazao") OR ("Dominica") OR ("Republica Dominicana") OR ("Ecuador") OR ("El Salvador") OR ("Granada") OR ("Guatemala") OR ("Guyana") OR ("Haití") OR ("Honduras") OR ("Jamaica") OR ("México") OR ("Nicaragua") OR ("Panamá") OR ("Paraguay") OR ("Perú") OR ("Puerto Rico") OR ("San Cristóbal y Nieves ") OR ("Santa Lucía") OR ("San Vicente y las Granadinas ") OR ("Surinam") OR ("Trinidad y Tobago") OR ("Turcas y Caicos ") OR ("Uruguay") OR ("Venezuela") OR ("América Latina") OR ("Latinoamérica") OR ("América del Sur") OR ("Sudamérica") OR ("Suramérica") OR ("América Central") OR ("Centroamérica") OR ("América del Centro") OR ("Caribe"))

E-Table 4: PRISMA Checklist

Section/topic	#	Checklist item	Reported on page #
TITLE			
Title	1	Identify the report as a systematic review, meta-analysis, or both.	01
ABSTRACT			
Structured summary	2	Provide a structured summary including, as applicable: background; objectives; data sources; study eligibility criteria, participants, and interventions; study appraisal and synthesis methods; results; limitations; conclusions and implications of key findings; systematic review registration number.	02
INTRODUCTION			
Rationale	3	Describe the rationale for the review in the context of what is already known.	03
Objectives	4	Provide an explicit statement of questions being addressed with reference to participants, interventions, comparisons, outcomes, and study design (PICOS).	03
METHODS			
Protocol and registration	5	Indicate if a review protocol exists, if and where it can be accessed (e.g., Web address), and, if available, provide registration information including registration number.	03-04
Eligibility criteria	6	Specify study characteristics (e.g., PICOS, length of follow-up) and report characteristics (e.g., years considered, language, publication status) used as criteria for eligibility, giving rationale.	04
Information sources	7	Describe all information sources (e.g., databases with dates of coverage, contact with study authors to identify additional studies) in the search and date last searched.	04
Search	8	Present full electronic search strategy for at least one database, including any limits used, such that it could be repeated.	Suppl material e-Tables 1-3
Study selection	9	State the process for selecting studies (i.e., screening, eligibility, included in systematic review, and, if applicable, included in the meta-analysis).	04
Data collection process	10	Describe method of data extraction from reports (e.g., piloted forms, independently, in duplicate) and any processes for obtaining and confirming data from investigators.	04

Data items	11	List and define all variables for which data were sought (e.g., PICOS, funding sources) and any assumptions and simplifications made.	04
Risk of bias in individual studies	12	Describe methods used for assessing risk of bias of individual studies (including specification of whether this was done at the study or outcome level), and how this information is to be used in any data synthesis.	04-05
Summary measures	13	State the principal summary measures (e.g., risk ratio, difference in means).	05
Synthesis of results	14	Describe the methods of handling data and combining results of studies, if done, including measures of consistency (e.g., I^2) for each meta-analysis.	05
Risk of bias across studies	15	Specify any assessment of risk of bias that may affect the cumulative evidence (e.g., publication bias, selective reporting within studies).	NA
Additional analyses	16	Describe methods of additional analyses (e.g., sensitivity or subgroup analyses, meta-regression), if done, indicating which were pre-specified.	05
RESULTS			
Study selection	17	Give numbers of studies screened, assessed for eligibility, and included in the review, with reasons for exclusions at each stage, ideally with a flow diagram.	05 and Figure 1
Study characteristics	18	For each study, present characteristics for which data were extracted (e.g., study size, PICOS, follow-up period) and provide the citations.	05 and Table 1
Risk of bias within studies	19	Present data on risk of bias of each study and, if available, any outcome level assessment (see item 12).	Suppl Material eTable 5
Results of individual studies	20	For all outcomes considered (benefits or harms), present, for each study: (a) simple summary data for each intervention group (b) effect estimates and confidence intervals, ideally with a forest plot.	06 and Suppl Material e-Table 6
Synthesis of results	21	Present results of each meta-analysis done, including confidence intervals and measures of consistency.	06 and Tables
Risk of bias across studies	22	Present results of any assessment of risk of bias across studies (see Item 15).	NA
Additional analysis	23	Give results of additional analyses, if done (e.g., sensitivity or subgroup analyses, meta-regression [see Item 16]).	06 and Suppl Material e-Figure 1-4

For peer review only

DISCUSSION			
Summary of evidence	24	Summarize the main findings including the strength of evidence for each main outcome; consider their relevance to key groups (e.g., healthcare providers, users, and policy makers).	06
Limitations	25	Discuss limitations at study and outcome level (e.g., risk of bias), and at review-level (e.g., incomplete retrieval of identified research, reporting bias).	07
Conclusions	26	Provide a general interpretation of the results in the context of other evidence, and implications for future research.	07-08
FUNDING			
Funding	27	Describe sources of funding for the systematic review and other support (e.g., supply of data); role of funders for the systematic review.	01

For peer review only

E-Table 5: Coverage and conditions included in the definition of multimorbidity

First author	Study coverage	Number of conditions	Conditions included in the definition of multimorbidity
Andrade [1]	Sub-national	8	Back pain, hypertension, asthma, headache, heart problems, diabetes, stroke, and cancer
Andrade [1]	Sub-national	15	Cognitive impairment, non-affective psychosis, panic attacks, generalized anxiety, any phobias, obsessive-compulsive disorders, depression, dysthymia, bipolar disorder, bulimia, alcohol use disorder, substance use disorder, nicotine dependence, somatoform disorder, and dissociative disorder
Boing [2]	Sub-national	11	Problems in the spine or on the back, arthritis or rheumatism, cancer, diabetes, bronchitis or asthma, hypertension, heart disease, chronic renal failure, tuberculosis, tendinitis or tenosynovitis, and cirrhosis
De Souza Santos Machado [3]	Sub-national	5	Depression, hypertension, diabetes mellitus, urinary incontinence, and insomnia
Aguiar [4]	Sub-national	10	Hypertension, myocardial infarction, stroke, diabetes, pulmonary emphysema, bronchitis/asthma, arthrosis, osteoporosis, cataract, and urinary incontinence
Nunes [5]	Sub-national	17	High blood pressure, diabetes, lung problem, heart problem, stroke, rheumatism, arthritis or arthrosis, disease in spinal column (any problem reported), cancer, kidney problem, cognitive impairment, depression, urinary incontinence, amputation in any part of the body, eyesight problem, hearing problem, problem chewing food, and falls
Agrawal [6]	National	9	Angina pectoris, arthritis, asthma, chronic lung disease, diabetes, hypertension, stroke, depression, and low visual acuity
Nunes [7]	Sub-national	11	High blood pressure, depression, heart disease, hypercholesterolemia, diabetes, bronchitis, asthma/wheezy bronchitis, hypothyroidism, osteoporosis, joint problems, and emphysema
Valadares [8]	Sub-national	11	Hypertension, diabetes mellitus, dyslipidemia, myocardial infarction, stroke, deep venous thrombosis or pulmonary embolus, asthma or bronchitis, chronic rheumatic diseases, depression, anxiety, and cancer
Bustos-Vazquez [9]	National	7	Cancer, systemic arterial hypertension, diabetes, depression, cerebral infarction, hypercholesterolemia, and cardiovascular diseases
Cavalcanti [10]	Sub-national	8	Systemic arterial hypertension, diabetes mellitus, rheumatism, pulmonary problems (asthma, bronchitis and pulmonary emphysema), stroke/cerebral ischemia, arthritis/osteoporosis, heart problems (coronary disease, angina, congestive disease, others), and depression

For peer review only

Nunes [11]	National	22	High blood pressure, spinal column problem, hypercholesterolemia, depression, diabetes, arthritis/rheumatism, asthma/wheezy bronchitis, cancer, other heart disease, work-related muscle-skeletal disorders, stroke, heart attack, kidney problem, heart failure, bronchitis, angina, emphysema, other lung disease, bipolar disorder, other mental disease, schizophrenia, and obsessive-compulsive disorder
Nunes [12]	Sub-national	17	Systemic arterial hypertension, diabetes mellitus, heart disease, pulmonary problems, stroke, rheumatism/arthritis/osteoarthritis, spine problems, cancer, kidney failure, cognitive deficit, depression, visual problems, urinary incontinence, amputation, auditory problems, problem or difficulty chewing food, and falls
Olivares [13]	Sub-national	9	Hypertension, diabetes, high cholesterol, AMI or stroke, asthma, hypothyroidism, celiac disease, cancer and other chronic diseases (arthritis, osteoarthritis, osteoporosis, depression, chronic allergies, COPD; gastritis, migraine, irritable bowel syndrome, epilepsy, psychosis, multiple sclerosis, psoriasis, gout disease, herniated disc, inflammatory vasculitis)
Taype-Rondan [14]	Sub-national	6	Hypertension, chronic bronchitis, depressive mood, type 2 diabetes, heart disease, and stroke
Amaral [15]	Sub-national	8	Hypertension, diabetes, cardiovascular disorder (poor circulation, cerebrovascular accident (CVA or stroke) and heart problem), musculoskeletal disorder (spinal problem, rheumatism, arthritis/ arthrosis and osteoporosis), insomnia, cataracts, anemia and gastrointestinal/urinary disorders
Araujo [16]	Sub-national	12	Hypertension, diabetes, high cholesterol, heart disease (heart attack, angina, heart failure or other), stroke, asthma or asthmatic bronchitis, arthritis or rheumatism, depression, pulmonary disease (pulmonary emphysema, chronic bronchitis or chronic obstructive pulmonary disease), cancer, chronic kidney disease, and chronic spinal problems (chronic back or neck pain, low back pain, sciatic pain, vertebral or disc problems)
Camargo-Casas [17]	Sub-national	12	Hypertension, diabetes, cancer, chronic obstructive pulmonary disease, heart attack, heart failure, stroke, arthritis or osteoarthritis, osteoporosis, gastroesophageal reflux disease, gastritis, and ulcer
Costa [18]	Sub-national	29	High blood pressure, diabetes, heart problem, heart failure, asthma, bronchitis, emphysema, arthritis, Parkinson's disease, kidney failure, hypercholesterolemia, seizure, stomach ulcer, osteoporosis, urinary incontinence, constipation, fecal incontinence, depression, glaucoma, deafness, difficulty swallowing, insomnia, fainting, rhinitis, difficulty speaking, stroke, obesity, mental disorders, and cancer
Nunes [19]	National	19	Systemic arterial hypertension, back problems, high cholesterol, cataract, arthritis or rheumatism, depression, diabetes, osteoporosis heart problem, glaucoma, emphysema

For peer review only

			chronic bronchitis or chronic obstructive pulmonary disease, cerebrovascular accident, cancer, asthma, chronic renal failure, diabetic retinopathy, macular degeneration, Parkinson's disease, and Alzheimer's disease
Bao [20]	National	15	Vision, hearing, joints, skin, gastrointestinal, stroke, chronic obstructive pulmonary disease, hypertension, diabetes, ischemic heart disease, heart failure, heart valve disease, depression, anxiety, and dementia
Macinko [21]	National	8	Arthritis, asthma, cancer, depression, diabetes, heart disease, high cholesterol, and hypertension
Miranda [22]	Sub-national	6	Alcohol disorder, asthma, chronic obstructive pulmonary disease, depression, diabetes, and hypertension
Petarli [23]	Sub-national	20	Heart arrhythmia, infarction, stroke, diabetes mellitus, herniated disk, arthrosis, repetitive strain injuries/work-related musculoskeletal disorders, renal disease, Parkinson's, Alzheimer's, hepatic cirrhosis, infertility, cancer, asthma, chronic bronchitis, and pulmonary emphysema, arterial hypertension, dyslipidemia, depression and thyroid disorders
Tavares [24]	Sub-national	25	Rheumatism, arthritis/arthrosis, osteoporosis, asthma or bronchitis, tuberculosis, embolism, systemic arterial hypertension, poor circulation (varicose veins), heart problems, diabetes mellitus, obesity, stroke, Parkinson's, urinary incontinence, fecal incontinence, intestinal constipation, sleeping problems, cataract, glaucoma, back problems, kidney problem, accident/trauma sequelae, malignant tumors, benign tumors, and vision problems
Wang [25]	Sub-national	16	Cardiovascular diseases (heart attack, heart disease, and stroke), hypertension, diabetes mellitus, arthritis, chronic musculoskeletal pain, headache or migraine, digestive, respiratory (seasonal allergies, asthma, obstructive pulmonary disease, and emphysema), cancer, neurological diseases (Parkinson disease, epilepsy, and multiple sclerosis), anxiety disorder, mood disorder, heavy drinking, impulse-control disorder, substance use disorder, and premenstrual dysphoria
Montes [26]	Sub-national	28	Systemic arterial hypertension, myocardial infarction, diabetes, heart failure, emphysema, asthma, bronchitis, arthritis, Parkinson's disease, renal failure, seizure, hypercholesterolemia, stomach ulcer, osteoporosis, urinary incontinence, constipation, fecal incontinence, depression, glaucoma, deafness, difficulty swallowing, insomnia, fainting, rhinitis, difficulty speaking, stroke, mental disorders and cancer
Padilha Pereira [27]	Sub-national	28	Arterial hypertension, diabetes, heart problems, heart failure, asthma, bronchitis, emphysema, arthritis, Parkinson's disease, kidney failure, hypercholesterolemia, convulsions, stomach ulcer, osteoporosis, urinary incontinence, constipation, fecal

For peer review only

			incontinence, depression, glaucoma, deafness, difficulty in swallowing, insomnia, fainting, rhinitis, difficulty in speaking, stroke, mental disorders, and cancer
Da Silva Almeida [28]	Sub-national	5	Hypertension, diabetes, dyslipidemias, obesity, and arthritis or rheumatism

For peer review only

E-Table 6: Risk of bias of included studies

First author	Representativeness	Sample size	Non-respondents	Exposure	Outcome	Statistical test
Andrade [1]	Low	Low	High	NA	High	NA
Boing [2]	Low	Low	Low	NA	High	NA
De Souza Santos Machado [3]	Low	Low	Low	NA	High	NA
Aguiar [4]	Low	Low	Low	NA	High	NA
Nunes [5]	Low	Low	Low	NA	High	NA
Agrawal [6]	Low	Low	High	NA	High	NA
Nunes [7]	Low	Low	Low	NA	High	NA
Valadares [8]	Low	Low	Low	NA	High	NA
Bustos-Vazquez [9]	Low	Low	Low	NA	High	NA
Cavalcanti [10]	Low	Low	Low	NA	High	NA
Nunes [11]	Low	Low	High	NA	High	NA
Nunes [12]	Low	Low	Low	NA	High	NA
Olivares [13]	Low	Low	Low	NA	Low	NA
Taype-Rondan [14]	Low	Low	Low	NA	Low	NA
Amaral [15]	Low	Low	High	NA	High	NA
Araujo [16]	Low	Low	Low	NA	High	NA
Camargo-Casas [17]	Low	Low	Low	NA	High	NA
Costa [18]	Low	Low	Low	NA	High	NA
Nunes [19]	Low	Low	Low	NA	High	NA
Bao [20]	Low	High	Low	NA	Low	NA
Macinko [21]	Low	High	High	NA	High	NA
Miranda [22]	Low	High	Low	NA	Low	NA
Petarli [23]	Low	Low	Low	NA	High	NA
Tavares [24]	Low	Low	Low	NA	High	NA
Wang [25]	Low	Low	Low	NA	High	NA
Montes [26]	Low	Low	Low	NA	High	NA
Padilha Pereira [27]	Low	Low	Low	NA	High	NA
Da Silva Almeida [28]	Low	Low	Low	NA	Low	NA

For peer review only

REFERENCES

1. Andrade LH, Benseñor IM, Viana MC, Andreoni S, Wang YP. Clustering of psychiatric and somatic illnesses in the general population: multimorbidity and socioeconomic correlates. *Braz j med biol res* 2010;43(5):483-91.
2. Boing AF, Melo GR, Boing AC, Moretti-Pires RO, Peres KG, Peres MA. Associação entre depressão e doenças crônicas: um estudo populacional. *Rev saúde pública* 2012;46(4):617-23.
3. de Souza Santos Machado V, Valadares ALR, da Costa-Paiva LS, Moraes SS, Pinto-Neto AM. Multimorbidity and associated factors in Brazilian women aged 40 to 65 years: a population-based study. *Menopause (New York, NY)* 2012;19(5):569-75.
4. Aguiar LB, Baccaro LF, de Souza Santos Machado V, Pinto-Neto AM, Costa-Paiva L. Disability and multimorbidity in women older than 50 years: a population-based household survey. *Menopause (New York, NY)* 2015;22(6):660-6.
5. Nunes BP, Thume E, Facchini LA. Multimorbidity in older adults: magnitude and challenges for the Brazilian health system. *BMC public health* 2015;15:1172.
6. Agrawal S, Agrawal PK. Association Between Body Mass index and Prevalence of Multimorbidity in Low-and Middle-income Countries: A Cross-Sectional Study. *International journal of medicine and public health* 2016;6(2):73-83.
7. Nunes BP, Camargo-Figuera FA, Guttier M, de Oliveira PD, Munhoz TN, Matijasevich A, et al. Multimorbidity in adults from a southern Brazilian city: occurrence and patterns. *International journal of public health* 2016;61(9):1013-20.
8. Valadares ALR, Lui-Filho JF, Costa-Paiva L, Pinto-Neto AM. Middle-aged female sexual dysfunction and multimorbidity: a population-based study. *Menopause (New York, NY)* 2016;23(3):304-10.
9. Bustos-Vazquez E, Fernandez-Nino JA, Astudillo-Garcia CI. Self-rated health, multimorbidity and depression in Mexican older adults: Proposal and evaluation of a simple conceptual model. *Biomedica : revista del Instituto Nacional de Salud* 2017;37(0):92-103.
10. Cavalcanti G, Doring M, Portella MR, Bortoluzzi EC, Mascarelo A, Dellani MP. Multimorbidity associated with polypharmacy and negative self-perception of health. *Rev bras geriatr gerontol (Online)* 2017;20(5):634-42.
11. Nunes BP, Chiavegatto Filho ADP, Pati S, Cruz Teixeira DS, Flores TR, Camargo-Figuera FA, et al. Contextual and individual inequalities of multimorbidity in Brazilian adults: a cross-sectional national-based study. *BMJ open* 2017;7(6):e015885.
12. Nunes BP, Soares MU, Wachs LS, Volz PM, Saes MdO, Duro SMS, et al. Hospitalization in older adults: association with multimorbidity, primary health care and private health plan. *Rev saúde pública* 2017;51:43-.
13. Olivares DEV, Chambi FRV, Chani EMM, Craig WJ, Pacheco SOS, Pacheco FJ. Risk Factors for Chronic Diseases and Multimorbidity in a Primary Care Context of Central Argentina: A Web-Based Interactive and Cross-Sectional Study. *International journal of environmental research and public health* 2017;14(3).
14. Taype-Rondan A, Abbs ES, Lazo-Porras M, Checkley W, Gilman RH, Smeeth L, et al. Association between chronic conditions and health-related quality of life: differences by level of urbanization in Peru. *Quality of life research : an international journal of quality of life aspects of treatment, care and rehabilitation* 2017;26(12):3439-47.

For peer review only

15. Amaral TLM, Amaral CdA, Lima NSd, Herculano PV, Prado PRd, Monteiro GTR. Multimorbidity, depression and quality of life among elderly people assisted in the Family Health Strategy in Senador Guiomard, Acre, Brazil. *Multimorbidade, depressao e qualidade de vida em idosos atendidos pela Estrategia de Saude da Familia em Senador Guiomard, Acre, Brasil* 2018;23(9):3077-84.
16. Araujo MEA, Silva MT, Galvao TF, Nunes BP, Pereira MG. Prevalence and patterns of multimorbidity in Amazon Region of Brazil and associated determinants: a cross-sectional study. *BMJ open* 2018;8(11):e023398.
17. Camargo-Casas S, Suarez-Monsalve S, Zepeda MUP, Garcia-Pena C, Cano-Gutierrez CA. [Multimorbidity, Depressive Symptoms, and Self-Reported Health in Older Adults: a Secondary Analysis of the Sabe Bogota Study]. *Revista de investigacion clinica; organo del Hospital de Enfermedades de la Nutricion* 2018;70(4):192-7.
18. Costa CdS, Flores TR, Wendt A, Neves RG, Tomasi E, Cesar JA, et al. Inequalities in multimorbidity among elderly: a population-based study in a city in Southern Brazil. *Cad Saúde Pública (Online)* 2018;34(11):e00040718-e.
19. Nunes BP, Batista SRR, Andrade FBd, Souza Junior PRBd, Lima-Costa MF, Facchini LA. Multimorbidity: The Brazilian Longitudinal Study of Aging (ELSI-Brazil). *Revista de saude publica* 2018;52Suppl 2(Suppl 2):10s.
20. Bao J, Chua K-C, Prina M, Prince M. Multimorbidity and care dependence in older adults: a longitudinal analysis of findings from the 10/66 study. *BMC public health* 2019;19(1):585.
21. Macinko J, Andrade FCD, Nunes BP, Guanais FC. Primary care and multimorbidity in six Latin American and Caribbean countries. *Rev panam salud pública* 2019;43:e8-e.
22. Miranda JJ, Bernabe-Ortiz A, Gilman RH, Smeeth L, Malaga G, Wise RA, et al. Multimorbidity at sea level and high-altitude urban and rural settings: The CRONICAS Cohort Study. *Journal of Comorbiddity* 2019;9:1-10.
23. Petarli GB, Cattafesta M, Sant'Anna MM, Bezerra OMdPA, Zandonade E, Salaroli LB. Multimorbidity and complex multimorbidity in Brazilian rural workers. *PLoS ONE* 2019;14(11):e0225416.
24. Tavares DMDS, Pelizaro PB, Pegorari MS, Paiva MMd, Marchiori GF. Prevalence of self-reported morbidities and associated factors among community-dwelling elderly in Uberaba, Minas Gerais, Brazil. *Prevalencia de morbidades autorreferidas e fatores associados entre idosos comunitarios de Uberaba, Minas Gerais, Brasil* 2019;24(9):3305-13.
25. Wang Y-P, Nunes BP, Coelho BM, Santana GL, do Nascimento CF, Viana MC, et al. Multilevel Analysis of the Patterns of Physical-Mental Multimorbidity in General Population of Sao Paulo Metropolitan Area, Brazil. *Scientific reports* 2019;9(1):2390.
26. Montes MC, Bortolotto CC, Tomasi E, Gonzalez MC, Barbosa-Silva TG, Domingues MR, et al. Strength and multimorbidity among community-dwelling elderly from southern Brazil. *Nutrition* 2020;71.
27. Padilha Pereira B, Cardozo Bortolotto C, Tomasi E, Gonzalez MC, Gomes AP, Goncalves H, et al. Food consumption and multimorbidity among non-institutionalized elderly people in Pelotas, 2014: a cross-sectional study. *Epidemiol serv saúde* 2020;29(3):e2019050.
28. da Silva Almeida IL, dos Santos SR, Morbeck de Queiroz B, de Freitas Mussi RF. Lifestyle, morbidity and multimorbidity in adult Quilombolas. *ABCS health sci* 2020;45:1325.

BMJ Open

Multimorbidity in Latin America and the Caribbean: A systematic review and meta-analysis

Journal:	*BMJ Open*
Manuscript ID	bmjopen-2021-050409.R3
Article Type:	Original research
Date Submitted by the Author:	09-Jul-2021
Complete List of Authors:	Huaquía-Díaz, Alessandra; Universidad Científica del Sur<br>Chalán-Dávila, Tarick; Universidad Científica del Sur<br>Carrillo-Larco, Rodrigo; Imperial College London, ; CRONICAS Center of Excellence in Chronic Diseases<br>Bernabe-Ortiz, Antonio; Universidad Peruana Cayetano Heredia, CRONICAS Centre of Excellence in Chronic Diseases; Universidad Cientifica del Sur
**Primary Subject Heading**:	Epidemiology
Secondary Subject Heading:	Global health, Public health
Keywords:	EPIDEMIOLOGY, PUBLIC HEALTH, Hypertension < CARDIOLOGY, DIABETES & ENDOCRINOLOGY

SCHOLARONE™
Manuscripts

I, the Submitting Author has the right to grant and does grant on behalf of all authors of the Work (as defined in the below author licence), an exclusive licence and/or a non-exclusive licence for contributions from authors who are: i) UK Crown employees; ii) where BMJ has agreed a CC-BY licence shall apply, and/or iii) in accordance with the terms applicable for US Federal Government officers or employees acting as part of their official duties; on a worldwide, perpetual, irrevocable, royalty-free basis to BMJ Publishing Group Ltd ("BMJ") its licensees and where the relevant Journal is co-owned by BMJ to the co-owners of the Journal, to publish the Work in this journal and any other BMJ products and to exploit all rights, as set out in our licence.

The Submitting Author accepts and understands that any supply made under these terms is made by BMJ to the Submitting Author unless you are acting as an employee on behalf of your employer or a postgraduate student of an affiliated institution which is paying any applicable article publishing charge ("APC") for Open Access articles. Where the Submitting Author wishes to make the Work available on an Open Access basis (and intends to pay the relevant APC), the terms of reuse of such Open Access shall be governed by a Creative Commons licence – details of these licences and which Creative Commons licence will apply to this Work are set out in our licence referred to above.

Other than as permitted in any relevant BMJ Author's Self Archiving Policies, I confirm this Work has not been accepted for publication elsewhere, is not being considered for publication elsewhere and does not duplicate material already published. I confirm all authors consent to publication of this Work and authorise the granting of this licence.

For peer review only

Multimorbidity in Latin America and the Caribbean: A systematic review and meta-analysis

Alessandra M. Huaquía-Diaz, BS[1]; Tarick S. Chalán-Dávila, BS[1]; Rodrigo M. Carrillo-Larco, MD[2,3,4]; Antonio Bernabé-Ortiz, MD, MPH, PhD[1,2,5]

1. Universidad Científica del Sur, Lima, Peru
2. CRONICAS Center of Excellence in Chronic Diseases, Universidad Peruana Cayetano Heredia, Lima, Peru
3. Department of Epidemiology and Biostatistics, School of Public Health, Imperial College London, London, UK
4. Universidad Católica Los Ángeles de Chimbote, Instituto de Investigación, Chimbote, Peru
5. School of Public Health and Administration, Universidad Peruana Cayetano Heredia, Lima, Peru

Corresponding author:

Antonio Bernabe-Ortiz, MD, MPH, PhD

Universidad Científica del Sur, Lima, Peru

Email: Antonio.Bernabe@upch.pe

For peer review only

ABSTRACT

Objective: To estimate the pooled prevalence of multimorbidity (≥2 non-communicable diseases in the same individual) among adults of the general population of LAC.

Design: Systematic review and meta-analysis.

Data sources: Medline, Embase, Global Health, Scopus, and LILACS up to July 1st, 2020.

Eligibility criteria for selecting studies: The outcome was the prevalence of multimorbidity. Reports were selected whether they enrolled adult individuals (age ≥18 years) from the general population.

Data extraction and synthesis: Reviewers extracted relevant data and assessed risk of bias independently. A random-effects meta-analysis was conducted to report pooled prevalence estimates of multimorbidity; pooled estimates by pre-specified sub-groups (e.g., national studies) was also pursued.

Results: From 5,830 results, we selected 28 reports, mostly from Brazil and 16 were based on a nationally representative sample. From the 28 selected reports, 26 were further included in the meta-analysis revealing a pooled multimorbidity prevalence of 43% (95% CI: 35% - 51%; I^2: 99.9%). When only reports with a nationally representative sample were combined, the pooled prevalence was 37% (95% CI: 27%-47%; I^2: 99.9%). When the ascertainment of multimorbidity was based on self-reports alone, the pooled prevalence was 40% (95% CI: 31% - 48%; I^2: 99.9%); this raised to 52% (95% CI: 33% - 70%; I^2: 99.9%) for reports including self-reported and objective diagnosis.

Conclusions: Our results complement and advance those from global efforts by incorporating much more reports from LAC. We revealed a larger presence of multimorbidity in LAC than previously reported.

Key words: Multimorbidity, systematic review, prevalence, Latin American and the Caribbean

For peer review only

STRENGHTS AND LIMITATIONS OF THIS STUDY

- Most of the analyzed data came from Brazin preventing region representativity.
- High heterogeneity was present in the analysis, mainly due to different conditions included to define multimorbidity.
- Participant's age seems to explain high heterogeneity as age range in studies is very wide.
- A bias due to self-reporting of conditions may underestimate the real burden of multimorbidity in LAC region.

For peer review only

INTRODUCTION

The Academy of Medical Sciences defines multimorbidity as "*the existence of two or more medical chronic conditions in a single individual*" [1]. Subjects with multimorbidity tend to increase healthcare utilization and costs of primary and secondary care services [2]; also, multimorbidity has a subsequent impact on quality of life [3].

In high-income countries, multimorbidity rates are heterogeneous but seem depend on individual's age. Thus, in a cross-sectional study using the dataset of medical practices in Scotland, the prevalence of multimorbidity was 23% using a list of 40 conditions, and was present mainly in older people [4]. Moreover, multimorbidity seems to be increasing in low- and middle-income countries, where data is yet scarce [5]. The increase of life expectancy in Latin American and the Caribbean (LAC) has been associated with greater incidence of noncommunicable conditions (NDC) [6], with the consequent emergence of multimorbidity.

Multimorbidity prevalence has been explored and summarized in some systematic reviews around the world [7, 8] and results ranged from 5% to 98%; nevertheless, their results were mainly informed by data from high-income countries. In a relatively recent systematic review [9], 31 low- and middle-income countries (LMIC) were included with a prevalence of multimorbidity of 30%, compared to an estimate of 38% in high-income countries. However, only nine studies from the LAC region were included. Moreover, pooled estimates by region were not elucidated, preventing to have appropriate indicators of the burden of multimorbidity in this region. A more recent systematic review has reported a pooled prevalence of multimorbidity in LMIC between 3% and 90%, with almost 80% of the studies being from Brazil, China, South Africa, India, Mexico, and Iran [10].

The lack of evidence about multimorbidity may have important consequences for research, public health, and clinical management in LAC region. For example, multimorbidity was not appropriately defined up to 2018; in addition, whether estimates depend upon sex or setting characteristics (i.e., rural vs. urban areas) should be also studied. Moreover, the need of surveillance systems to assess multimorbidity may be elucidated as these estimates have not been estimated in LAC region. Thus, from the public health perspective may not be easy to take appropriate decisions or implement adequate strategies to tackle the problem of multimorbidity.

As a result, we aimed at providing robust evidence about multimorbidity prevalence estimates in LAC region through a systematic review and meta-analysis of population-based surveys. These

For peer review only

results evidence may help to guide interventions and policies so that they can focus on the most pressing frequent multimorbidity phenotypes in LAC.

METHODS

Protocol

This systematic review was registered in PROSPERO under the code CRD42020196177. We aimed to identify the population-based prevalence of multimorbidity in LAC, and to study whether this prevalence varies by multimorbidity definitions, sex, and urban-rural settings.

Eligibility criteria

Reports were selected whether they enrolled adult individuals (age ≥18 years) from the general population. We focused on LAC populations; therefore, we excluded studies with LAC individuals in countries outside the LAC region, and studies with only foreign subjects in LAC nations. Population-based studies were defined as those following a random sampling approach, and such sample was taken from the general population. On the contrary, studies addressing specific populations (e.g., pregnant women), those with individuals with specific conditions (e.g., people with hypertension), or subjects with specific risk factors (e.g., obese or alcohol disorders) were excluded.

The outcome of interest was the prevalence of multimorbidity, defined as the existence of ≥ 2 chronic conditions in the same person [1]. Different definitions of multimorbidity were considered in this review (e.g., ≥2, ≥3, ≥4, or ≥5 conditions) as the current definition (≥2 conditions) is relatively recent. In addition, the presence of chronic conditions could have been measured, self-reported or a combination of these approaches.

Information sources

The search was conducted on January 10, 2020, and then updated on July 1st, 2020. We used Ovid search engine, comprising Medline, Embase and Global Health databases; and in parallel, we also searched Scopus and LILACS. In all of these, searching was carried out without time or language restriction. The search strategy and terms used is detailed in Supplementary Material: e-Table 1-3.

Study selection

Results from each search engine were downloaded and saved in EndNote where duplicates were removed. After that, information was transferred to Rayyan, an open-access online tool for

For peer review only

systematic reviews [11]. Titles and abstracts were reviewed by two researchers in an independent way, and disagreements were solved by a third party. After this screening phase, selected reports were downloaded and independently studied in detail by two researchers, and similarly, discrepancies were solved by a third party. Finally, selected studies were examined again to check for data duplication, i.e., different reports that used the same data (e.g., multiple reports based on the same underlying data). In this case, the paper with more information or the one with the largest sample size was included in the review and meta-analysis.

Data collection

An extraction template form was built by the authors and tested with a random sample of selected studies. After starting data collection, the form was not further modified. This form included study characteristics: study design, country, if it was a nationally representative sample, sample size, year of data collection, age range, age mean, proportion of women, and if it was urban, rural or both. The extraction form also collated the definition of multimorbidity used, self-reported or a combination of self-reported and measured, the number and a list of chronic conditions studies, and prevalence of multimorbidity (overall, by sex, and by rural or urban settings).

Risk of bias of individual studies

Risk of bias of selected studies was evaluated using the Newcastle-Ottawa Quality Assessment Scale adapted for cross-sectional studies as in a previous report [12]. This tool is focused on selection process (representativeness, sample size, and non-respondents), and the assessment of the outcome (independent blind assessment, self-report or not description). The items of this scale were implemented in an Excel spreadsheet and assessed independently by two reviewers; discrepancies were solved by a third party.

Summary measures

Our systematic review followed the Preferred Reporting Items for Systematic Review and Meta-Analysis (PRISMA) toolkit (See Checklist in Supplemental Material: e-Table 4). We presented a qualitative and quantitative summary. The qualitative summary described the characteristics of the study (as listed above), whereas the quantitative summary explored pooled prevalence estimates.

Statistical analyses were performed using Stata version 16 for Windows (StataCorp, College Station, TX, US). The "*metaprop*" command attains a pooled estimate as a weighted average, by fitting a logistic-normal random-effect model without covariates, but random intercepts [13]. After

For peer review only

that, the pooled estimate was calculated using the Freeman-Tukey Arcsine Transformation as suggested in literature [14].

Because the selected studies were different in nature, scope (e.g., national surveys versus community/subnational studies, or urban vs. rural settings) and sample size, we conducted random-effects meta-analysis for comparing estimates in specific subgroups (e.g., national studies only). In addition, stratified meta-analysis (e.g., by sex, and by urban/rural settings) was pursued. Sensitivity analyses were also conducted focused on age by including studies with individuals aged 50+ and 60+ years, and also with studies whose data was collected from 2010 and onwards, as well as 2015 and onwards. Besides, as many of the studies were conducted in Brazil, a comparison of pooled estimated between Brazil and other countries together was also pursued.

Finally, meta-regression was also conducted as a high level of heterogeneity was expected. Meta-regression command in STATA investigates if between-study heterogeneity can be explained by one or more of the variables included in the review.

Patient and Public Involvement

Patients or the public were not involved in the design, or conduct, or reporting, or dissemination plans of our research.

Ethical considerations

The systematic review protocol was reviewed and approved by the Ethical Committee in Universidad Científica del Sur, Lima, Peru.

RESULTS

Selection process

The search strategy yielded 5,830 titles and abstracts after removing duplicates (See Figure 1); of these 66 were studied in detail and 28 reports met the inclusion criteria. For quantitative analyses, 26 reports were included (2 were excluded as the definition of multimorbidity was ≥5 chronic conditions), representing information for 12 LAC countries.

General characteristics of selected studies

As shown in Table 1, Brazil contributes with a total of 20 data points [15-34]; Mexico with 4 data points [23, 35-37]; Peru with 3 data points [36, 38, 39]; Colombia with 2 data points [23, 40]; and Argentina [41], Cuba [36], Dominican Republic [36], El Salvador [23], Jamaica [23], Panama [23],

For peer review only

Puerto Rico [36], and Venezuela [36] with 1 data point each. On the other hand, only 16 studies analyzed a nationally representative sample (Table 1).

Data from 37 country-level points were used to calculate pooled estimates using a definition of multimorbidity of ≥2 chronic conditions. Only one country (Brazil) used an additional definition of multimorbidity (i.e., ≥3 chronic conditions) in 8 different reports. Fourteen reports had information to calculate estimates for urban settings [15, 17-19, 22, 25-27, 29, 33, 35, 38, 39, 42], whereas 7 had information from rural areas [18, 25, 27, 35, 38, 39, 42].

Finally, the number of conditions evaluated to define multimorbidity ranged from 5 to 29, with a mean of 12.3 (SD: 5.7) conditions (Supplemental Material: e-Table 5). Hypertension and type 2 diabetes were the only conditions included in all the definitions of multimorbidity.

Synthesis of results

The meta-analysis included 26 studies with information with a pooled estimate of multimorbidity defined as ≥2 chronic conditions of 43% (95% CI: 35% - 51%; I^2: 99.9%; See Figure 2); whereas the pooled estimate for multimorbidity using ≥3 chronic conditions was 40% (95% CI: 22% - 57%; I^2: 99.9%).

As many of the studies were from Brazil, the pooled estimate of multimorbidity (≥2 chronic conditions) for this country was 50% (95% CI: 37% - 63%; I^2: 99.9%), whereas the pooled estimate for other countries together was 35% (95% CI: 26% - 43%; I^2: 99.7%). Nationally representative samples had a pooled estimate of multimorbidity of 37% (95% CI: 27% - 47%; I^2: 99.9%), whilst this estimate was 48% (95% CI: 35% - 61%; I^2: 99.9%) for non-nationally representative samples. Similarly, when multimorbidity was assessed as self-reported, pooled prevalence was 40% (95% CI: 31% - 48%; I^2: 99.9%), but the prevalence was 52% (95% CI: 33% - 70%; I^2: 99.9%) for those which have objectively measured and self-reported chronic conditions.

On the other hand, when analysis was conducted using studies whose data was collected from 2010 and onwards, pooled prevalence of multimorbidity was 48% (95% CI: 34% - 61%; I^2: 99.9%), whereas this estimate was 44% (95% CI: 24% - 65%, I^2: 99.8%) for studies from 2015 and onwards. This approach was used because time can have an impact on estimations due to the health transition in LMIC. Similarly, when assessing only studies including subjects of 50 years and over, the pooled prevalence of multimorbidity was 62% (95% CI: 51% - 73%; I^2: 99.9%), and there was no variation when the pooled prevalence was estimated for subject of 60 years and over: 62% (95% CI: 49% - 75%; I^2: 99.9%).

For peer review only

In stratified analyses, pooled prevalence of multimorbidity was 38.9% (95% CI: 28.6% - 49.1%; I^2: 99.7%) for males and 50.5% (95% CI: 38.3% - 62.7%; I^2: 99.8%) for females. These estimates were 38.1% (95% CI: 26.1% - 50.1%; I^2: 87.3%) and 24.7% (95% CI: 12.5% - 36.8%; I^2: 50.8%) for urban and rural dwellers, respectively (See Figures in Supplementary Material: e-Figure 1-4). In meta-regression analysis, the number of chronic conditions defining multimorbidity was strongly associated with heterogeneity (β: 0.02 per additional condition, p <0.001). Similarly, mean age was also strongly associated (β: 0.01 per additional year, p <0.001) and the proportion of women involved in the study (β: 0.75, p = 0.008). In addition, setting (urban vs. rural) was almost associated with heterogeneity (β: 0.59 compared to rural settings, p = 0.06).

Risk of bias

All reviewed studies had low risk of bias (Supplementary Material: e-Table 6). Nevertheless, sample size was not justified in three studies [23, 36, 38], and the outcome was self-reported in most of the studies except in 5 of them [21, 36, 38, 39, 41].

DISCUSSION

Summary of evidence

This systematic review provides a comprehensive analysis of the burden of multimorbidity in the LAC region. Accordingly, the pooled prevalence of multimorbidity was 43%. Nevertheless, there was a high heterogeneity among studies included, and results varied by the characteristics of the study as well as some of population characteristics. Thus, the pooled prevalence was lower in nationally representative compared to sub-national samples, whereas estimates were higher when only studies with measured and self-reported chronic conditions were included. In addition, age seems to be an important predictor as the prevalence of multimorbidity among those aged 50 years and over was high compared to the pooled estimate of multimorbidity. Finally, the pooled prevalence of multimorbidity was higher among females compared to males, highlighting the link between sex and multimorbidity, and among urban compared to those rural dwellers highlighting perhaps better access to diagnostic care in urban sites.

Limitations of the review

There are some limitations in this review that should be highlighted. First, high heterogeneity is present in almost all the results. This finding may be attributable to age group inclusion criteria since a great proportion of studies enrolled individuals aged ≥50+ years and the pooled estimate

For peer review only

of multimorbidity was high in this group. In addition, the proportion of women enrolled in each individual study may be relevant as pointed out by the meta-regression analyses. Second, the number of chronic conditions as well as the list of them used to define multimorbidity is very dissimilar. Defining specific clusters of multimorbidity is needed to guarantee comparability between studies, but this is not usually reported. Therefore, it is relevant to standardize the definition of multimorbidity and the conditions included in such definition to estimate which clusters of multimorbidity are more frequent and relevant for LAC region. In addition to that, before recent definition of multimorbidity, some reports used other different definitions (≥ 3 or ≥ 5 chronic conditions); which could affect pooled results. Fortunately, analysis was possible to include only those with ≥2 chronic conditions. Third, most of the studies included in the review were from Brazil, preventing inferability to the whole region, but also highlighting the need of population-based studies on multimorbidity in other countries of the region. Of note, the pooled prevalence of multimorbidity was higher in Brazil compared to other countries, perhaps because Brazilian researchers have addressed a common definition of multimorbidity, using a list of 12 conditions. Finally, a bias due to self-reporting can affect our results. Therefore, whether multimorbidity is defined by self-report or by more objective measurements may have an impact on prevalence estimates. Awareness of some chronic conditions are usually low and varying; for example, hypertension and diabetes awareness is around 64% and 78%, respectively in urban areas of Latin America [43], but tend to be lower in rural settings [44]. Thus, our results may be underestimating the real burden of multimorbidity in the LAC region.

Public Health Relevance

Global trends suggest that multimorbidity is a public health challenge; thus, understanding the epidemiology of multimorbidity in LAC region may be relevant as this issue has received little attention from researchers, but especially, policymakers. Moreover, much of the response of the health system has been developed based on one specific condition or specific body system, instead of an integral approach whereby multiple conditions are addressed synergistically. Therefore, disperse and heterogeneous information have been available for this systematic review.

Our results highlight the need of implementing a surveillance system focused on multimorbidity. This can be done by including some specific conditions in routine health surveys (Demographic Health Surveys or similar) and other population-based research studies. In addition, these surveys

For peer review only

may include the most common clusters of multimorbidity, and those with higher morbidity and mortality [45] , however, such relevant clusters need to be appropriately defined.

Our results also imply that health systems need to be adapted to face the challenge of multimorbidity which increases healthcare use and costs related to primary and secondary prevention [2]. This adaptation process includes the appropriate training of human resources as well as improving of health services infrastructure and care delivery. It is also needed to develop guidelines for multimorbidity care as that of NICE that includes clinical assessment and adequate management [46], but also highlight related issues as polypharmacy and life expectancy. Therefore, a holistic approach may be needed to tackle this global health problem.

Conclusions

Our systematic review shows that four of ten participants have multimorbidity at the population level in LAC. There is, however, a marked variability, depending on participant's age and the number of chronic conditions assessed, highlighting the need of better designed and standardized studies to inform the landscape of multimorbidity in LAC.

Statements

Contributorship statement: TSC-D, AMH-D and AB-O conceived the idea of the systematic review and manuscript. TSC-D and AMH-D conducted the review with support of AB-O and RMC-L. RMC-L and AB-O conducted the statistical analysis. TSC-D and AMH-D drafted the first version of the manuscript, with relevant comments and suggestions of RMC-L and AB-O. All the authors approved the version submitted for publication.

Competing interests: None declared.

Funding: Rodrigo M Carrillo-Larco is supported by a Wellcome Trust International Training Fellowship (214185/Z/18/Z).

Data sharing statement: All the information of the present systematic review is included in the manuscript. There is no additional data available.

REFERENCES

1. The Academy of Medical Sciences. *Multimorbidity: a priority for health research*. London, UK: Academy of Medical Sciences; 2018.

For peer review only

2. Soley-Bori M, Ashworth M, Bisquera A, Dodhia H, Lynch R, Wang Y, et al. Impact of multimorbidity on healthcare costs and utilisation: a systematic review of the UK literature. *Br J Gen Pract* 2021;71(702):e39-e46.
3. Makovski TT, Schmitz S, Zeegers MP, Stranges S, van den Akker M. Multimorbidity and quality of life: Systematic literature review and meta-analysis. *Ageing Res Rev* 2019;53:100903.
4. Barnett K, Mercer SW, Norbury M, Watt G, Wyke S, Guthrie B. Epidemiology of multimorbidity and implications for health care, research, and medical education: a cross-sectional study. *Lancet* 2012;380(9836):37-43.
5. Banerjee A, Hurst J, Fottrell E, Miranda JJ. Multimorbidity: Not Just for the West. *Glob Heart* 2020;15(1):45.
6. Bilal U, Hessel P, Perez-Ferrer C, Michael YL, Alfaro T, Tenorio-Mucha J, et al. Life expectancy and mortality in 363 cities of Latin America. *Nat Med* 2021.
7. Violan C, Foguet-Boreu Q, Flores-Mateo G, Salisbury C, Blom J, Freitag M, et al. Prevalence, determinants and patterns of multimorbidity in primary care: a systematic review of observational studies. *PLoS ONE* 2014;9(7):e102149.
8. Pati S, Swain S, Hussain MA, van den Akker M, Metsemakers J, Knottnerus JA, et al. Prevalence and outcomes of multimorbidity in South Asia: a systematic review. *BMJ open* 2015;5(10):e007235.
9. Nguyen H, Manolova G, Daskalopoulou C, Vitoratou S, Prince M, Prina AM. Prevalence of multimorbidity in community settings: A systematic review and meta-analysis of observational studies. *J Comorb* 2019;9:2235042x19870934.
10. Abebe F, Schneider M, Asrat B, Ambaw F. Multimorbidity of chronic non-communicable diseases in low- and middle-income countries: A scoping review. *J Comorb* 2020;10:2235042x20961919.
11. Ouzzani M, Hammady H, Fedorowicz Z, Elmagarmid A. Rayyan-a web and mobile app for systematic reviews. *Syst Rev* 2016;5(1):210.
12. Modesti PA, Reboldi G, Cappuccio FP, Agyemang C, Remuzzi G, Rapi S, et al. Panethnic Differences in Blood Pressure in Europe: A Systematic Review and Meta-Analysis. *PLoS ONE* 2016;11(1):e0147601.

For peer review only

13. Nyaga VN, Arbyn M, Aerts M. Metaprop: a Stata command to perform meta-analysis of binomial data. *Arch Public Health* 2014;72(1):39.
14. Freeman MF, Tukey JW. Transformations related to the angular and the square root. *Ann Math Stat* 1950;21:607-11.
15. Aguiar LB, Baccaro LF, de Souza Santos Machado V, Pinto-Neto AM, Costa-Paiva L. Disability and multimorbidity in women older than 50 years: a population-based household survey. *Menopause (New York, NY)* 2015;22(6):660-6.
16. Amaral TLM, Amaral CdA, Lima NSd, Herculano PV, Prado PRd, Monteiro GTR. Multimorbidity, depression and quality of life among elderly people assisted in the Family Health Strategy in Senador Guiomard, Acre, Brazil. *Multimorbidade, depressao e qualidade de vida em idosos atendidos pela Estrategia de Saude da Familia em Senador Guiomard, Acre, Brasil* 2018;23(9):3077-84.
17. Andrade LH, Benseñor IM, Viana MC, Andreoni S, Wang YP. Clustering of psychiatric and somatic illnesses in the general population: multimorbidity and socioeconomic correlates. *Braz j med biol res* 2010;43(5):483-91.
18. Araujo MEA, Silva MT, Galvao TF, Nunes BP, Pereira MG. Prevalence and patterns of multimorbidity in Amazon Region of Brazil and associated determinants: a cross-sectional study. *BMJ open* 2018;8(11):e023398.
19. Boing AF, Melo GR, Boing AC, Moretti-Pires RO, Peres KG, Peres MA. Associação entre depressão e doenças crônicas: um estudo populacional. *Rev saúde pública* 2012;46(4):617-23.
20. Costa CdS, Flores TR, Wendt A, Neves RG, Tomasi E, Cesar JA, et al. Inequalities in multimorbidity among elderly: a population-based study in a city in Southern Brazil. *Cad Saúde Pública (Online)* 2018;34(11):e00040718-e.
21. da Silva Almeida IL, dos Santos SR, Morbeck de Queiroz B, de Freitas Mussi RF. Lifestyle, morbidity and multimorbidity in adult Quilombolas. *ABCS health sci* 2020;45:1325.
22. de Souza Santos Machado V, Valadares ALR, da Costa-Paiva LS, Moraes SS, Pinto-Neto AM. Multimorbidity and associated factors in Brazilian women aged 40 to 65 years: a population-based study. *Menopause (New York, NY)* 2012;19(5):569-75.
23. Macinko J, Andrade FCD, Nunes BP, Guanais FC. Primary care and multimorbidity in six Latin American and Caribbean countries. *Rev panam salud pública* 2019;43:e8-e.

For peer review only

24. Montes MC, Bortolotto CC, Tomasi E, Gonzalez MC, Barbosa-Silva TG, Domingues MR, et al. Strength and multimorbidity among community-dwelling elderly from southern Brazil. *Nutrition* 2020;71.

25. Nunes BP, Batista SRR, Andrade FBd, Souza Junior PRBd, Lima-Costa MF, Facchini LA. Multimorbidity: The Brazilian Longitudinal Study of Aging (ELSI-Brazil). *Revista de saude publica* 2018;52Suppl 2(Suppl 2):10s.

26. Nunes BP, Camargo-Figuera FA, Guttier M, de Oliveira PD, Munhoz TN, Matijasevich A, et al. Multimorbidity in adults from a southern Brazilian city: occurrence and patterns. *International journal of public health* 2016;61(9):1013-20.

27. Nunes BP, Chiavegatto Filho ADP, Pati S, Cruz Teixeira DS, Flores TR, Camargo-Figuera FA, et al. Contextual and individual inequalities of multimorbidity in Brazilian adults: a cross-sectional national-based study. *BMJ open* 2017;7(6):e015885.

28. Nunes BP, Soares MU, Wachs LS, Volz PM, Saes MdO, Duro SMS, et al. Hospitalization in older adults: association with multimorbidity, primary health care and private health plan. *Rev saúde pública* 2017;51:43-.

29. Nunes BP, Thume E, Facchini LA. Multimorbidity in older adults: magnitude and challenges for the Brazilian health system. *BMC public health* 2015;15:1172.

30. Padilha Pereira B, Cardozo Bortolotto C, Tomasi E, Gonzalez MC, Gomes AP, Goncalves H, et al. Food consumption and multimorbidity among non-institutionalized elderly people in Pelotas, 2014: a cross-sectional study. *Epidemiol serv saúde* 2020;29(3):e2019050.

31. Petarli GB, Cattafesta M, Sant'Anna MM, Bezerra OMdPA, Zandonade E, Salaroli LB. Multimorbidity and complex multimorbidity in Brazilian rural workers. *PLoS ONE* 2019;14(11):e0225416.

32. Tavares DMDS, Pelizaro PB, Pegorari MS, Paiva MMd, Marchiori GF. Prevalence of self-reported morbidities and associated factors among community-dwelling elderly in Uberaba, Minas Gerais, Brazil. *Prevalencia de morbidades autorreferidas e fatores associados entre idosos comunitarios de Uberaba, Minas Gerais, Brasil* 2019;24(9):3305-13.

33. Valadares ALR, Lui-Filho JF, Costa-Paiva L, Pinto-Neto AM. Middle-aged female sexual dysfunction and multimorbidity: a population-based study. *Menopause (New York, NY)* 2016;23(3):304-10.

For peer review only

34. Wang Y-P, Nunes BP, Coelho BM, Santana GL, do Nascimento CF, Viana MC, et al. Multilevel Analysis of the Patterns of Physical-Mental Multimorbidity in General Population of Sao Paulo Metropolitan Area, Brazil. *Scientific reports* 2019;9(1):2390.
35. Agrawal S, Agrawal PK. Association Between Body Mass index and Prevalence of Multimorbidity in Low-and Middle-income Countries: A Cross-Sectional Study. *International journal of medicine and public health* 2016;6(2):73-83.
36. Bao J, Chua K-C, Prina M, Prince M. Multimorbidity and care dependence in older adults: a longitudinal analysis of findings from the 10/66 study. *BMC public health* 2019;19(1):585.
37. Bustos-Vazquez E, Fernandez-Nino JA, Astudillo-Garcia CI. Self-rated health, multimorbidity and depression in Mexican older adults: Proposal and evaluation of a simple conceptual model. *Biomedica : revista del Instituto Nacional de Salud* 2017;37(0):92-103.
38. Miranda JJ, Bernabe-Ortiz A, Gilman RH, Smeeth L, Malaga G, Wise RA, et al. Multimorbidity at sea level and high-altitude urban and rural settings: The CRONICAS Cohort Study. *Journal of Comorbiddity* 2019;9:1-10.
39. Taype-Rondan A, Abbs ES, Lazo-Porras M, Checkley W, Gilman RH, Smeeth L, et al. Association between chronic conditions and health-related quality of life: differences by level of urbanization in Peru. *Quality of life research : an international journal of quality of life aspects of treatment, care and rehabilitation* 2017;26(12):3439-47.
40. Camargo-Casas S, Suarez-Monsalve S, Zepeda MUP, Garcia-Pena C, Cano-Gutierrez CA. [Multimorbidity, Depressive Symptoms, and Self-Reported Health in Older Adults: a Secondary Analysis of the Sabe Bogota Study]. *Revista de investigacion clinica; organo del Hospital de Enfermedades de la Nutricion* 2018;70(4):192-7.
41. Olivares DEV, Chambi FRV, Chani EMM, Craig WJ, Pacheco SOS, Pacheco FJ. Risk Factors for Chronic Diseases and Multimorbidity in a Primary Care Context of Central Argentina: A Web-Based Interactive and Cross-Sectional Study. *International journal of environmental research and public health* 2017;14(3).
42. Cavalcanti G, Doring M, Portella MR, Bortoluzzi EC, Mascarelo A, Dellani MP. Multimorbidity associated with polypharmacy and negative self-perception of health. *Rev bras geriatr gerontol (Online)* 2017;20(5):634-42.

For peer review only

43. Silva H, Hernandez-Hernandez R, Vinueza R, Velasco M, Boissonnet CP, Escobedo J, et al. Cardiovascular risk awareness, treatment, and control in urban Latin America. *Am J Ther* 2010;17(2):159-66.

44. Lerner AG, Bernabe-Ortiz A, Gilman RH, Smeeth L, Miranda JJ. The "rule of halves" does not apply in Peru: awareness, treatment, and control of hypertension and diabetes in rural, urban, and rural-to-urban migrants. *Crit Pathw Cardiol* 2013;12(2):53-8.

45. Whitty CJM, Watt FM. Map clusters of diseases to tackle multimorbidity. *Nature* 2020;579(7800):494-6.

46. National Institute for Health and Care Excellence (NICE). Multimorbidity: clinical assessment and management: NICE guideline [NG56]. NICE; 2016 [updated 2016; cited 2021 January 30]; Available from: https://www.nice.org.uk/guidance/ng56.

For peer review only

TABLES AND FIGURES

Figure 1: Flowchart of studies included in the systematic review

For peer review only

Figure 2: Forest plot of the pooled prevalence of multimorbidity defined as ≥2 chronic conditions

For peer review only

Table 1: Characteristics of the studies included in the systematic review

First author	Country	Study design	Data collected in	Sample size	Definition of multimorbidity	Age range	Age mean	% women	% urban
Andrade [17]	Brazil	Cross-sectional	1994-1995	1,464	≥2 (self-reported)	18+	--	57.4%	100%
Boing [19]	Brazil	Cross-sectional	2009-2010	1,720	≥2 (self-reported)	20 - 59	--	55.6%	100%
De Souza Santos Machado [22]	Brazil	Cross-sectional	2005	377	≥2 (self-reported)	40 - 65	--	100%	100%
Aguiar [15]	Brazil	Cross-sectional	2011	622	≥2 (self-reported)	50+	64.1	100%	100%
Nunes [29]	Brazil	Cross-sectional	2008	1,593	≥2 and ≥3 (self-reported)	60+	--	62.8%	100%
Agrawal [35]	Mexico	Cross-sectional	2007-2010	2,725	≥2 (self-reported)	18+	63.1	61.8%	73.7%
Nunes [26]	Brazil	Cross-sectional	2012	2,927	≥2 and ≥3 (self-reported)	20+	45.7	58.9%	100%
Valadares [33]	Brazil	Cross-sectional	2012-2013	736	≥2 (self-reported)	45 - 60	52.5	100%	100%
Bustos-Vazquez [37]	Mexico	Cross-sectional	2012	7,967	≥2 (self-reported)	60+	69.3	53.4%	--
Cavalcanti [42]	Brazil	Cross-sectional	2010-2011	676	≥2 (self-reported)	60+	70.0	54.6%	69.4%
Nunes [27]	Brazil	Cross-sectional	2013	60,202	≥2 and ≥3 (self-reported)	18+	43.7	55.1%	86.5%
Nunes [28]	Brazil	Cross-sectional	2008	1,593	≥2 and ≥3 (self-reported)	60+	--	62.8%	--
Olivares [41]	Argentina	Cross-sectional	2014-2015	1,044	≥2 (self-reported)	18+	43.0	65%	--

For peer review only

Taype-Rondan [39]	Peru	Longitudinal	2013-2014	2,433	≥2 (self-reported and measured)	35+	57.2	51.3%	51.3%
Amaral [16]	Brazil	Cross-sectional	2010	264	≥2 (self-reported)	60+	--	61%	100%
Araujo [18]	Brazil	Cross-sectional	2015	4,001	≥2 and ≥3 (self-reported)	18+	--	52.7%	86.8%
Camargo-Casas [40]	Colombia	Cross-sectional	2012	2,000	≥2 (self-reported)	60+	71.1	63.4%	--
Costa [20]	Brazil	Cross-sectional	2014	1,451	≥2 and ≥3 (self-reported and measured)	60+	--	63%	--
Nunes [25]	Brazil	Longitudinal	2015-2016	9,412	≥2 and ≥3 (self-reported)	50+	62.9	54%	84.7%
Bao [36]	Cuba	Longitudinal	2003-2007	2,944	≥2 (self-reported and measured)	65+	73.9	64%	--
	Dominican Republic			2,011			74.5	65%	--
	Puerto Rico			2,009			75.2	67%	--
	Venezuela			1,965			71.5	63%	--
	Peru			1,933			74.2	60%	--
	Mexico			2,003			73.7	64%	--
Macinko [23]	Brazil	Cross-sectional	2013-2014	1,486	≥2 (self-reported)	18+	--	51.1%	--
	Colombia			1,485			--	51.1%	--
	El Salvador			1,460			--	52.8%	--
	Jamaica			1,480			--	52.6%	--
	Mexico			1,492			--	51.4%	--
	Panama			1,475			--	51.6%	--
Miranda [38]	Peru	Longitudinal	2010-2012	2,890	≥2 (self-reported and measured)	35+	55.2	51%	50.9%
Petarli [31]	Brazil	Cross-sectional	2016-2017	790	≥2 and ≥3 (self-reported)	18 - 59	--	47.7%	--
Tavares [32]	Brazil	Cross-sectional	2012	1,691	≥2 (self-reported)	60+	72.5	63.7%	100%

For peer review only

Wang [34]	Brazil	Cross-sectional	2005-2007	2,713	≥2 (self-reported)	18 - 64	--	52,4%	--
Montes [24]	Brazil	Cross-sectional	2014	1,336	≥5 (self-reported)	60+	--	63.1%	--
Padilha Pereira [30]	Brazil	Cross-sectional	2014	1,426	≥5 (self-reported)	60+	--	63%	--
Da Silva Almeida [21]	Brazil	Cross-sectional	2016	850	≥2 (self-reported and measured)	18+	--	61.2%	--

For peer review only

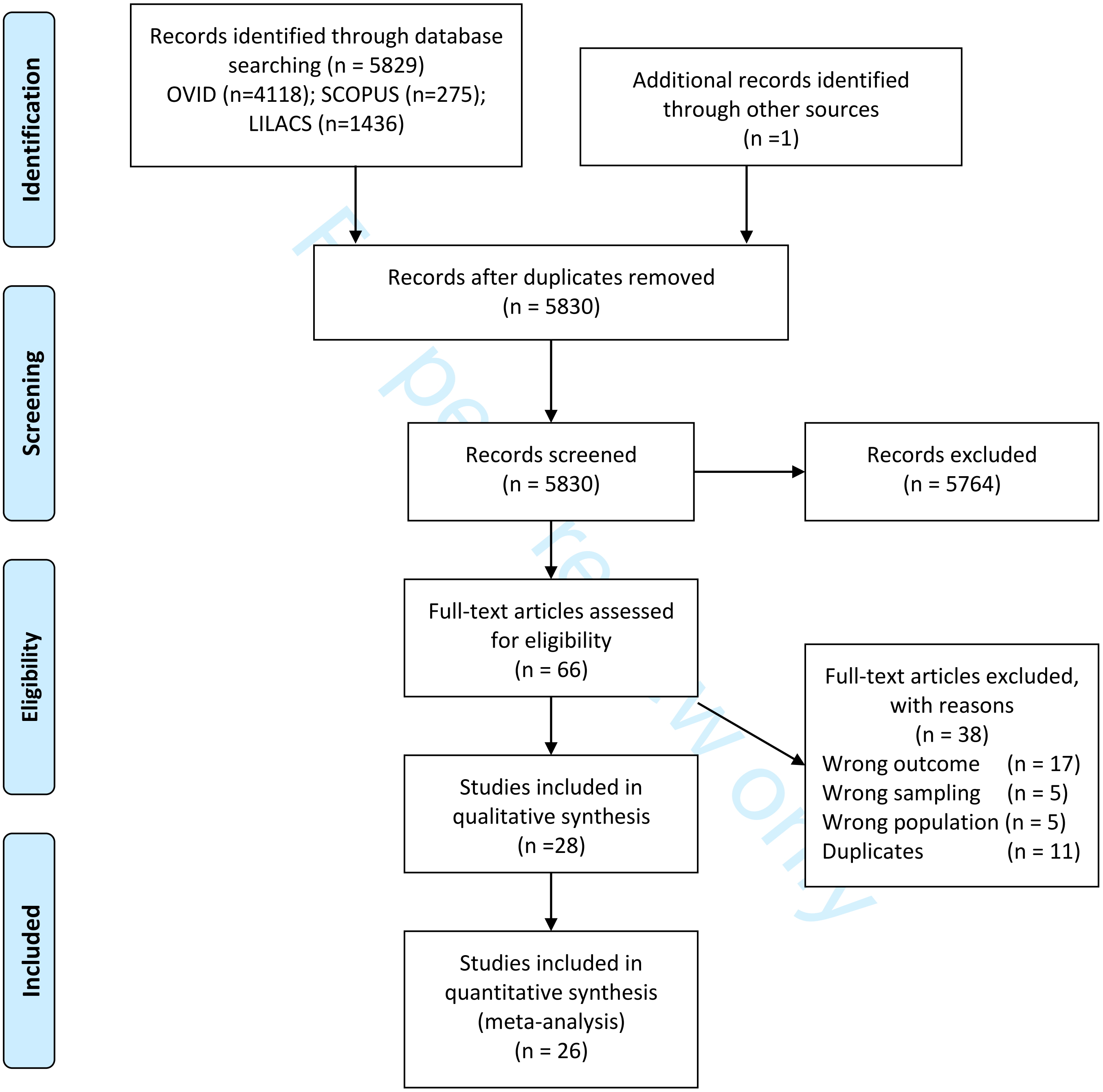

For peer review only - http://bmjopen.bmj.com/site/about/guidelines.xhtml

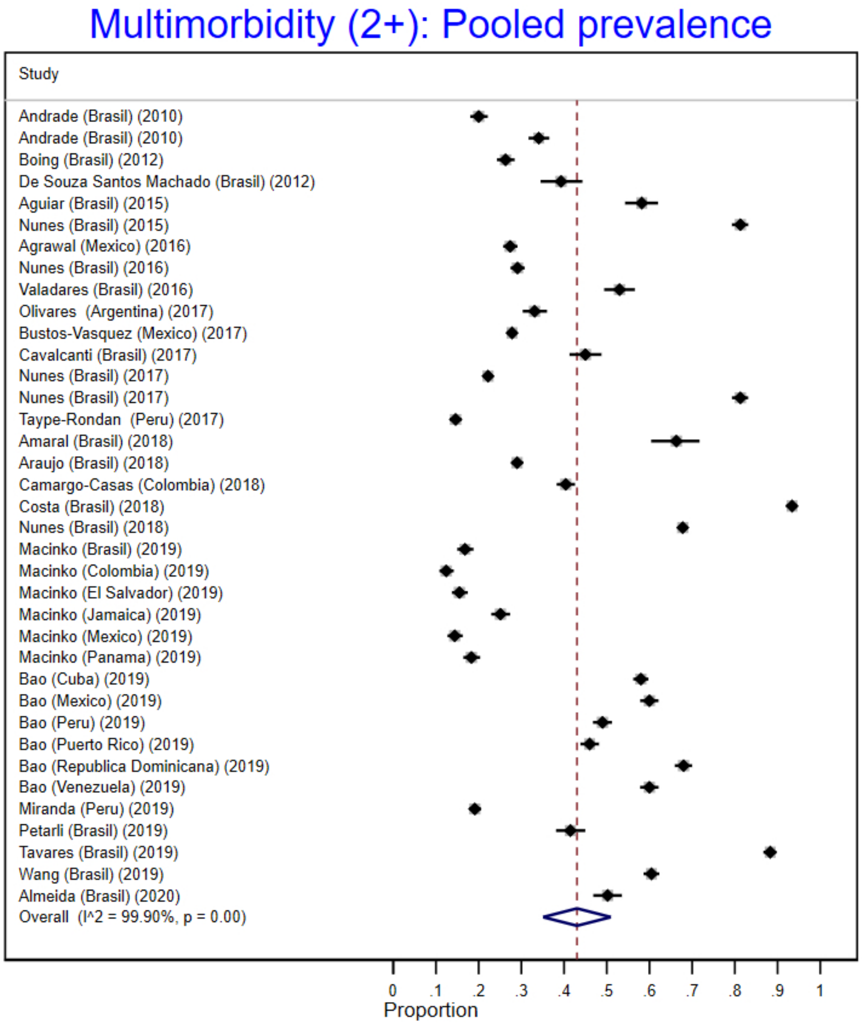

Figure 2: Forest plot of the pooled prevalence of multimorbidity defined as ≥2 chronic conditions

291x319mm (72 x 72 DPI)

SUPPLEMENTAL MATERIAL

E-Figure 1: Forest plot with pooled estimates of multimorbidity among males

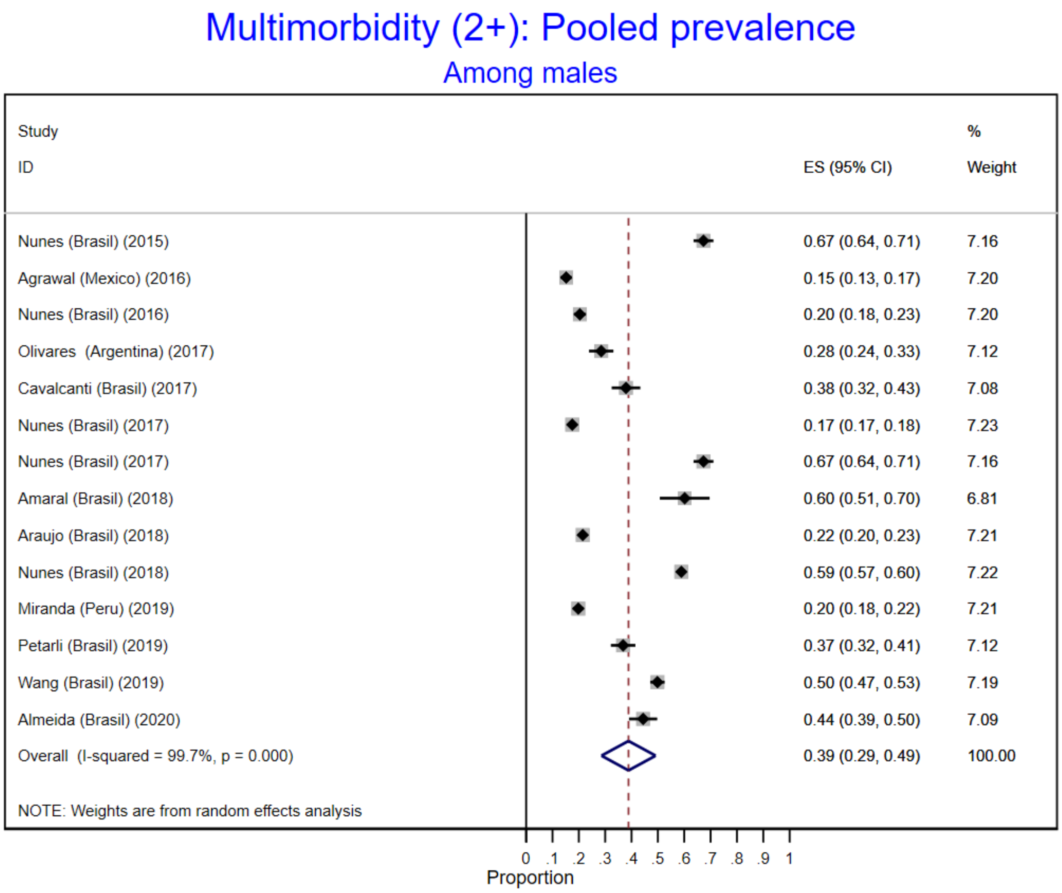

E-Figure 2: Forest plot with pooled estimates of multimorbidity among females

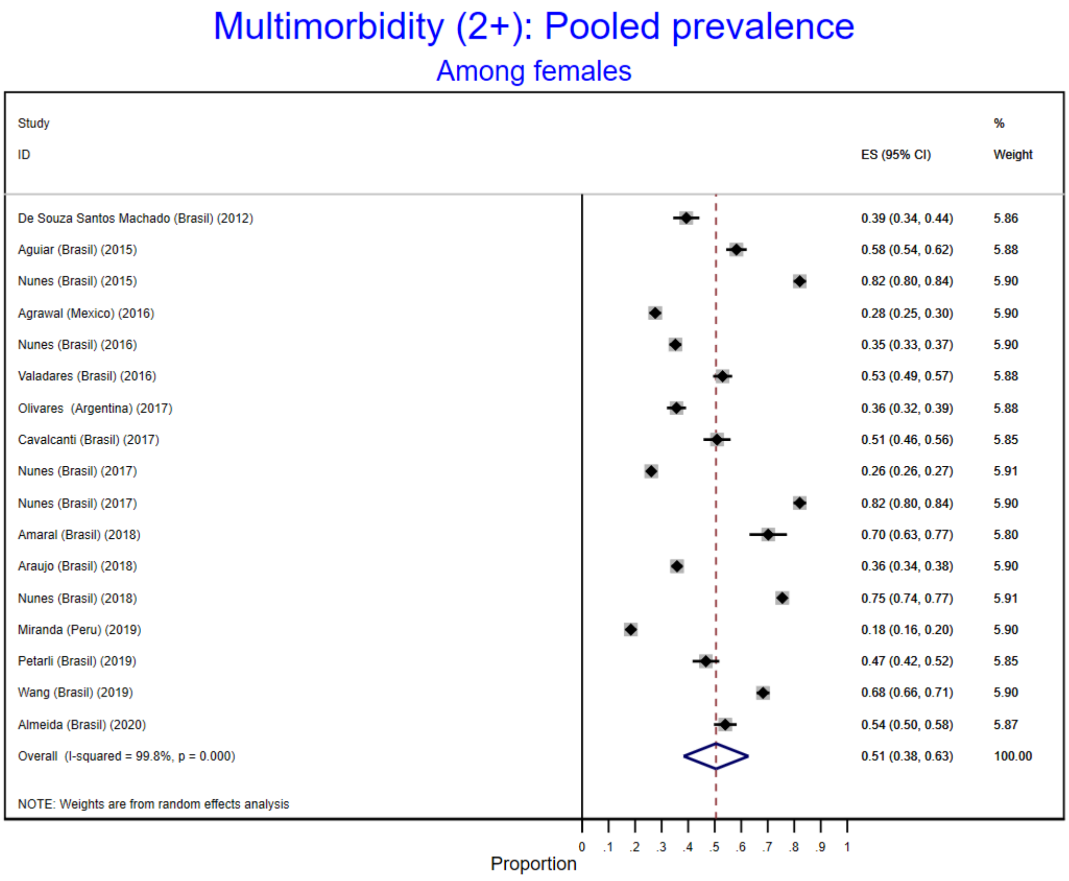

E-Figure 3: Forest plot with pooled estimates of multimorbidity among urban dwellers

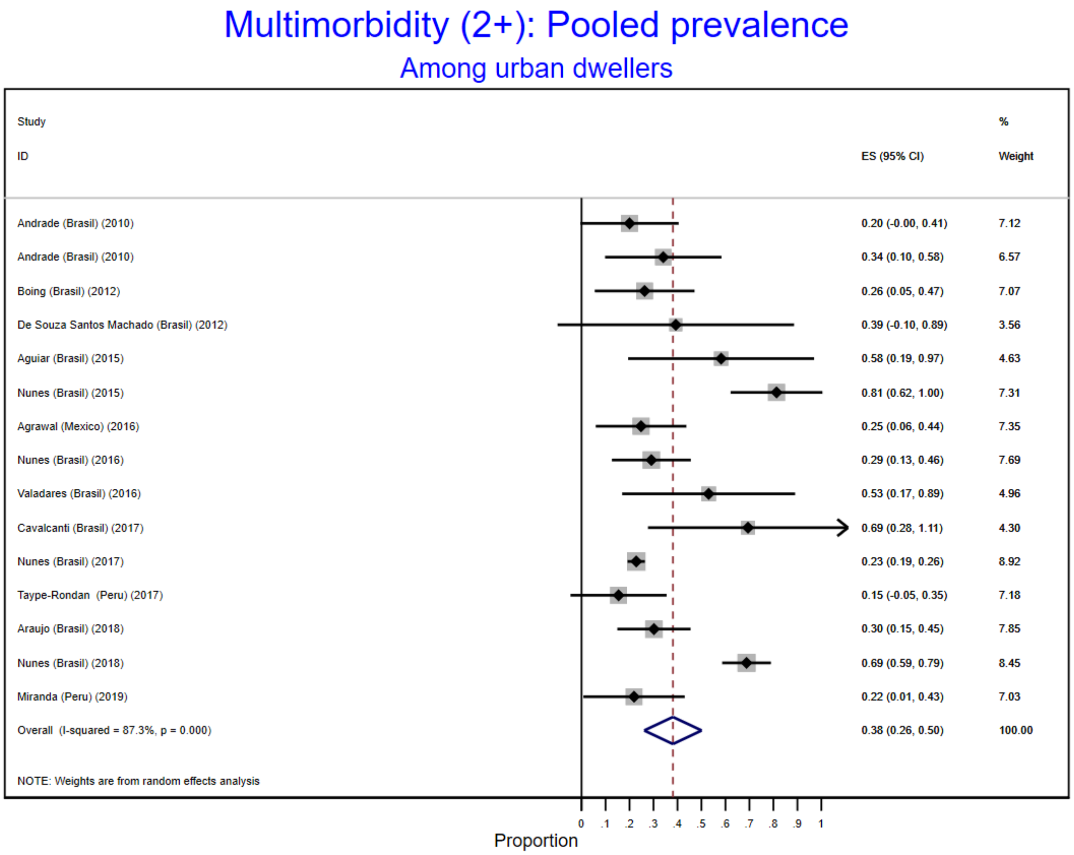

E-Figure 4: Forest plot with pooled estimates of multimorbidity among rural dwellers

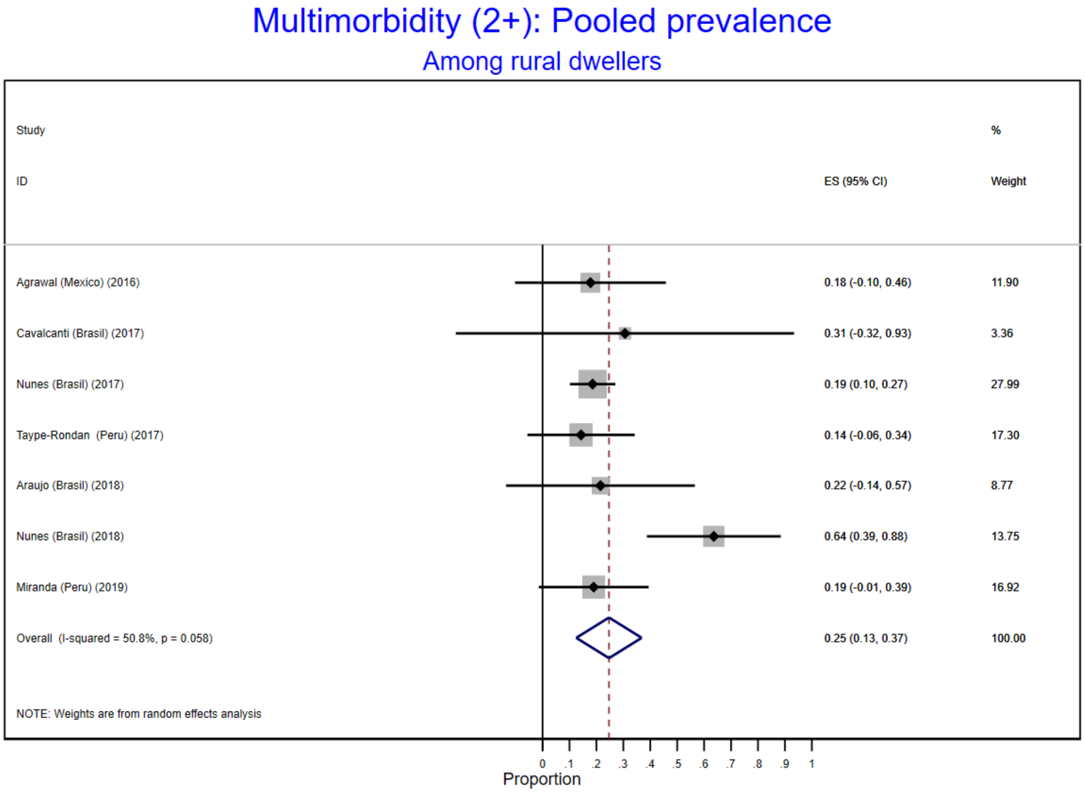

nly

E-Table 1: Search terms and strategy using OVID (Embase, Medline and Global Health)

1	multimorbidit*.mp
2	multi-morbidit*.mp
3	(multi adj morbidit*).mp
4	multiple morbidit*.mp
5	(co adj morbidit*).mp
6	comorbidit*.mp
7	“multiple chronic conditions”.mp
8	“multiple chronic illnesses”.mp
9	“multiple chronic diseases”.mp
10	(poly adj morbidit*).mp
11	polymorbidit*.mp
12	poly-morbidit*.mp
13	1 or 2 or 3 or 4 or 5 or 6 or 7 or 8 or 9 or 10 or 11 or 12
14	(("Antigua and Barbuda") or ("Argentina") or ("Bahamas") or ("Barbados") or ("Belize") or ("Bolivia") or ("Brazil") or ("United States Virgin Islands") or ("British Virgin Islands") or ("Chile") or ("Colombia") or ("Costa Rica") or ("Cuba") or ("Dominica") or ("Dominican Republic") or ("Ecuador") or ("El Salvador") or ("Grenada") or ("Guatemala") or ("Guyana") or ("Haiti") or ("Honduras") or ("Jamaica") or ("Mexico") or ("Nicaragua") or ("Panama") or ("Paraguay") or ("Peru") or ("Puerto Rico") or ("Saint Kitts and Nevis") or ("Saint Lucia") or ("Saint Vincent and the Grenadines") or ("Suriname") or ("Trinidad and Tobago") or ("West Indies") or ("Uruguay") or ("Venezuela") or ("Latin America") or latin amer$ or ("South America") or south amer$ or ("Central America") or central amer$ or ("Caribbean Region"))
15	13 and 14
16	exp animals/ not humans.sh.
17	15 not 16
18	remove duplicates from 17

For peer review only

E-Table 2: Search terms and strategy using Scopus

(TITLE-ABS-KEY(multimorbidity$) OR TITLE-ABS-KEY(multi-morbidit$) OR TITLE-ABS-KEY(multi-morbidit$) OR TITLE-ABS-KEY(multi Pre/1 morbidit$) OR TITLE-ABS-KEY(multiple morbidit$) OR TITLE-ABS-KEY(co Pre/1 morbidit$) OR TITLE-ABS-KEY(comorbidit$) OR TITLE-ABS-KEY(multiple chronic conditions) OR TITLE-ABS-KEY(multiple chronic illnesses) OR TITLE-ABS-KEY(multiple chronic diseases) OR TITLE-ABS-KEY(poly Pre/1 morbidit$) OR TITLE-ABS-KEY(polymorbidit$) OR TITLE-ABS-KEY(poly-morbidit$)) AND (TITLE-ABS-KEY("Antigua and Barbuda") or TITLE-ABS-KEY("Argentina") or TITLE-ABS-KEY("Bahamas") or TITLE-ABS-KEY("Barbados") or TITLE-ABS-KEY("Belize") or TITLE-ABS-KEY("Bolivia") or TITLE-ABS-KEY("Brazil") or TITLE-ABS-KEY("United States Virgin Islands") or TITLE-ABS-KEY("British Virgin Islands") or TITLE-ABS-KEY("Chile") or TITLE-ABS-KEY("Colombia") or TITLE-ABS-KEY("Costa Rica") or TITLE-ABS-KEY("Cuba") or TITLE-ABS-KEY("Dominica") or TITLE-ABS-KEY("Dominican Republic") or TITLE-ABS-KEY("Ecuador") or TITLE-ABS-KEY("El Salvador") or TITLE-ABS-KEY("Grenada") or TITLE-ABS-KEY("Guatemala") or TITLE-ABS-KEY("Guyana") or TITLE-ABS-KEY("Haiti") or TITLE-ABS-KEY("Honduras") or TITLE-ABS-KEY("Jamaica") or TITLE-ABS-KEY("Mexico") or TITLE-ABS-KEY("Nicaragua") or TITLE-ABS-KEY("Panama") or TITLE-ABS-KEY("Paraguay") or TITLE-ABS-KEY("Peru") or TITLE-ABS-KEY("Puerto Rico") or TITLE-ABS-KEY("Saint Kitts and Nevis") or TITLE-ABS-KEY("Saint Lucia") or TITLE-ABS-KEY("Saint Vincent and the Grenadines") or TITLE-ABS-KEY("Suriname") or TITLE-ABS-KEY("Trinidad and Tobago") or TITLE-ABS-KEY("West Indies") or TITLE-ABS-KEY("Uruguay") or TITLE-ABS-KEY("Venezuela") or TITLE-ABS-KEY("Latin America") or TITLE-ABS-KEY(latin amer$) or TITLE-ABS-KEY("South America") or TITLE-ABS-KEY(south amer$) or TITLE-ABS-KEY("Central America") or TITLE-ABS-KEY(central amer$) or TITLE-ABS-KEY("Caribbean Region")) AND NOT DBCOLL(medl)

E-Table 3: Search terms and strategy using LILACS

((multi-morbilidad) OR (multimorbilidad) OR (co-morbilidad) OR (comorbilidad) OR (multiple morbilidad) OR (multiples condiciones cronicas) OR (multiples enfermedades cronicas) OR (multiples patologias cronicas) OR (poli-morbilidad) OR (polimorbilidad)) AND (("Antigua y Barbuda") OR ("Argentina") OR ("Aruba") OR ("Bahamas") OR ("Barbados") OR ("Belice") OR ("Bolivia") OR ("Brasil") OR ("Islas Vírgenes de los Estados Unidos") OR ("Islas Vírgenes Británicas") OR ("Islas Caimán") OR ("Chile") OR ("Colombia") OR ("Costa Rica") OR ("Cuba") OR ("Curazao") OR ("Dominica") OR ("Republica Dominicana") OR ("Ecuador") OR ("El Salvador") OR ("Granada") OR ("Guatemala") OR ("Guyana") OR ("Haití") OR ("Honduras") OR ("Jamaica") OR ("México") OR ("Nicaragua") OR ("Panamá") OR ("Paraguay") OR ("Perú") OR ("Puerto Rico") OR ("San Cristóbal y Nieves ") OR ("Santa Lucía") OR ("San Vicente y las Granadinas ") OR ("Surinam") OR ("Trinidad y Tobago") OR ("Turcas y Caicos ") OR ("Uruguay") OR ("Venezuela") OR ("América Latina") OR ("Latinoamérica") OR ("América del Sur") OR ("Sudamérica") OR ("Suramérica") OR ("América Central") OR ("Centroamérica") OR ("América del Centro") OR ("Caribe"))

E-Table 4: PRISMA Checklist

Section/topic	#	Checklist item	Reported on page #
TITLE			
Title	1	Identify the report as a systematic review, meta-analysis, or both.	01
ABSTRACT			
Structured summary	2	Provide a structured summary including, as applicable: background; objectives; data sources; study eligibility criteria, participants, and interventions; study appraisal and synthesis methods; results; limitations; conclusions and implications of key findings; systematic review registration number.	02
INTRODUCTION			
Rationale	3	Describe the rationale for the review in the context of what is already known.	03
Objectives	4	Provide an explicit statement of questions being addressed with reference to participants, interventions, comparisons, outcomes, and study design (PICOS).	03
METHODS			
Protocol and registration	5	Indicate if a review protocol exists, if and where it can be accessed (e.g., Web address), and, if available, provide registration information including registration number.	03-04
Eligibility criteria	6	Specify study characteristics (e.g., PICOS, length of follow-up) and report characteristics (e.g., years considered, language, publication status) used as criteria for eligibility, giving rationale.	04
Information sources	7	Describe all information sources (e.g., databases with dates of coverage, contact with study authors to identify additional studies) in the search and date last searched.	04
Search	8	Present full electronic search strategy for at least one database, including any limits used, such that it could be repeated.	Suppl material e-Tables 1-3
Study selection	9	State the process for selecting studies (i.e., screening, eligibility, included in systematic review, and, if applicable, included in the meta-analysis).	04
Data collection process	10	Describe method of data extraction from reports (e.g., piloted forms, independently, in duplicate) and any processes for obtaining and confirming data from investigators.	04

For peer review only

Data items	11	List and define all variables for which data were sought (e.g., PICOS, funding sources) and any assumptions and simplifications made.	04
Risk of bias in individual studies	12	Describe methods used for assessing risk of bias of individual studies (including specification of whether this was done at the study or outcome level), and how this information is to be used in any data synthesis.	04-05
Summary measures	13	State the principal summary measures (e.g., risk ratio, difference in means).	05
Synthesis of results	14	Describe the methods of handling data and combining results of studies, if done, including measures of consistency (e.g., I^2) for each meta-analysis.	05
Risk of bias across studies	15	Specify any assessment of risk of bias that may affect the cumulative evidence (e.g., publication bias, selective reporting within studies).	NA
Additional analyses	16	Describe methods of additional analyses (e.g., sensitivity or subgroup analyses, meta-regression), if done, indicating which were pre-specified.	05
RESULTS			
Study selection	17	Give numbers of studies screened, assessed for eligibility, and included in the review, with reasons for exclusions at each stage, ideally with a flow diagram.	05 and Figure 1
Study characteristics	18	For each study, present characteristics for which data were extracted (e.g., study size, PICOS, follow-up period) and provide the citations.	05 and Table 1
Risk of bias within studies	19	Present data on risk of bias of each study and, if available, any outcome level assessment (see item 12).	Suppl Material eTable 5
Results of individual studies	20	For all outcomes considered (benefits or harms), present, for each study: (a) simple summary data for each intervention group (b) effect estimates and confidence intervals, ideally with a forest plot.	06 and Suppl Material e-Table 6
Synthesis of results	21	Present results of each meta-analysis done, including confidence intervals and measures of consistency.	06 and Tables
Risk of bias across studies	22	Present results of any assessment of risk of bias across studies (see Item 15).	NA
Additional analysis	23	Give results of additional analyses, if done (e.g., sensitivity or subgroup analyses, meta-regression [see Item 16]).	06 and Suppl Material e-Figure 1-4

For peer review only

DISCUSSION			
Summary of evidence	24	Summarize the main findings including the strength of evidence for each main outcome; consider their relevance to key groups (e.g., healthcare providers, users, and policy makers).	06
Limitations	25	Discuss limitations at study and outcome level (e.g., risk of bias), and at review-level (e.g., incomplete retrieval of identified research, reporting bias).	07
Conclusions	26	Provide a general interpretation of the results in the context of other evidence, and implications for future research.	07-08
FUNDING			
Funding	27	Describe sources of funding for the systematic review and other support (e.g., supply of data); role of funders for the systematic review.	01

For peer review only

E-Table 5: Coverage and conditions included in the definition of multimorbidity

First author	Study coverage	Number of conditions	Conditions included in the definition of multimorbidity
Andrade [1]	Sub-national	8	Back pain, hypertension, asthma, headache, heart problems, diabetes, stroke, and cancer
Andrade [1]	Sub-national	15	Cognitive impairment, non-affective psychosis, panic attacks, generalized anxiety, any phobias, obsessive-compulsive disorders, depression, dysthymia, bipolar disorder, bulimia, alcohol use disorder, substance use disorder, nicotine dependence, somatoform disorder, and dissociative disorder
Boing [2]	Sub-national	11	Problems in the spine or on the back, arthritis or rheumatism, cancer, diabetes, bronchitis or asthma, hypertension, heart disease, chronic renal failure, tuberculosis, tendinitis or tenosynovitis, and cirrhosis
De Souza Santos Machado [3]	Sub-national	5	Depression, hypertension, diabetes mellitus, urinary incontinence, and insomnia
Aguiar [4]	Sub-national	10	Hypertension, myocardial infarction, stroke, diabetes, pulmonary emphysema, bronchitis/asthma, arthrosis, osteoporosis, cataract, and urinary incontinence
Nunes [5]	Sub-national	17	High blood pressure, diabetes, lung problem, heart problem, stroke, rheumatism, arthritis or arthrosis, disease in spinal column (any problem reported), cancer, kidney problem, cognitive impairment, depression, urinary incontinence, amputation in any part of the body, eyesight problem, hearing problem, problem chewing food, and falls
Agrawal [6]	National	9	Angina pectoris, arthritis, asthma, chronic lung disease, diabetes, hypertension, stroke, depression, and low visual acuity
Nunes [7]	Sub-national	11	High blood pressure, depression, heart disease, hypercholesterolemia, diabetes, bronchitis, asthma/wheezy bronchitis, hypothyroidism, osteoporosis, joint problems, and emphysema
Valadares [8]	Sub-national	11	Hypertension, diabetes mellitus, dyslipidemia, myocardial infarction, stroke, deep venous thrombosis or pulmonary embolus, asthma or bronchitis, chronic rheumatic diseases, depression, anxiety, and cancer
Bustos-Vazquez [9]	National	7	Cancer, systemic arterial hypertension, diabetes, depression, cerebral infarction, hypercholesterolemia, and cardiovascular diseases
Cavalcanti [10]	Sub-national	8	Systemic arterial hypertension, diabetes mellitus, rheumatism, pulmonary problems (asthma, bronchitis and pulmonary emphysema), stroke/cerebral ischemia, arthritis/osteoporosis, heart problems (coronary disease, angina, congestive disease, others), and depression

For peer review only

Nunes [11]	National	22	High blood pressure, spinal column problem, hypercholesterolemia, depression, diabetes, arthritis/rheumatism, asthma/wheezy bronchitis, cancer, other heart disease, work-related muscle-skeletal disorders, stroke, heart attack, kidney problem, heart failure, bronchitis, angina, emphysema, other lung disease, bipolar disorder, other mental disease, schizophrenia, and obsessive-compulsive disorder
Nunes [12]	Sub-national	17	Systemic arterial hypertension, diabetes mellitus, heart disease, pulmonary problems, stroke, rheumatism/arthritis/osteoarthritis, spine problems, cancer, kidney failure, cognitive deficit, depression, visual problems, urinary incontinence, amputation, auditory problems, problem or difficulty chewing food, and falls
Olivares [13]	Sub-national	9	Hypertension, diabetes, high cholesterol, AMI or stroke, asthma, hypothyroidism, celiac disease, cancer and other chronic diseases (arthritis, osteoarthritis, osteoporosis, depression, chronic allergies, COPD; gastritis, migraine, irritable bowel syndrome, epilepsy, psychosis, multiple sclerosis, psoriasis, gout disease, herniated disc, inflammatory vasculitis)
Taype-Rondan [14]	Sub-national	6	Hypertension, chronic bronchitis, depressive mood, type 2 diabetes, heart disease, and stroke
Amaral [15]	Sub-national	8	Hypertension, diabetes, cardiovascular disorder (poor circulation, cerebrovascular accident (CVA or stroke) and heart problem), musculoskeletal disorder (spinal problem, rheumatism, arthritis/ arthrosis and osteoporosis), insomnia, cataracts, anemia and gastrointestinal/urinary disorders
Araujo [16]	Sub-national	12	Hypertension, diabetes, high cholesterol, heart disease (heart attack, angina, heart failure or other), stroke, asthma or asthmatic bronchitis, arthritis or rheumatism, depression, pulmonary disease (pulmonary emphysema, chronic bronchitis or chronic obstructive pulmonary disease), cancer, chronic kidney disease, and chronic spinal problems (chronic back or neck pain, low back pain, sciatic pain, vertebral or disc problems)
Camargo-Casas [17]	Sub-national	12	Hypertension, diabetes, cancer, chronic obstructive pulmonary disease, heart attack, heart failure, stroke, arthritis or osteoarthritis, osteoporosis, gastroesophageal reflux disease, gastritis, and ulcer
Costa [18]	Sub-national	29	High blood pressure, diabetes, heart problem, heart failure, asthma, bronchitis, emphysema, arthritis, Parkinson's disease, kidney failure, hypercholesterolemia, seizure, stomach ulcer, osteoporosis, urinary incontinence, constipation, fecal incontinence, depression, glaucoma, deafness, difficulty swallowing, insomnia, fainting, rhinitis, difficulty speaking, stroke, obesity, mental disorders, and cancer
Nunes [19]	National	19	Systemic arterial hypertension, back problems, high cholesterol, cataract, arthritis or rheumatism, depression, diabetes, osteoporosis heart problem, glaucoma, emphysema

For peer review only

			chronic bronchitis or chronic obstructive pulmonary disease, cerebrovascular accident, cancer, asthma, chronic renal failure, diabetic retinopathy, macular degeneration, Parkinson's disease, and Alzheimer's disease
Bao [20]	National	15	Vision, hearing, joints, skin, gastrointestinal, stroke, chronic obstructive pulmonary disease, hypertension, diabetes, ischemic heart disease, heart failure, heart valve disease, depression, anxiety, and dementia
Macinko [21]	National	8	Arthritis, asthma, cancer, depression, diabetes, heart disease, high cholesterol, and hypertension
Miranda [22]	Sub-national	6	Alcohol disorder, asthma, chronic obstructive pulmonary disease, depression, diabetes, and hypertension
Petarli [23]	Sub-national	20	Heart arrhythmia, infarction, stroke, diabetes mellitus, herniated disk, arthrosis, repetitive strain injuries/work-related musculoskeletal disorders, renal disease, Parkinson's, Alzheimer's, hepatic cirrhosis, infertility, cancer, asthma, chronic bronchitis, and pulmonary emphysema, arterial hypertension, dyslipidemia, depression and thyroid disorders
Tavares [24]	Sub-national	25	Rheumatism, arthritis/arthrosis, osteoporosis, asthma or bronchitis, tuberculosis, embolism, systemic arterial hypertension, poor circulation (varicose veins), heart problems, diabetes mellitus, obesity, stroke, Parkinson's, urinary incontinence, fecal incontinence, intestinal constipation, sleeping problems, cataract, glaucoma, back problems, kidney problem, accident/trauma sequelae, malignant tumors, benign tumors, and vision problems
Wang [25]	Sub-national	16	Cardiovascular diseases (heart attack, heart disease, and stroke), hypertension, diabetes mellitus, arthritis, chronic musculoskeletal pain, headache or migraine, digestive, respiratory (seasonal allergies, asthma, obstructive pulmonary disease, and emphysema), cancer, neurological diseases (Parkinson disease, epilepsy, and multiple sclerosis), anxiety disorder, mood disorder, heavy drinking, impulse-control disorder, substance use disorder, and premenstrual dysphoria
Montes [26]	Sub-national	28	Systemic arterial hypertension, myocardial infarction, diabetes, heart failure, emphysema, asthma, bronchitis, arthritis, Parkinson's disease, renal failure, seizure, hypercholesterolemia, stomach ulcer, osteoporosis, urinary incontinence, constipation, fecal incontinence, depression, glaucoma, deafness, difficulty swallowing, insomnia, fainting, rhinitis, difficulty speaking, stroke, mental disorders and cancer
Padilha Pereira [27]	Sub-national	28	Arterial hypertension, diabetes, heart problems, heart failure, asthma, bronchitis, emphysema, arthritis, Parkinson's disease, kidney failure, hypercholesterolemia, convulsions, stomach ulcer, osteoporosis, urinary incontinence, constipation, fecal

For peer review only

			incontinence, depression, glaucoma, deafness, difficulty in swallowing, insomnia, fainting, rhinitis, difficulty in speaking, stroke, mental disorders, and cancer
Da Silva Almeida [28]	Sub-national	5	Hypertension, diabetes, dyslipidemias, obesity, and arthritis or rheumatism

For peer review only

E-Table 6: Risk of bias of included studies

First author	Representativeness	Sample size	Non-respondents	Exposure	Outcome	Statistical test
Andrade [1]	Low	Low	High	NA	High	NA
Boing [2]	Low	Low	Low	NA	High	NA
De Souza Santos Machado [3]	Low	Low	Low	NA	High	NA
Aguiar [4]	Low	Low	Low	NA	High	NA
Nunes [5]	Low	Low	Low	NA	High	NA
Agrawal [6]	Low	Low	High	NA	High	NA
Nunes [7]	Low	Low	Low	NA	High	NA
Valadares [8]	Low	Low	Low	NA	High	NA
Bustos-Vazquez [9]	Low	Low	Low	NA	High	NA
Cavalcanti [10]	Low	Low	Low	NA	High	NA
Nunes [11]	Low	Low	High	NA	High	NA
Nunes [12]	Low	Low	Low	NA	High	NA
Olivares [13]	Low	Low	Low	NA	Low	NA
Taype-Rondan [14]	Low	Low	Low	NA	Low	NA
Amaral [15]	Low	Low	High	NA	High	NA
Araujo [16]	Low	Low	Low	NA	High	NA
Camargo-Casas [17]	Low	Low	Low	NA	High	NA
Costa [18]	Low	Low	Low	NA	High	NA
Nunes [19]	Low	Low	Low	NA	High	NA
Bao [20]	Low	High	Low	NA	Low	NA
Macinko [21]	Low	High	High	NA	High	NA
Miranda [22]	Low	High	Low	NA	Low	NA
Petarli [23]	Low	Low	Low	NA	High	NA
Tavares [24]	Low	Low	Low	NA	High	NA
Wang [25]	Low	Low	Low	NA	High	NA
Montes [26]	Low	Low	Low	NA	High	NA
Padilha Pereira [27]	Low	Low	Low	NA	High	NA
Da Silva Almeida [28]	Low	Low	Low	NA	Low	NA

For peer review only

REFERENCES

1. Andrade LH, Benseñor IM, Viana MC, Andreoni S, Wang YP. Clustering of psychiatric and somatic illnesses in the general population: multimorbidity and socioeconomic correlates. *Braz j med biol res* 2010;43(5):483-91.
2. Boing AF, Melo GR, Boing AC, Moretti-Pires RO, Peres KG, Peres MA. Associação entre depressão e doenças crônicas: um estudo populacional. *Rev saúde pública* 2012;46(4):617-23.
3. de Souza Santos Machado V, Valadares ALR, da Costa-Paiva LS, Moraes SS, Pinto-Neto AM. Multimorbidity and associated factors in Brazilian women aged 40 to 65 years: a population-based study. *Menopause (New York, NY)* 2012;19(5):569-75.
4. Aguiar LB, Baccaro LF, de Souza Santos Machado V, Pinto-Neto AM, Costa-Paiva L. Disability and multimorbidity in women older than 50 years: a population-based household survey. *Menopause (New York, NY)* 2015;22(6):660-6.
5. Nunes BP, Thume E, Facchini LA. Multimorbidity in older adults: magnitude and challenges for the Brazilian health system. *BMC public health* 2015;15:1172.
6. Agrawal S, Agrawal PK. Association Between Body Mass index and Prevalence of Multimorbidity in Low-and Middle-income Countries: A Cross-Sectional Study. *International journal of medicine and public health* 2016;6(2):73-83.
7. Nunes BP, Camargo-Figuera FA, Guttier M, de Oliveira PD, Munhoz TN, Matijasevich A, et al. Multimorbidity in adults from a southern Brazilian city: occurrence and patterns. *International journal of public health* 2016;61(9):1013-20.
8. Valadares ALR, Lui-Filho JF, Costa-Paiva L, Pinto-Neto AM. Middle-aged female sexual dysfunction and multimorbidity: a population-based study. *Menopause (New York, NY)* 2016;23(3):304-10.
9. Bustos-Vazquez E, Fernandez-Nino JA, Astudillo-Garcia CI. Self-rated health, multimorbidity and depression in Mexican older adults: Proposal and evaluation of a simple conceptual model. *Biomedica : revista del Instituto Nacional de Salud* 2017;37(0):92-103.
10. Cavalcanti G, Doring M, Portella MR, Bortoluzzi EC, Mascarelo A, Dellani MP. Multimorbidity associated with polypharmacy and negative self-perception of health. *Rev bras geriatr gerontol (Online)* 2017;20(5):634-42.
11. Nunes BP, Chiavegatto Filho ADP, Pati S, Cruz Teixeira DS, Flores TR, Camargo-Figuera FA, et al. Contextual and individual inequalities of multimorbidity in Brazilian adults: a cross-sectional national-based study. *BMJ open* 2017;7(6):e015885.
12. Nunes BP, Soares MU, Wachs LS, Volz PM, Saes MdO, Duro SMS, et al. Hospitalization in older adults: association with multimorbidity, primary health care and private health plan. *Rev saúde pública* 2017;51:43-.
13. Olivares DEV, Chambi FRV, Chani EMM, Craig WJ, Pacheco SOS, Pacheco FJ. Risk Factors for Chronic Diseases and Multimorbidity in a Primary Care Context of Central Argentina: A Web-Based Interactive and Cross-Sectional Study. *International journal of environmental research and public health* 2017;14(3).
14. Taype-Rondan A, Abbs ES, Lazo-Porras M, Checkley W, Gilman RH, Smeeth L, et al. Association between chronic conditions and health-related quality of life: differences by level of urbanization in Peru. *Quality of life research : an international journal of quality of life aspects of treatment, care and rehabilitation* 2017;26(12):3439-47.

For peer review only

15. Amaral TLM, Amaral CdA, Lima NSd, Herculano PV, Prado PRd, Monteiro GTR. Multimorbidity, depression and quality of life among elderly people assisted in the Family Health Strategy in Senador Guiomard, Acre, Brazil. *Multimorbidade, depressao e qualidade de vida em idosos atendidos pela Estrategia de Saude da Familia em Senador Guiomard, Acre, Brasil* 2018;23(9):3077-84.
16. Araujo MEA, Silva MT, Galvao TF, Nunes BP, Pereira MG. Prevalence and patterns of multimorbidity in Amazon Region of Brazil and associated determinants: a cross-sectional study. *BMJ open* 2018;8(11):e023398.
17. Camargo-Casas S, Suarez-Monsalve S, Zepeda MUP, Garcia-Pena C, Cano-Gutierrez CA. [Multimorbidity, Depressive Symptoms, and Self-Reported Health in Older Adults: a Secondary Analysis of the Sabe Bogota Study]. *Revista de investigacion clinica; organo del Hospital de Enfermedades de la Nutricion* 2018;70(4):192-7.
18. Costa CdS, Flores TR, Wendt A, Neves RG, Tomasi E, Cesar JA, et al. Inequalities in multimorbidity among elderly: a population-based study in a city in Southern Brazil. *Cad Saúde Pública (Online)* 2018;34(11):e00040718-e.
19. Nunes BP, Batista SRR, Andrade FBd, Souza Junior PRBd, Lima-Costa MF, Facchini LA. Multimorbidity: The Brazilian Longitudinal Study of Aging (ELSI-Brazil). *Revista de saude publica* 2018;52Suppl 2(Suppl 2):10s.
20. Bao J, Chua K-C, Prina M, Prince M. Multimorbidity and care dependence in older adults: a longitudinal analysis of findings from the 10/66 study. *BMC public health* 2019;19(1):585.
21. Macinko J, Andrade FCD, Nunes BP, Guanais FC. Primary care and multimorbidity in six Latin American and Caribbean countries. *Rev panam salud pública* 2019;43:e8-e.
22. Miranda JJ, Bernabe-Ortiz A, Gilman RH, Smeeth L, Malaga G, Wise RA, et al. Multimorbidity at sea level and high-altitude urban and rural settings: The CRONICAS Cohort Study. *Journal of Comorbiddity* 2019;9:1-10.
23. Petarli GB, Cattafesta M, Sant'Anna MM, Bezerra OMdPA, Zandonade E, Salaroli LB. Multimorbidity and complex multimorbidity in Brazilian rural workers. *PLoS ONE* 2019;14(11):e0225416.
24. Tavares DMDS, Pelizaro PB, Pegorari MS, Paiva MMd, Marchiori GF. Prevalence of self-reported morbidities and associated factors among community-dwelling elderly in Uberaba, Minas Gerais, Brazil. *Prevalencia de morbidades autorreferidas e fatores associados entre idosos comunitarios de Uberaba, Minas Gerais, Brasil* 2019;24(9):3305-13.
25. Wang Y-P, Nunes BP, Coelho BM, Santana GL, do Nascimento CF, Viana MC, et al. Multilevel Analysis of the Patterns of Physical-Mental Multimorbidity in General Population of Sao Paulo Metropolitan Area, Brazil. *Scientific reports* 2019;9(1):2390.
26. Montes MC, Bortolotto CC, Tomasi E, Gonzalez MC, Barbosa-Silva TG, Domingues MR, et al. Strength and multimorbidity among community-dwelling elderly from southern Brazil. *Nutrition* 2020;71.
27. Padilha Pereira B, Cardozo Bortolotto C, Tomasi E, Gonzalez MC, Gomes AP, Goncalves H, et al. Food consumption and multimorbidity among non-institutionalized elderly people in Pelotas, 2014: a cross-sectional study. *Epidemiol serv saúde* 2020;29(3):e2019050.
28. da Silva Almeida IL, dos Santos SR, Morbeck de Queiroz B, de Freitas Mussi RF. Lifestyle, morbidity and multimorbidity in adult Quilombolas. *ABCS health sci* 2020;45:1325.

For peer review only
