# Supplementary material for: Multimorbidity in Latin America and the Caribbean: a systematic review and meta-analysis
Source: BMJ Open. 2021 Jul 23;11(7):e050409. doi: 10.1136/bmjopen-2021-050409 (PMC8311299; doi:10.1136/bmjopen-2021-050409)
Supplement: Reviewer comments [file bmjopen-2021-050409.reviewer_comments.pdf]

PEER REVIEW HISTORY

BMJ Open publishes all reviews undertaken for accepted manuscripts. Reviewers are asked to complete a checklist review form (**http://bmjopen.bmj.com/site/about/resources/checklist.pdf**) and are provided with free text boxes to elaborate on their assessment. These free text comments are reproduced below.

ARTICLE DETAILS

**TITLE (PROVISIONAL)**	Multimorbidity in Latin America and the Caribbean: A systematic review and meta-analysis
**AUTHORS**	Huaquía-Díaz, Alessandra; Chalán-Dávila, Tarick; Carrillo-Larco, Rodrigo; Bernabe-Ortiz, Antonio

VERSION 1 – REVIEW

**REVIEWER**	Maria Lorenza Muiesan<br>University of Brescia
**REVIEW RETURNED**	25-Apr-2021

**GENERAL COMMENTS**	Authors have focused their attention on the prevalence of multimorbidity (as “the existence of two or more medical chronic conditions in a single individual”) in studies performed in Latin American and Caribbean regions. Authors have taken into account 26 studies , although most of them are reporting data in Brazil e fewer in other nations of South America (2 putting together several countries) , only 2 from Mexico , 2 from Peru, 1 from Colombia and 1 from Argentina .<br><br><br>Major comments<br>The issue is of importance. Methods are clearly described, I would like more details on results.<br><br>Most studies report data from Brazil and this represents the main limitation , but also the boost for collecting new data in other nations (mostly Argentina , Peru , Colombia and Caribbean Islands)<br><br>Since in most studies the definition of chronic condition was provided, I would suggest to report the more frequent clusters of multimorbidities (i.e. hypertension & diabetes ? hypertension and obesity ?) at least in a smaller number of studies.<br>Could authors compare Brazil (putting together all studies coming from Brazil, despite their heterogeneity) to other nations of South America and to multinational studies ?

**REVIEWER**	Prince Peprah<br>Kwame Nkrumah University of Science and Technology
**REVIEW RETURNED**	28-Apr-2021

**GENERAL COMMENTS**	I do not really understand what the authors mean by ‘their results’<br>I do not quite follow this phrase ‘which prevent to better’<br>I think the paper needs proof-reading by someone with English background. This is because the construction is very hard to follow especially the punctuation.<br>The authors should expand their discussion. It its current form the

	findings are not adequately and properly discussed<br>The summary of evidence heading should be removed.

**REVIEWER**	Chifa Chiang<br>Nagoya University Graduate School of Medicine Faculty of Medicine, Public Health and Health Systems
**REVIEW RETURNED**	30-Apr-2021

**GENERAL COMMENTS**	The study aims to estimate the pooled prevalence of multimorbidity among general adult population in Latin America the Caribbean. This is an important topic and findings of the study might add to the existing literature; however, there are several critical insufficiencies in this manuscript have to be addressed.<br><br>#1 OVERALL<br>In epidemiology, prevalence is commonly defined as a disease or a risk factor at a specific time. However, this study pooled data collected between 1994 and 2016 in the region.<br>The world has been going through a huge health transition especially in low- and middle-income countries, including Latin America the Caribbean, over the past 20-30 years. Prevalence of non-communicable diseases today is much different from, let's say, year of 2000. Why did the authors have a study design to pool prevalence of multimorbidity over a period of >20 years? Please rationalize it!<br><br>#2 OVERALL<br>As the authors mentioned in the Discussion section, a great proportion of studies enrolled individuals aged >=50 years in the systematic review. The limitation makes the study design difficult to represent the general population aged >=18 years. Analyses stratified by age or limited to the older population might be required.<br><br>#3 OVERALL (E-Table 5)<br>Conditions included in the definition of multimorbidity were ranged from 5 to 29 in number. Although all of the studies might use the same term of multimorbidity or comorbidity, they were very different study designs. Why those various studies can be pooled? Please rationalize it!<br><br>#4 OVERALL<br>Most of the studies (20 out of 28) were conducted in Brazil. Since the systematic review tried to cover entire region of Latin America the Caribbean, a stratified analysis (Brazil vs others) or sensitivity analysis is required.<br><br>#5 Table 1<br>Please include mean or median age of each study in addition to the age range in Table 1.

VERSION 1 – AUTHOR RESPONSE

Reviewer 1: Dr. Maria Lorenza Muiesan, University of Brescia

Comments to the Author:

Authors have focused their attention on the prevalence of multimorbidity (as "the existence of two or more medical chronic conditions in a single individual") in studies performed in Latin American and

Caribbean regions. Authors have taken into account 26 studies, although most of them are reporting data in Brazil and fewer in other nations of South America (2 putting together several countries), only 2 from Mexico, 2 from Peru, 1 from Colombia, and 1 from Argentina.

Major comments:

The issue is of importance. Methods are clearly described. I would like more details on results.

We have added some information in the results sections as requested regarding the comparison between Brazil and other LAC countries, as well as an analysis regarding age group, focused on those aged 50+ years (as suggested by other reviewer).

Most studies report data from Brazil and this represents the main limitation, but also the boost for collecting new data in other nations (mostly Argentina, Peru, Colombia and Caribbean Islands).

We have a sentence regarding this point in the Limitations section. This sentence reads:

"Third, most of the studies included in the review were from Brazil, preventing inferability to the whole region, but also highlighting the need of population-based studies on multimorbidity in other countries of the region."

Since in most studies the definition of chronic condition was provided, I would suggest to report the more frequent clusters of multimorbidities (i.e., hypertension & diabetes? hypertension and obesity?) at least in a smaller number of studies. Could authors compare Brazil (putting together all studies coming from Brazil, despite their heterogeneity) to other nations of South America and to multinational studies?

We agree with the reviewer regarding this point. However, whereas the list of conditions is detailed, there are not estimations regarding the combination of clusters of multimorbidities in the manuscripts assessed. Thus, we will require the prevalence of each of the clusters of interest and this is not provided by the papers. This is a very relevant topic we have discussed in the limitations of the review:

"Second, the number of chronic conditions as well as the list of them to define multimorbidity is very dissimilar. Defining specific clusters of multimorbidity is needed to guarantee appropriate comparability between studies, but this is not usually reported. Thus, it is relevant to standardize the definition of multimorbidity and the conditions included in such definition to estimate which clusters of multimorbidity are more frequent."

We have, however, added the requested comparison between Brazil to other nations of LAC region, and added the results in the manuscript:

"As many of the studies were from Brazil, the pooled estimate of multimorbidity (≥2 chronic conditions) for this country was 50% (95% CI: 37% - 63%; I2: 99.9%), whereas the pooled estimate for other countries together was 35% (95% CI: 26% - 43%; I2: 99.7%)."

In addition, we have added, as requested by other reviewer, the pooled estimate of multimorbidity among those studies including individuals aged 50+ years. We have added this in the results section:

“When assessing only studies including subjects of 50 years and over, the pooled prevalence of multimorbidity was 62% (95% CI: 51% - 73%; I2: 99.9%).”

Reviewer 2: Dr. Prince Peprah, Kwame Nkrumah University of Science and Technology

Comments to the Author:

I do not really understand what the authors mean by ‘their results’

We have reviewed the sections of the papers to clarify these points and typos.

I do not quite follow this phrase ‘which prevent to better’

We have modified this sentence.

I think the paper needs proof-reading by someone with English background. This is because the construction is very hard to follow especially the punctuation.

The paper has been proof-read by a native-English speaker as suggested.

The authors should expand their discussion. It its current form the findings are not adequately and properly discussed.

We have expanded our discussion according to other reviewers’ suggestions and comments. The structure of the Discussion section is based on the PRISMA checklist as suggested by the rules of the journal.

The summary of evidence heading should be removed.

We have followed the PRISMA toolkit for reporting and the sections of the Discussion are those detailed in the checklist used.

Reviewer 3: Dr. Chifa Chiang, Nagoya University Graduate School of Medicine Faculty of Medicine
Comments to the Author:

The study aims to estimate the pooled prevalence of multimorbidity among general adult population in Latin America the Caribbean. This is an important topic and findings of the study might add to the existing literature; however, there are several critical insufficiencies in this manuscript have to be addressed.

In epidemiology, prevalence is commonly defined as a disease or a risk factor at a specific time. However, this study pooled data collected between 1994 and 2016 in the region. The world has been going through a huge health transition especially in low- and middle-

income countries, including Latin America the Caribbean, over the past 20-30 years. Prevalence of non-communicable diseases today is much different from, let's say, year of 2000. Why did the authors have a study design to pool prevalence of multimorbidity over a period of >20 years? Please rationalize it!

We agree with the reviewer regarding the definition of prevalence. However, our study aimed at estimating the pooled prevalence of multimorbidity using a systematic review approach. The idea behind is not only to have an estimate but also to highlight the concerns about such indicator. Thus, a limited number of papers determining the prevalence of multimorbidity have been found using a systematic approach. Moreover, despite of low risk of bias, studies are very heterogenous, not only because the time framework used, but also because definitions of multimorbidity and age groups included in the review are very dissimilar.

As this is a systematic review, we tried to include all the papers found with a similar definition (2+ chronic conditions) despite of different list of conditions assessed. To address the reviewer concern, we have conducted a sensitivity analysis by including only studies which data was collected from 2010 and onwards. We have added that in the Results section:

"When analysis was conducted using information collected from 2010 and onwards, as time can have an impact on estimation due to the health transition in LMIC, pooled prevalence of multimorbidity was 48% (95% CI: 34% - 61%; I^2: 99.9%)".

As the authors mentioned in the Discussion section, a great proportion of studies enrolled individuals aged >=50 years in the systematic review. The limitation makes the study design difficult to represent the general population aged >=18 years. Analyses stratified by age or limited to the older population might be required.

We have added this information as requested also by another reviewer. These results have been added to the Results section:

"Similarly, when assessing only studies including subjects of 50 years and over, the pooled prevalence of multimorbidity was 62% (95% CI: 51% - 73%; I^2: 99.9%)."

Conditions included in the definition of multimorbidity were ranged from 5 to 29 in number. Although all of the studies might use the same term of multimorbidity or comorbidity, they were very different study designs. Why those various studies can be pooled? Please rationalize it!

We agree with the reviewer regarding this point. Studies may be pooled because they have used the same definition of multimorbidity (two or more conditions, regardless which diseases these were). Nevertheless, as claimed by the reviewer, this approximation has to pay a price, the high heterogeneity in the results. We have added that in the discussion section as part of the limitations of the review:

"Second, the number of chronic conditions as well as the list of them to define multimorbidity is very dissimilar. Defining clusters of multimorbidity is needed to guarantee comparability between studies, but this is not usually reported. Thus, it is relevant to standardize the definition of multimorbidity and the conditions included in such definition to estimate which clusters of multimorbidity are more frequent."

Most of the studies (20 out of 28) were conducted in Brazil. Since the systematic review tried to cover entire region of Latin America the Caribbean, a stratified analysis (Brazil vs others) or sensitivity analysis is required.

We have added the requested comparison between Brazil to other nations of LAC region, and added the results in the manuscript:

"As many of the studies were from Brazil, the pooled estimate of multimorbidity (≥2 chronic conditions) for this country was 50% (95% CI: 37% - 63%; I2: 99.9%), whereas the pooled estimate for other countries together was 35% (95% CI: 26% - 43%; I2: 99.7%)."

Table 1

Please include mean or median age of each study in addition to the age range in Table 1.

Information has been included as requested.

VERSION 2 – REVIEW

**REVIEWER**	Maria Lorenza Muiesan<br>University of Brescia
**REVIEW RETURNED**	30-Jun-2021

**GENERAL COMMENTS**	I do not have additive comments. Authors responded appropriately.

**REVIEWER**	Prince Peprah<br>Kwame Nkrumah University of Science and Technology
**REVIEW RETURNED**	26-Jun-2021

**GENERAL COMMENTS**	Congratulations to the authors for addressing the review comments

**REVIEWER**	Chifa Chiang<br>Nagoya University Graduate School of Medicine Faculty of Medicine, Public Health and Health Systems
**REVIEW RETURNED**	06-Jul-2021

**GENERAL COMMENTS**	#1 The study design is appropriate for a systemic review but may not appropriate for a meta-analysis.<br>Very high heterogeneity (I2 >99%) was shown among each original study in both the pooled analyses and sub-group analyses. As shown in Figure 2, the prevalence of multimorbidity ranges from approx. 15% to approx. 95%, and it seems well distributed on both side of the forest plot. It is no surprise that if we have a pooled analysis with such high heterogeneity, we will always have a pooled prevalence equal to 40-50%. Accordingly, it is risky to apply the findings to public health or clinical settings.<br><br>#2 Authors have added the results of "sensitivity analysis" in the text of the revised manuscript. Since the heterogeneity of the pooled prevalence was very high, sub-group analysis (sensitivity analysis) is highly required to ascertain what caused the heterogeneity. Please have a table summarizing the results of the sub-group (sensitivity) analyses. This is a major part of the current study.<br><br>#3 In the STRENGHTS AND LIMITATIONS OF THIS STUDY, authors have stated that high heterogeneity was mainly due to the

	variety of multimorbidity definition and the age range. Is there any evidence to support this statement in the current study?<br><br>#4 As stated in the STRENGHTS AND LIMITATIONS OF THIS STUDY, there are significant limitations in the current meta-analysis. If the heterogeneity could not be deal with, removing the meta-analysis from the systemic review might be one of the options.<br><br>#5 Please add standard deviations of age after mean ages in Table 1, if available.

VERSION 2 – AUTHOR RESPONSE

Reviewer 3:

Dr. Chifa Chiang, Nagoya University Graduate School of Medicine Faculty of Medicine

Comments to the Author:

#1 The study design is appropriate for a systemic review but may not appropriate for a meta-analysis. Very high heterogeneity (I2 >99%) was shown among each original study in both the pooled analyses and sub-group analyses. As shown in Figure 2, the prevalence of multimorbidity ranges from approx. 15% to approx. 95%, and it seems well distributed on both side of the forest plot. It is no surprise that if we have a pooled analysis with such high heterogeneity, we will always have a pooled prevalence equal to 40-50%. Accordingly, it is risky to apply the findings to public health or clinical settings.

Meta-analysis can be done even if heterogeneity is as high as 100%. Although some authors suggest the need of deal with heterogeneity, this is not always possible. We have verified our data for potential mistakes and explore heterogeneity with data extracted (see meta-regression and sensitivity analysis as suggested by reviewers).

For dealing with this problem, we have conducted a random effect meta-analysis, using this approach as heterogeneity was not explained for the study characteristics. We believe meta-analysis (and the forest plot) is needed not only to show a pooled estimate but also to highlight how heterogeneous results were. Perhaps, the editor may decide on this point.

#2 Authors have added the results of "sensitivity analysis" in the text of the revised manuscript. Since the heterogeneity of the pooled prevalence was very high, sub-group analysis (sensitivity analysis) is highly required to ascertain what caused the heterogeneity. Please have a table summarizing the results of the sub-group (sensitivity) analyses. This is a major part of the current study.

A sensitivity and subgroup analysis were added according to reviewers' suggestion in our previous version of the manuscript. Despite of our sensitivity analysis (see Table below), heterogeneity was not reduced. Heterogeneity is only reduced when the analysis was stratified by setting (urban vs. rural).

Meta-analysis	Pooled estimates (95%	Heterogeneity

	CI)	(I^2)
**Overall results**		
MM (2+ conditions)	43% (35% - 51%)	99.9%
MM (3+ conditions)	40% (22% - 57%)	99.9%
**Sensitivity analysis (MM: 2+ conditions)**		
MM definition		
Only self-reported	40% (31% - 48%)	99.9%
Measured and self-reported	52% (33% - 70%)	99.9%
Coverage		
National	37% (27% - 47%)	99.9%
Non-national	48% (35% - 61%)	99.9%
Age		
50+ years	62% (51% - 73%)	99.9%
60+ years	62% (49% - 75%)	99.9%
Year		
From 2010 and onwards	48% (34% - 61%)	99.9%
From 2015 and onwards	44% (24% - 65%)	99.8%
Country		
Brazil	50% (37% - 63%)	99.9%
Other countries	35% (26% - 43%)	99.7%
**Sub-group analysis**		
By sex		
Males	39% (29% - 49%)	99.7%
Females	51% (38% - 63%)	99.8%
By setting		
Urban	38% (26% - 50%)	87.3%

Rural	25% (13% - 37%)	50.8%

#3 In the STRENGHTS AND LIMITATIONS OF THIS STUDY, authors have stated that high heterogeneity was mainly due to the variety of multimorbidity definition and the age range. Is there any evidence to support this statement in the current study?

That statement is based on our meta-regression analysis. We have added that information in the Results section to support our limitation:

"In meta-regression analysis, the number of chronic conditions defining multimorbidity was strongly associated with heterogeneity (β: 0.02 per additional condition, p <0.001). Similarly, mean age was also strongly associated (β: 0.01 per additional year in the mean age, p <0.001) and the proportion of women involved in the study (β: 0.75, p = 0.008). In addition, setting (urban vs. rural) was almost associated with heterogeneity (β: 0.59 compared to rural settings, p = 0.06)."

#4 As stated in the STRENGHTS AND LIMITATIONS OF THIS STUDY, there are significant limitations in the current meta-analysis. If the heterogeneity could not be deal with, removing the meta-analysis from the systemic review might be one of the options.

Unfortunately, heterogeneity could not be dealt with our sensitivity analyses; however, we believe that statistical techniques used (i.e., random effect meta-analysis) may be enough to show pooled estimates despite of the problem of heterogeneity. Perhaps the editor may help to take an appropriate decision regarding this point.

#5 Please add standard deviations of age after mean ages in Table 1, if available.

This information was not extracted during the review process. Usually, only mean age and age range is only extracted.

VERSION 3 – REVIEW

**REVIEWER**	Chifa Chiang<br>Nagoya University Graduate School of Medicine Faculty of Medicine, Public Health and Health Systems
**REVIEW RETURNED**	09-Jul-2021

**GENERAL COMMENTS**	#1 The quality of the manuscript has been improved a lot after the revision.<br>#2 Some of the results for the sensitivity analysis shown in the table (pooled prevalence of "60+ years" and "from 2015 and onwards"), provided in the response letter, were not included in the manuscript. Please add them into a revised manuscript and revise the Methods section accordingly.

VERSION 3 – AUTHOR RESPONSE

Reviewer 3:

Dr. Chifa Chiang, Nagoya University Graduate School of Medicine Faculty of Medicine

Comments to the Author:

#1 The quality of the manuscript has been improved a lot after the revision.

Thanks for your helpful comments to improve our manuscript.

#2 Some of the results for the sensitivity analysis shown in the table (pooled prevalence of "60+ years" and "from 2015 and onwards"), provided in the response letter, were not included in the manuscript. Please add them into a revised manuscript and revise the Methods section accordingly.

We have added those results as requested by the reviewer. In the Summary Measures section of the Methods, we have added: "Sensitivity analyses were also conducted focused on age by including studies with individuals aged 50+ and 60+ years, and also with studies whose data was collected from 2010 and onwards, as well as 2015 and onwards."

In the same line, we have added the estimates in the Results section as follows: "On the other hand, when analysis was conducted using studies whose data was collected from 2010 and onwards, pooled prevalence of multimorbidity was 48% (95% CI: 34% - 61%; I2: 99.9%), whereas this estimate was 44% (95% CI: 24% - 65%, I2: 99.8%)", and "Similarly, when assessing only studies including subjects of 50 years and over, the pooled prevalence of multimorbidity was 62% (95% CI: 51% - 73%; I2: 99.9%), and there was no variation when the pooled prevalence was estimated for subject of 60 years and over: 62% (95% CI: 49% - 75%; I2: 99.9%)."
